# New Inhibitors of Neuronal Nitric Oxide Synthase for the Treatment of Melanoma

**DOI:** 10.1021/acs.jmedchem.5c02154

**Published:** 2026-01-30

**Authors:** Amardeep Awasthi, Anika Patel, Huiying Li, Koon Mook Kang, Christine D. Hardy, Anas Ansari, Raghad Nowar, Md. Emtiaz Hasan, Sun Yang, Thomas L. Poulos, Richard B. Silverman

**Affiliations:** † Department of Chemistry, Chemistry of Life Processes Institute, Center for Developmental Therapeutics, 3270 Northwestern University, 2145 Sheridan Road, Evanston, Illinois 60208-3113, United States; ‡ Department of Pharmacy Practice, 6226 Chapman University School of Pharmacy, Harry and Diane Rinker Health Science Campus, 9401 Jeronimo Road, Irvine, California 92618, United States; § Departments of Molecular Biology and Biochemistry, Pharmaceutical Sciences, and Chemistry, 8788 University of California, Irvine, California 92697-3900, United States; ∥ Department of Molecular Biosciences, 3270 Northwestern University, Evanston, Illinois 60208, United States; ⊥ Department of Pharmacology, Feinberg School of Medicine, 3270 Northwestern University, Chicago, Illinois 60611, United States

## Abstract

In 2024, an estimated 100,640 new cases of invasive melanoma were diagnosed in the U.S., with 9290 deaths. Our previous studies revealed that neuronal nitric oxide synthase (nNOS) derived nitric oxide plays a critical role in melanoma progression, making nNOS inhibition a promising strategy. High structural similarity among NOS isoforms requires careful design of nNOS inhibitors to avoid off-target effects. Our previous lead, HH044, demonstrated potent antimelanoma activity but exhibited only moderate nNOS selectivity. Here, we utilized a structure-based approach to design nNOS inhibitors that promote interactions with human nNOS-specific residue His342. Compound **9** exhibited inhibition of both human (*K*_i_ = 1.7 nM) and rat nNOS (*K*_i_ = 2.3 nM), with 5654-fold selectivity over human eNOS and 250-fold selectivity over iNOS. X-ray crystallography and molecular modeling revealed a novel SAR, forming the basis for nNOS inhibition and providing a foundation for further innovative design of nNOS inhibitors for melanoma treatment.

## Graphical abstract

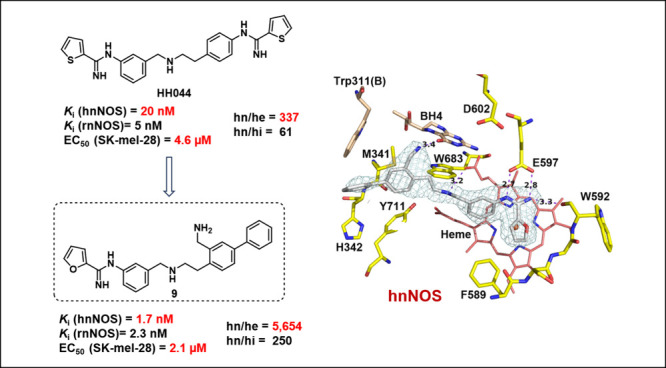

## Introduction

Human invasive melanoma poses a significant public health concern due to its high fatality rate and resistance to classical chemotherapy.^1^ Cutaneous melanoma had 97,610 new cases in 2023 in the U.S., which were estimated to increase to 100,640 in 2024.^2^ Despite significant advancements in melanoma treatment over the past decade, substantial challenges remain, particularly in managing advanced-stage disease.^3^ Immunotherapies blocking immune checkpoints, such as PD-1/PD-L1 and CTLA-4, have revolutionized melanoma management by enhancing the antitumor immune response. However, not all patients respond favorably, with a substantial proportion exhibiting primary or acquired resistance. Furthermore, the high incidence of immunotherapy-related adverse events has restricted the patient population that can benefit from this treatment.^4^ In addition, the rapid development of drug resistance due to the emergence of mutations has also limited the clinical effectiveness of targeted therapy.^3^ Therefore, there remains an urgent unmet medical need for developing novel and effective treatments for melanoma.

Nitric oxide synthases (NOSs) catalyze the conversion of l-arginine to L-citrulline in the presence of oxygen and NADPH, resulting in the production of nitric oxide (NO).^5–8^ Melanocytes are derived from the neural crest and, therefore, exhibit gene expression characteristics similar to those of neural cells. Earlier studies showed that nNOS is important in regulating NO levels in melanocytes, which subsequently interacts with superoxide to generate highly reactive oxidants, causing DNA damage and protein modifications.^9,10^ Our research group and others have demonstrated that nNOS-mediated NO signaling plays a critical role in the pathophysiology of melanoma.^10–12^ Overexpression of nNOS in melanoma biopsy samples^13,14^ was shown to be associated with disease progression by interacting with the interferon-gamma/STAT/PD-L1 ^11,15^ and COX-2/PGE axes,^16^ which not only impact cell proliferation and tumor growth but also regulate the tumor microenvironment and immune response. Targeting nNOS with selective inhibitors exhibited potent antimelanoma activity, presenting a novel and promising therapeutic approach.^4^

As shown in previous studies^11,12,17,18^ the Silverman group has developed a wide range of nNOS inhibitors for melanoma treatment, from which MAC-3-190 and HH044 (Figure 1) were identified as the lead compounds. HH044 was shown to be effective in treating melanoma in a human xenograft mouse model.^11^ In addition, we found that HH044 reduced PD-L1 expression in melanoma cells and that the combination of HH044 with an immune checkpoint inhibitor (ICI) significantly enhanced of antimelanoma activity.^8^ Based on these results, we hypothesized that selective nNOS inhibitors either alone or in combination with checkpoint blockade immunotherapy, can effectively treat melanoma.^8^ HH044, a thiophene-2-carboximidamide-based nNOS inhibitor, showed only moderate selectivity^17^ and displayed cytotoxicity in melanocytes and fibroblast cells.

**Figure 1:**
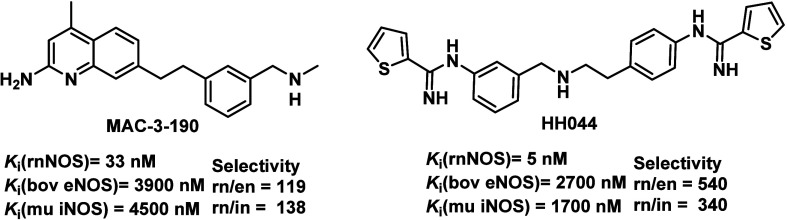
Representative nNOS inhibitors that share similar pharmacophores.

One reason for the observed cytotoxicity may be the poor isoform selectivity of HH044, with the potential to inhibit either eNOS or iNOS, resulting in off-target toxicities. However, achieving highly selective nNOS inhibition is challenging, given the high degree of similarity in the heme active sites of the three NOS isoforms.^19–24^

Most of the reported nNOS inhibitors are competitive inhibitors that mimic the guanidinium group of the substrate, l-Arg, which interacts with the conserved active site Glu.^20–25^ Many of these inhibitors have the “head” of the inhibitor interacting with the active site Glu, with the “tail” end of the inhibitor connected to the head group by a linker of varying lengths that enables the tail to extend out of the active site. Since this interaction between the head group and the active site Glu is conserved in all NOS isoforms, efforts to improve selectivity have focused on modification of the linker and tail end of the inhibitor that offer the possibility of interacting with amino acids outside the active site that are not conserved in all NOS isoforms.^20^

It is also desirable to have a lead compound that roughly equally inhibits rodent nNOS (for preclinical studies) and human nNOS (for clinical studies). We recently observed that the potencies of previously developed nNOS inhibitors did not correlate well between human nNOS (hnNOS) and rat nNOS (rnNOS),^26^ particularly for HH044 (Table 1). In this study, we aim to lay the foundation for the development of clinically drug-like pharmaceutical inhibitors by designing potent and selective nNOS inhibitors and evaluating their antimelanoma activities through *in vitro* studies.

## Results and Discussion

### Overall Strategy

We have identified a potentially important residue difference in a peripheral nNOS inhibitor binding pocket that interacts with the tail end of many nNOS inhibitors.^26^ This residue, Leu337 in rat nNOS or Phe105 in human eNOS, corresponds to His342 at the periphery of the binding pocket in human nNOS (Figure 2). The previously determined crystal structure of rnNOS-HH044 ^17^ showed that, as expected, the thiophene-2-carboximidamide head group interacts with the active site Glu while the tail phenyl ring is nestled in a nonpolar pocket formed by Met336, Leu337, and Trp306 (chain B). That Leu337 is His342 in human nNOS could, in part, account for why HH044 exhibits higher potency toward rat nNOS^17^ as Leu337 in rat nNOS provides a more favorable nonpolar environment for the tail end of the inhibitor than does His342 in human nNOS. We therefore hypothesized that better human nNOS binding would occur upon increasing the polarity of the thiophene group of HH044 (Figure 3). To validate our hypothesis, we started a structure–activity relationship (SAR) study by replacing the thiophene head group in HH044 with a furan head group in **1** (Figure 3) and found somewhat better potency and selectivity in enzymatic assays, supporting our approach.

**Figure 2:**
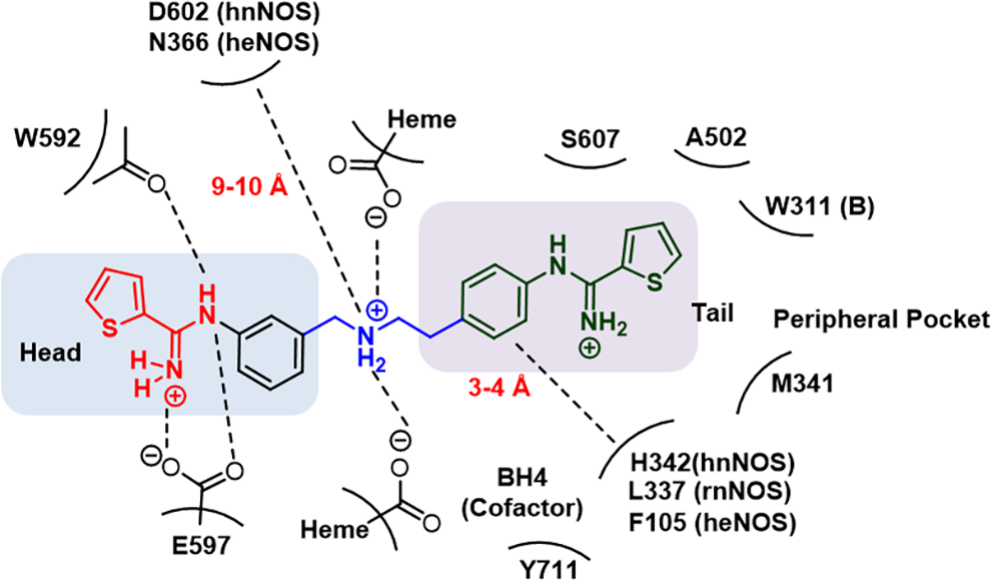
Schematic diagram of the binding mode of HH044 in the active site of NOS.

**Figure 3:**
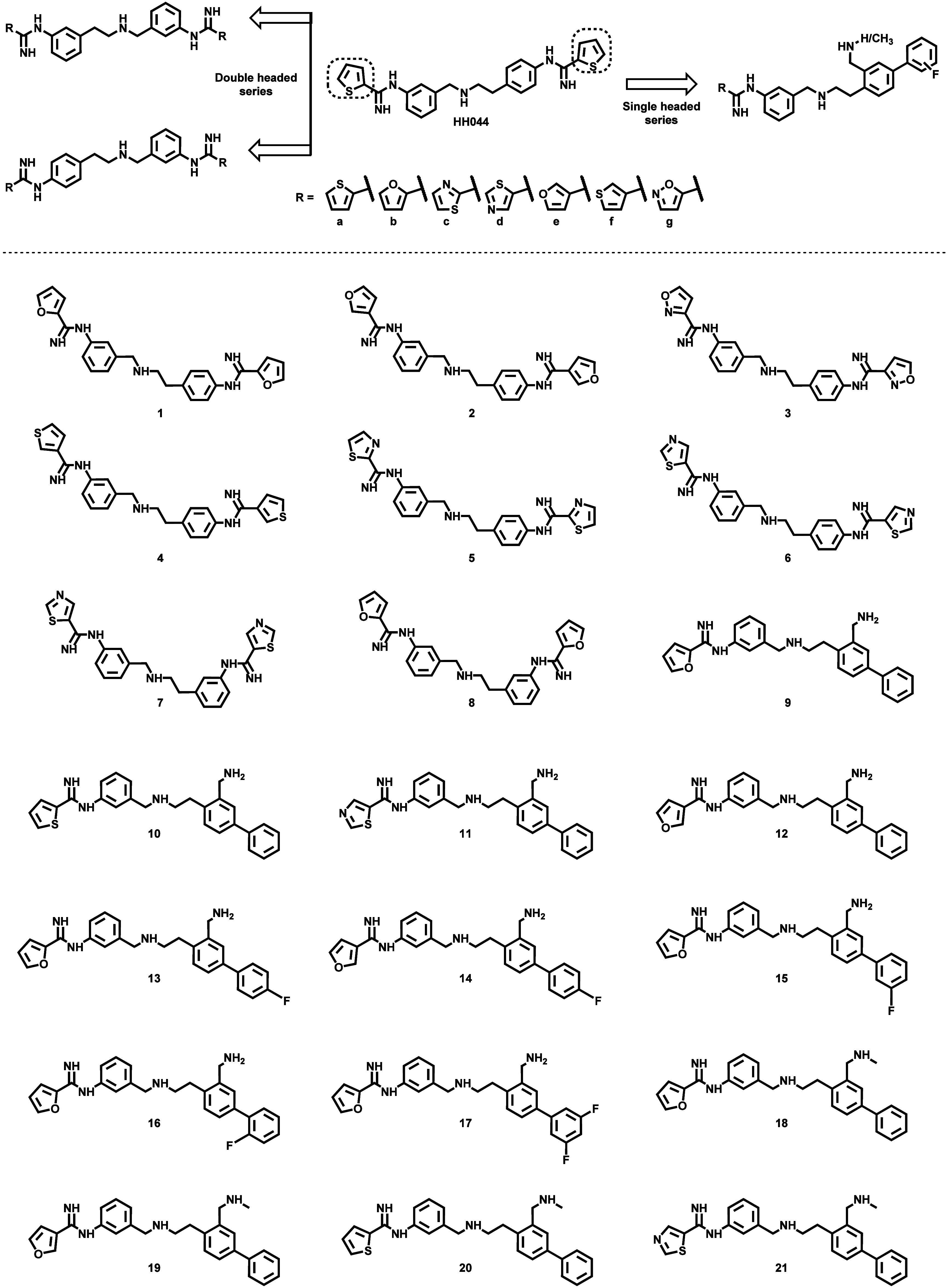
Structural modifications of the lead compound HH044 to improve isoform selectivity and newly designed hnNOS inhibitors in the present study.

Encouraged by these initial experiments, we synthesized a double-headed series by changing the thiophene ring with different 5-membered heterocycles (Figure 3). We further developed a single headed series, in which the second 5-membered heterocycle carboximidamide phenyl moiety was replaced with a biphenyl tail. We also introduced an aminomethyl group on the first phenyl ring to induce hydrogen bonds with the BH_4_ cofactor, and to allow the phenyl tail to induce π stacking with the human nNOS-specific His342 residue. Furthermore, we evaluated melanoma activity and PD-L1 expression with both the double-headed and single headed inhibitors. Finally, crystallographic studies and computational molecular modeling were performed to evaluate our hypotheses and help inform the observed SAR.

### Chemistry

Syntheses of target compounds (**1–21**) are illustrated in Schemes 1–5. To explore different heterocycles as a head group, a series of diverse head groups were synthesized, as shown in Scheme 1. Treating the various aryl nitriles (**21a**–**g**) with ammonium sulfide and triethylamine in pyridine at 50 °C gave thioamides (**22a–** **g**), which were further allowed to react with iodomethane to yield a series of diverse head groups as hydrogen iodide salts^27^ (**23a**–**g**) (Scheme 1).

**Scheme 1:**
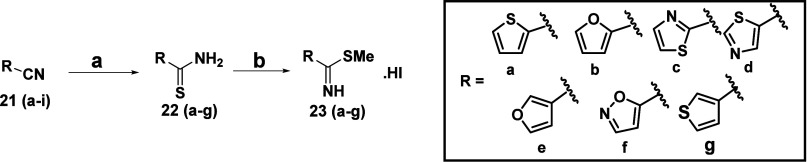
Synthesis of Head Groups **26a–g** ^a^

a Reagents and conditions: (a) (NH_4_)_2_S, NEt_3_, Py, 50 °C, 12 h and (b) CH_3_I, acetone, 12 h.

The synthesis of *meta-*, *para-*substituted double-headed NOS inhibitors **1–6**, is depicted in Scheme 2. Reductive amination of commercially available 2-(4-nitrophenyl)­ethan-1-amine (**24)** with *m*-nitrobenzaldehyde in the presence of cat. AcOH, sodium cyanoborohydride, and in situ Boc protection gave secondary amine intermediate^28^ **25**. The nitro groups of **25** were reduced to the corresponding amines with Pd/C and H_2_ to give intermediate^29^ **26.** Coupling of **26** containing an aromatic amine with synthesized head groups^17^ (**23a**–**g**) and Boc deprotection with 3 M HCl in dioxane gave *meta*-, *para*-substituted double-headed nNOS inhibitors **1–6** as HCl salts (Scheme 2).

**Scheme 2:**
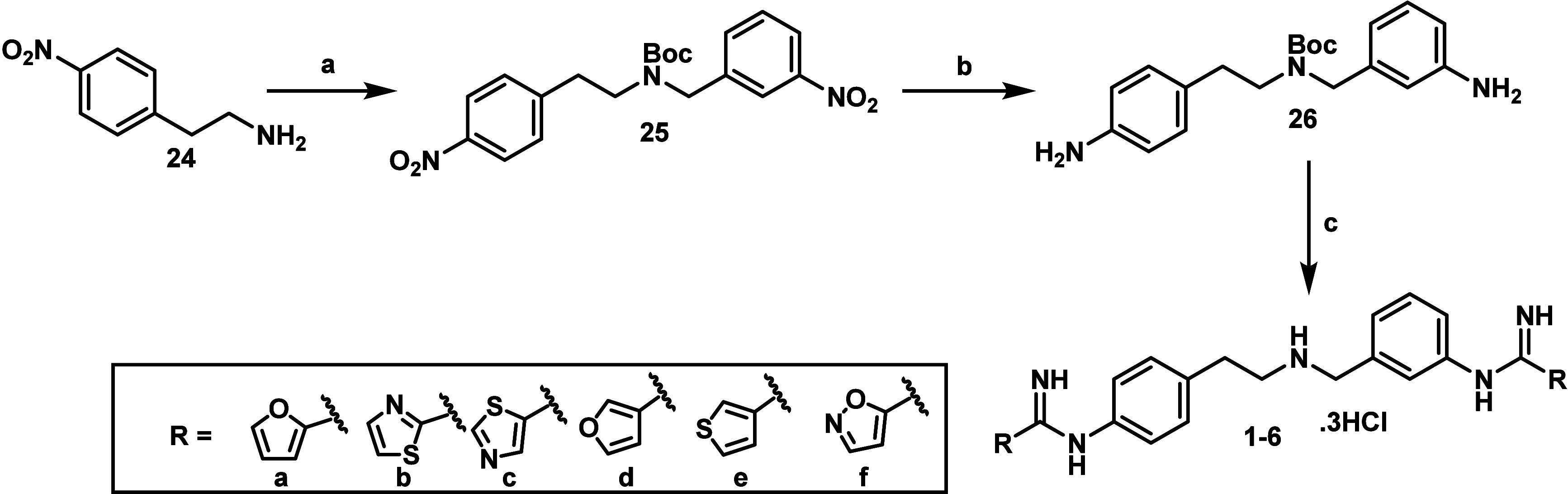
Synthesis of *meta-*, *para*-Substituted Double-Headed nNOS Inhibitors **1**–**6** ^a^

a Reagents and conditions: (a) (i) *m*-nitro benzaldehyde, NaBH_3_CN, cat. glacial acetic acid, THF, 0 °C to rt, 2 h; (ii) (Boc)_2_O, NaHCO_3_ rt, 1 h; (b) Pd/C, H_2_, MeOH, rt, 24 h; and (c) (i) R­(NH)­SMe·HI, EtOH, rt, 24 h; (ii) HCl (3 M in dioxane), rt, 2 h.

Synthesis of *meta-*, *meta-*substituted double-headed NOS inhibitors **7** and **8** (Scheme 3) was carried out similarly as depicted in Scheme 2 with commercially available 2-(3-nitrophenyl)­ethan-1-amine (**27**).

**Scheme 3:**
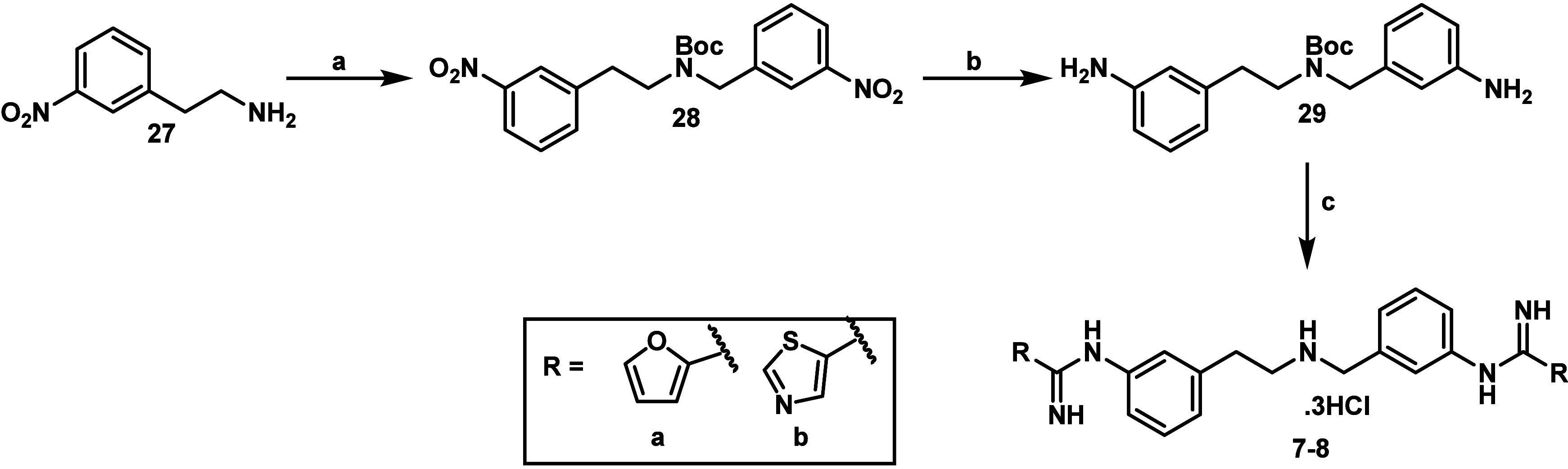
Synthesis of *meta-*, *meta-*Substituted Double-Headed nNOS Inhibitors **7** and **8** ^a^

a Reagents and conditions: (a) (i) *m*-nitrobenzaldehyde, NaBH_3_CN, cat. glacial acetic acid, THF, 0 °C to rt, 2 h; (ii) (Boc)_2_O, NaHCO_3_ rt, 1 h; (b) Pd/C, H_2_, MeOH, rt, 24 h; and (c) (i) R­(NH)­SMe·HI, EtOH, rt, 24 h; (ii) HCl (3 M in dioxane), rt, 2 h.

The syntheses of compounds **9–17** were performed via Scheme 4, starting from commercially available 5-iodo-2-methylbenzonitrile (**30**); Suzuki coupling of **30** with various aryl boronic acids produced intermediates^30^ **31–35**. These biphenyl intermediates (**31–35**) were treated with NiCl_2_·6H_2_O and sodium borohydride to reduce the nitrile group to the corresponding primary amines, which were immediately protected with two Boc groups *in situ*, yielding compounds^31^ **36–40**. Upon further treatment with AIBN and NBS, brominated intermediates **41–45** were obtained.^32^ These intermediates were then treated with KCN in a DCM:water (9:1) mixture, yielding compounds^33^ **46–50**, which were subsequently reduced to the corresponding amines (**51–55**). Reductive amination of intermediates **51–55** with *m*-nitrobenzaldehyde, followed by *in situ* Boc protection, produced intermediates **56–60**. The nitro groups were then reduced with Pd/C and H_2_, yielding the corresponding amines (**61–65**). Finally, these amines (**61–65**) were coupled with head groups and Boc deprotected with 3 M HCl in dioxane to produce the target molecules (**9–17**) as hydrochloride salts.

**Scheme 4:**
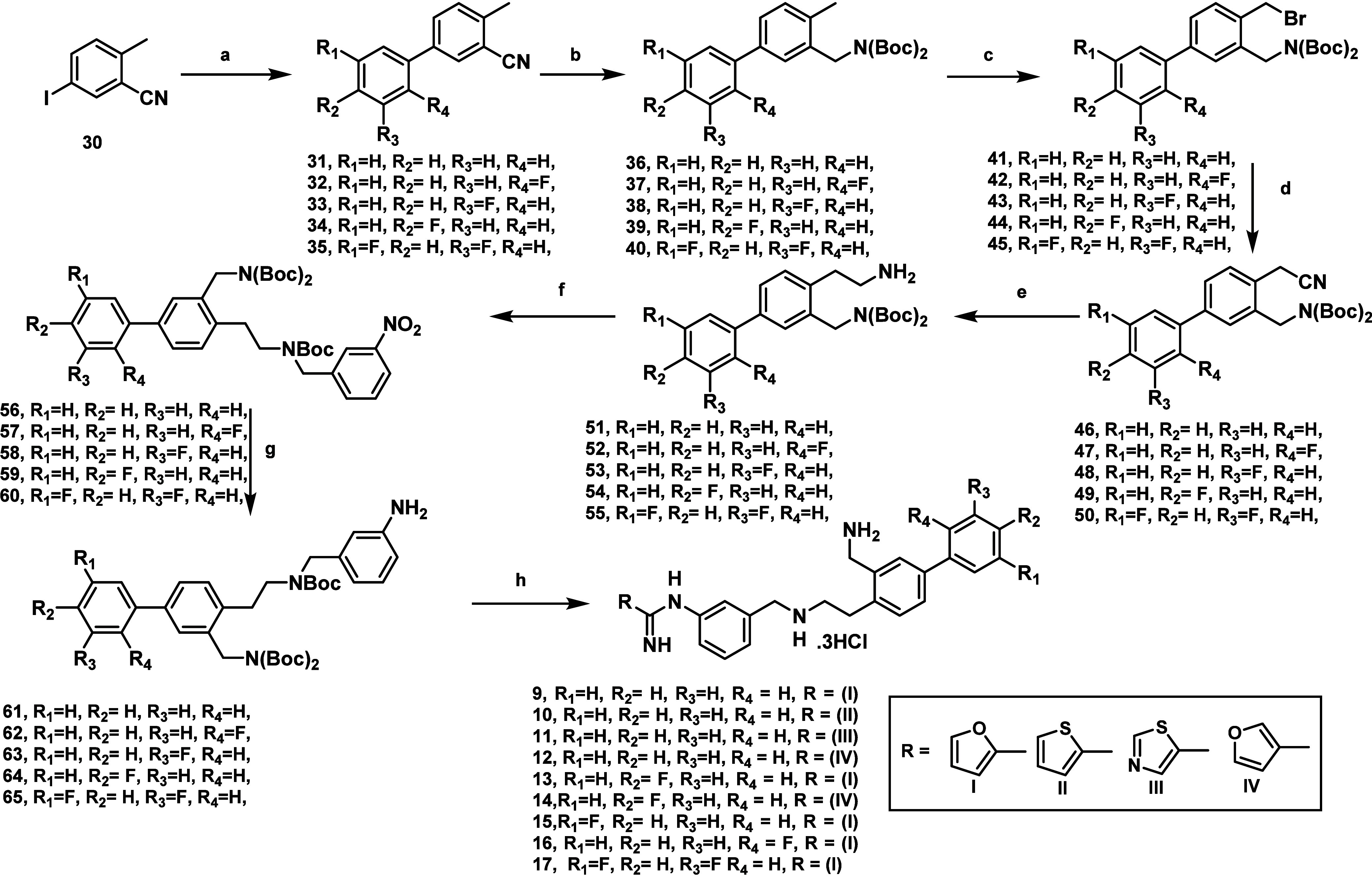
Synthesis of nNOS Inhibitors Containing Biphenyl and Aminomethyl as the Tail (**9–17**)­^a^

a Reagents and conditions: (a) ArB­(OH)_2,_ Pd­(dppf)­Cl_2_, dioxane-H_2_O (9:1), 110 °C, 1h; (b) (i) NiCl_2_·6H_2_O, NaBH_4_, (Boc)_2_O, MeOH, rt, 12 h, (ii) (Boc)_2_O, cat. DMAP, NEt_3,_ ACN, 50 °C, 1 h (c) NBS, AIBN, CCl_4_, reflux, 12 h; (d) KCN, TBAI, DCM-H_2_O (9:1), 24 h; (e) NiCl_2_·6H_2_0, NaBH_4_, MeOH, rt, 12 h, (f) *m*-nitro benzaldehyde, NaBH_3_CN, glacial AcOH, THF, 0 °C to rt, 2 h, then (Boc)_2_. NaHCO_3_ ACN, rt, 1 h, (g) Pd/C, H_2_, MeOH, rt, 24 h; and (h) (i) R­(NH)­SMe·HI, EtOH, rt, 24 h; (ii) HCl (3 M in dioxane), rt, 2 h.

Compounds **18–21** were accessed by a synthetic route (Scheme 5) similar to that in Scheme 4. Compound **66** was treated first with potassium cyanide in a DCM:water (9:1) mixture to make the corresponding nitrile, then partially deprotected to mono-Boc protected **67**. Mono protection allowed later functionalization of the amino group to a secondary amine at a later stage. This intermediate was subsequently reduced to the corresponding primary amine with NiCl_2_·6H_2_O and sodium borohydride to give **68**. Reductive amination of **68** with *m*-nitrobenzaldehyde, followed by *in situ* Boc protection, produced **69**. To obtain the secondary amino group, **69** was treated with sodium hydride and methyl iodide to yield **70**. The nitro group was reduced using catalytic Pd/C and hydrogen gas, yielding the corresponding aromatic amine (**71**), which was coupled with head groups and Boc deprotected with 3 M HCl in dioxane to produce the target molecules (**18–21**) as hydrochloride salts.

**Scheme 5:**
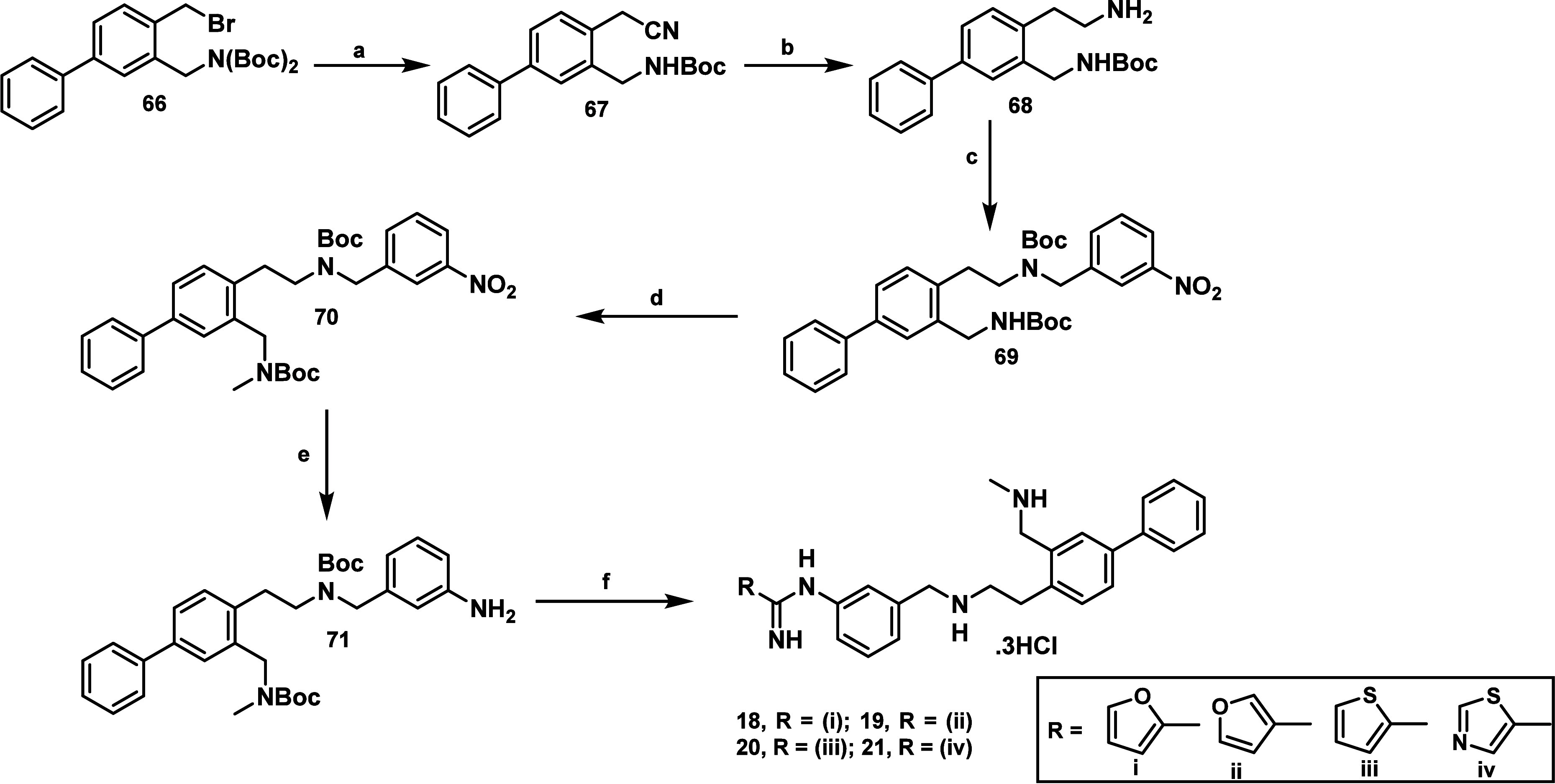
Synthesis of nNOS Inhibitors Containing Biphenyl and Secondary Amine as tail **18**–**21** ^a^

a Reagents and conditions: (a) (i) KCN, TBAI, DCM-H_2_O (9:1), 24 h. (ii) TFA, DCM, rt, 1 h; (b) NiCl_2_.6H_2_O, NaBH_4_, MeOH, rt, 12 h; (c) *m*-nitrobenzaldehyde, NaBH_3_CN, glacial AcOH, THF, 0 °C to rt, 2 h, then (Boc)_2_. NaHCO_3_ ACN, rt, 1 h, (d) NaH, MeI, 0 °C to rt, 2 h; (e) Pd/C, H_2_, MeOH, rt, 24 h; and (f) (i) R­(NH)­SMe·HI, EtOH, rt, 24 h; (ii) HCl (3 M in dioxane), rt, 2 h.

### Biochemical and Antimelanoma Activities of Novel nNOS Inhibitors 1–21

All the biochemical enzymatic assays were performed with HCl salts of the synthesized compounds. The potency and selectivity of NOS inhibition with the newly synthesized molecules were determined using the NO hemoglobin (Hb) capture assay,^24^ and the results are summarized in Table 1. The synthesized molecules were tested with both rat and human nNOS enzymes for preclinical and clinical purposes, respectively, with a ratio of *K*_i_ of hn/rn ∼ 1 being preferable. Cytotoxicity and PD-L1 expression analyses were conducted on the most promising compounds (*K*_i_ hnNOS < 20 nM), which were also screened for their antimelanoma activity. The parallel artificial membrane permeability blood brain barrier (PAMPA-BBB) assay was performed to assess the membrane permeability of lead compounds.

**Table 1:** *K*_i_ Values and Selectivities of Novel Synthesized Molecules

	*K*_i_ (nM)^a^				selectivity^b^		
compound no.	rat nNOS (rn)	human nNOS (hn)	human iNOS (hi)	human eNOS (he)	hn/rn	hn/hi	hn/he
HH044	5	20	1215	6735	4	61	337
**1**·3HCl	6	6	502	3712	1	90	663
**2**·3HCl	3.8	1.6	580	4287	2.3	362	2679
**3**·3HCl	268	319	20,773	8524	1.2	65	29
**4**·3HCl	11	12	711	4489	1.1	59	374
**5**·3HCl	128	149	22,640	42,283	1.1	151	284
**6**·3HCl	20	23	6667	7023	1.1	152	284
**7**·3HCl	30	22	12,680	8008	1.4	576	364
**8**·3HCl	3	9	512	3642	3	60	428
**9**·3HCl	2.3	1.7	426	9612	1.3	250	5654
**10**·3HCl	4.4	3.7	657	3215	1.2	177	945
**11**·3HCl	15	17	12,462	35,044	1.1	733	2061
**12**·3HCl	4	4	577	873	1	144	218
**13**·3HCl	5	8	638	1041	1.6	80	130
**14**·3HCl	4	5	627	1419	1.2	125	289
**15**·3HCl	5	5	610	12,861	1	122	2572
**16**·3HCl	9	8	789	6097	1.1	87	762
**17**·3HCl	ND	47	ND	ND	ND	ND	ND
**18**·3HCl	45	74	5827	15,813	1.6	79	214
**19**·3HCl	14	9	2589	6838	1.5	287	758
**20**·3HCl	97	82	13,742	27,920	1.2	167	340
**21**·3HCl	26	21	2632	11,776	1.2	125	561

a *K*_i_ values were calculated from the IC_50_ values of the corresponding dose–response curves using the Cheng–Prusoff equation. For each compound, 8–11 concentrations were tested, and the IC_50_ value was calculated from an average of at least two duplicates. All standard errors were less than 5%.

b Selectivity values were determined by calculating the ratios of respective *K*_i_ values. The ratio hn/rn is desired to be as close to 1.0 as possible to avoid significant differences between rat and human dosages for clinical studies. For hn/hi and hn/he ratios, higher values are favorable. hn is human nNOS, rn is rat nNOS, hi is human iNOS, he is human eNOS.

Initial screening and SAR studies suggested the importance of a polar furan head group over a thiophene. Molecules in the double-headed series (**1–8**) with a furan ring showed strong potency and good isoform selectivity. For example, **1**, **2**, and **8** had *K*_i_ values for hnNOS < 5 nM, which is superior to the previous lead compound (HH044). However, substitution of an oxazole (**3**) or a 2-thiazole (**5**) gave less potent and less selective compounds. 5-Thiazole substituted **6** and **7** showed better potency than **3** and **5** as both **6** and **7** can make electrostatic interaction from the ring nitrogen atom to the hydroxyl group of the Ser602 side chain, but **5** lacks this interaction because only carbon atoms face Ser602. This could explain why **5** is less potent than **6** or **7** (Supplementary Figures S3 and S4).

Interestingly, the most potent and selective molecule (**2**) (*K*_i_ hnNOS < 2 nM and hn/he = 2679) in the double headed series (**1–8**) did not show better antimelanoma activity than the parent molecule HH044 (Table 2). Furthermore, melanoma screening of the rest of the double-headed compounds revealed that except for HH044, double-headed molecules are not very active against melanoma cancer cells, possibly due to their poor lipophilicity. Hence, we synthesized a single headed series aiming to promote interactions with hnNOS-specific residue His342 and improve potency and selectivity and to address the lipophilicity issue with the aim to improve antimelanoma activity.

**Table 2:** Cytotoxicity (EC_50_) of Molecules 1–21 in Human Melanoma Cells, Melanoblast Cells, and Primary Fibroblast Cells^a^

	human melanoma cells		human melanoblast cells	
compound no.	EC_50_ in A375 (μM)	EC_50_ in SK-MEL-28 (μM)	EC_50_ in Hermes 1 (μM)	EC_50_ in human primary fibroblast cells (μM)
HH044	6.410 ± 0.147	4.557 ± 0.467	7.181 ± 2.681	2.470 ± 0.432
**1**	n/a	n/a	n/a	n/a
**2**	n/a	n/a	n/a	n/a
**3**	n/a	n/a	n/a	n/a
**4**	n/a	n/a	n/a	n/a
**5**	n/a	n/a	n/a	n/a
**6**	n/a	n/a	n/a	n/a
**7**	n/a	n/a	n/a	n/a
**8**	n/a	n/a	n/a	n/a
**9**	2.940 ± 0.716	2.081 ± 0.490	3.518 ± 1.546	3.700 ± 0.281
**10**	1.790 ± 1.122	1.926 ± 0.719	5.219 ± 1.664	3.238 ± 0.573
**11**	4.097 ± 2.027	2.995 ± 0.592	7.793 ± 3.972	6.836 ± 1.143
**12**	4.829 ± 1.363	4.726 ± 0.882	6.445 ± 0.237	4.010 ± 0.756
**13**	2.114 ± 0.501	2.381 ± 0.565	4.167 ± 1.329	2.342 ± 0.808
**14**	n/a	n/a	n/a	n/a
**15**	2.099 ± 0.905	0.825 ± 0.255	3.525 ± 0.398	0.940 ± 0.143
**16**	3.014 ± 0.483	2.161 ± 0.190	3.818 ± 0.144	2.413 ± 0.439
**17**	n/a	n/a	n/a	n/a
**18**	n/a	n/a	n/a	n/a
**19**	5.855 ± 1.956	2.818 ± 0.658	n/a	9.097 ± 0.777
**20**	3.199 ± 0.350	3.073 ± 0.687	6.524 ± 0.597	5.091 ± 1.597
**21**	4.625 ± 1.531	2.513 ± 0.686	4.437 ± 1.351	4.020 ± 0.461

a The EC_50_ for human melanoma cells was not equivalent or superior to that of the lead compound; n/a: no cytotoxicity was detected up to 10 μM.

In the single-headed biphenyl series (**9–21**), molecules synthesized with the biphenyl tail having a primary and secondary amine gave the best results. The most effective molecule (**9**), having a furan ring, showed potency less than 2 nM and hn/he isoform selectivity over 5600. **10** and **11**, with a thiophene and thiazole ring, respectively, also showed good hnNOS potency with hn/he isoform selectivities of 945 and 2061, respectively. Molecules with a biphenyl tail having a secondary amine (**18–21**) were only moderately potent, potentially due to the size of the methyl group, which could hinder the interaction with BH_4_ by weakening the hydrogen bonding between the secondary amino group and BH_4_.

To address the potential metabolic liability of the biphenyl tail of **9**, we introduced a fluorine atom into the terminal phenyl group. We found that *para*-fluoro substituted phenyl compound **13** was active against melanoma cells, although it showed only moderate selectivity compared to **9** in enzymatic assays. Therefore, *meta*- and *ortho*-fluoro derivatives on the phenyl tail (compounds **15** and **16**, respectively) were synthesized; **15** exhibited improved potency and selectivity (hn/he over 2500-fold). Both **15** and **16** also showed lower EC_50_ values than HH044; however, the difluoro derivative **17** was inactive against melanoma cells and showed poor hnNOS potency in enzyme assays.

Cytotoxicity analysis, conducted for screening the antimelanoma activity of the synthesized nNOS inhibitors, established that among all the double-headed synthesized nNOS inhibitors, only HH044 was active against melanoma cells. However, in the biphenyl series, compound **9** (AA-02-16) was identified as one of the most potent molecules, exhibiting enhanced cytotoxicity in two human melanoma cell lines, while showing less toxicity in human primary fibroblast cells in comparison to HH044 (Table 2). Additionally, **9** notably reduced PD-L1 expression levels in melanoma cells, both in the presence and absence of interferon-gamma, a pro-tumorigenic cytokine that stimulates melanoma progression (Figure 4).

**Figure 4:**
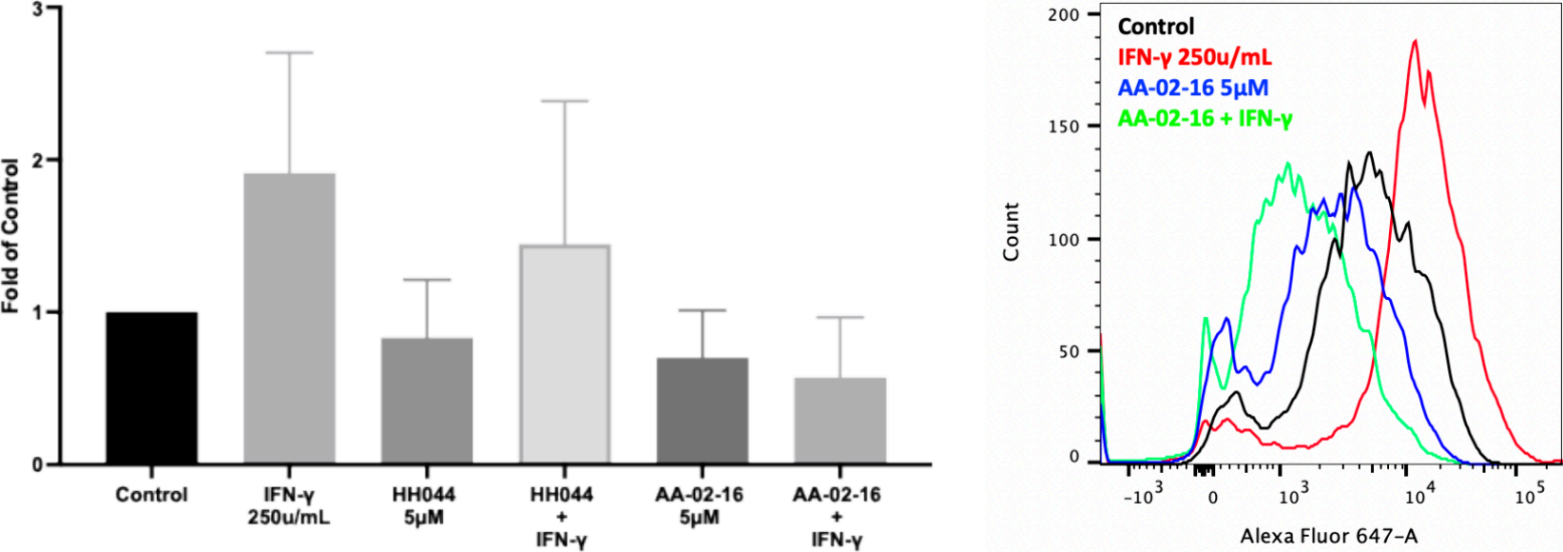
Effects of HH044 and **9** (AA-2–16) on interferon-gamma (IFN-γ)-induced PD-L1 expression levels in human melanoma A375 cells. The baseline PD-L1 levels were reduced to 70% of control after treatment with compound **9**. Co-treatment of **9** effectively reduced the PD-L1 levels from 1.9-fold of control to 57% of control, while the PD-L1 levels with HH044 cotreatment only decreased to 1.4-fold of control.

To assess the potential cellular effects of compound **9** and its direct activity on cellular NO production, we tested its effects on NO levels in HT22 cells using the DAF-FM NO probe as a supportive readout for cell viability. At 0.5 μM, **9** reduced glutamate-induced NO accumulation toward levels seen in vehicle-treated controls. Preliminary dose–response analyses suggested a concentration-dependent trend (see Supplementary Figure S11).

### Crystal Structures of Inhibitor–NOS Enzyme Complexes

One of the goals of this study was to develop inhibitors that are equally potent for both rodent and human nNOS to make a smooth translation from preclinical to clinical studies. The crystal structure of lead compound HH044 bound to rnNOS has been previously described.^17^ Here, we solved the structure of HH044 bound to hnNOS (Figure 5), demonstrating a clear difference between the binding modes of HH044 with rnNOS and hnNOS. As with other inhibitors, HH044 anchors to the active site of hnNOS with H-bonding (salt bridge) interactions between the head carboximidamide moiety and Glu597 (Figure 5A). However, while the amine at the linker sits on top of the heme propionates in both the rnNOS and hnNOS structures, the tail portion of HH044 fits into a pocket capped by a Ser residue (Ser602 in rnNOS and Ser607 in hnNOS), and is positioned quite differently. In hnNOS, the tail thiophene is squeezed in a narrow cleft between Ala502 and Ser607. In contrast, the tail thiophene of HH044 in rnNOS makes van der Waals contacts with Trp306 (chain B, equivalent to Trp311 in hnNOS), shown in Figure 5A in magenta. As a result, the second phenyl ring in the two structures are more than 4.0 Å apart. HH044 has slightly tighter binding to rnNOS than to hnNOS, which can best be explained by the more favorable nonpolar interactions in rnNOS.

**Figure 5:**
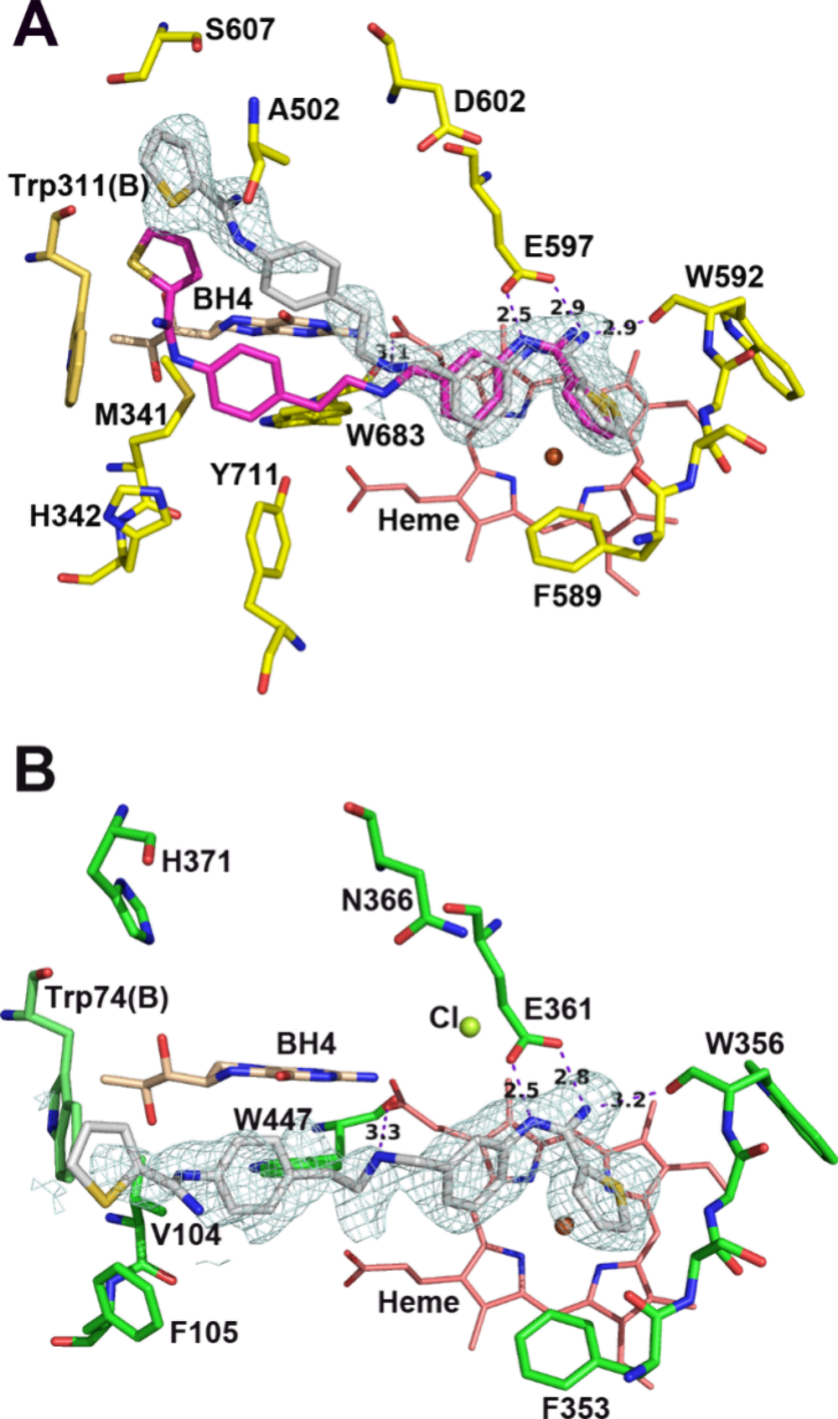
Compound HH044 bound to (A) hnNOS (PDB code: 9MWA) and (B) heNOS (PDB code: 9MWN). The HH044 position found in rnNOS structure (PDB code: 4KCL) is displayed in magenta after the rnNOS-HH044 structure is superimposed onto hnNOS-HH044. In this and all the following structural figures, major H-bonds key to inhibitor binding are depicted with dashed lines. The bond distances are labeled in Å. The omit difference Polder map for the inhibitor is displayed at 2.5–3.0 σ contour level.

The binding position of HH044 in hnNOS represents a unique case that is not observed with other double headed compounds in either hnNOS or rnNOS. In human eNOS, the tail thiophene of HH044 adopts a totally different position next to Phe105 and Trp74 (chain B, Figure 5B), which is quite far from the tail positions seen in both hnNOS and rnNOS. This is because at the end of the pocket where the tail thiophene fits in hnNOS, there is a small Ser residue (Ser607). The equivalent residue in heNOS is the bulkier His371 that prevents the tail thiophene from adopting the orientation observed in hnNOS. The position of the tail portion of HH044 in heNOS is also stabilized by the hydrophobic contacts between the second phenyl ring of HH044 and Phe105. As a result, HH044 shows only a modest hn/he selectivity (341).

### Double-Headed Inhibitors: Optimizing Head Groups

The first modification of HH044 was to replace the thiophene with other 5-membered heterocycles. When a furan replaces thiophene in **1**, the binding mode in rnNOS closely resembles that observed for HH044 and displays similar nonpolar contacts (Figure 6A,B).

**Figure 6:**
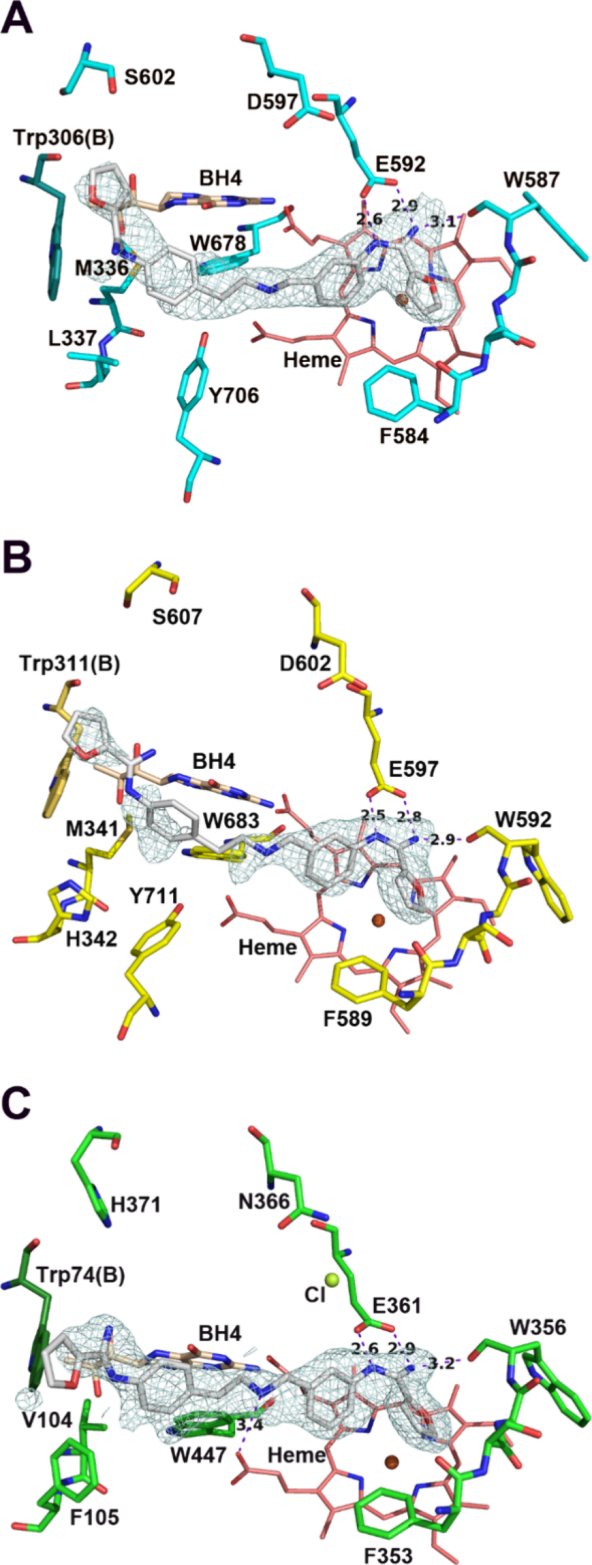
Compound **1** bound to (A) rnNOS (PDB code: 9MWD), (B) hnNOS (PDB code: 9MWB), and (C) heNOS (PDB code: 9MWO).

The similar binding mode of **1** in both rnNOS and hnNOS translates into identical potency, a desirable feature, and the hn/he selectivity is improved by 2-fold (663) compared to HH044. Figure 6C shows the binding of **1** to heNOS, which is very similar to HH044. This relatively modest 2- to 3-fold gain in potency toward both hnNOS and heNOS likely results from the difference in p*K*_a_ values between furan and thiophene. A lower p*K*_a_ for furan compared with thiophene makes it easier to bury into a hydrophobic environment near Trp311 in hnNOS or Trp74 in heNOS.

Changing from a 2-substituted furan in **1** to a 3-substituted furan in **2** also resulted in an improvement in both potency and selectivity (Table 1). A discussion of **2** is in the Supporting Information.

Another double-headed inhibitor that bears a furan ring is **8**, which has its second furan carboximidamide moiety as a *meta*-substituent on the second phenyl ring relative to the linker position, in contrast to **1** and **2**, which have a *para*-substituent. This structural change does not appreciably affect the potency and selectivity of **8** compared to **1**. The structure of rnNOS-**8** shows that the *meta*-substituent in **8** brings the tail furan ring deeper into the pocket so that the furan ring is closer to the end-capped Ser602 residue (Figure 7A). In addition, the tail phenyl ring can form slightly favorable interactions with Met336 of rnNOS as compared to **1**. The binding mode of **8** in heNOS (Figure 7B) is also similar to that of **1** in heNOS (Figure 6C). Overall, the potencies of **8** and **1** (Table 1) are similar across rnNOS and heNOS. That means the substituent pattern on the second phenyl ring has little impact on the inhibitory activity.

**Figure 7:**
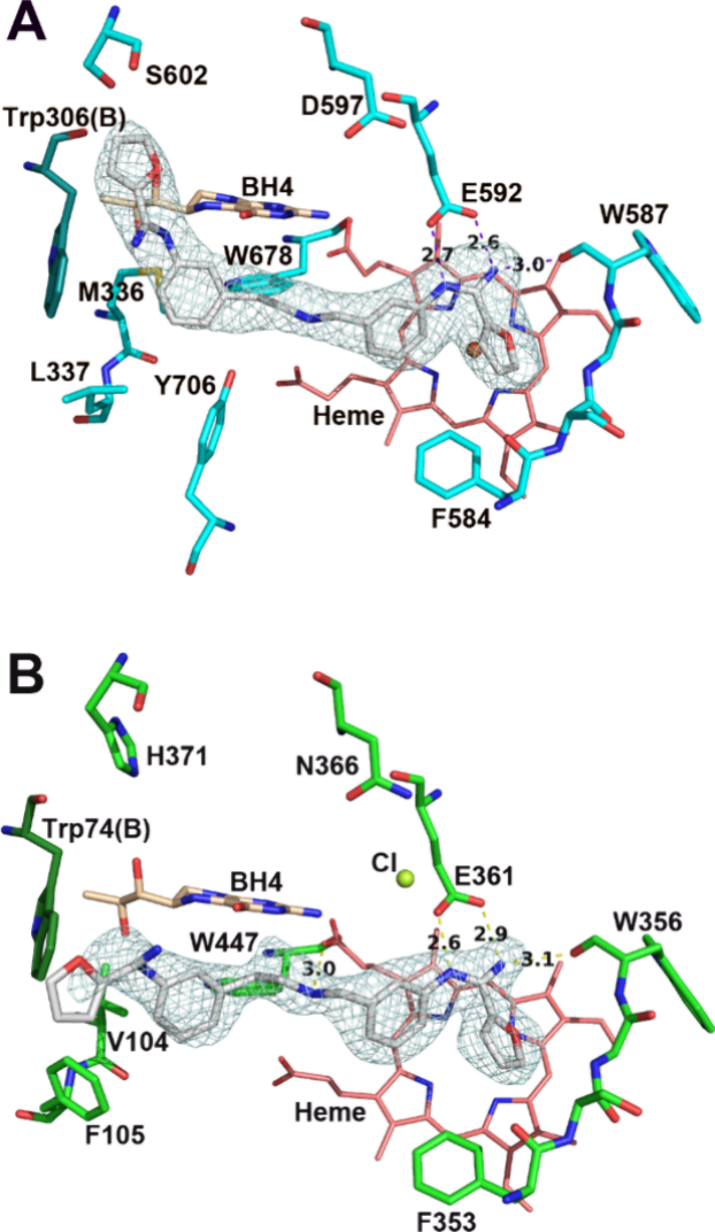
Compound **8** bound to (A) rnNOS (PDB code: 9MWJ) and (B) heNOS (PDB code: 9MWT).

Replacing the thiophene of HH044 with other 5-membered heterocycles was not always successful. Compound **3** contains an oxazole while **5**, **6**, and **7** contain a thiazole. The potencies of these compounds for all NOS isoforms are decreased as compared to **1**. The SAR for these weaker inhibitors is discussed in the Supporting Information.

### Single-Headed Inhibitors: Optimizing the Head Group

#### Lead Compound 9

Cytotoxicity results of double headed compounds against human melanoma cell lines prompted us to extend our inhibitor search to single headed NOS inhibitor analogs. The second 5-membered ring carboximidamide phenyl moiety was replaced by a biphenyl unit to improve lipophilicity and to create π stacking with the hnNOS-specific His342 residue. Furan-containing **9** showed outstanding potency and selectivity. The structure of rnNOS-**9** (Figure 8A) reveals an additional H-bond from the aminomethyl group off the first phenyl ring to the oxygen atom of BH_4_. The first phenyl ring is in van der Waals distances from Met336. The electron density of the second phenyl is weak so the exact orientation cannot be determined, although this ring must contact Trp306 (chain B). The binding of **9** to hnNOS is similar (Figure 8B). The H-bonding interactions and van der Waals contacts between the hydrophobic phenyl tail and protein make **9** a very potent inhibitor for both rnNOS and hnNOS (Table 1). **9** also exhibits strong activity against melanoma (Table 2), making **9** the most therapeutically promising compound in this series.

**Figure 8:**
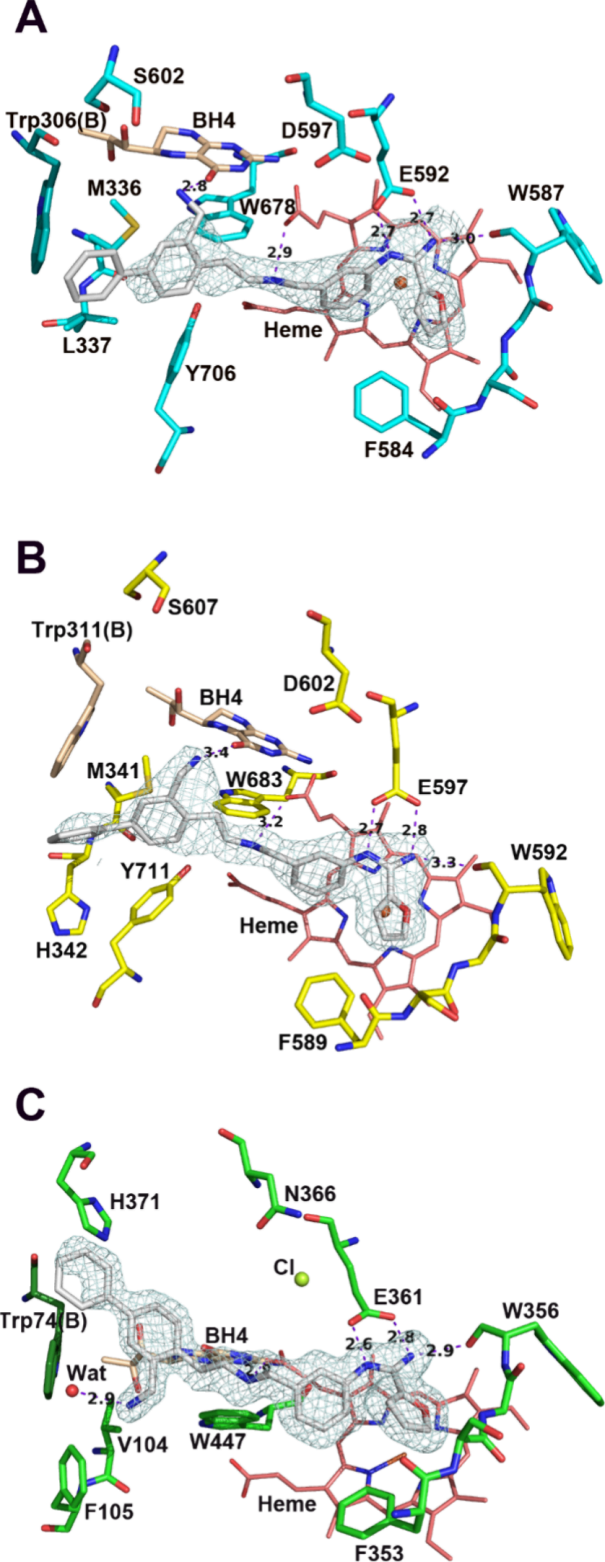
Compound **9** bound to (A) rnNOS (PDB code: 9MWK), (B) hnNOS (PDB code: 9MWC), and (C) heNOS (PDB code: 9MWU).

To our surprise, in heNOS, the diphenyl moiety of **9** extends into the pocket end-capped by His371 (Figure 8C). This residue is the reason that the tail of the double headed inhibitors cannot fit in this pocket in heNOS; however, the shorter diphenyl moiety has the right length to make direct van der Waals contacts with His371. The aminomethyl group in **9** H-bonds with a nearby water molecule in heNOS instead of interacting with BH_4_ as in nNOS. In heNOS, losing the hydrogen bond with BH_4_ and having a not so favorable fit of the biphenyl group in the His371 capped pocket result in a poorer potency of **9** against heNOS. Because of its strong potency toward hnNOS, **9** shows excellent hn/he selectivity.

Compounds **10** and **11** are analogs of **9** with the 5-membered ring changed from furan to thiophene and thiazole, respectively. The relatively weak electron density of the biphenyl moiety of **10** and **11** in rnNOS (Figure 9A,B) suggests that the aminomethyl does not make the same H-bonding interactions as that seen for **9**. The binding of **10** and **11** to heNOS (Figure 9C,D) closely resembles that of **9** with the tail phenyl ring in contact with His371 and the aminomethyl group pointing away from BH_4_ and making a H-bond with a nearby water molecule. The reason **11** is much less potent with heNOS than **9** and **10** is not obvious. The thiazole containing double headed inhibitors discussed earlier also showed poor potency. The chemical nature of the thiazole ring, closely interacting with heme and the protein backbone, might be the cause since there are no apparent structural differences that can be used to interpret the poorer potency for these thiazole-containing inhibitors.

**Figure 9:**
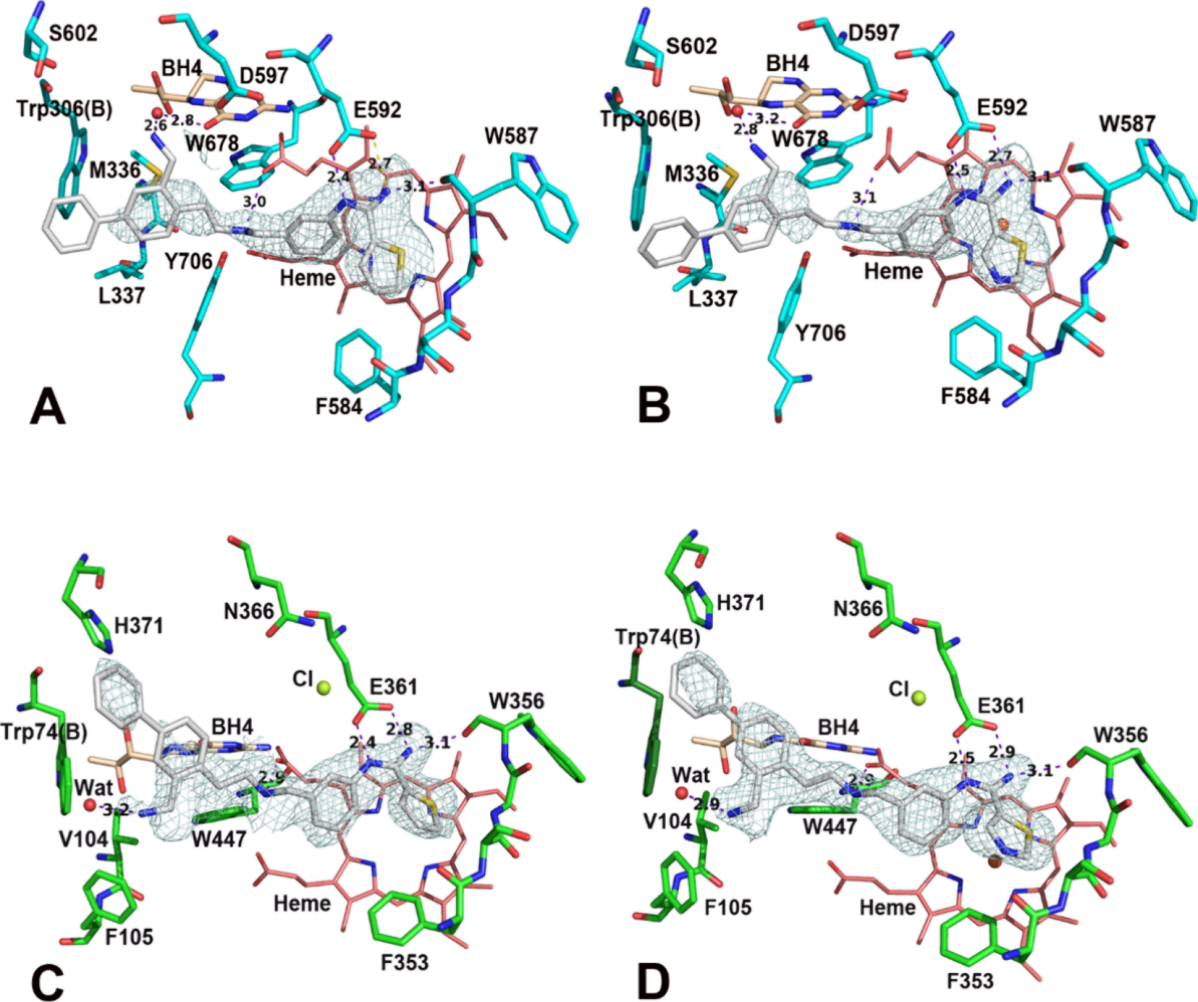
(A) Compound **10** bound to rnNOS (PDB code: 9MWL), (B) compound **11** bound to rnNOS (PDB code: 9MWM), (C) compound **10** bound to heNOS (PDB code: 9MWV), and (D) compound **11** bound to heNOS (PDB code: 9MWW).

#### Analysis of Fluorine Substitution on the Phenyl Tail of 13, 15, and 16 through Molecular Modeling Studies

Because *para*-, *meta*-, and *ortho*-substitution at the terminal phenyl of **9** (**13**, **15**, and **16**, respectively) allows for differentiation of the activity with heNOS, but not with hnNOS, a possible interacting partner of the fluorine substituent was searched for by comparing 100 ns MD simulation trajectories from the crystal structures of **9** in hnNOS and heNOS. In the MD trajectories simulated from the heNOS-**9** crystal structure (9MWU), the minimum distance between the terminal phenyl ring centroid and the ε-nitrogen atom of Lys72 (Chain B) (N_Lys72(B)_) was 3.7 Å, whereas that in hnNOS was 5.5 Å (Figure S5). The direction of the aminomethyl group from the first phenyl of the biphenyl moiety and whether there is a H-bond to BH_4_ are the keys for this difference in distance between the two NOS isoforms. Also, note that in the structure of heNOS-**9**, the biphenyl moiety is trapped in the His371 capped pocket (Figure 8C). However, the flexibility of the linker between the phenyl next to the carboximidamide and the biphenyl allows the latter to swing out of the pocket (see Figure 10). This swing out position of the biphenyl is critical for the fluorine-substituted biphenyl compounds because they no longer fit into the His371-capped pocket. For the model generation, the appropriate frame with a close phenyl–N_Lys72(B)_ distance (4.5 Å) was selected among the frames maintaining the key tail interactions with the BH_4_ and heme propionate. The model was generated by an atomic replacement for each fluorine substitution and subsequent minimization of the selected template frame. It was then subjected to metadynamics simulation pursuing various target distances.

**Figure 10:**
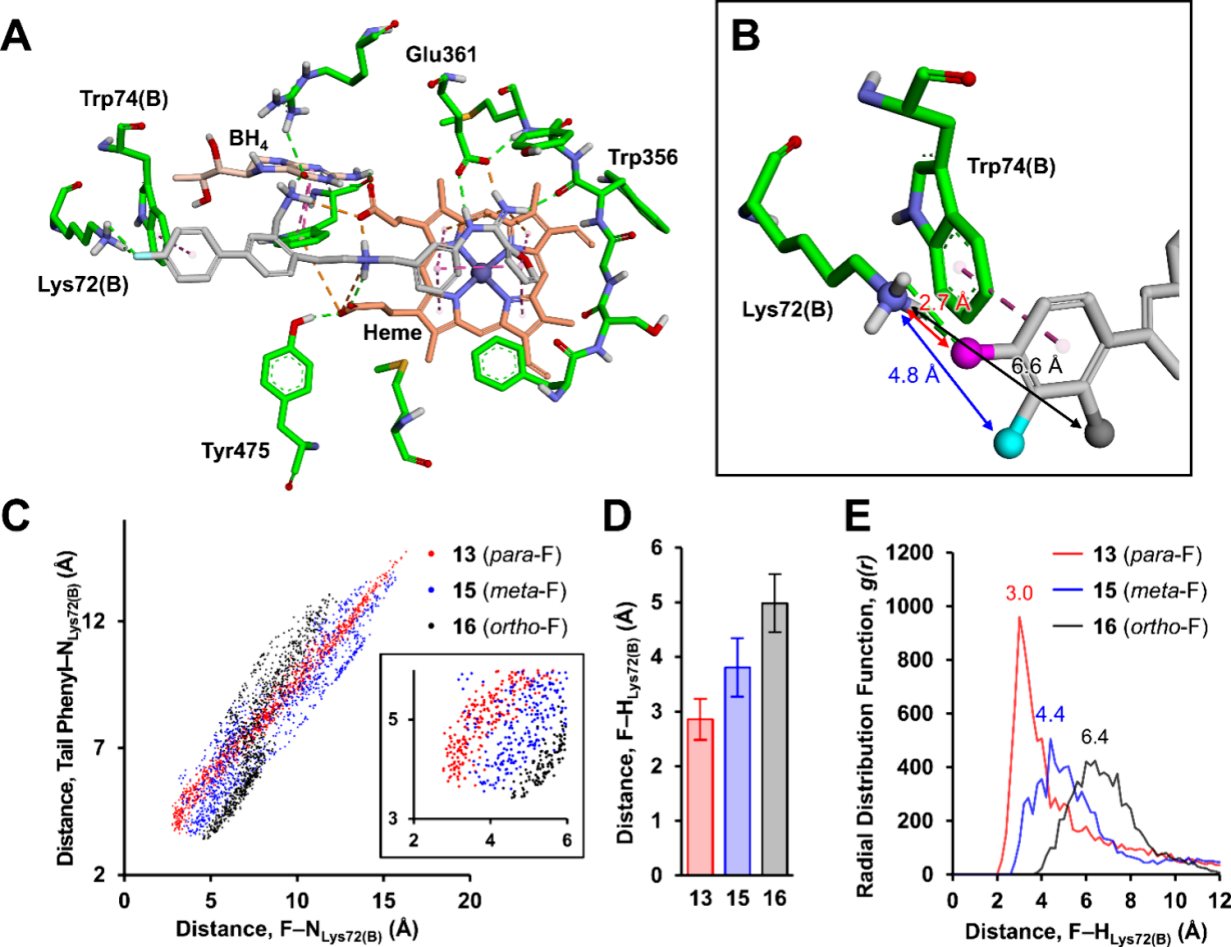
Predicted effect of fluorine substitution of **9** using metadynamics simulation in heNOS using the heNOS-**9** crystal structure (PDB ID: 9MWU). (A) Representative frame of the binding mode of the *para*-substituted derivative **13** interacting with Lys72­(B) and (B) comparison of the distances from the other positions. Green, orange, and gray sticks indicate the heNOS binding site residue atoms, cofactors, and **13**, respectively. (C) Scatter plot displaying the distance from the tail phenyl ring centroid and each substituted fluorine atom to the Lys72­(B) ε-nitrogen atom (N_Lys72(B)_). (D) Distance between each fluorine atom and the nearest proton of the Lys72­(B) amine (mean ± SD) from the frames with proximal tail phenyl and N_Lys72(B)_ (< 5.0 Å). (E) Radial distribution function, *g*(*r*), of protons of the Lys72­(B) amine around each substituted fluorine atom in the trajectories.

As a result of enhanced sampling by metadynamics, **13**, a *para*-fluorine-substituted derivative of **9**, was observed to allow the interaction with Lys72­(B) while maintaining the anchor between its tail amino group and the BH_4_ carbonyl and heme propionate (Figure 10A). In this snapshot of the metadynamics trajectory, the tail was additionally positioned by an edge-to-face π-interaction of the terminal phenyl group and the Trp74­(B) side chain. Since the distance from the head-binding active site of heNOS to Lys72­(B) is too far (∼20 Å) to reach N_Lys72(B)_, the linker portion of the molecule must be as stretched as possible, and the whole structure must be linear. Even with the maximum stretch and linear conformation of the ligand, the distances between N_Lys72(B)_ and the *ortho*- and *meta*-substituents were too far, so it was observed as impossible to form an interaction (Figure 10B). In addition, in the collective analysis of all the trajectories (Figure 10C), when the tail phenyl ring is proximal to the N_Lys72(B)_ (< 5 Å), each distance between the fluorine substituent and the protons of N_Lys72(B)_ was 3.3 ± 0.4, 4.5 ± 0.6, and 5.7 ± 0.5 Å, for *para*-, *meta*-, and *ortho*-substitution, respectively (Figure 10D). Notably, based on the radial distribution function (*g*(*r*) in Figure 10E), the peak distances of **13** (3.0 Å), **15** (4.4 Å), and **16** (6.4 Å) indicated that *para* substitution is the only option allowing H-bond formation with Lys72­(B), considering the generally accepted range (< 2.8 Å). Therefore, this could be one plausible explanation for the improved heNOS activity by *para*-fluorine substitution (**13**), resulting in reduced isoform selectivity.

#### Rationalization of the Flexibility of the Tail of Both Double-Headed and Single-Headed Inhibitors through Molecular Modeling

Based on the information from the crystal structures of various NOS isoforms, the dynamic characteristics in the binding of double- and single-headed inhibitors to the NOS isoforms were investigated. Since the crystal structure represents an averaged coordinate of the snapshots of every molecule in the crystal during the collection time,^34^ crystallographic data not only include the atomic coordinates but also the atomic displacements (B-factor),^35^ which reflects the density probability distribution of atoms from their average position caused by thermal motion. According to the crystal structures of double- and single-headed inhibitors bound in various NOS isoforms from our previous report^17^ and this study, the head portions of the inhibitors tightly bind to the active site of the enzyme exhibiting well-defined constant conformations in every complex (Figure 11A). However, the tail portions at the entrance of the enzyme active site are exposed to the largely open vestibule (Figure 11A). Hence, the tail group atoms showed diffused, less-defined electron density and relatively high atomic displacement compared to the corresponding head group atoms (Figure 11B). This reflects their high degree of flexibility and raises the possibility that the tail group does not adopt a single static conformation but rather dynamic heterogeneous conformations in the NOS binding pocket, especially in the region where NOS isoform amino acid variants reside.

**Figure 11:**
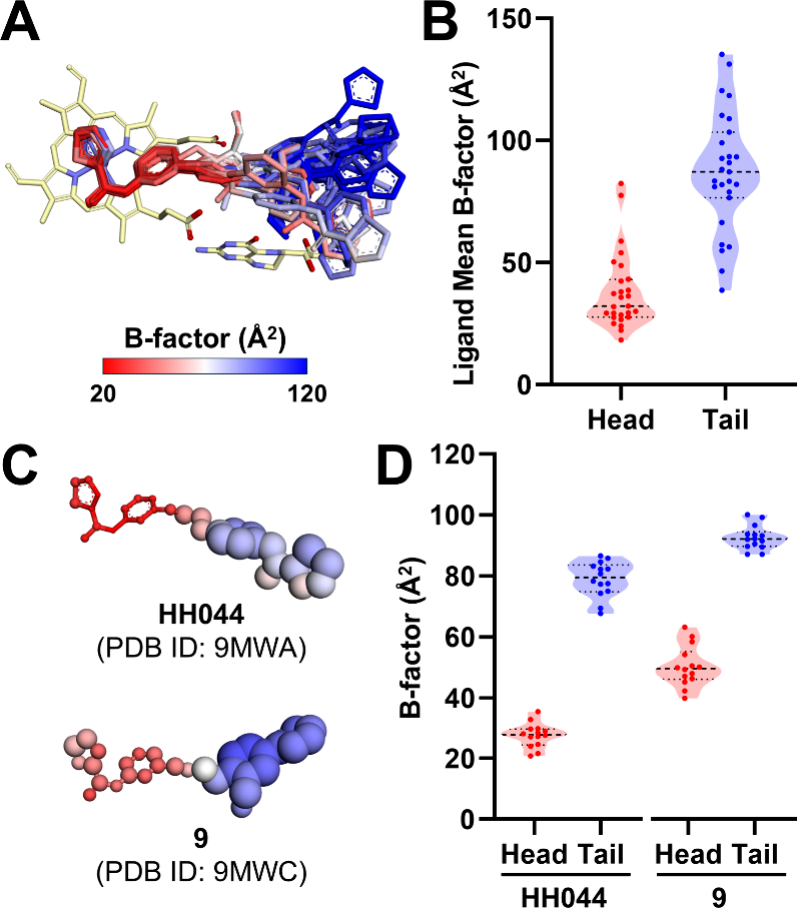
Comparison of the flexibility of the head and tail portions of double- and single-headed inhibitors by their atomic displacement (B-factor) in various NOS isoform crystal structures. (A) Superimposed ligand atoms of the rnNOS, hnNOS, and heNOS crystals complexed with reported double-headed inhibitors^17^ and with new inhibitors (this study). The ligand atoms are colored by their B-factor. The ivory sticks indicate cofactors in the hnNOS-HH044 crystal structure (PDB code: 9MWA). (B) Distribution of each averaged B-factor of the ligand’s head and tail group atoms from the NOS isoform crystal structures. (C) Representations of B-factors on the HH044 and **9** ligand atoms in the hnNOS crystal structures, colored and scaled by B-factor. (D) Distribution of B-factor of each head and tail group atoms of HH044 and **9** in the hnNOS crystal structures. The dotted lines in violin plots indicate quartile and median values.

To investigate the putative heterogeneous binding modes of HH044 induced by the dynamic conformations of its tail group, a 100 ns molecular dynamics (MD) simulation was conducted on the hnNOS-HH044 complex. Since the MD simulation allows serial slight structural changes from the initial structure, a large number of the complex ensembles can be massively generated for simulating the dynamic binding mode.^35^ The result of the MD simulation started from the hnNOS-HH044 crystal structure (PDB ID: 9MWA) showed that the mean of the root-mean-square fluctuation (RMSF) of head group atoms was 0.94 Å (Figure 12A), and the root-mean-square deviation (RMSD) of the head group was maintained at less than 0.5 Å in most frames (Figure 12B). The mean RMSF of the tail group atoms, however, was substantially higher (1.67 Å) than that of the head group atoms, consistent with the observations from the atomic displacement differences in the initial crystal structure (Figure 11C,D). Based on the RMSD, the tail group exhibited drastic movement during the first 35 ns (Figure 12B), suggesting a flexible swinging motion. This result from a restraint-free, unbiased MD simulation reinforces the possible dynamic motion of the tail part and the accompanying heterogeneous binding mode of HH044.

**Figure 12:**
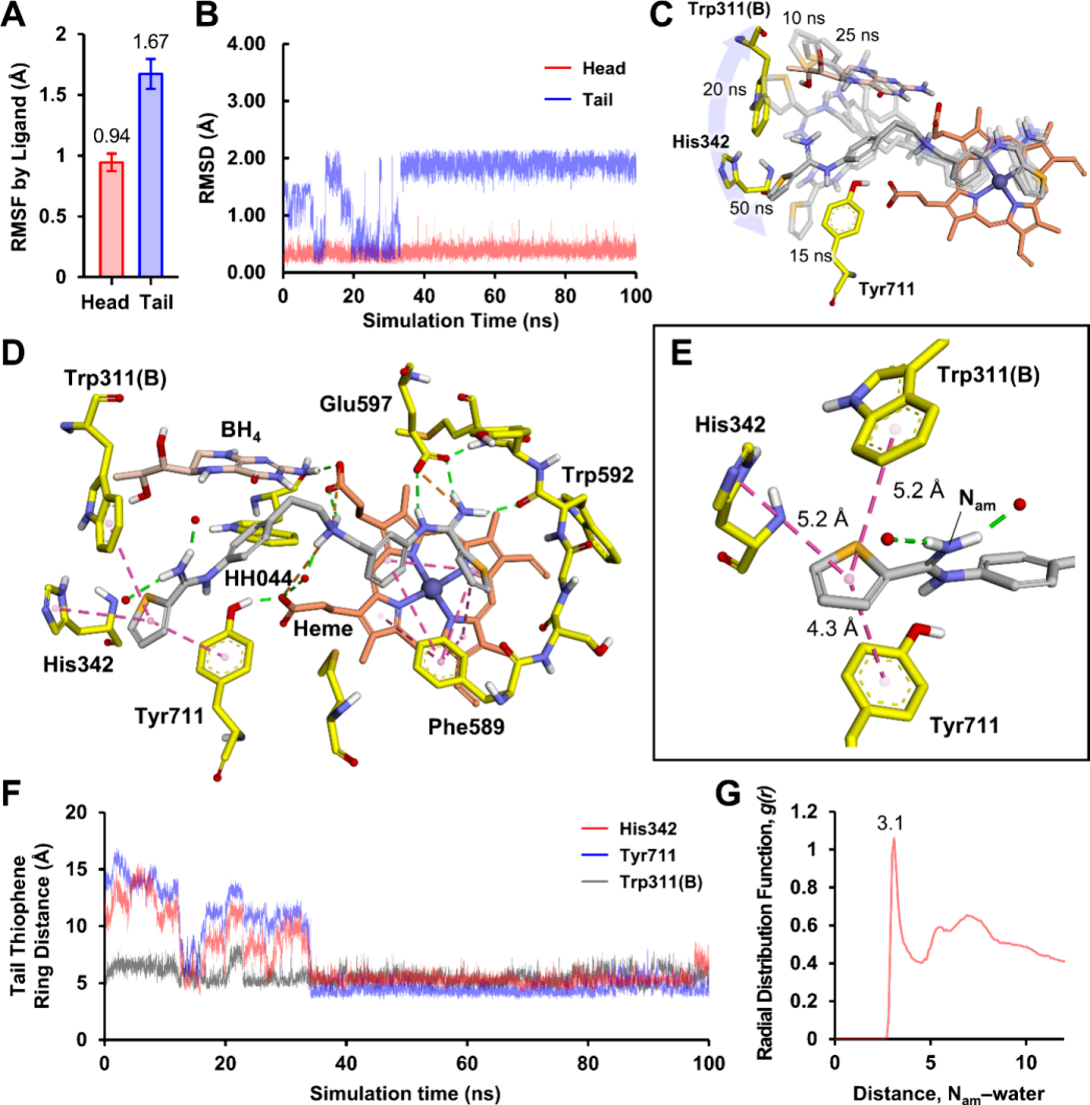
Molecular dynamics (MD) simulation result of HH044 in hnNOS using the hnNOS-HH044 crystal structure (PDB ID: 9MWA). (A) Root-mean-square fluctuation (RMSF) of the ligand head and tail part calculated by average ligand structure during the MD simulation (mean ± SEM). (B) Root-mean-square deviation (RMSD) of the ligand head and tail group atoms. (C) Superimposition of the representative snapshots with various tail conformations. (D) Representative binding mode of HH044 with His342 interaction and (E) detailed interactions by the terminal tail thiophene in the same structure. In panels (C), (D), and (E), yellow, orange, and gray sticks indicate the hnNOS binding site residue atoms, cofactors, and HH044, respectively. Water molecules are shown in red spheres. Green, yellow, and pink-dotted lines indicate hydrogen bond, charge interaction, and pi-interaction, respectively. (F) Ring centroid distance from the terminal tail thiophene to each indicated side chain. (G) Radial distribution function, *g*(*r*), of water molecules around the charged amidine nitrogen atom (N_am_) in the MD trajectories.

During the simulation, the tail group of HH044 exposed to the large vestibule periphery from the enzyme active site exhibited various interactions by a swinging motion (Figure 12C). The interactions include participating in the water network mediated by the tail amidine group according to the radial distribution functions between the charged amidine nitrogen atom (N_am_) and water molecules (Figure 12E,G), and π-stacking with His342, Tyr711, and/or Trp311­(B) by the thiophene (Figure 12D). Notably, the structural equivalents of His342 are Phe105 and Thr121 in heNOS and hiNOS, respectively (Figure S6); thus, the interactions of HH044 with the different amino acid variants possibly provide a basis for the selectivity of HH044 among NOS isoforms. During ∼70% of the simulation time, the π-stacking with His342 was stable with ∼5 Å distance, while at the same time the distance for two additional π-interactions was maintained (Figure 12E,F). The distance between the tail thiophene and Phe105 in heNOS was more than 10 Å (Figure S7) in most MD trajectories simulated from the heNOS-HH044 crystal structure (PDB ID: 9MWN), further reinforcing His342’s possible contribution toward the hnNOS selectivity of HH044.

Using the crystal structure of hnNOS (PDB ID: 9MWC), the MD simulation was also conducted for our most promising compound (**9**), which has a biphenyl tail fragment. During the simulation, the side chain of His342, which was stacked between the Tyr711 side chain and Ser344 main chain in the input crystal structure (His-out conformation in Figure S8), changed its conformation to the His-in conformation at 3 ns and kept this conformation for the rest of the simulation (Figure S8). The transition to the His-in conformation allowed for proximity of the imidazole ring of His342 and the phenyl ring of **9** (∼6 Å, Figures[Fig fig12]E,F and 13C,E), suggesting the possibility of a π-interaction. In addition, the aminomethyl group of the first phenyl group was tightly held by the strong interactions with the carbonyl group of the BH_4_ cofactor molecule and the heme propionate A during the simulation (Figure 13B,D). The strong interactions and extra stabilization by solvation (solvent shell radius = 3.0 Å, Figure 13F) then led the tail group atoms to a relatively stable conformation as compared to HH044 according to their fluctuations (Figures 12A and 13G) and RMSDs (Figures 12B and 13H).

**Figure 13:**
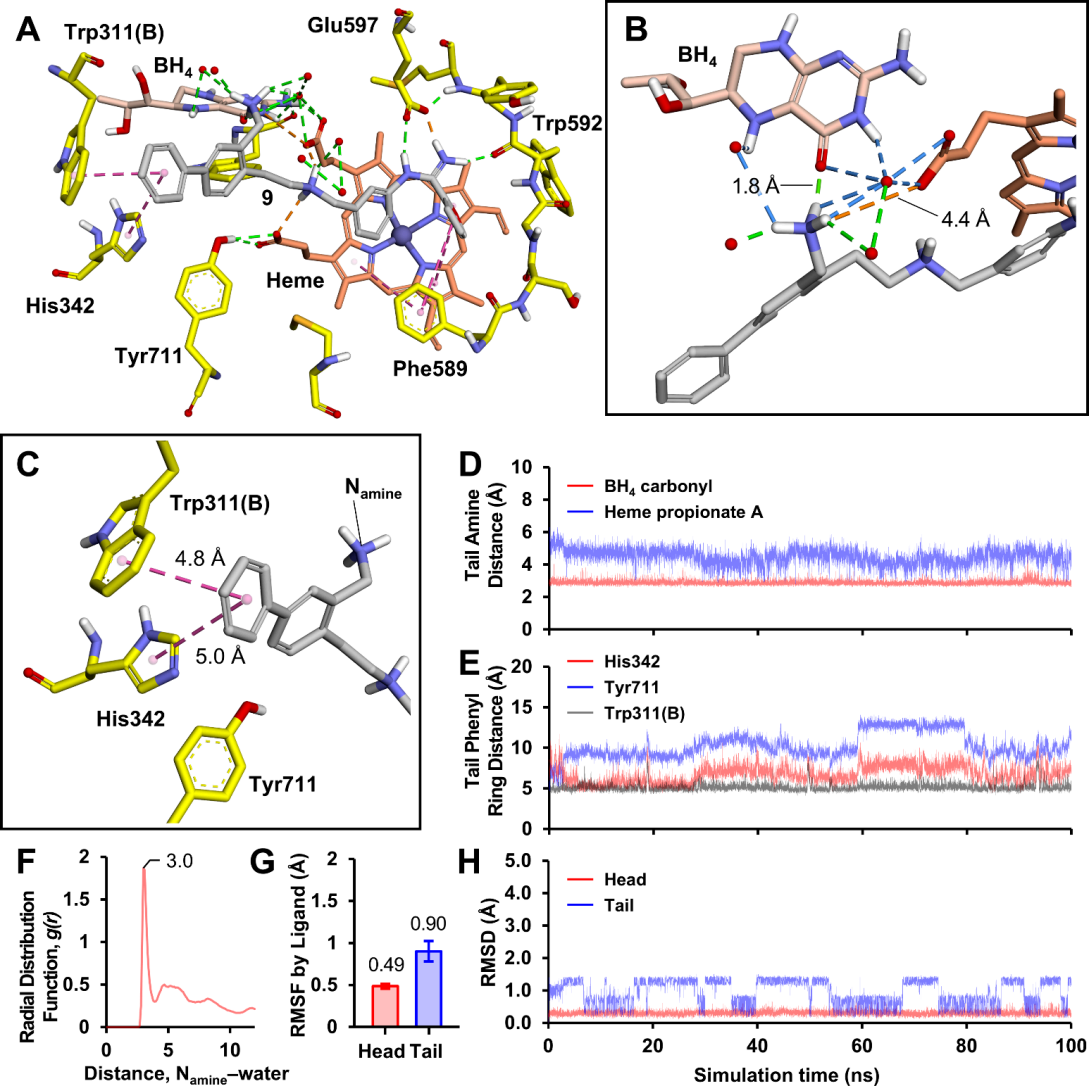
Molecular dynamics (MD) simulation result of **9** in hnNOS using the hnNOS-9 crystal structure (PDB ID: 9MWC). (A) Representative binding mode of **9** showing the His342 interaction. (B) Detailed interactions by the aminomethyl group of the first phenyl group. (C) Terminal tail phenyl group in the representative binding mode. In panels A, B, and C, yellow, orange, and gray sticks indicate the hnNOS binding site residue atoms, cofactors, and **9**, respectively. Water molecules are shown as red spheres. Green, yellow, pink, and blue-dotted lines indicate hydrogen bond, charge interaction, π-interaction, and water-mediated interactions, respectively. (D) Distance between the aminomethyl group and each indicated interacting group. (E) Ring centroid distance from the terminal tail phenyl to each indicated side chain. (F) Radial distribution function, *g*(*r*), of water molecules around the tail amine nitrogen atom (N_am_) in the MD trajectories. (G) Root-mean-square fluctuation (RMSF) of the ligand head and tail groups calculated by average ligand structure during the MD simulation (mean ± SEM). (H) Root-mean-square deviation (RMSD) of the ligand head and tail groups from the first frame of the simulation.

The same tight binding of the tail amino group and the subsequent stability of tail group atoms were observed in the simulation of the heNOS-**9** complex (Figure S9). However, the distance between the tail phenyl ring and Phe105 diverged (∼15 Å), dismissing the possibility of interaction with Phe105, the structural equivalent of His342 in the heNOS (Figure S9). Therefore, even though a His-out conformation was observed in the crystal structure of hnNOS-**9**, the stable and selective interaction with His342 described in the MD simulation may occur under physiological conditions, which probably contributes to the remarkable selectivity of **9** toward hnNOS.

#### Assessment of Permeability of the nNOS Inhibitors with the PAMPA-BBB Assay

A set of highly potent and selective nNOS inhibitors was selected for assessment of blood–brain barrier permeability using the PAMPA-BBB assay. The selected nNOS inhibitors showed moderate to poor BBB permeability, which is acceptable for the treatment of melanoma. **10** showed the best permeability (*P*_e_ = 8.70 × 10^–6^ cm s^–1^), as the head group is a lipophilic thiophene group whereas the furan derivative (**9**) showed slightly less permeability (*P*_e_ = 6.91 × 10^–6^ cm s^–1^), likely because of its polar head group. Surprisingly, **2**, bearing two 3-substituted furan rings, was not permeable (*P*_e_ = 0.23 × 10^–6^ cm s^–1^). A similar pattern was observed with the double-headed HH044 and **1**: HH044 with a thiophene was more permeable (*P*_e_ = 7.52 × 10^–6^ cm s^–1^) than the furan derivative **1** (*P*_e_ = 3.07 × 10^–6^ cm s^–1^) (Table 3).

**Table 3:** Effective Permeability (*P*_e_) of Two Commercial Drugs and Novel nNOS Inhibitors in the PAMPA-BBB Assay

compound	reported *P*_e_ (10^–6^ cm s^–1^)	determined *P*_e_ ^a^ (10^–6^ cm s^–1^)	**prediction** ^b^
(±)-verapamil	16	20.1 ± 1.26	CNS (+)
theophylline	0.12	0.08 ± 0.04	CNS (−)
HH044		7.52 ± 0.44	CNS (+)
**1**		3.07 ± 0.07	CNS (+)
**2**		0.23 ± 0.12	CNS (−)
**9**		6.91 ± 0.34	CNS (+)
**10**		8.70 ± 0.60	CNS (+)
**13**		7.48 ± 0.28	CNS (+)

a Effective permeability values were obtained in our in-house conditions. All assays were in a triplicate for each compound at 200 μM concentration over 17 h (see SI).

b CNS (+) = likely high BBB permeation. CNS (−) = likely low BBB permeation.

#### *In Vivo* Pharmacokinetic Profile of Compound 9

*In vivo* PK evaluation of compound **9** was performed in mice following intravenous (3 mg/kg) and oral (10 mg/kg) administration (Table 4). Compound **9** exhibited moderate clearance (85.4 mL/min/kg), a long half-life (11.3 h), and a large volume of distribution, indicating extensive tissue distribution. Oral bioavailability was only 8%, but measurable brain penetration (brain to plasma AUC ratio ∼2.6) was observed.

**Table 4:** *In Vivo* PK Parameters of Compound 9 in Mice Following Single Intravenous (3 mg/kg) and Oral (10 mg/kg) Administration

route	matrix	dose (mg/kg)	*T*_max_ (h)	*C*_0_/*C*_max_ (ng/mL)	AUC_last_ (h·ng/mL)	*T*_1/2_ (h)	CL (mL/min/kg)	*V*_ss_ (L/kg)	%F
IV	plasma	3		2477.24	500.82	11.27	85.37	49.55	
	brain		0.08	87.68	1056.77	0.09	2.11		
PO	plasma	10	0.50	14.46	128.81	20.16			8
	brain		48.00	10.46	336.23	0.72	2.61		

## Conclusions

In summary, we report a structure-based approach to design nNOS inhibitors for therapeutic use by promotioning interactions with the hnNOS-specific His342 residue. Using this approach, inhibitors with improved isoform selectivity and a more favorable ratio of hnNOS/rnNOS *K*_i_ were developed. We screened ten nNOS inhibitors from the developed series that showed greater antimelanoma activity compared to the previously reported lead compound HH044. In particular, **9** was the most potent in inhibiting both human (*K*_i_ = 1.7 nM) and rat nNOS (*K*_i_ = 2.3 nM) and displayed 5654-fold selectivity over human eNOS. Compound **9** also showed an EC_50_ close to 2 μM in melanoma cell lines and significantly reduced PD-L1 expression levels, both in the presence and absence of interferon-gamma. Selected nNOS inhibitors exhibited moderate to poor BBB permeability, suggesting minimal CNS toxicity liability. X-ray crystallography studies and molecular modeling of the developed nNOS inhibitors revealed the significance of the hnNOS-specific His342 residue. Molecular modeling of **9** with hnNOS demonstrated that the tail part of the inhibitor resides in the flexible part of the binding site, where the tail phenyl group π-stacking with His342 was stable with ∼5 Å distance and two additional π-interactions with Tyr711 and Trp311­(B) were also maintained. These findings provide a strong foundation for the development of first-in-class drug candidates targeting nNOS for melanoma treatment.

## Experimental Section

### Chemistry

All chemicals were purchased from Sigma-Aldrich, TCI, Combi Blocks, AA Blocks companies. Solvents such as THF, acetonitrile, and DMF used for chemical reactions were purchased from Thermo Fisher and distilled with a solvent purification system. Unless stated otherwise, all reactions were performed under nitrogen or argon. Microwave reactions were performed in a Biotage microwave synthesizer. Merck TLC silica gel 60 F254, 0.25 mm glass plates, were used for thin layer chromatography to monitor the reaction, and components were visualized by UV light (254 nM). Flash column chromatography was performed on a Teledyne CombiFlash Nextgen 300+ instrument with different column stations.

^1^H and ^13^C NMR spectra were recorded on a Bruker Avance III NMR spectrometer with operating frequencies of 500 and 126 MHz, respectively, in CDCl_3_, CD_3_OD, DMSO or D_2_O. Chemical shifts are reported in parts per million with coupling constants “*J*” in hertz (Hz), and multiplicities are indicated by s = singlet, d = doublet, t = triplet, q = quartet, sep = septet, dd = doublet of doublet, dt = doublet of triplet, m = multiplet, and br = broad resonance. High-resolution mass spectral (HRMS) data were obtained with an Agilent 6210 LC-TOF spectrometer in the positive-ion mode using electrospray ionization (ESI) with an Agilent G1312A high-performance liquid chromatography (HPLC) pump and an Agilent G1367B autoinjector at the Integrated Molecular Structure Education and Research Center (IMSERC), Northwestern University. All final products used in assays were >95% pure by Shimadzu high-performance liquid chromatography (HPLC). Detailed experimental procedures for all reaction intermediates are discussed in the Supporting Information.

### General Procedure 1: Suzuki Reaction

A Biotage microwave vial (2–6 mL) was charged with aryl halides (1 equiv), aryl boronic acid (1.1 equiv), potassium carbonate (3 equiv) and PdCl_2_(dppf) (0.02 equiv). The vial was sealed with a microwave vial cap containing a Reseal septum and evacuated/backfilled with nitrogen (×3). Dioxane (3 mL) and water (0.5 mL) were added via syringe. The resulting biphasic mixture was stirred at 70 °C for 45 min. After cooling to room temperature, the reaction mixture was filtered through Celite, and the filtrate was partitioned between water (300 mL) and EtOAc (50 mL). The organic layer was separated, and the aqueous layer extracted with EtOAc (2 × 50 mL). The combined organic phases were dried (Na_2_SO_4_), filtered, and concentrated in vacuo. The residue was purified by CombiFlash column chromatography described for individual compounds in the Supporting Information.

### General Procedure 2: Reductive Amination

To a solution of amine intermediates (1 equiv) and *m-*nitrobenzaldehyde (1.1 equiv) in anhydrous THF (10 mL) was added glacial acetic acid (0.1 equiv), and the reaction was stirred for 1 h. NaBH_3_CN (1.1 equiv) was added in portions for 10 min and stirred overnight at room temperature. The reaction was quenched with dropwise addition of MeOH (2 mL) and concentrated in vacuo. The solid residue was taken up with EtOAc (15 mL) and washed with saturated 1 M NaOH (5 mL) and brine (20 mL). The organic layer was dried over anhydrous Na_2_SO_4,_ concentrated, and dried in vacuo to yield a highly viscous orange oil. To this mixture were added di*tert*-butyl dicarbonate (1.5 equiv), NaHCO_3_ (1.2 equiv), and anhydrous acetonitrile (10 mL). The reaction mixture was stirred for 1 h at room temperature. The reaction mixture was concentrated in vacuo, and the solid residues were taken up in EtOAc (50 mL). The organic layer was washed with brine (30 mL), dried over anhydrous Na_2_SO_4_, concentrated, and dried in vacuo. The residue was purified by CombiFlash column chromatography described for individual compounds in the Supporting Information.

### General Procedure 3: Preparation of Head Groups

Triethylamine (1.2 equiv) and ammonium sulfide 20 wt % solution in water (1.2 equiv) were added to a solution of aryl carbonitrile (1 equiv) in pyridine (8 mL). The reaction mixture was stirred at 50 °C for 12h. After completion of the reaction, the mixture was diluted with cold water and extracted with ethyl acetate (3 × 50 mL). The organic layers were washed with brine, dried over Na_2_SO_4,_ and concentrated in a vacuum to give the carbothioamide intermediates. The product structures were confirmed by LCMS and were carried out to the next step without purification. A solution of carbothioamide intermediates (1 equiv) in 20 mL of acetone (reagent grade) was treated with iodomethane (1.1 equiv) and stirred for 12 h at laboratory temperature. After completion of the reaction, the reaction mixture was concentrated and washed three times with acetone and dried in vacuo to yield various head groups.

### General Procedure 4: Coupling Reactions of the Amine with Various Head Groups Containing a Carbimidothioate Moiety and Salt Formation of the Final Compounds (1–21)

To a solution of the amine (1 equiv) in EtOH (absolute, 2 mL) was added previously synthesized head groups consisting of a carbimidothioate (hydroiodide salt) (2 equiv). The reaction mixture was stirred at room temperature for 24 h, and the reaction mixture was concentrated in vacuo. The crude residue was purified as described below under subheadings for all final compounds. The semisolid was dissolved in 3 M HCl in dioxane (2 mL). The reaction mixture was stirred at room temperature for 24 h, and the reaction mixture was concentrated in vacuo. Anhydrous MeOH (1 mL) was added to the crude solid residue, and the mixture was warmed in a 40 °C water bath. To this transparent solution, anhydrous diethyl ether was added dropwise (∼3–5 mL total) until the solution turned cloudy. The resulting solid precipitate was collected by decanting the solution, washed five times with ice-cold anhydrous diethyl ether (20 mL portions), and dried in vacuo to yield all final molecules (**1–21**) as trihydrochloride salts.

### *N*-(4-(2-((3-(Furan-2-carboximidamido)­benzyl)­amino)­ethyl)­phenyl)­furan-2-carboximidamide Trihydrochloride Salt (1)

Compound **1** was prepared by following General Procedure 4 via coupling of **2b** (324 mg, 1.20 mmol) and **26** (165 mg, 0.4838 mmol). Purification by flash column chromatography using 60% EtOAc in hexane followed by salt formation as described in General Procedure 4, yielded **1** in the trihydrochloride salt form as a yellow solid (120 mg, 46%).^1^H NMR (500 MHz, CD_3_OD) δ 8.07 (dd, *J* = 10.7, 1.7 Hz, 2H), 7.84–7.66 (m, 5H), 7.58 (t, *J* = 8.7 Hz, 3H), 7.45 (d, *J* = 8.0 Hz, 2H), 6.91–6.87 (m, 2H), 4.40 (s, 2H), 3.48–3.41 (m, 2H), 3.30–3.21 (m, 2H); ^13^C NMR (125 MHz, CD_3_OD) δ 152.5, 152.2, 148.9, 148.8, 141.1, 141.0, 137.9, 134.2, 133.5, 132.5, 130.8, 130.6, 130.5, 127.3, 126.6, 126.1, 119.1, 118.8, 113.2, 113.1, 50.4, 48.4, 31.4. HRMS (ESI) calcd for C_25_H_26_N_5_O_2_ [(M + H)^+^], 428.2081; found, 428.2088.

### *N*-(4-(2-((3-(Furan-3-carboximidamido)­benzyl)­amino)­ethyl)­phenyl)­furan-3-carboximidamide Trihydrochloride Salt (2)

Compound **2** was prepared by following General Procedure 4 via coupling of **2e** (98 mg, 0.3665 mmol) and **26** (50 mg, 0.1466 mmol). Purification by flash column chromatography using 100% EtOAc followed by salt formation as described in General Procedure 4, yielded **2** in the trihydrochloride salt form as a pale yellow solid (30 mg, 38%).^1^H NMR (500 MHz, CD_3_OD) δ 8.56 (d, *J* = 21.3 Hz, 2H), 7.90–7.84 (m, 2H), 7.78 (s, 1H), 7.76–7.67 (m, 2H), 7.58 (d, *J* = 8.2 Hz, 3H), 7.46 (d, *J* = 7.9 Hz, 2H), 7.15–7.08 (m, 2H), 4.40 (s, 2H), 3.47–3.40 (m, 2H), 3.27–3.20 (m, 2H).^13^C NMR (125 MHz, CD_3_OD) δ 157.7, 157.5, 147.5, 147.3, 145.5, 145.5, 137.9, 134.4, 133.5, 132.8, 130.9, 130.6, 130.5, 127.3, 126.5, 126.1, 116.2, 108.1, 108.0, 50.4, 48.4, 31.4. HRMS (ESI) calcd for C_25_H_26_N_5_O_2_ [(M + H)^+^], 428.2081; found, 428.2080.

### *N*-(4-(2-((3-(Isoxazole-3-carboximidamido)­benzyl)­amino)­ethyl)­phenyl)­isoxazole-3-carboximidamide Trihydrochloride Salt (3)

Compound **3** was prepared by following General Procedure 4 via coupling of **2f** (69 mg, 0.2565 mmol) and **26** (35 mg, 0.1026 mmol). Purification by flash column chromatography using 5% methanol in dichloromethane followed by salt formation as described in General Procedure 4, gave **3** in the trihydrochloride salt form as a white solid (20 mg, 36%). ^1^H NMR (500 MHz, CD_3_OD) δ 9.16 (dd, *J* = 7.3, 1.4 Hz, 2H), 7.92–7.68 (m, 3H), 7.62 (d, *J* = 7.3 Hz, 3H), 7.50 (d, *J* = 7.4 Hz, 2H), 7.30 (dd, *J* = 20.3, 1.6 Hz, 2H), 4.42 (s, 2H), 3.49–3.42 (m, 2H), 3.27–3.24 (m, 2H). ^13^C NMR (125 MHz, CD_3_OD) δ 162.7, 154.5, 154.3, 153.5, 138.4, 133.8, 133.6, 132.2, 131.1, 131.0, 130.8, 127.3, 126.5, 126.0, 104.1, 103.9, 50.5, 48.5, 31.5. HRMS (ESI) calcd for C_23_H_24_N_7_O_2_ [(M + H)^+^], 430.1986; found, 430.1987.

### *N*-(4-(2-((3-(Thiophene-3-carboximidamido)­benzyl)­amino)­ethyl)­phenyl)­thiophene-3-carboximidamide Trihydrochloride Salt (4)

Compound **4** was prepared by following General Procedure 4 via coupling of **2g** (100 mg, 0.3519 mmol) and **26** (40 mg, 0.1173 mmol). Purification by flash column chromatography using 10% methanol in dichloromethane followed by salt formation as described in General Procedure 4, gave **4** in the trihydrochloride salt form as a pale yellow solid (20 mg, 33%). ^1^H NMR (500 MHz, CD_3_OD) δ 8.60–8.49 (m, 2H), 7.80 (td, *J* = 5.8, 2.9 Hz, 3H), 7.75–7.66 (m, 4H), 7.59 (d, *J* = 7.9 Hz, 3H), 7.47 (d, *J* = 8.2 Hz, 2H), 4.40 (s, 2H), 3.48–3.35 (m, 2H), 3.26–3.17 (m, 2H); ^13^C NMR (125 MHz, CD_3_OD) δ 158.7, 158.5, 137.8, 134.7, 133.5, 133.1, 132.7, 132.4, 130.9, 130.6, 130.4, 130.3, 129.3, 128.5, 127.2, 126.5, 126.0, 125.9, 125.8, 50.4, 48.4, 31.4. HRMS (ESI) calcd for C_25_H_26_N_5_S_2_ [(M + H)^+^], 460.1624; found, 460.1608.

### *N*-(4-(2-((3-(Thiazole-2-carboximidamido)­benzyl)­amino)­ethyl)­phenyl)­thiazole-2-carboximidamide Trihydrochloride Salt (5)

Compound **5** was prepared by following General Procedure 4 via coupling of **2c** (280 mg, 0.9853 mmol) and **26** (120 mg, 0.3519 mmol). Purification by flash column chromatography using 70% EtOAc in hexane followed by salt formation as described in General Procedure 4, yielded **5** in the trihydrochloride salt form as a yellow solid (50 mg, 25%).^1^H NMR (500 MHz, CD_3_OD) δ 8.29 (d, *J* = 8.4 Hz, 1H), 7.83 (s, 1H), 7.79–7.70 (m, 2H), 7.61 (q, *J* = 6.5 Hz, 3H), 7.51 (d, *J* = 8.2 Hz, 2H), 4.42 (s, 2H), 3.46 (dd, *J* = 9.9, 6.2 Hz, 2H), 3.25 (dd, *J* = 9.9, 6.0 Hz, 2H). ^13^C NMR (125 MHz, CD_3_OD) δ 155.3, 155.1, 153.7, 153.6 145.0, 145.0, 138.2, 134.2, 133.5, 132.6, 130.9, 130.8, 130.7, 129.7, 127.7, 127.5, 127.4, 126.6, 126.1, 123.5, 50.5, 48.4, 31.4. HRMS (ESI) calcd for C_23_H_24_N_7_S_2_ [(M + H)^+^], 462.1529; found, 462.1539.

### *N*-(4-(2-((3-(Thiazole-5-carboximidamido)­benzyl)­amino)­ethyl)­phenyl)­thiazole-5-carboximidamide Trihydrochloride Salt (6)

Compound **6** was prepared by following General Procedure 4 via coupling of **2d** (180 mg, 0.6334 mmol) and **26** (90 mg, 0.2639 mmol). Purification by flash column chromatography using 60% EtOAc in hexane followed by salt formation as described in General Procedure 4, gave **6** in the trihydrochloride salt form as a pale yellow solid (30 mg, 20%). ^1^H NMR (500 MHz, CD_3_OD) δ 9.40 (d, *J* = 33.5 Hz, 2H), 8.69 (d, *J* = 23.5 Hz, 2H), 7.69–7.53 (m, 5H), 7.46 (d, *J* = 8.2 Hz, 3H), 4.37 (s, 1H), 3.44–3.38 (m, 1H), 3.22–3.18 (m, 1H). ^13^C NMR (125 MHz, CD_3_OD) δ 159.8, 159.2, 154.7, 148.8, 147.7, 138.9, 137.6, 133.2, 130.7, 130.5, 130.3, 128.9, 126.1, 125.6, 125.4, 122.2, 50.6, 48.2, 31.4. HRMS (ESI) calcd for C_23_H_24_N_7_S_2_ [(M + H)^+^], 462.1529; found, 462.1526.

### *N*-(3-(2-((3-(Thiazole-5-carboximidamido)­benzyl)­amino)­ethyl)­phenyl)­thiazole-5-carboximidamide Trihydrochloride Salt (7)

Compound **7** was prepared by following General Procedure 4 via coupling of **2d** (200 mg, 0.6304 mmol) and **29** (86 mg, 0.2521 mmol). Purification by flash column chromatography using 60% EtOAc in hexane followed by salt formation as described in General Procedure 4, gave **7** in trihydrochloride salt form as a white solid (40 mg, 26%). ^1^H NMR (500 MHz, CD_3_OD) δ 8.07 (d, *J* = 7.3 Hz, 2H), 7.81–7.66 (m, 5H), 7.62–7.53 (m, 2H), 7.50 (d, *J* = 7.3 Hz, 2H), 7.39 (d, *J* = 8.4 Hz, 1H), 6.89 (ddd, *J* = 4.5, 3.7, 1.8 Hz, 2H), 4.40 (s, 2H), 3.50–3.42 (m, 2H), 3.25–3.23 (m, 2H). ^13^C NMR (125 MHz, CD_3_OD) δ 152.4, 152.2, 148.9, 148.8, 141.1, 141.0, 139.2, 134.1, 134.0, 133.5, 130.8, 130.6, 130.5, 129.4, 127.3, 126.6, 126.1, 124.4, 119.1, 118.8, 113.2, 113.1, 50.5, 48.3, 31.5. HRMS (ESI) calcd for C_23_H_24_N_7_S_2_ [(M + H)^+^], 462.1529; found, 462.15238.

### *N*-(3-(2-((3-(Furan-2-carboximidamido)­benzyl)­amino)­ethyl)­phenyl)­furan-2-carboximidamide Trihydrochloride Salt (8)

Compound **8** was prepared by following General Procedure 4 via coupling of **2b** (265 mg, 0.9912 mmol) and **29** (130 mg, 0.3812 mmol). Purification by flash column chromatography using 70% EtOAc in hexane followed by salt formation as described in General Procedure 4, gave **8** in the trihydrochloride salt form as a white solid (100 mg, 49%). ^1^H NMR (500 MHz, CD_3_OD) δ 8.07 (dd, *J* = 10.7, 1.7 Hz, 2H), 7.84–7.66 (m, 5H), 7.58 (t, *J* = 8.7 Hz, 3H), 7.45 (d, *J* = 8.0 Hz, 2H), 6.91–6.87 (m, 2H), 4.40 (s, 2H), 3.48–3.41 (m, 2H), 3.30–3.21 (m, 2H); ^13^C NMR (125 MHz, CD_3_OD) δ 152.5, 152.2, 148.9, 148.8, 141.1, 141.0, 137.9, 134.2, 133.5, 132.5, 130.8, 130.6, 130.5, 127.3, 126.6, 126.1, 119.1, 118.8, 113.2, 113.1, 50.4, 48.4, 31.4. HRMS (ESI) calcd for C_25_H_26_N_5_O_2_ [(M + H)^+^], 428.2081; found, 428.2080.

### *N-*(3-(((2-(3-(Aminomethyl)-[1,1′-biphenyl]-4-yl)­ethyl)­amino)­methyl)­phenyl)­furan-2-carboximidamide Trihydrochloride Salt (9)

Compound **9** was prepared by following General Procedure 4 via coupling of **2b** (33 mg, 0.1225 mmol) and **61** (43 mg, 0.0680 mmol). Purification by flash column chromatography using 80% EtOAc in hexane followed by salt formation as described in General Procedure 4, yielded **9** in the trihydrochloride salt form as a white yellow solid (20 mg, 55%). ^1^H NMR (500 MHz, D_2_O) δ 7.93 (s, 1H), 7.76–7.68 (m, 5H), 7.64–7.53 (m, 5H), 7.49–7.44 (m, 3H), 6.76–6.72 (m, 1H), 4.38 (s, 2H), 4.35 (s, 2H), 3.42 (t, *J* = 8.0 Hz, 2H), 3.24–3.21 (m, 2H). ^13^C NMR (125 MHz, D_2_O) δ 152.4, 149.0, 140.5, 139.2, 134.1, 133.8, 132.8, 131.6, 131.3, 130.9, 130.4, 129.2, 128.1, 128.1, 128.0, 127.0, 126.9, 126.7, 119.3, 113.4, 50.4, 47.1, 39.7, 28.1. HRMS (ESI) calcd for C_27_H_29_N_4_O [(M + H)^+^], 425.2336; found, 425.2344.

### *N*-(3-(((2-(3-(Aminomethyl)-[1,1′-biphenyl]-4-yl)­ethyl)­amino)­methyl)­phenyl)­thiophene-2-carboximidamide Trihydrochloride Salt (10)

Compound **10** was prepared by following General Procedure 4 via coupling of **2a** (35 mg, 0.1225 mmol) and **61** (43 mg, 0.0680 mmol). Purification by flash column chromatography using 70% EtOAc in hexane followed by salt formation as described in General Procedure 4, gave **10** in the trihydrochloride salt form as a yellow solid (22 mg, 61%). ^1^H NMR (500 MHz, D_2_O) δ 8.03–7.96 (m, 2H), 7.79–7.68 (m, 5H), 7.64 (d, *J* = 7.8 Hz, 1H), 7.56 (dt, *J* = 15.2, 7.7 Hz, 3H), 7.51–7.44 (m, 3H), 7.36 (s, 1H), 4.39 (s, 2H), 4.36 (s, 2H), 3.42 (t, *J* = 7.7 Hz, 2H), 3.23 (t, *J* = 8.0 Hz, 2H). ^13^C NMR (125 MHz, D_2_O) δ 158.0, 140.4, 139.2, 134.5, 134.4, 134.0, 132.1, 131.6, 131.3, 130.9, 130.4, 129.2, 128.9, 128.1, 128.1, 128.0, 126.9, 126.8, 50.4, 47.1, 39.8, 28.1. HRMS (ESI) calcd for C_27_H_29_N_4_S [(M + H)^+^], 441.2107; found, 441.2118.

### *N*-(3-(((2-(3-(Aminomethyl)-[1,1′-biphenyl]-4-yl)­ethyl)­amino)­methyl)­phenyl)­thiazole-5-carboximidamide Trihydrochloride Salt (11)

Compound **11** was prepared by following General Procedure 4 via coupling of **2d** (35 mg, 0.1225 mmol) and **61** (43 mg, 0.0680 mmol). Purification by flash column chromatography using 60% EtOAc in hexane followed by salt formation as described in General Procedure 4, produced **11** in the trihydrochloride salt form as a white solid (25 mg, 66%). ^1^H NMR (500 MHz, D_2_O) δ 9.41 (s, 1H), 8.64 (s, 1H), 7.79–7.69 (m, 5H), 7.64 (d, *J* = 7.7 Hz, 1H), 7.61–7.52 (m, 3H), 7.50–7.40 (m, 3H), 4.39 (s, 2H), 4.36 (s, 2H), 3.48–3.38 (m, 2H), 3.23 (t, *J* = 7.9 Hz, 2H). ^13^C NMR (125 MHz, D_2_O) δ 160.9, 156.5, 147.6, 140.5, 139.2, 134.3, 134.1, 132.8, 131.7, 131.4, 130.9, 130.6, 129.2, 128.2, 128.1, 128.0, 126.8, 126.6, 125.7, 50.3, 47.1, 39.7, 28.1. HRMS (ESI) calcd for C26H28N5S [(M + H)^+^], 442.2060; found, 442.2073

### *N*-(3-(((2-(3-(Aminomethyl)-[1,1′-biphenyl]-4-yl)­ethyl)­amino)­methyl)­phenyl)­furan-3-carboximidamide Trihydrochloride Salt (12)

Compound **12** was prepared by following General Procedure 4 via coupling of **2e** (38 mg, 0.1411 mmol) and **61** (50 mg, 0.0783 mmol). Purification by flash column chromatography using 70% EtOAc in hexane followed by salt formation as described in General Procedure 4, gave **12** in the trihydrochloride salt form as a yellow-white solid (26 mg, 62%). ^1^H NMR (500 MHz, D_2_O) δ 8.39 (s, 1H), 7.80–7.68 (m, 6H), 7.64 (d, *J* = 7.8 Hz, 1H), 7.56 (q, *J* = 7.0 Hz, 3H), 7.51–7.43 (m, 3H), 6.96 (s, 1H), 4.39 (s, 2H), 4.36 (s, 2H), 3.42 (t, *J* = 8.0 Hz, 2H), 3.23 (t, *J* = 8.0 Hz, 2H). ^13^C NMR (125 MHz, D_2_O) δ 157.8, 147.2, 145.6, 140.4, 139.2, 134.0, 134.0, 132.8, 131.6, 131.3, 130.9, 130.5, 129.2, 128.1, 128.1, 128.0, 127.0, 126.8, 115.7, 108.1, 50.4, 47.2, 39.8, 28.1. HRMS (ESI) calcd for C_27_H_29_N_4_O [(M + H)^+^], 425.2336; found, 425.2349.

### *N-*(3-(((2-(3-(Aminomethyl)-4′-fluoro-[1,1′-biphenyl]-4-yl)­ethyl)­amino)­methyl)­phenyl)­furan-2-carboximidamide Trihydrochloride Salt (13)

Compound **13** was prepared by following General Procedure 4 via coupling of **2b** (26 mg, 0.9695 mmol) and **64** (35 mg, 0.0538 mmol). Purification by flash column chromatography using 80% EtOAc in hexane followed by salt formation as described in General Procedure 4, produced **13** in the trihydrochloride salt form as a yellow solid (18 mg, 60%). ^1^H NMR (500 MHz, D_2_O) δ 7.85 (s, 1H), 7.61 (dd, *J* = 15.1, 8.1 Hz, 5H), 7.54 (d, *J* = 7.8 Hz, 1H), 7.48 (t, *J* = 7.5 Hz, 2H), 7.38 (d, *J* = 7.9 Hz, 1H), 7.31 (s, 1H), 7.15 (t, *J* = 8.6 Hz, 2H), 6.73 (d, *J* = 3.8 Hz, 1H), 4.29 (s, 2H), 4.25 (s, 2H), 3.33 (t, *J* = 7.9 Hz, 2H), 3.13 (t, *J* = 7.8 Hz, 2H). ^13^C NMR (125 MHz, D_2_O) δ 163.5, 161.5, 152.3, 149.0, 140.4, 139.6, 135.3 (d, *J* = 3.2 Hz), 134.0, 133.7, 132.7, 131.6, 131.3, 130.5, 130.4, 128.5 (d, *J* = 8.4 Hz), 128.0 (d, *J* = 4.3 Hz), 126.9 (d, *J* = 7.4 Hz), 119.3, 115.8 (d, *J* = 21.7 Hz), 113.4, 50.3, 47.0, 39.7, 28.0. HRMS (ESI) calcd for C_27_H_28_FN_4_O [(M + H)^+^], 443.2242; found, 443.2258.

### *N-*(3-(((2-(3-(Aminomethyl)-4′-fluoro-[1,1′-biphenyl]-4-yl)­ethyl)­amino)­methyl)­phenyl)­furan-3-carboximidamide Trihydrochloride Salt (14)

Compound **14** was prepared by following General Procedure 4 via coupling of **2e** (26 mg, 0.9695 mmol) and **64** (35 mg, 0.0538 mmol). Purification by flash column chromatography using 80% EtOAc in hexane followed by salt formation as described in General Procedure 4, yielded **14** in the trihydrochloride salt form as a yellow solid (20 mg, 67%). ^1^H NMR (500 MHz, D_2_O) δ 8.39 (s, 1H), 7.78 (s, 1H), 7.74–7.66 (m, 5H), 7.63 (d, *J* = 7.8 Hz, 1H), 7.57 (d, *J* = 8.0 Hz, 1H), 7.50–7.43 (m, 2H), 7.29–7.21 (m, 2H), 6.96 (s, 1H), 4.39 (s, 2H), 4.35 (s, 2H), 3.42 (t, *J* = 8.0 Hz, 2H), 3.22 (t, *J* = 7.9 Hz, 2H). ^13^C NMR (125 MHz, D_2_O) δ 163.5, 161.5, 157.8, 147.1, 145.6, 139.6, 135.4 (d, *J* = 3.0 Hz), 134.0, 132.8, 131.6, 131.3, 130.9, 130.5, 128.6 (d, *J* = 8.4 Hz), 128.0 (d, *J* = 5.1 Hz), 127.0, 115.8 (d, *J* = 21.7 Hz), 108.1, 50.4, 47.1, 39.7, 28.0. HRMS (ESI) calcd for C_27_H_28_FN_4_O [(M + H)^+^], 443.2242; found, 443.22570.

### *N*-(3-(((2-(3-(Aminomethyl)-3′-fluoro-[1,1′-biphenyl]-4-yl)­ethyl)­amino)­methyl)­phenyl)­furan-2-carboximidamide Trihydrochloride Salt (15)

Compound **15** was prepared by following General Procedure 4 via coupling of **2b** (41 mg, 0.1539 mmol) and **62** (50 mg, 0.0769 mmol). Purification by flash column chromatography using 80% EtOAc in hexane followed by salt formation as described in General Procedure 4, gave **15** in the trihydrochloride salt form as a pale yellow solid (24 mg, 56%). ^1^H NMR (500 MHz, D_2_O) δ 7.94 (s, 1H), 7.76–7.67 (m, 3H), 7.64 (d, *J* = 7.8 Hz, 1H), 7.60–7.45 (m, 2H), 7.49 (q, *J* = 7.2 Hz, 3H), 7.44–7.38 (m, 2H), 7.16 (tt, *J* = 8.7, 3.8 Hz, 1H), 6.81 (t, *J* = 2.7 Hz, 1H), 4.39 (s, 2H), 4.35 (s, 2H), 3.42 (t, *J* = 7.9 Hz, 2H), 3.23 (t, *J* = 8.0 Hz, 2H). ^13^C NMR (125 MHz, D_2_O) δ 163.9, 162.0, 152.3, 149.0, 141.4 (d, *J* = 7.8 Hz), 140.4, 139.2 (d, *J* = 2.3 Hz), 134.7, 133.7, 132.7, 131.7, 131.3, 130.9, 130.8 (d, *J* = 8.6 Hz), 130.5, 128.1 (d, *J* = 1.7 Hz), 126.9 (d, *J* = 4.8 Hz), 122.5 (d, *J* = 2.6 Hz), 119.3, 114.7, 114.5, 113.5, 113.3 (d, *J* = 7.3 Hz), 50.4, 47.1, 39.7, 28.1. HRMS (ESI) calcd for C_27_H_28_FN_4_O [(M + H)^+^], 443.2242; found, 443.2252.

### *N*-(3-(((2-(3-(Aminomethyl)-2′-fluoro-[1,1′-biphenyl]-4-yl)­ethyl)­amino)­methyl)­phenyl)­furan-2-carboximidamide Trihydrochloride Salt (16)

Compound **16** was prepared by following General Procedure 4 via coupling of **2b** (37 mg, 0.1385 mmol) and **62** (45 mg, 0.0692 mmol). Purification by flash column chromatography using 70% EtOAc in hexane followed by salt formation as described in General Procedure 4, gave **16** in the trihydrochloride salt form as a pale yellow solid (24 mg, 63%). ^1^H NMR (500 MHz, D_2_O) δ 7.93 (s, 1H), 7.73–7.66 (m, 3H), 7.64–7.53 (m, 4H), 7.51–7.43 (m, 2H), 7.40 (s, 1H), 7.34 (t, *J* = 7.5 Hz, 1H), 7.28 (dd, *J* = 11.3, 8.3 Hz, 1H), 6.84–6.79 (m, 1H), 4.37 (s, 2H), 4.34 (s, 2H), 3.42 (t, *J* = 7.8 Hz, 2H), 3.23 (t, *J* = 7.9 Hz, 2H). ^13^C NMR (125 MHz, D_2_O) δ 160.3, 158.3, 152.4, 148.9, 140.6, 135.5, 134.5, 132.8, 131.3 (d, *J* = 10.7 Hz), 130.5, 130.5 (d, *J* = 3.5 Hz), 130.3, 130.2, 130.0 (d, *J* = 7.8 Hz), 127.1, 127.0, 126.9 (d, *J* = 12.5 Hz), 124.8 (d, *J* = 4.1 Hz), 119.1, 116.1 (d, *J* = 22.5 Hz), 113.3, 50.4, 47.0, 39.6, 28.1. HRMS (ESI) calcd for C_27_H_28_FN_4_O [(M + H)^+^], 443.2242; found, 443.2255.

### *N*-(3-(((2-(3-(Aminomethyl)-3′,5′-difluoro-[1,1′-biphenyl]-4-yl)­ethyl)­amino)­methyl)­phenyl)­furan-2-carboximidamide Trihydrochloride Salt (17)

Compound **17** was prepared by following General Procedure 4 via coupling of **2b** (30 mg, 0.1124 mmol) and **65** (50 mg, 0.0749 mmol). Purification by flash column chromatography using 80% EtOAc in hexane followed by salt formation as described in General Procedure 4, produced **17** in the trihydrochloride salt form as a pale yellow solid (26 mg, 60%). ^1^H NMR (500 MHz, D_2_O) δ 7.90 (s, 1H), 7.71–7.59 (m, 4H), 7.58–7.50 (m, 2H), 7.45 (d, *J* = 8.0 Hz, 1H), 7.40 (d, *J* = 1.9 Hz, 1H), 7.25–7.17 (m, 2H), 6.91 (td, *J* = 9.2, 2.1 Hz, 1H), 6.80–6.76 (m, 1H), 4.37 (s, 2H), 4.32 (s, 2H), 3.40 (t, *J* = 8.0 Hz, 2H), 3.21 (t, *J* = 8.0 Hz, 2H). ^13^C NMR (125 MHz, D_2_O) δ 164.0 (d, *J* = 13.5 Hz), 162.0 (d, *J* = 13.5 Hz), 152.2, 149.0, 142.4 (t, *J* = 9.8 Hz), 140.3, 138.1, 135.2, 133.6, 132.6, 131.7, 131.3, 131.0, 130.5, 128.1 (d, *J* = 5.1 Hz), 126.9 (d, *J* = 2.0 Hz), 119.3, 113.3, 109.6 (d, *J* = 6.3 Hz), 109.5 (d, *J* = 6.3 Hz), 102.8 (t, *J* = 25.9 Hz), 50.3, 47.0, 39.7, 28.1. HRMS (ESI) calcd for C_27_H_27_F_2_N_4_O [(M + H)^+^], 461.2147; found, 461.2161

### *N*-(3-(((2-(3-((Methylamino)­methyl)-[1,1′-biphenyl]-4-yl)­ethyl)­amino)­methyl)­phenyl)­furan-2-carboximidamide Trihydrochloride Salt (18)

Compound **18** was prepared by following General Procedure 4 via coupling of **2b** (40 mg, 0.1467 mmol) and **71** (40 mg, 0.0733 mmol). Purification by flash column chromatography using 5% methanol in dichloromethane followed by salt formation as described in General Procedure 4, gave **18** in the trihydrochloride salt form as a yellow solid (21 mg, 52%). ^1^H NMR (500 MHz, D_2_O) δ 7.94 (s, 1H), 7.81–7.76 (m, 2H), 7.71 (dd, *J* = 11.8, 7.7 Hz, 3H), 7.62 (d, *J* = 7.8 Hz, 1H), 7.57–7.45 (m, 6H), 7.43 (d, *J* = 2.6 Hz, 1H), 6.82 (t, *J* = 2.8 Hz, 1H), 4.38–4.37 (m, 4H), 3.41 (t, *J* = 7.8 Hz, 2H), 3.24 (t, *J* = 7.9 Hz, 2H), 2.82 (s, 3H). ^13^C NMR (125 MHz, D_2_O) δ 152.4, 149.0, 140.5, 140.4, 139.0, 134.6, 133.8, 132.7, 131.3, 131.0, 130.4, 129.9, 129.2, 129.1, 128.6, 128.2, 127.0, 126.9, 126.7, 119.3, 113.4, 50.3, 48.9, 47.2, 32.4, 28.1. HRMS (ESI) calcd for C_28_H_31_N_4_O [(M + H)^+^], 439.2492; found, 439.2504.

### *N*-(3-(((2-(3-((Methylamino)­methyl)-[1,1′-biphenyl]-4-yl)­ethyl)­amino)­methyl)­phenyl)­furan-3-carboximidamide Trihydrochloride Salt (19)

Compound **19** was prepared by following General Procedure 4 via coupling of **2e** (40 mg, 0.1467 mmol) and **71** (40 mg, 0.0733 mmol). Purification by flash column chromatography using 60% EtOAc in hexane followed by salt formation as described in General Procedure 4, yielded **19** in the trihydrochloride salt form as a yellow solid (23 mg, 57%). ^1^H NMR (500 MHz, D_2_O) δ 8.29 (s, 1H), 7.70 (d, *J* = 7.1 Hz, 3H), 7.63 (t, *J* = 7.7 Hz, 3H), 7.53 (d, *J* = 7.9 Hz, 1H), 7.47 (t, *J* = 7.7 Hz, 3H), 7.43–7.33 (m, 3H), 6.86 (s, 1H), 4.30–4.29 (m, 2H), 3.34–3.30 (m, 2H), 3.14 (t, *J* = 8.0 Hz, 2H), 2.74 (s, 3H). ^13^C NMR (125 MHz, D_2_O) δ 157.8, 155.6, 147.1, 145.6, 140.5, 139.1, 134.6, 134.0, 132.8, 131.3, 131.0, 130.5, 129.9, 129.2, 129.1, 128.6, 128.2, 127.0, 126.9, 126.8, 115.7, 108.0, 50.4, 48.9, 47.2, 32.4, 28.1. HRMS (ESI) calcd for C_28_H_31_N4O [(M + H)^+^]; 439.2492. found, 439.2503.

### *N*-(3-(((2-(3-((Methylamino)­methyl)-[1,1′-biphenyl]-4-yl)­ethyl)­amino)­methyl)­phenyl)­thiophene-2-carboximidamide Trihydrochloride Salt (20)

Compound **20** was prepared by following General Procedure 4 via coupling of **2a** (40 mg, 0.1467 mmol) and **71** (40 mg, 0.0733 mmol). Purification by flash column chromatography using 60% EtOAc in hexane followed by salt formation as described in General Procedure 4, gave **20** in the trihydrochloride salt form as a yellow solid (24 mg, 58%). ^1^H NMR (500 MHz, D_2_O) δ 8.06–7.92 (m, 2H), 7.79–7.77 (m, 2H), 7.73–7.70 (m, 3H), 7.65–7.45 (m, 7H), 7.36 (s, 1H), 4.39–4.38 (m, 4H), 3.41–3.40 (m, 2H), 3.24 (t, *J* = 7.9 Hz, 2H), 2.83 (s, 3H).^13^C NMR (125 MHz, D_2_O) δ 158.1, 140.5, 139.1, 134.6, 134.5, 134.0, 132.8, 131.3, 131.0, 130.4, 130.0, 129.2, 129.1, 128.9, 128.6, 128.2, 126.9, 126.8, 50.4, 48.9, 47.2, 32.4, 28.1. HRMS (ESI) calcd for C_28_H_31_N_4_S [(M + H)^+^], 455.2264; found, 455.2281.

### *N*-(3-(((2-(3-((Methylamino)­methyl)-[1,1′-biphenyl]-4-yl)­ethyl)­amino)­methyl)­phenyl)­thiazole-5-carboximidamide Trihydrochloride Salt (21)

Compound **21** was prepared by following General Procedure 4 via coupling of **2d** (40 mg, 0.1467 mmol) and **71** (40 mg, 0.0733 mmol). Purification by flash column chromatography using 60% EtOAc in hexane followed by salt formation as described in General Procedure 4, produced **21** in the trihydrochloride salt form as a yellow solid (22 mg, 53%). ^1^H NMR (500 MHz, D_2_O) δ 9.40 (s, 1H), 8.63 (s, 1H), 7.82–7.76 (m, 2H), 7.73–7.69 (m, 3H), 7.63 (d, *J* = 7.8 Hz, 1H), 7.59–7.46 (m, 5H), 7.42 (s, 1H), 4.39–4.35 (m, 4H), 3.42–3.40 (m, 2H), 3.24 (t, *J* = 7.9 Hz, 2H), 2.83 (s, 3H). ^13^C NMR (125 MHz, D_2_O) δ 160.9, 156.4, 147.5, 140.6, 139.0, 134.6, 132.8, 131.4, 131.0, 130.5, 130.0, 129.2, 129.1, 128.6, 128.2, 126.8, 126.7, 126.6, 50.3, 48.9, 47.1, 32.4, 28.1. HRMS (ESI) calcd for C_27_H_30_N_5_S [(M + H)^+^], 456.2216; found, 456.2224.

### MTT Colorimetric Assay

Cell viability was determined after treatments with nNOS inhibitors in human melanoma A375 and SK-MEL-28 cell lines, in comparison to human melanoblast Hermes 1 cells^15^ and primary fibroblast cells^37^ (generously provided by Dr. Jennifer Totonchy, Chapman University). The protocol for the cytotoxicity assay was described previously.^36^ Half-maximal effective concentration (EC_50_) was determined using GraphPad Prism 10 through log­(inhibitor) versus normalized response analysis.

### PD-L1-Level Detection via Flow Cytometry

The PD-L1 antibodies used were conjugated with Alexa Fluor 647 (#41726S; Cell Signaling Technology, Danvers, MA). The staining protocol for PD-L1 expression was described previously.^15^ Mean fluorescence was determined using the BD FAC Symphony A1 Cell Analyzer (BD Biosciences, Franklin Lakes, NJ, USA).

## Supplementary Material





## Notes

NOS enzyme inhibition assay and permeability assay; crystallographic data collection and refinement statistics for rat nNOS, human nNOS, and human eNOS; computational details for MD simulation; synthetic details and characterization for the key intermediates and the final compounds; and copies of NMR spectra and HPLC traces for the final compounds (**1–21**) (PDF)
Molecular formula strings (CSV)

## Notes

PDB codes for X-ray structures described in this study are as follows: hnNOS-HH044, 9MWA; hnNOS-**1**, 9MWB; hnNOS-**9**, 9MWC; rnNOS-**1**, 9MWD; rnNOS-**2**, 9MWE; rnNOS-**3**, 9MWF; rnNOS-**5**, 9MWG; rnNOS-**6**, 9MWH; rnNOS-**7**, 9MWI; rnNOS-**8**, 9MWJ; rnNOS-**9**, 9MWK; rnNOS-**10**, 9MWL; rnNOS-**11**, 9MWM; heNOS-HH044, 9MWN; heNOS-**1**, 9MWO; heNOS-**2**, 9MWP; heNOS-**3**, 9MWQ; heNOS-**5**, 9MWR; heNOS-**6**, 9MWX; heNOS-**7**, 9MWS; heNOS-**8**, 9MWT; heNOS-**9**, 9MWU; heNOS-**10**, 9MWV; and heNOS-**11**, 9MWW. The authors will release the atomic coordinates upon article publication.

## Abbreviations

AUC_l__a__s__t_: area under the concentration–time curve from time zero to last measurable concentration
BBB: blood–brain barrier
BH_4_: (6*R*)-5,6,7,8-tetrahydrobiopterin
CL: clearance
*C*_max_/*C*_0_: maximum concentration/initial concentration
CNS: central nervous system
eNOS: endothelial nitric oxide synthase
FAD: flavin adenine dinucleotide
F: fraction (used in bioavailability, sometimes %F)
hnNOS: human neuronal nitric oxide synthase
iNOS: inducible nitric oxide synthase
IC_50_: half maximal inhibitory concentration
IV: intravenous
*K*_i_: inhibition constant
l-Arg: l-arginine
nNOS: neuronal nitric oxide synthase
NO: nitric oxide
PAMPA: parallel artificial membrane permeability assay
PO: oral (per os)
rnNOS: rat neuronal nitric oxide synthase
*T*_1/2_: half-life
*T*_max_: time to reach maximum concentration
*V*_ss_: volume of distribution at steady state
