# Supplementary material for: New Inhibitors of Neuronal Nitric Oxide Synthase for the Treatment of Melanoma
Source: J Med Chem. 2026 Jan 30;69(3):2310–29. doi: 10.1021/acs.jmedchem.5c02154 (PMC12895717; doi:10.1021/acs.jmedchem.5c02154)
Supplement: Supplementary file 1 [file jm5c02154_si_001.pdf]

Supporting Information

New Inhibitors of Neuronal Nitric Oxide Synthase for the Treatment of Melanoma

Amardeep Awasthi[†], Anika Patel[€], Huiying Li[§], Koon Mook Kang[†], Christine D. Hardy[§], Anas Ansari[†], Raghad Nowar[†], Md. Emtiaz Hasan[€], Sun Yang[€], Thomas L. Poulos[*, §], and Richard B. Silverman[*, †, ¶]

[†] Department of Chemistry, Department of Molecular Biosciences, Chemistry of Life Processes Institute, Center for Developmental Therapeutics, Northwestern University, 2145 Sheridan Road, Evanston, Illinois 60208-3113, United States

[¶] Department of Pharmacology, Feinberg School of Medicine, Northwestern University, Chicago, Illinois 60611, United States

[€] Department of Pharmacy Practice, Chapman University School of Pharmacy, Harry and Diane Rinker Health Science Campus, 9401 Jeronimo Road, Irvine, CA 92618, USA

[§] Departments of Molecular Biology and Biochemistry, Pharmaceutical Sciences, and Chemistry, University of California, Irvine, California 92697-3900, United States

*Corresponding authors:

Thomas L. Poulos

http://orcid.org/0000-0002-5648-3510 ; Email: poulos@uci.edu Tel: +1 949 824 7020

Richard B. Silverman

http://orcid.org/0000-0001-9034-1084 ; Email: Agman@chem.northwestern.edu Tel: +1 847 491 5653. Fax: +1 847 491 7713

Table of Contents:

S. No		Pages
1	NOS Enzyme Inhibition Assay Protocol	S3
2	PAMPA BBB assay Protocol	S3-4
3	Preparation of NOS heme domain samples for crystallography	S5-6
4	Inhibitor Complex Crystal Preparation	S6
5	X-ray Diffraction Data Collection, Data Processing, and Structural Refinement	S7-8
6	Table S1. Crystallographic data collection and refinement statistics	S8-15
7	Compounds **2**, **3**, **5**, **6**, and **7** with Figures S1, S2, S3, and S4	S16-21
8	Computational Details: Materials and Methods	S22-27
9	Melanoma Assay details: Materials and Methods	S28
10	DAF-FM NO experiment: Materials and Methods	S29-S30
11	Experimental procedures	S31-49
12	Copies of ^{1}H NMR and ^{13}C NMR	S50-70
13	HPLC traces	S71-81
14	References	S82-84

1. NOS Enzyme Inhibition Assay Protocol

The NOS inhibitory activity of **1-21** was measured by the hemoglobin (Hb) NO capture assay following a protocol described previously.[1-2] The production of NO was monitored by the rapid oxidation of oxyhemoglobin (oxyHb) to methemoglobin (metHb) by NO.[1]

Purified recombinant full-length NOSs, including rat nNOS (rnNOS),[3-4] human nNOS (hnNOS),[5] human iNOS (hiNOS),[6] and human eNOS (heNOS),[5] were used in activity assays. These proteins were expressed in *Escherichia coli* and purified as described previously.[3-6] Activity assays were performed in 100 mM HEPES (4-(2-hydroxyethyl)-1-piperazine-ethanesulfonic acid) buffer with 10% glycerol (pH 7.4-7.5) at 37 °C in the presence of 10 μM L-Arg, 10 μM H_4B, 100 μM NADPH, 0.83 mM $CaCl_2$, 320 units/mL calmodulin, and 3 μM human oxyhemoglobin. A concentration of L-Arg of 10 μM was used as it does not cause NOS uncoupling and is close to the K_m values of all three NOS isoforms so that competitive inhibitors can be detected effectively. In the case of iNOS, $CaCl_2$ and calmodulin were omitted and replaced by HEPES buffer (100 mM, 10 % glycerol, pH 7.4-7.5) because iNOS activation is calcium-independent. The assay was performed in 96-well plates using a Biotek Gen5 microplate reader, with NOS enzymes and hemoglobin dispensed automatically by the plate reader. NO production was kinetically monitored at 401 nm for 6 min. The inhibition constants (K_i) for all NOSs were calculated from the IC_{50} values of the dose-response curves using the Cheng-Prusoff equation,[7]

$$K_i = \frac{IC_{50}}{\left(1 + \frac{[S]}{K_m}\right)}$$

where K_m is the Michaelis constant: $K_{m\ (hnNOS)} = 1.6\ \mu M$; $K_{m\ (rnNOS)} = 1.3\ \mu M$; $K_{m\ (hiNOS)} = 8\ \mu M$; $K_{m\ (heNOS)} = 3.9\ \mu M$.[8] Dose−response curves were constructed from ten to eleven test concentrations (10 mM to 10 nM), and IC_{50} values were calculated by nonlinear regression using GraphPad Prism software. The calculated standard deviations from dose−response curves of the assays were less than 10% with all NOSs.

2. PAMPA-BBB Assay Protocol

Blood-brain barrier penetration was estimated using the PAMPA−BBB assay, in which a porcine brain lipid (PBL) was used as an artificial membrane.[9-11] The five commercial drugs, (±)-verapamil, desipramine, chlorpromazine, dopamine, and theophylline; phosphate buffer saline (PBS, 10 mM);

DMSO (for biology); and dodecane (analytical standard) were purchased from Sigma-Aldrich. The porcine brain lipid (PBL) was obtained from Avanti Polar Lipids (100 mg, powder, catalogue no. 141101P). The donor plate used in the assay is a 96-well filter plate with a hydrophobic polyvinylidene fluoride (PVDF) membrane (pore size 0.45 μm, nonsterile, catalogue no. MAIPNTR10), and the acceptor plate is a 96-well transport receiver plate (catalogue no. MATRNPS50), both from Millipore Sigma. A 96-well UV plate with a flat bottom obtained from Greiner Bio- One was used for UV measurements (catalogue no. 655801). Test compounds were first dissolved in DMSO to make a 10 mM stock solution. Then 40 μL of the stock solution was diluted with 1960 μL of 10 mM PBS buffer (pH 7.5) to generate a final concentration of 200 μM (2% DMSO). The acceptor plate was filled with 250 μL of 10 mM PBS (2% DMSO, pH 7.5). The donor plate was first coated with 4 μL of PBL (20 mg/mL in dodecane), then 250 μL of a test compound (200 μM) was added to the donor plate. Each test compound was measured in triplicate. The donor plate was then carefully placed on top of the acceptor plate to make a "sandwich", which was incubated at 25 °C for 17 h in a saturated humidity atmosphere with an orbital agitation at 100 rpm. During this time, compounds diffuse from the donor plate to the acceptor plate. After incubation, 150 μL of test solution was taken from each well from both donor and acceptor plates and transferred to the UV plate for measurement. The concentration of a compound in each donor and acceptor well was determined by using a standard curve, which was built from its UV absorbance at λmax of various concentrations (1−200 μM). The effective permeability (P_e) was calculated using the following equation[12],

$$P_e = \frac{2.303}{A\bullet(t-\tau_{ss})} \bullet \frac{V_A \cdot V_D}{(V_A+V_D)} \quad \lg\left[1-\left(\frac{(V_A+V_D)}{(1-R)\cdot V_D}\right)\bullet\left(\frac{C_A(t)}{C_D(0)}\right)\right]$$

where P_e is the effective permeability (cm s−1); VA and VD are the volume of the acceptor and donor wells (0.25 cm^3), respectively; CA (t) is the concentration of the acceptor well at time t; CD(0) and CD(t) are the concentrations of the donor well at t0 and t, respectively; A is the filter well area (0.21 cm2); t is the incubation time (s); τss is the time to reach a S4 steady state (usually very short compared with the incubation time); and R is the retention membrane factor, which was calculated using the following equation:

$$R = \left[1 - \frac{C_D(t)}{C_D(0)} - \frac{V_A}{V_D} \cdot \frac{C_A(t)}{C_D(0)}\right]$$

P_e is reported as an average of triplicates with a standard deviation.

3. Preparation of NOS heme domain samples for crystallography

Preparation of the heme domains of rnNOS, hnNOS, and heNOS for crystallography by trypsin digestion of full-length proteins was carried out as described previously.[4-5] All heNOS heme domain protein used in structural studies was prepared in this way. For rnNOS and hnNOS, in addition to heme domain samples obtained from trypsin-digested full-length proteins, the heme domains alone of rnNOS and hnNOS were subcloned and S5purified as described below and used in some structures.

To generate the expression construct for the rat nNOS heme domain, amino acids Arg299 to Trp716 of rat nNOS were amplified from the rnNOS expression construct, pCWorirnNOS,[4] which includes an R349A mutation. A thrombin-cleavage site was engineered into the reverse primer to create a thrombin-cleavable C-terminal His6 tag. The resulting PCR product was cloned into the NdeI and XhoI sites of pET22b. Similarly, amino acids Cys302 to Lys722 from human nNOS were cloned into pET22b by amplification of this region from the construct pCWori-hnNOS HD (which includes both PDZ and heme domains),[5] which also contains R354A and G356D mutations. The PCR product was then cloned into the NdeI and XhoI sites of pET22b.

The design of primers was reported previously.[13] Expression of the rnNOS and hnNOS heme domain constructs was carried out in BL21/DE3 cells. Cell growth and Ni column purification were described in detail previously.[13] Peak fractions from the Ni column eluate were pooled and concentrated using an AmiconUltra centrifugal filter unit (Millipore, 30 kD cutoff). One round of buffer exchange was carried out using 150 mM NaCl, 20 mM Tris, 10% glycerol pH 8.5, and the sample was concentrated to ⩽3 mL. This sample was digested with thrombin (MP Biomedicals) using a ratio of 30 U thrombin per mg of protein and incubated overnight at 4 °C. The thrombin-digested sample was passed over a Ni column in Ni column buffer (as above except also including

0.25 mM PMSF and 20 mM imidazole) to remove uncleaved protein and His tags. The flow-through was collected and concentrated to ≤1.5 mL. The sample was then loaded onto an S-200 column and run as reported previously.[13] Fractions from the S-200 column were pooled based on spectral ratio (280 nm/405 nm) and concentrated to > 8 mg/mL for crystallization.

To study the new Zn-binding site observed in many rnNOS structures,[14] we mutated one of the Zn binding ligands, His692, to a Phe to disrupt Zn binding. This mutation was engineered into the full-length rnNOS expression construct by site-directed mutagenesis using PrimerSTAR Max DNA polymerase (Takara). The full-length rnNOS H692F was S6 expressed and purified as previously described for wild-type nNOS[4] which was then used to generate the heme domain by trypsin digestion as previously described for wild-type nNOS.[4] In this study, this heme domain sample of rnNOS H692F was used only in one structure, rnNOS H692F-18 (Table S1). Because of the large distance between His692 and the active site of rnNOS, the mutant was expected to behave like the wild-type protein with regard to inhibitor binding.

4. Inhibitor Complex Crystal Preparation

The cloning, expression, and purification for the heme domain of rnNOS and hnNOS was reported previously.[13] The heme domain of heNOS for crystallography was generated by trypsin digestion of full-length proteins as described.[5] The sitting drop vapor diffusion method was used to grow crystals at 4 °C for the heme domains of rnNOS R349A (8 mg/mL containing 20 mM histidine), hnNOS R354A/G357D (10 mg/mL), and wild-type heNOS (10 mg/mL). The crystal growth conditions were as described previously.[4-5] Seeding techniques were applied for all cases often at a reduced protein concentration to improve the size and quality of crystals. As described previously,[4] the final step of cryo-soakings for both rnNOS and hnNOS crystals were carried out in HEPES buffer (pH 7.5); for heNOS, Bis−Tris buffer at pH 7.5 was used. Crystals were further soaked with 5−10 mM inhibitor for 2−4 h at 4 °C before being flash cooled with liquid nitrogen and stored until data collection. For a few inhibitors in which the soaking of chemicals into the crystal lattice did not produce clear density for the inhibitors, co-crystallization was used, that is, 2 mM of inhibitor was included in the crystallization buffer. The resulting density for inhibitors was much better.

5. X-ray Diffraction Data Collection, Data Processing, and Structural Refinement

The cryogenic (100 K) X-ray diffraction data were collected remotely at the Stanford Synchrotron Radiation Lightsource (SSRL) or the Advanced Light Source (ALS) through data collection control software Blu-Ice[15] and a crystal-mounting robot. Although the crystal growth conditions were not changed, the rnNOS and hnNOS crystals produced with the cloned heme domain proteins showed different symmetry from crystals produced from the heme domain proteins generated by trypsin cleavage of their respective full-length NOS.[4,5] The rnNOS crystals changed from $P2_12_12_1$ space group[4] to $C222_1$, with only one subunit per asymmetric unit. The hnNOS crystals changed from $P2_12_12_1$ to $P2_1$ space group, even though the β angle showed almost no deviation from 90.0° with two dimers in the asymmetric unit.[16] Owing to the random choices of the asymmetric unit by the program the cell dimensions vary by the rearrangement of the three cell edges. In this study, all the rnNOS and hnNOS structures were determined using the subcloned and expressed heme domain protein. All the heNOS crystals were in the $P2_1$ space group rather than orthorhombic $P2_12_12_1$ reported previously,[5] with a β angle less than 1° off 90°. Therefore, a molecular replacement calculation with PHASER-MR[17] was needed initially to solve the structure. In the $P2_1$ space group, there are two heNOS dimers in the asymmetric unit.

Whenever duplicate data sets were available, merging and scaling the two together was attempted. For the fine-sliced pixel array data the higher redundancy of merged data from two crystals often led to better scaling statistics and were thus used in the following structure refinements. The number of crystals used for the refinement of each structure are listed in Table S1. Raw CCD data frames were indexed, integrated, and scaled using iMOSFLM,[18] but the pixel array data were preferably processed with XDS[19] and scaled with Aimless.[20] The binding of inhibitors was detected by initial difference Fourier maps calculated with REFMAC.[21] The inhibitor molecules were then modeled in Coot[22] and refined using REFMAC and then PHENIX.[23] Disordering in portions of inhibitors bound in the NOS active sites was often observed, sometimes resulting in poor density quality. However, partial structural features were usually still visible if the contour level of the sigma A weighted $2m|Fo| - D|Fc|$ map was dropped to 0.5σ, which enabled the building of reasonable models into disordered regions. Water molecules were added in PHENIX and checked visually in Coot. The TLS[24] protocol was implemented in the PHENIX refinements with each subunit as one TLS group. The omit $Fo - Fc$ density maps were calculated by the Polder map

routine in PHENIX by removing the bound inhibitors in the map calculation.[25] The refined structures were validated through the validation facility in wwPDB before the final deposition to the Protein Data Bank. The crystallographic data collection and refinement statistics are reported in Table S1, which includes the PDB codes for all structures in this study.

6. Table S1. Crystallographic data collection and refinement statistics

Data set[a]	hnNOS R354A G357D-**HH044**	hnNOS R354A G357D-**1**	hnNOS R354A G357D-**9**
**Data collection**			
PDB code	9MWA	9MWB	9MWC
Space group	$P2_1$	$P2_1$	$P2_1$
Cell dimensions			
a, *b*, *c* (Å)	52.3 118.7 165.5	118.5 52.3 165.1	53.3 118.1 165.2
β (º)	90.19	90.00	90.15
Resolution (Å)	2.00 (2.03-2.00)	1.99 (2.03-1.99)	2.50 (2.56-2.50)
R_{merge}	0.331 (1.552)	0.267 (4.713)	0.136 (1.431)
R_{pim}	0.133 (0.976)	0.140 (2.549)	0.069 (0.776)
$R_{measure}$	0.357 (1.840)	0.302 (5.380)	0.153 (1.636)
CC 1/2	0.988 (0.488)	0.997 (0.557)	0.994 (0.426)
$< I / \sigma I >$	5.4 (1.57) [c]	5.1 (1.05) [c]	8.3 (2.22) [c]
No. unique reflections	145729 (6664)	138029 (5998)	68781 (4529)
Completeness (%)	99.4 (98.7)	99.4 (88.4)	99.0 (98.0)
Redundancy	6.2 (3.5)	4.5 (4.0)	4.8 (4.4)
No. crystals	1	1	1
Wilson B factor (Å)	26.5	30.4	48.8
**Refinement**			
Resolution (Å)	2.00	1.99	2.50
No. reflections used	135291	137739	68054
R_{work} / R_{free}^{b}	0.185/0.230	0.201/0.254	0.175/0.249
No. atoms, Protein	13579	13653	13607
Ligand/ion	397	406	417
Water	886	957	347
R.m.s. deviations			
Bond lengths (Å)	0.007	0.007	0.008
Bond angles (º)	1.02	1.00	1.02

Data set[a]	rnNOS R349A-**1**	rnNOS R349A-**2**	rnNOS R349A-**3**	rnNOS R349A-**5**
**Data collection**				
PDB code	9MWD	9MWE	9MWF	9MWG
Space group	$C222_1$	$C222_1$	$C222_1$	$C222_1$
Cell dimensions				
a, *b*, *c* (Å)	48.8 113.7 162.8	49.0 114.5 164.6	48.8.114.2 164.6	48.1 113.6 163.0
Resolution (Å)	1.94 (1.99-1.94)	2.04 (2.10-2.04)	2.07 (2.13-2.07)	1.89 (1.93-1.89)
R_{merge}	0.051 (4.461)	0.243 (6.363)	0.196 (6.466)	0.152 (8.274)
R_{pim}	0.024 (2.224)	0.059 (1.602)	0.061 (2.131)	0.044 (2.555)
R_{measure}	0.056 (5.008)	0.250 (6.565)	0.205 (6.825)	0.158 (9.327)
CC 1/2	0.999 (0.359)	0.998 (0.594)	0.998 (0.453)	0.999 (0.382)
$< I / \sigma I >$	14.1 (1.16) [c]	8.8 (1.45) [c]	6.8 (1.36) [c]	8.8 (1.26) [c]
No. unique reflections	33554 (2052)	30055 (2280)	28486 (2170)	36345 (2338)
Completeness (%)	98.7 (92.2)	100.0 (99.8)	99.8 (99.8)	100.0 (100.0)
Redundancy	5.6 (4.6)	18.4 (16.8)	11.0 (9.7)	13.2 (13.2)
No. crystals	1	3	2	2
Wilson B factor (Å)	49.9	40.6	44.4	41.2
**Refinement**				
Resolution (Å)	1.94	2.04	2.07	1.89
No. reflections used	32839	29335	27530	35565
R_{work} / $R_{\mathrm{free}}{}^{b}$	0.207/0.259	0.207/0.263	0.215/0.275	0.204/0.243
No. atoms, Protein	3344	3324	3340	3344
Ligand/ion	88	108	108	88
Water	68	57	29	73
R.m.s. deviations				
Bond lengths (Å)	0.008	0.006	0.008	0.008
Bond angles (º)	1.32	0.96	1.13	1.06G

Data set[a]	rnNOS R349A-**6**	rnNOS R349A-**7**	rnNOS R349A-**8**
**Data collection**			
PDB code	9MWH	9MWI	9MWJ
Space group	$C222_1$	$C222_1$	$C222_1$
Cell dimensions			
a, *b*, *c* (Å)	48.6 114.8 164.8	48.6 114.8 164.8	49.0 114.0 164.5
Resolution (Å)	1.96 (2.00-1.96)	2.05 (2.10-2.05)	1.98 (2.03-1.98)
R_{merge}	0.125 (4.312)	0.112 (5.801)	0.065 (7.290)
R_{pim}	0.038 (1.769)	0.050 (2.524)	0.025 (2.978)
$R_{measure}$	0.131 (4.672)	0.123 (6.343)	0.070 (7.896)
CC 1/2	0.998 (0.584)	0.989 (0.334)	0.999 (0.322)
$< I / \sigma I >$	9.1 (1.21) [c]	6.6 (0.91) [c]	14.8 (1.31) [c]
No. unique reflections	33737 (2219)	29132 (2230)	32276 (2194)
Completeness (%)	99.6 (94.7)	98.8 (98.0)	99.0 (96.9)
Redundancy	11.7 (6.5)	6.0 (6.0)	7.4 (6.8)
No. crystals	2	1	1
Wilson B factor (Å)	44.8	45.0	49.1
**Refinement**			
Resolution (Å)	1.96	2.05	1.98
No. reflections used	32941	27156	29976
R_{work} / R_{free}[b]	0.206/0.254	0.207/0.271	0.223/0.279
No. atoms, Protein	3348	3385	3337
Ligand/ion	98	98	98
Water	41	50	38
R.m.s. deviations			
Bond lengths (Å)	0.008	0.009	0.008
Bond angles (º)	1.15	1.17	1.05

Data set[a]	rnNOS R349A-**9**	rnNOS R349A-**10**	rnNOS R349A-**11**
**Data collection**			
PDB code	9MWK	9MWL	9MWM
Space group	$C222_1$	$C222_1$	$C222_1$
Cell dimensions			
a, *b*, *c* (Å)	48.5 114.9 164.9	48.4 113.8 162.9	48.2 113.8 163.1
Resolution (Å)	2.00 (2.05-2.00)	1.87 (1.91-1.87)	1.95 (2.00-1.95)
R_{merge}	0.141 (5.790)	0.061 (4.525)	0.077 (3.525)
R_{pim}	0.043 (2.538)	0.021 (1.532)	0.033 (1.434)
$R_{measure}$	0.148 (6.337)	0.065 (4.782)	0.084 (3.810)
CC 1/2	0.997 (0.443)	1.000 (0.348)	0.998 (0.453)
$< I / \sigma I >$	8.3 (1.12)[c]	16.3 (1.11)[c]	13.1 (1.35)[c]
No. unique reflections	31791 (2256)	32337 (2225)	32977 (2376)
Completeness (%)	99.7 (97.1)	99.9 (99.9)	99.4 (99.4)
Redundancy	11.2 (6.1)	9.4 (9.6)	6.4 (6.9)
No. crystals	2	1	1
Wilson B factor (Å)	50.3	46.8	50.0
**Refinement**			
Resolution (Å)	2.00	1.87	1.95
No. reflections used	30818	37152	32414
R_{work} / $R_{\mathrm{free}}{}^{b}$	0.207/0.255	0.203/0.237	0.198/0.244
No. atoms, Protein	3344	3338	3338
Ligand/ion	98	98	98
Water	42	89	70
R.m.s. deviations			
Bond lengths (Å)	0.007	0.006	0.007
Bond angles (º)	1.03	0.93	0.99

Data set[a]	heNOS-**HH044**	heNOS-**1**	heNOS-**2**	heNOS-**3**
**Data collection**				
PDB code	9MWN	9MWO	9MWP	9MWQ
Space group	$P2_1$	$P2_1$	$P2_12_12_1$	$P2_1$
Cell dimensions				
a, *b*, *c* (Å)	59.6 151.7 108.3	59.6 153.2 108.9	61.6 109.4 152.7	59.7 152.9 108.8
β (º)	90.9	90.9		90.8
Resolution (Å)	2.00 (2.03-2.00)	1.76 (1.79-1.76)	1.86 (1.89-1.86)	1.94 (1.97-1.94)
R_{merge}	0.155 (1.111)	0.072 (1.748)	0.122 (2.614)	0.192 (2.320)
R_{pim}	0.051 (0.434)	0.037 (0.990)	0.043 (1.206)	0.068 (0.876)
$R_{measure}$	0.163 (1.176)	0.081 (2.023)	0.130 (2.910)	0.204 (2.485)
CC 1/2	0.997 (0.581)	0.998 (0.360)	0.997 (0.359)	0.993 (0.490)
$< I / \sigma I >$	10.3 (1.57)[c]	10.9 (1.11)[c]	9.7 (1.09)[c]	5.9 (1.08)[c]
No. unique reflections	129489 (6356)	189993 (8940)	85985 (3842)	104103 (5172)
Completeness (%)	99.9 (99.6)	98.6 (94.3)	98.3 (83.9)	99.7 (93.5)
Redundancy	10.2 (7.5)	4.6 (3.9)	9.0 (5.5)	8.6 (7.6)
No. crystals	1	1	1	1
Wilson B factor (Å)	26.5	30.4	30.2	29.8

**Refinement**				
Resolution (Å)	2.00	1.76	1.86	1.94
No. reflections used	129311	187554	83777	143477
R_{work} / $R_{free}{}^{b}$	0.192/0.235	0.202/0.233	0.179/0.214	0.172/0.211
No. atoms, Protein	12973	12920	6487	13200
Ligand/ion	388	555	318	443
Water	839	728	730	903
R.m.s. deviations				
Bond lengths (Å)	0.008	0.006	0.009	0.009
Bond angles (º)	1.10	0.97	1.04	1.00

Data set[a]	heNOS-**5**	heNOS-**6**	heNOS-**7**	heNOS-**8**
**Data collection**				
PDB code	9MWR	9MWX	9MWS	9MWT
Space group	$P2_1$	$P2_1$	$P2_1$	$P2_1$
Cell dimensions				
a, *b*, *c* (Å)	58.8 152.1 107.6	59.5 153.0 108.5	59.5 153.3 108.7	59.7 153.0 108.0
β (º)	90.8	90.6	90.6	90.8
Resolution (Å)	1.97 (2.00-1.97)	1.84 (1.87-1.84)	1.90 (1.93-1.90)	1.90 (1.93-1.90)
R_{merge}	0.094 (0.402)	0.163 (1.643)	0.155 (1.238)	0.093 (1.964)
R_{pim}	0.046 (0.203)	0.062 (0.844)	0.054 (0.655)	0.048 (1.059)
$R_{measure}$	0.104 (0.452)	0.174 (1.854)	0.164 (1.407)	0.105 (2.241)
CC 1/2	0.995 (0.902)	0.992 (0.592)	0.996 (0.371)	0.998 (0.446)
$< I / \sigma I >$	8.4 (1.73)[c]	6.7 (1.60)[c]	8.7 (1.60)[c]	10.1 (1.36)[c]
No. unique reflections	127972 (5658)	164549 (6374)	152530 (7552)	151545 (7505)
Completeness (%)	96.3 (90.2)	98.8 (97.2)	99.7 (96.1)	98.9 (98.8)
Redundancy	5.1 (4.7)	7.3 (4.6)	8.9 (4.7)	4.6 (4.3)
No. crystals	1	1	1	1
Wilson B factor (Å)	28.1	29.1	25.6	32.2
**Refinement**				
Resolution (Å)	1.97	1.84	1.90	1.90
No. reflections used	127908	164457	151205	150000
R_{work} / R_{free}^{b}	0.172/0.211	0.198/0.234	0.170/0.203	0.192/0.233
No. atoms, Protein	12829	12884	12912	12871
Ligand/ion	504	527	535	541
Water	933	860	1101	830
R.m.s. deviations				
Bond lengths (Å)	0.009	0.010	0.009	0.009
Bond angles (º)	1.15	1.09	1.09	0.98

Data set[a]	heNOS-**9**	heNOS-**10**	heNOS-**11**
**Data collection**			
PDB code	9MWU	9MWV	9MWW
Space group	$P2_12_12_1$	$P2_1$	$P2_1$
Cell dimensions			
a, *b*, *c* (Å)	61.7 109.3 152.1	59.8 152.7 108.8	59.4 152.6 108.8
β (º)		90.9	90.04
Resolution (Å)	1.74 (1.77-1.74)	1.90 (1.93-1.90)	1.80 (1.83-1.80)
R_{merge}	0.097 (1.407)	0.116 (1.568)	0.112 (2.589)
R_{pim}	0.035 (1.037)	0.071 (1.013)	0.056 (1.284)
$R_{measure}$	0.103 (1.763)	0.136 (1.875)	0.126 (2.897)
CC 1/2	0.998 (0.249)	0.993 (0.347)	0.997 (0.430)
$< I / \sigma I >$	12.6 (1.11)[c]	5.5 (1.17)[c]	7.1 (1.34)[c]
No. unique reflections	102817 (2754)	147478 (7174)	177075 (8726)
Completeness (%)	96.3 (53.4)	96.3 (83.9)	99.2 (99.5)
Redundancy	8.1 (2.1)	3.6 (3.2)	4.9 (4.9)
No. crystals	1	1	1
Wilson B factor (Å)	23.5	32.5	29.1
**Refinement**			
Resolution (Å)	1.74	1.90	1.90
No. reflections used	102623	146882	176706
R_{work} / R_{free}^b	0.156/0.182	0.181/0.219	0.194/0.231
No. atoms, Protein	6467	12842	12834
Ligand/ion	299	554	574
Water	881	785	801
R.m.s. deviations			
Bond lengths (Å)	0.009	0.009	0.009
Bond angles (º)	1.03	0.99	1.01

[a] See Figure 3 for nomenclature and chemical formula of inhibitors.

[b] R_{free} was calculated with the 5% of reflections set aside throughout the refinement. The set of reflections for the R_{free} calculation were kept the same for all data sets according to those used in the data of the starting model (7TS9 for rnNOS in $C222_1$, 7TS7 for hnNOS in $P2_1$, 4D10 for heNOS in $P2_12_12_1$, and 5UO8 for heNOS in $P2_1$).

[c] The $< I / \sigma I >$ value in the highest resolution shell calculated by Xtriage and reported in the wwPDB X-ray Structure Validation Report.

7. Compounds 2, 3, 5, 6, and 7 with Figures S1, S2, S3, and S4

Changing from a 2-substituted furan in **1** to a 3-substituted furan in **2** also resulted in an improvement in both potency and selectivity (Table 1). It is difficult to assess from the experimental structures why the 3-substituted furan in **2** (**Figure S1**) binds more tightly than the 2-substituted furan in **1** (**Figure 6**), as the furan ring in both **1** and **2** have poorly defined electron density.

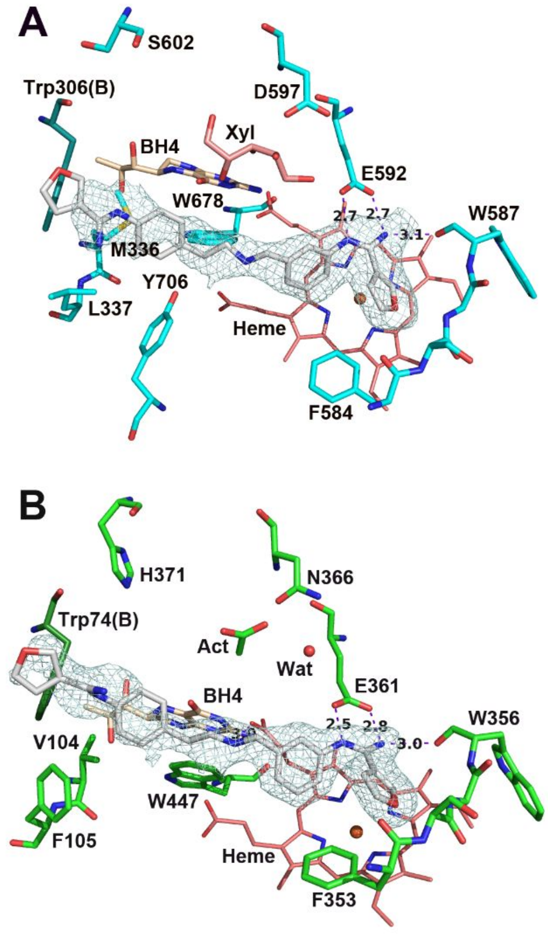

Figure S1. Compound **2** bound to (A) rnNOS (PDB code: 9MWE) and (B) heNOS (PDB code: 9MWP).

Figure S2A shows the binding of **3** to rnNOS. The tail oxazole ring of **3** is roughly perpendicular to Trp306 (chain B) which is similar to the position found for **2** in rnNOS (**Figure S1A)**. The two compounds, **2** and **3**, show a sharp contrast in potency and selectivity. This implies that it is the chemical nature of the 5-membered ring, furan or oxazole, rather than the binding position that determines the potency of each inhibitor. It is worth noting that the position of the tail 5-membered ring of **2** and **3** is quite different from the one observed for most other double headed compounds in this series. Both structures were obtained by co-crystallization of rnNOS in the presence of the inhibitor in the buffer. There is a xylitol molecule near the inhibitor in both structures which is the break down product of mannitol used in the cryo soaking of crystals. Possibly this extra sugar hinders the tail 5-membered from adopting the preferred position by the other inhibitors. Binding of **3** to heNOS is nothing unusual (**Figure S2B**). The tail oxazole ring of **3** makes van der Waals contact with Trp74 (Chain B) using its carbon side of the ring to avoid the non-favorite contact from its polar nitrogen atom.

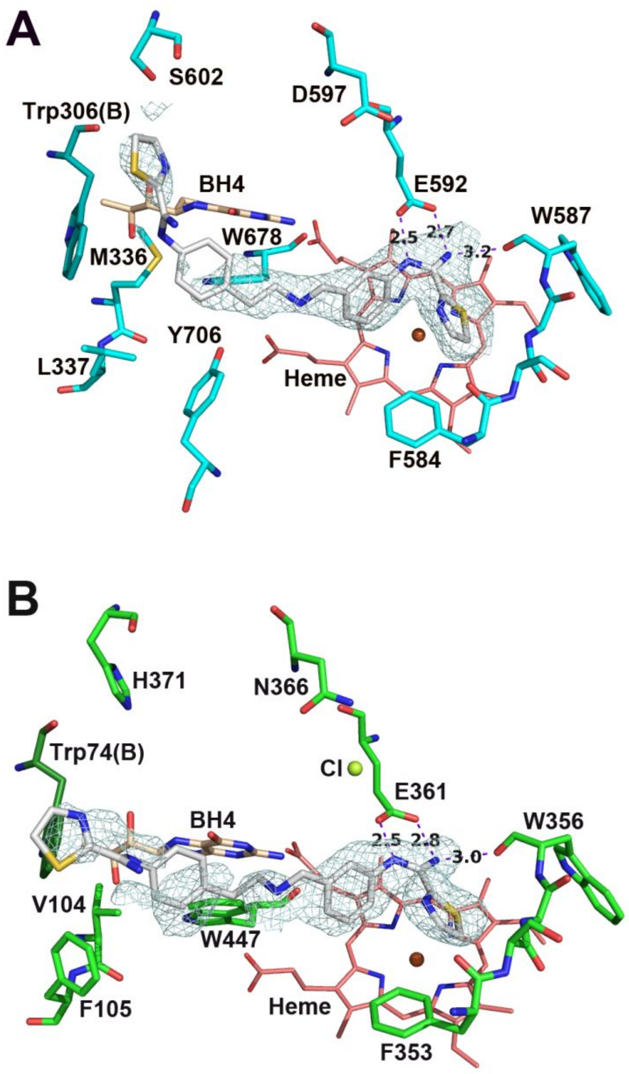

Figure S2. Compound **3** bound to (A) rnNOS (PDB code: 9MWF) and (B) heNOS (PDB code: 9MWQ).

In rnNOS all three thiazole bearing compounds, the 5-thiazole substituted **5** and the 2-thiazole substituted **6** and **7,** have their tail thiazole ring fit into the pocket capped by Ser602, as shown in **Figure S3**. Both **6** and **7** can make electrostatic interactions from the ring nitrogen atom to the hydroxyl group of the Ser602 side chain, but **5** lacks this interaction because its carbon atoms face Ser602. This explains why **5** is less potent than **6** or **7** in rnNOS.

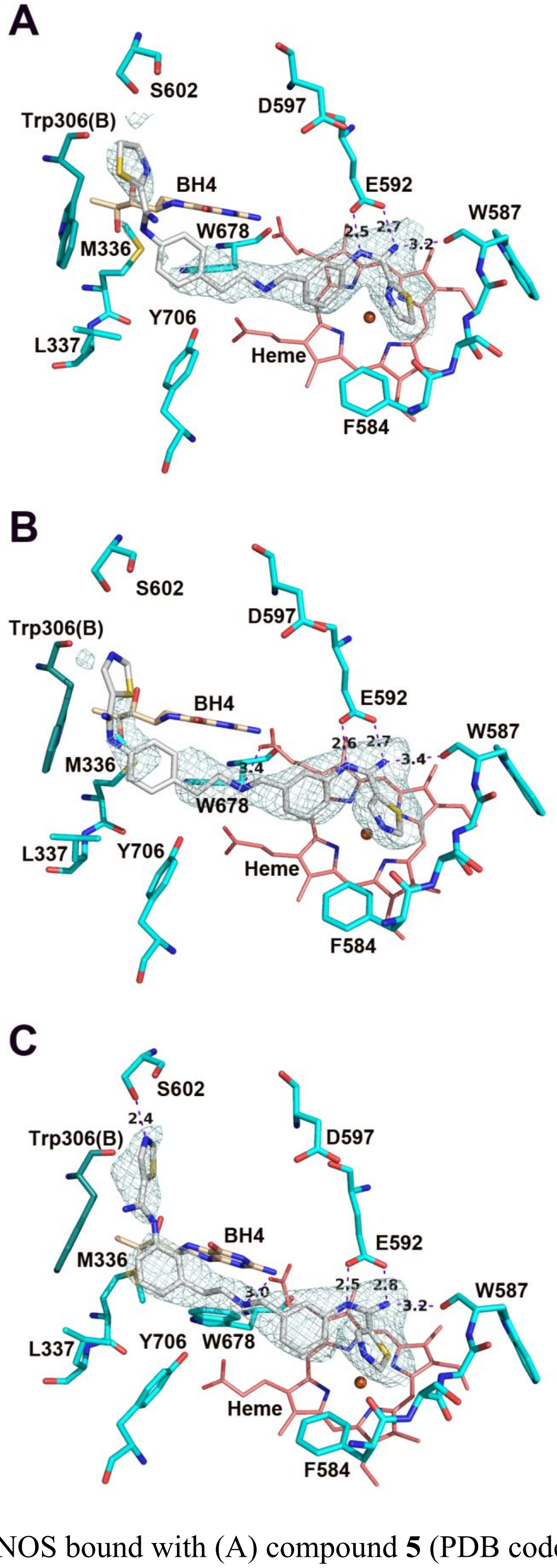

Figure S3. Structure of rnNOS bound with (A) compound **5** (PDB code: 9MWG), (B) compound **6** (PDB code: 9MWH), and (C) compound **7** (PDB code: 9MWI).

In heNOS the tail thiazole rings of all three compounds, **5**, **6**, and **7**, are in van der Waals contacts with both Phe105 and Trp74 (chain B), as shown in **Figure S4**. Because of the different heteroatom positions on the thiazole ring when approaching the two hydrophobic protein residues, it is more difficult for **5** to avoid the clashing with its polar atoms on the ring than **6** or **7**. This may account for the much poorer potency of **5** with heNOS.

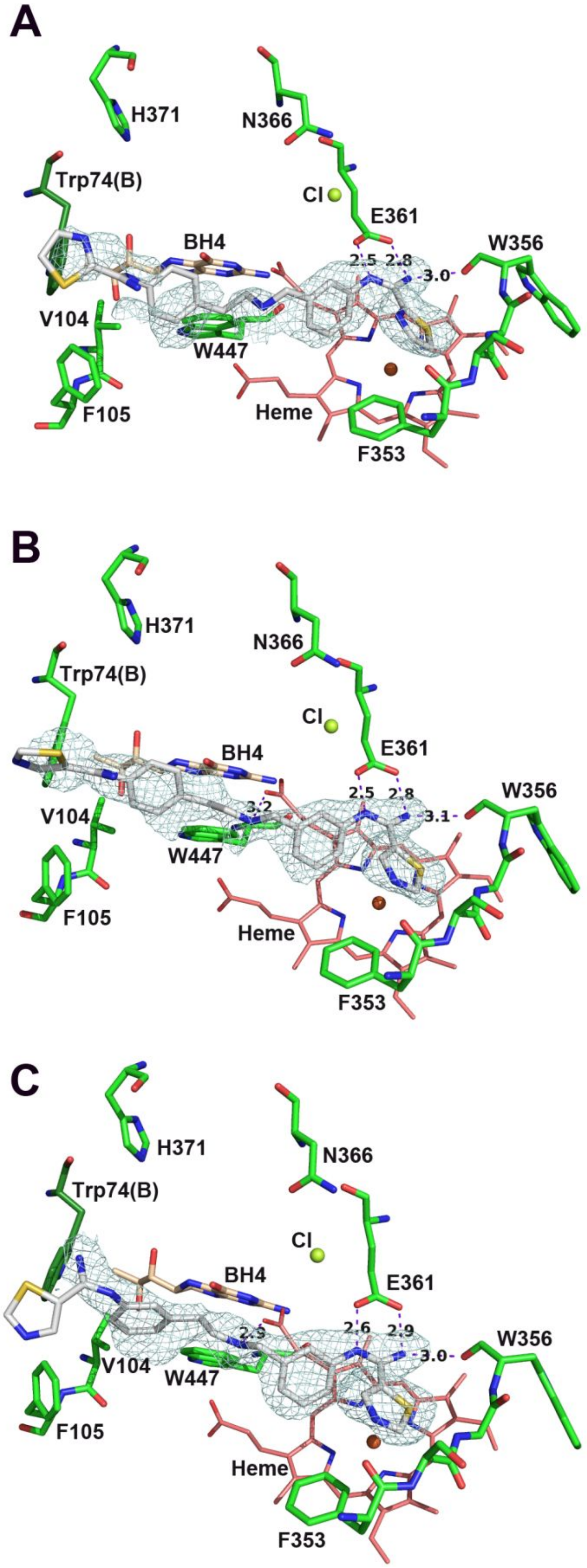

Figure S4. Structure of heNOS bound with (A) compound **5** (PDB code: 9MWR), (B) compound **6**(PDB code: 9MWX), and (C) compound **7**(PDB code: 9MWS).

8. Computational details: Materials and Methods

Most computational work was performed in the Maestro as a part of the Schrödinger Release 2023-2 program (Schrödinger, LLC, New York, NY). All figures from the computational modeling were visualized using BIOVIA Discovery Studio 2024 Visualizer (Dassault Systèmes, San Diego, CA). For the molecular dynamics (MD) simulation, each crystal structure obtained from this study (PDB ID: 9MWA, 9MWN, 9MWC, and 9MWU for hnNOS-HH044, heNOS-HH044, hnNOS-9, and heNOS-9, respectively) was subjected to the Protein Preparation Wizard[26] and Prime[27] minimization. The 'Automatic' option, combining the conjugate gradient and the truncated Newton method, was used in the minimization step, and the RMS gradient for convergence was 0.01 kcal/mol/Å. All the structures were typed by the OPLS4 force field[28] during the calculation. For the ligand-metal coordination, zero-order bonds were assigned. The iron-heme nitrogen and iron-cysteine sulfur distances were ~2.1 and ~2.5 Å during simulation, respectively. The input system for the MD simulation was then generated by the System Builder incorporated into Desmond[29] from each prepared complex. The complex was solvated in the orthorhombic box of SPC water with a minimum distance of 10 Å from the complex to the box boundary, and Na^+ and Cl^- ions were added to the system, achieving a desired ionic strength of 0.15 M NaCl with neutral net charge. The prepared system was subjected to a 5-step relaxation method provided by Desmond and the production step was performed under the NPT ensemble (Nose-Hoover chain thermostat, Martyna-Tobias-Klein barostat) at 300 K for 100 ns. The MD trajectories and calculated energies were recorded every 5 ps and analyzed using the Maestro interface and Desmond module.

In the MD trajectories from the heNOS-**9** complex crystal structure, the appropriate frame showing the key tail interactions (tail amino group with BH_4 carbonyl and heme propionate) and proximity between the tail phenyl ring and the side chain amino group of Lys72(B) ($N_{Lys72(B)}$) were extracted. Then, each proton at the *para*-, *meta*-, and *ortho*-positions was replaced by fluorine using Maestro 3D builder to generate initial structures of heNOS-**13**, **15**, and **16** complex, respectively. Each structure was then subjected to the preparation for MD simulation as mentioned above. The prepared structures were used for further metadynamics simulation. A metadynamics simulation enables the enhanced sampling of protein-ligand complexes with various values of specified variables (*e.g.,* distance, angle, and/or dihedral) by recording and avoiding previously visited values using a metadynamics accumulator. The distance between the center of mass of the tail phenyl ring and the $N_{Lys72(B)}$ was specified as a collectible variable. Each metadynamics simulation

was conducted under the NPT ensemble (Nose-Hoover chain thermostat, Martyna-Tobias-Klein barostat) at 300 K and 1 atm for 5 ns. Gaussian potentials were added every 0.09 ps with a 0.03 kcal/mol height and a 0.05 Å width.

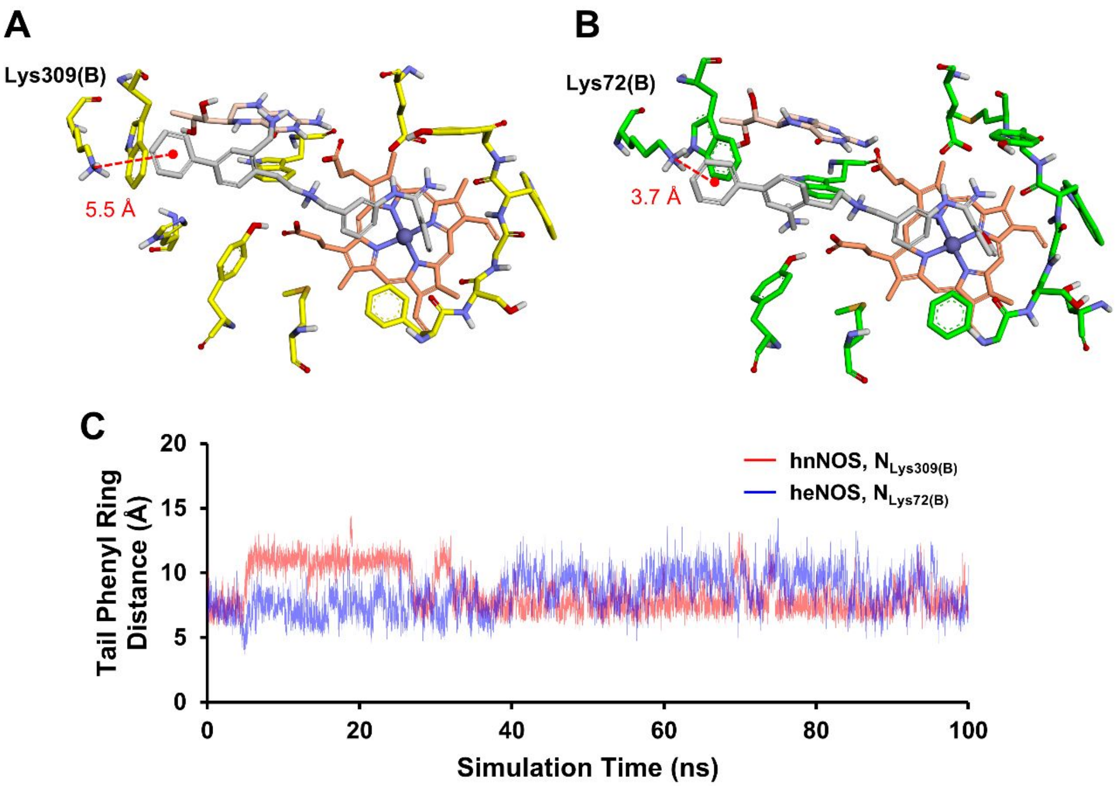

Figure S5. The binding modes of **9** from the molecular dynamics (MD) simulation trajectories in (**A**) hnNOS with the minimum tail phenyl-Lys309(B) distance and (**B**) heNOS with the minimum tail phenyl-Lys72(B) distance. Each was simulated from the crystal structures of the hnNOS-**9** and the heNOS-**9** complex. Yellow, green, orange, and gray sticks, respectively, indicate the hnNOS and heNOS binding site residues, cofactors, and **9**. (**C**) Tail phenyl ring distance to each indicated side chain amino group.

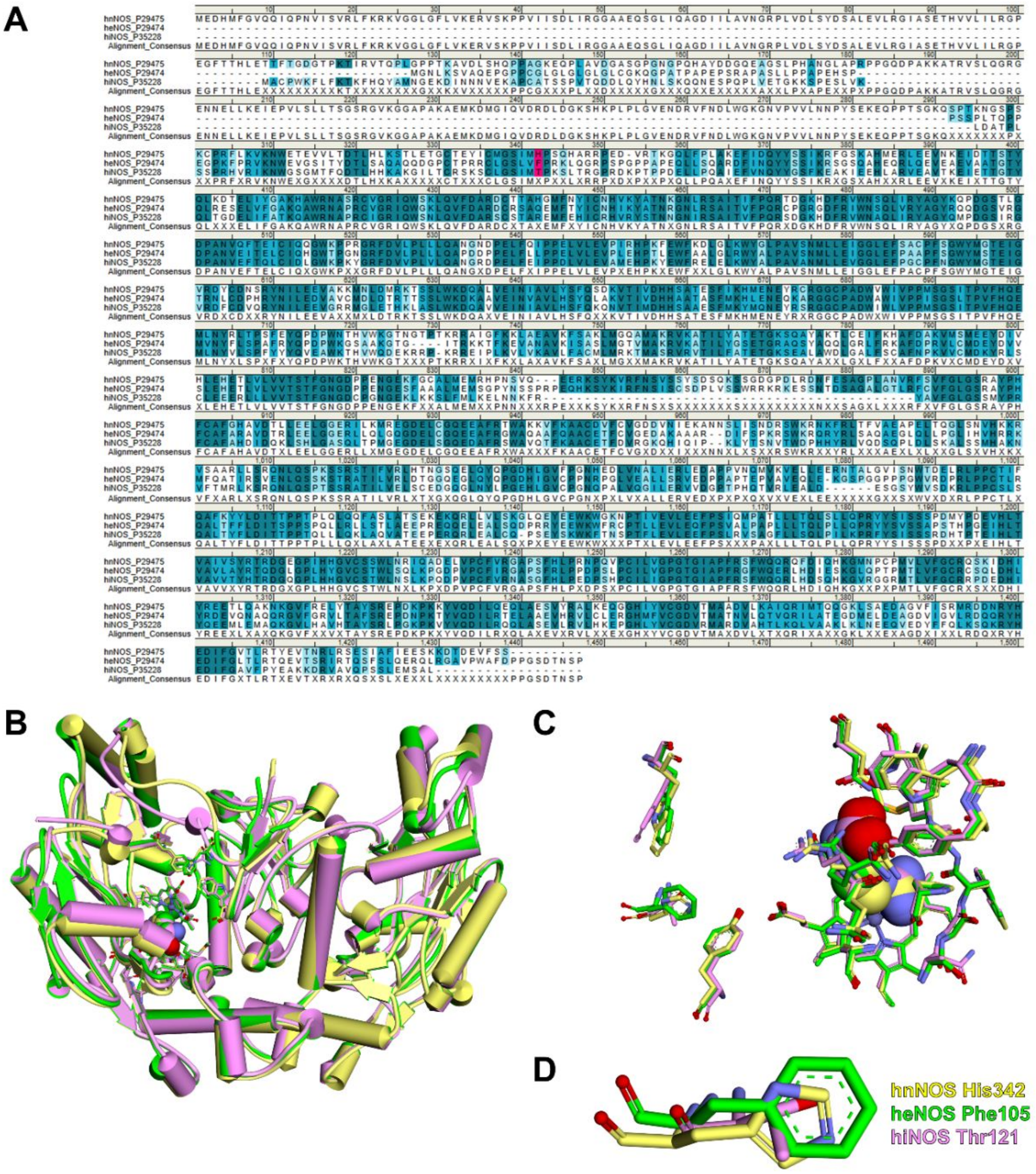

Figure S6. Sequence and structure alignments of the hnNOS, heNOS, and hiNOS. (**A**) Sequence alignment of the hnNOS, heNOS, and hiNOS. Each one-letter code was colored by the sequence similarity between three isoforms. His 342 in hnNOS, Phe105 in heNOS, and Thr121 in hiNOS, which are not conserved side chains in the active site, were shown in red. (**B**, **C**) Superimposed crystal structures of the hnNOS (yellow, PDB ID: 4D1N), heNOS (green, PDB ID: 4D1O), and hiNOS (pink, PDB ID: 1NSI) complexed with $_{\text{L}}$-arginine in (**B**) schematic representation and (**C**) stick model of binding site residues. CPK model indicates $_{\text{L}}$-arginine. (**D**) Superimposed conformations of indicated side chains in detailed view.

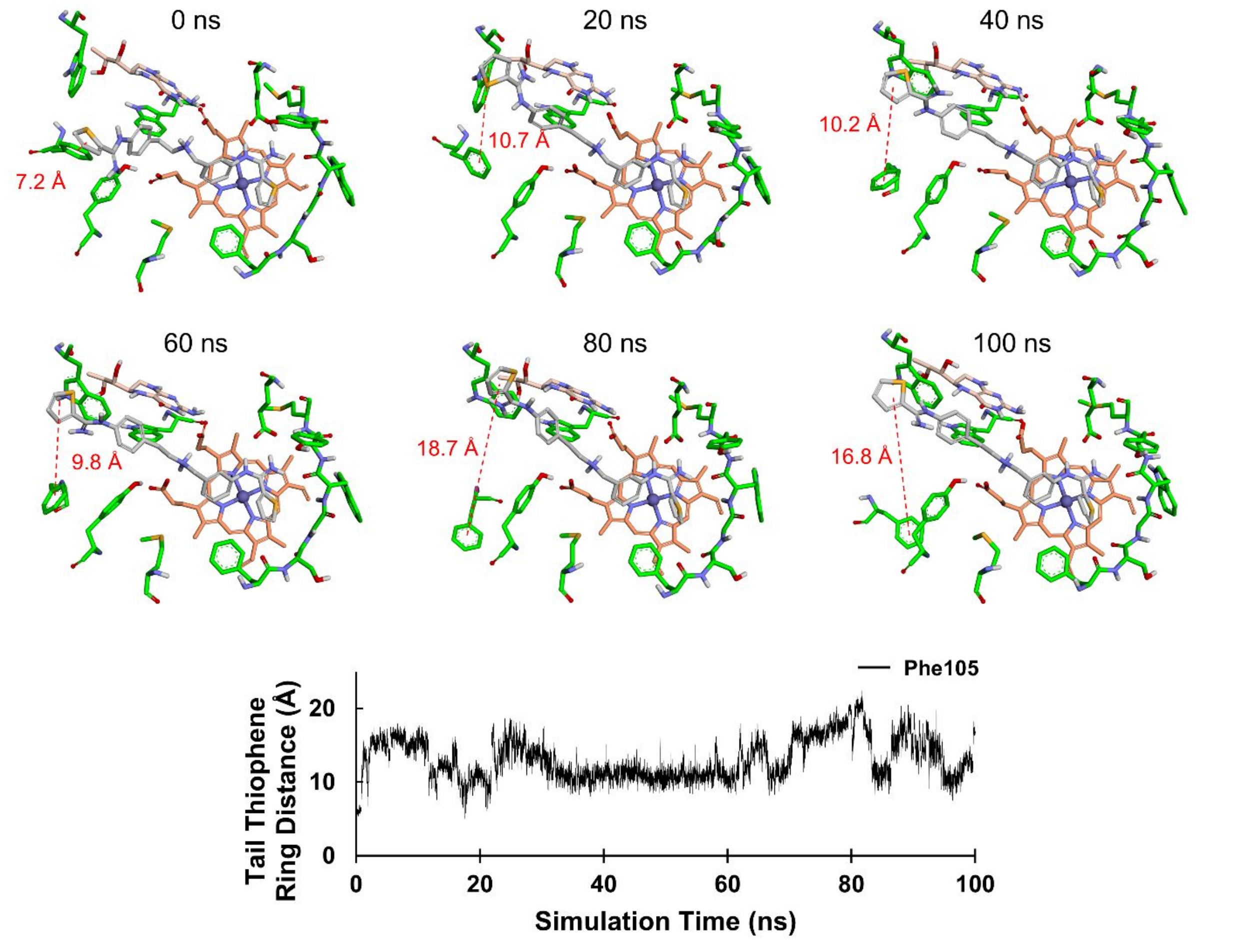

Figure S7. The binding modes of **HH044** and the tail phenyl ring distance to Phe105 side chain from the molecular dynamics (MD) simulation trajectories in heNOS after indicated simulation time. The trajectories were simulated from the crystal structures of the heNOS-**HH044**. Green, orange, and gray sticks, respectively, indicate the heNOS binding site residues, cofactors, and **HH044**.

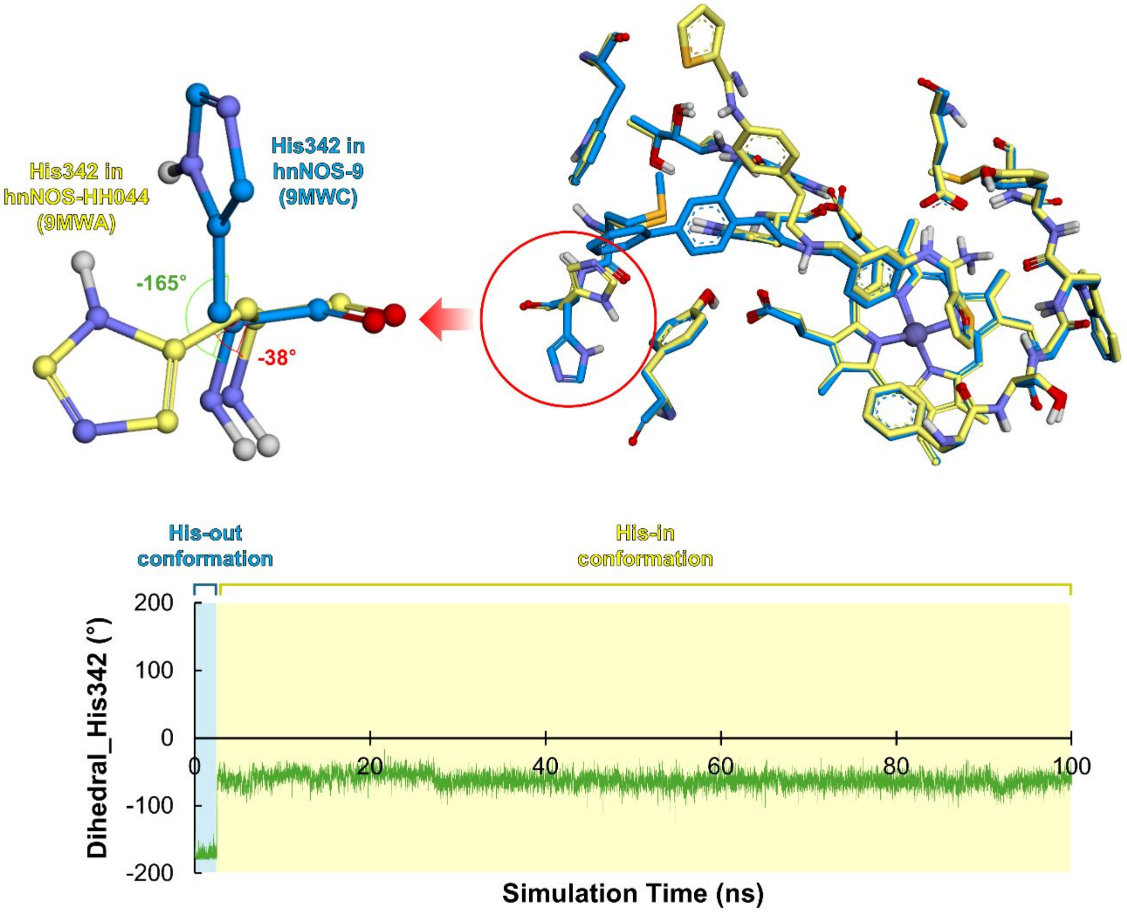

Figure S8. Two different conformations of His342 in hnNOS crystal structures. The conformation was monitored in the molecular dynamics (MD) simulation trajectories simulated from hnNOS-**9** crystal structure. The conformation was monitored by the indicated dihedral angle of His342. Yellow and blue, respectively, indicate the His-in (His342 side chain is in the binding pocket, so can interact with the ligand) and His-out (His342 side chain is out of the pocket) conformation.

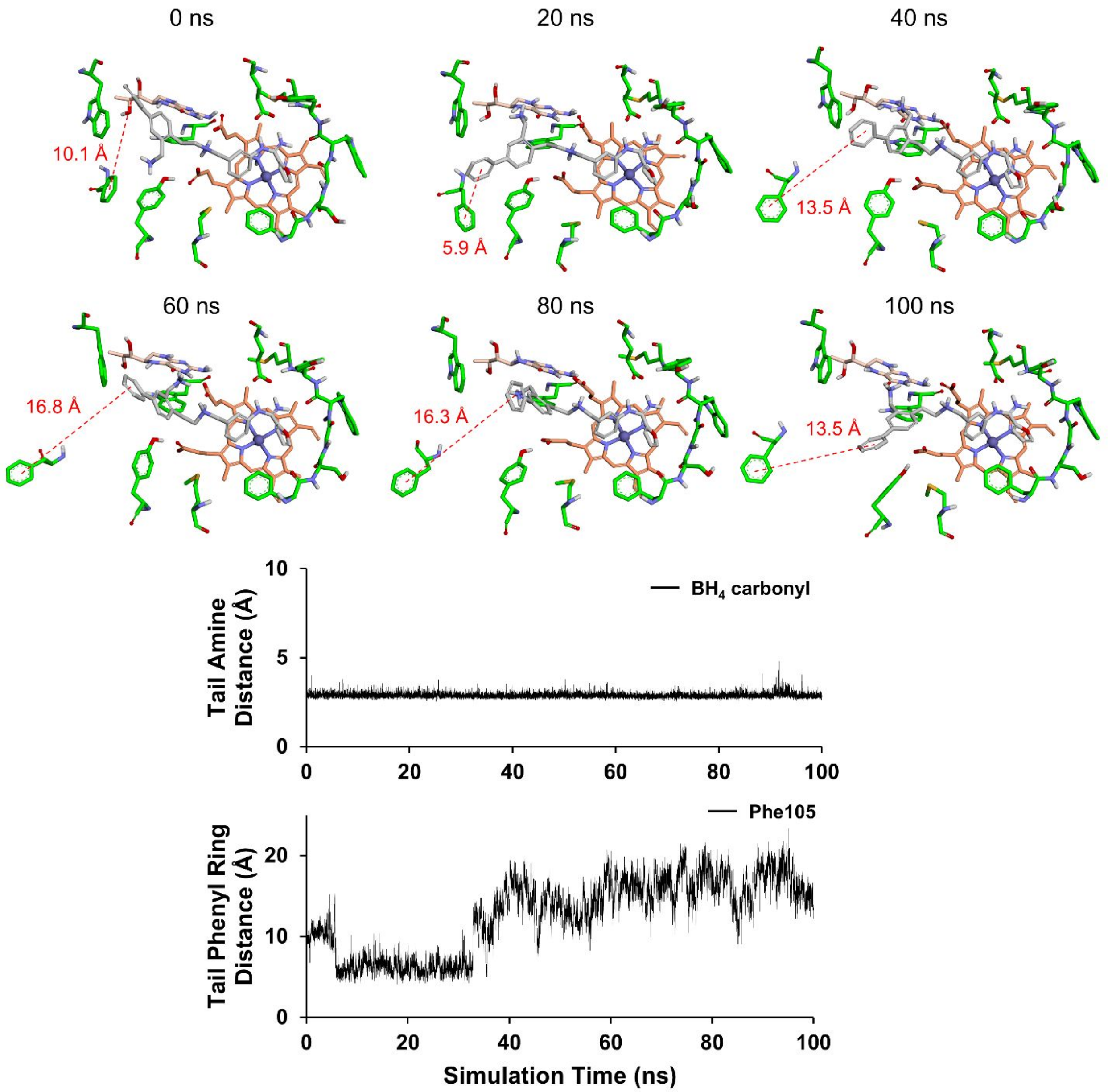

Figure S9. The binding modes of **9** and the indicated distances from the molecular dynamics (MD) simulation trajectories in heNOS after indicated simulation time. The trajectories were simulated from the crystal structures of the heNOS-**9**. Green, orange, and gray sticks, respectively, indicate the heNOS binding site residues, cofactors, and **9**.

9. Effects of synthesized molecules on interferon-gamma (IFN- γ)-induced PD-L1 expression levels in human melanoma A375 cells: Materials and Methods

In previous studies, our nNOS inhibitors have been shown to decrease programmed death ligand 1 (PD-L1) in the presence of IFN-γ, a protein expressed by the melanoma cell as a method to evade immune attack. Compounds AA-02-16 (**9**), AA-02-17 (**10**), AA-02-18 (**11**) were analyzed further to determine their impact on PD-L1 expression in A375 melanoma cells. Changes in PD-L1 expression, detected via flow cytometry, are shown in Figure S10. The bar graph represents mean ± SD as fold of control. The PD-L1 antibodies used were conjugated with Alexa Fluor 647 (#41726S; Cell Signaling Technology, Danvers, MA). The staining protocol for PD-L1 expression was described previously.[30] Mean fluorescence was determined using the BD FACSymphony A1 Cell Analyzer (BD Biosciences, Franklin Lakes, NJ, USA).

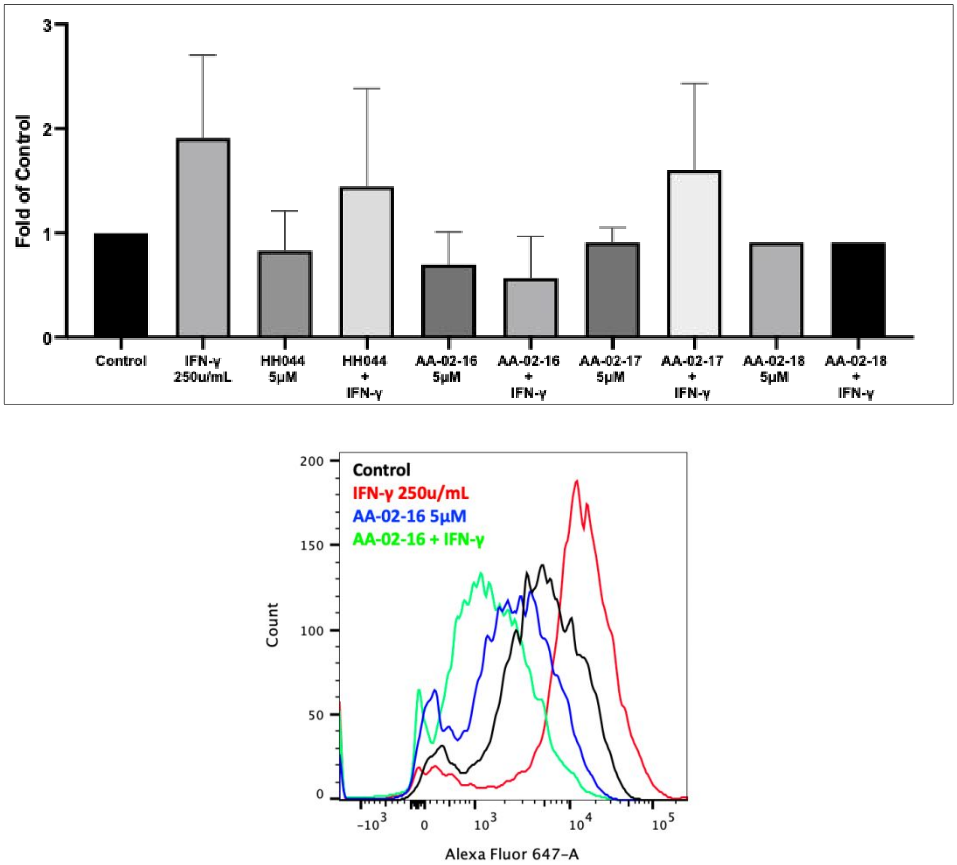

Figure S10. Effect of HH044, **9 (**AA-2-16), **10** (AA-2-17) and **10** (AA-2-18) on programmed death ligand 1 expression with and without IFN-γ.

10. Assessing the potential cellular effects of compound 9, and direct activity on cellular NO production using DAF-FM DA: Materials and Methods

To assess the potential cellular effects of compound **9**, and direct activity on cellular NO production we tested its effects on NO levels in HT22 cells using the DAF-FM NO probe as a supportive readout for cell viability. At 0.5 µM, **9** reduced glutamate-induced NO accumulation toward levels seen in vehicle-treated controls. Preliminary dose–response analyses suggested a concentration-dependent trend.

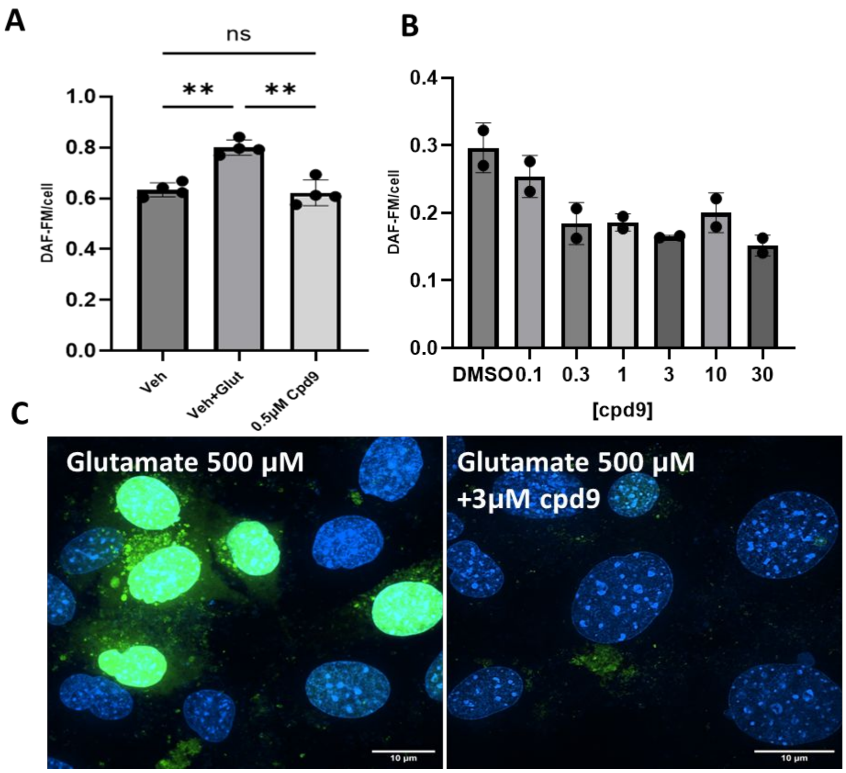

Figure S11. 9 reduces glutamate-induced NO production in HT22 cells. (A) HT22 cells were treated with glutamate (500 µM) in the presence or absence of **9** (0.5 µM). Intracellular NO was quantified using DAF-FM DA and normalized to Hoechst nuclear staining. Compound 9 significantly reduced NO compared with glutamate alone, approaching vehicle levels (**p=0.035**), [n = 3]. **(B)** Increasing concentrations of compound 9 produced a dose-dependent decrease in NO production [n = 2]. **(C)** Qualitative assessment of cellular NO using Super-resolution microscopy images (images with Nikon SORA) showing reduction of DAF-FM signal in 9–treated HT22 cells.

HT22 cells were kindly provided, and preliminary cell culture experiments were performed in the Klein laboratory at Northwestern University. NO levels were quantified using the fluorescent probe 4-amino-5-methylamino-2′,7′-difluorofluorescein diacetate (DAF-FM DA, 5 µM; Invitrogen, #D23844; stock concentration 5 mM). Differentiated HT22 cells were seeded in black-

walled, clear-bottom 96-well plates and treated with **9** at the indicated concentrations for 3 h, followed by glutamate (500 μM) for an additional 3 h. After treatment, cells were incubated with 5 μM DAF-FM DA in serum-free medium at 37 °C for 40 min, then equilibrated in fresh medium for 20 min to allow probe de-esterification. Hoechst 33342 (1:500) was included for nuclear staining to normalize fluorescence signal to cell number. Fluorescence was measured and quantified using a Synergy plate reader (BioTek) with excitation/emission settings of 495/515 nm for DAF-FM and 361/486 nm for Hoechst. DAF-FM fluorescence intensity was normalized to Hoechst signal to yield relative NO levels per cell. Data represent the mean of $n = 2$ independent experiments. For SORA imaging, cells were seeded in Lab-Tek slides #155409 and treated as described above using a single concentration of **9** (3 μM). Cells were imaged for qualitative assessment only of cellular NO in presence and absence of **9**; images were not used for quantitative analysis.

11. Experimental Procedures: Chemistry

General procedure for reductive amination and Boc protection

Syntheses of **25** and **28** were carried out similar to our previously reported method. To a solution of amine intermediates (1 equiv.) and *m*-nitrobenzaldehyde (1.1 equiv.) in anhydrous THF (10 mL) was added glacial acetic acid (0.1 equiv.), and the reaction was stirred for 1 h. $NaBH_3CN$ (1.1 equiv.) was added in portions for 10 min and stirred overnight at the room temperature. The reaction was quenched with dropwise addition of MeOH (2 mL) and concentrated in *vacuo*. The solid residue was taken up with EtOAc (15 mL) and washed with saturated 1M NaOH (5 mL) and brine (20 mL). The organic layer was dried over anhydrous Na_2SO_4, concentrated, and dried *in vacuo* to yield a highly viscous orange oil. To this mixture, *di-tert*-butyl dicarbonate (1.5 equiv.), $NaHCO_3$ (1.2 equiv.), and anhydrous acetonitrile (10 mL) were added. The reaction mixture was stirred for 1 h at room temperature. The reaction mixture was concentrated *in vacuo,* and the solid residues were taken up in EtOAc (50 mL). The organic layer was washed with brine (30 mL), dried over anhydrous Na_2SO_4, concentrated, and dried *in vacuo.* CombiFlash chromatography of the crude product using 15% EtOAc in hexane gave the intermediates.

***tert*-Butyl (3-nitrobenzyl)(4-nitrophenethyl)carbamate[1] (25)** ^{1}H NMR (500 MHz, $CDCl_3$) δ 8.16 – 8.06 (m, 4H), 7.54 – 7.48 (m, 2H), 7.33 – 7.29 (m, 2H), 4.48 - 4.40 (m, 2H), 3.62 – 3.46 (m, 2H), 2.94 - 2.92 (m, 2H), 1.46 (s, 9H).

***tert*-Butyl (3-nitrobenzyl)(3-nitrophenethyl)carbamate[1] (28)** ^{1}H NMR (500 MHz, CD_3OD) δ 8.13 – 8.03 (m, 4H), 7.63 – 7.48 (m, 4H), 4.55 - 4.51 (m, 2H), 3.60 – 3.59 (m, 2H), 2.99 - 2.94 (m, 2H), 1.40 (s, 9H).

General procedure for nitro group reduction

Syntheses of **26** and **29** were carried out similar to our previously reported method. The procedure was followed by the previously reported method. A mixture of *tert*-butyl (3-nitrobenzyl)(3-nitrophenethyl)carbamate (118 mg) and palladium on activated carbon (10%, 36 mg) in MeOH (10 mL) was stirred under a hydrogen atmosphere (1 atm) at rt overnight. The reaction mixture was filtered through a pad of Celite and concentrated under reduced pressure and carried onto the next step without further purification.

General procedure to synthesize head groups 23 (a-g)

Triethylamine (1.2 equiv.) and ammonium sulfide 20% wt solution in water (1.2 equiv.) were added to a solution of aryl carbonitrile (1 equiv.) in pyridine (8 mL). The reaction mixture was stirred at 50 °C for 12 h. After completion of the reaction, the mixture was diluted with cold water and extracted with ethyl acetate (3 x 50 mL). The organic layers were washed with brine, dried over Na_2SO_4, and concentrated in a vacuum to give the carbothioamide intermediates. The product's structure was confirmed by LCMS and was carried out to the next step without purification. A solution of carbothioamide intermediates (1 equiv.) in 20 mL of acetone (reagent grade) was treated with iodomethane (1.1 equiv.) and stirred for 12 h at laboratory temperature. After completion of the reaction, the reaction mixture was concentrated and washed three times with acetone and confirmed with LCMS and used directly in the next step without further purification.

Scheme 4

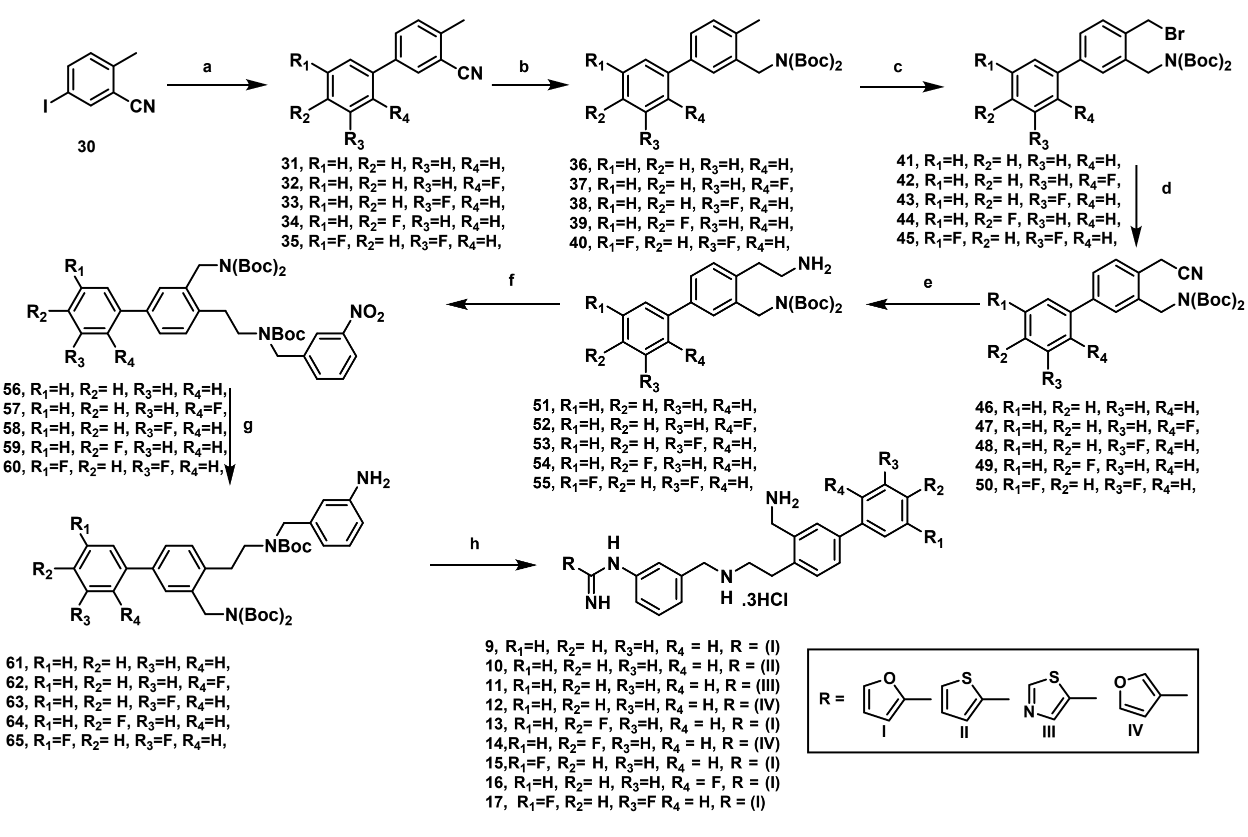

[a]Reagents and conditions: (a) $ArB(OH)_2$, $Pd(dppf)Cl_2$, dioxane-H_2O (9:1), 110 °C, 1h ; (b) (i) $NiCl_2.6H_20$, $NaBH_4$, $(Boc)_2O$, MeOH, rt, 12h, (ii) $(Boc)_2O$, cat. DMAP, NEt_3, ACN, 50 °C, 1h (c) NBS, AIBN, CCl_4, reflux, 12h; (d) KCN, TBAI, DCM-H_2O (9:1), 24 h; (e) $NiCl_2.6H_20$, $NaBH_4$, MeOH, rt, 12h, (f) *m*-nitro benzaldehyde, $NaBH_3CN$, glacial AcOH, THF, 0 °C to rt, 2h, then $(Boc)_2$. $NaHCO_3$ ACN, rt, 1 h, (g) Pd/C, H_2, MeOH, rt, 24 h; (i) (i) R(NH)SMe.HI, EtOH, rt, 24 h; (ii) HCl (3 M in dioxane), rt, 2 h

General procedure to synthesize 31, 32, 33, 34, 35 (Suzuki reaction)

A Biotage® microwave vial (2-6 mL) was charged with **30** (1 equiv.), arylboronic acid (1.1 equiv), potassium carbonate (3 equiv.), and $PdCl_2$(dppf) (0.02 equiv.) under air. The vial was sealed with a microwave vial cap containing a Reseal® septum and evacuated/backfilled with nitrogen (×3). Dioxane (10.8 mL) and water (1.2 mL) were added via syringe. The resulting biphasic mixture was stirred at 110 °C for 1 h (complete consumption of **30** by LCMS). After cooling to room temperature, the reaction mixture filtered through Celite, and the filtrate was partitioned between water (300 mL) and EtOAc (50 mL). The organic layer was separated and the aqueous layer extracted with EtOAc (2 × 50 mL). The combined organic phases were dried (Na_2SO_4), filtered, and concentrated in vacuo. The residue was purified by CombiFlash column chromatography using 3% EtOAc in hexane to afford **31, 32, 33, 34, 35.**

4-Methyl-[1,1'-biphenyl]-3-carbonitrile (31) ^{1}H NMR (500 MHz, CDCl3) δ 7.80 (s, 1H), 7.68 (d, *J* = 8.1 Hz, 1H), 7.53 (d, *J* = 9.2 Hz, 2H), 7.44 (t, *J* = 7.6 Hz, 2H), 7.41 – 7.34 (m, 2H), 2.57 (s, 3H).^{13}C NMR (125 MHz, $CDCl_3$) δ 140.6, 139.6, 138.8, 131.3, 130.9, 130.7, 129.0, 128.0, 126.9, 118.1, 113.3, 20.1.

2'-Fluoro-4-methyl-[1,1'-biphenyl]-3-carbonitrile (32) ^{1}H NMR (500 MHz, $CDCl_3$) δ 7.79 (s, 1H), 7.66 (d, *J* = 8.1 Hz, 1H), 7.39 (d, *J* = 7.9 Hz, 2H), 7.38 – 7.33 (m, 1H), 7.23 (t, *J* = 7.5 Hz, 1H), 7.20 – 7.15 (m, 1H), 2.60 (s, 3H). ^{13}C NMR (125 MHz, $CDCl_3$) δ 160.6, 158.6, 141.2, 134.1, 133.2 (d, *J* = 3.2 Hz), 132.7 (d, *J* = 3.2 Hz), 130.4, 130.3 (d, *J* = 3.2 Hz), 129.8 (d, *J* = 8.4 Hz), 126.7 (d, *J* = 13.2 Hz), 124.6 (d, *J* = 3.6 Hz), 118.0, 116.3 (d, *J* = 22.5 Hz), 113.1, 20.2.

3'-Fluoro-4-methyl-[1,1'-biphenyl]-3-carbonitrile (33) ^{1}H NMR (500 MHz, $CDCl_3$) δ 7.79 (s, 1H), 7.68 (d, *J* = 8.1 Hz, 1H), 7.47 – 7.37 (m, 2H), 7.32 (d, *J* = 7.8 Hz, 1H), 7.24 (d, *J* = 9.8 Hz, 2H), 7.08 (t, *J* = 6.8 Hz, 1H), 2.59 (s, 3H).^{13}C NMR (125 MHz, $CDCl_3$) δ 164.3, 162.4, 141.5, 141.2 (d, *J* = 7.7 Hz), 138.4 (d, *J* = 2.3 Hz), 131.3, 131.0, 130.7 (d, *J* = 8.4 Hz), 122.6 (d, *J* = 3.0 Hz), 118.0, 115.0 (d, *J* = 21.1 Hz), 114.0 (d, *J* = 22.3 Hz), 113.6, 20.3.

4'-Fluoro-4-methyl-[1,1'-biphenyl]-3-carbonitrile (34) ^{1}H NMR (500 MHz, CDCl3) δ 7.76 (d,

J = 2.1 Hz, 1H), 7.64 (dd, *J* = 8.1, 2.1 Hz, 1H), 7.50 (dd, *J* = 8.9, 5.2 Hz, 2H), 7.38 (d, *J* = 8.1 Hz, 1H), 7.15 (t, *J* = 8.7 Hz, 2H), 2.58 (s, 3H) ^{13}C NMR (125 MHz, $CDCl_3$) δ 163.9, 162.0, 140.8, 138.7, 135.1 (d, *J* = 3.4 Hz), 131.2, 130.9 (d, *J* = 7.7 Hz), 128.7 (d, *J* = 8.2 Hz), 118.1, 116.1 (d, *J* = 21.6 Hz), 113.5, 20.2.

3',5'-Difluoro-4-methyl-[1,1'-biphenyl]-3-carbonitrile (35) ^{1}H NMR (500 MHz, $CDCl_3$) δ 7.77 (d, *J* = 2.1 Hz, 1H), 7.65 (dd, *J* = 8.1, 2.1 Hz, 1H), 7.41 (d, *J* = 8.1 Hz, 1H), 7.10 – 7.02 (m, 2H), 6.83 (tt, *J* = 8.8, 2.3 Hz, 1H), 2.60 (s, 3H). ^{13}C NMR (125 MHz, $CDCl_3$) δ 164.5 (d, *J* = 12.9 Hz), 162.6 (d, *J* = 13.2 Hz), 143.4 – 141.4 (m), 137.4 (t, *J* = 2.6 Hz), 131.2 (d, *J* = 5.2 Hz), 130.9, 117.8, 113.8, 110.1 – 109.8 (m), 103.5 (t, *J* = 25.3 Hz), 20.3

General procedure to synthesize 36, 37, 38, 39, 40

To a stirred solution of **31-35** (1 equiv.) in dry CH_3OH (15 mL), cooled to 0 °C, were added di-*tert*-butyl dicarbonate (2 equiv.) and nickel (II) chloride hexahydrate (2 equiv.). $NaBH_4$ (4 equiv.) was then added in portions over 5 min. The reaction was effervescent, immediately forming a finely divided black solid; upon complete addition of the borohydride, the reaction was allowed to progress for 12 h. Evaporation of the solvent gave a purple residue, which was dissolved in diethyl ether and filtered with Celite. The filtrate was extracted with ethyl acetate and saturated $NaHCO_3$ (2×50 mL). The organic layer was dried over Na_2SO_4, and the solvent was removed by rotary evaporation to yield the mono-Boc product, which was dissolved in acetonitrile. To this solution were added di-*tert*-butyl decarbonate (1.6 equiv.), DMAP (0.2 equiv.), and NEt_3 (2 equiv.). The reaction was heated at 50 °C overnight. The reaction mixture was partitioned between water (20 mL) and EtOAc (20 mL). The organic layer was separated and the aqueous layer extracted with EtOAc (2 × 20 mL). The combined organic phases were dried (Na_2SO_4), filtered, and concentrated in vacuo. The residue was purified by CombiFlash column chromatography using 2% EtOAc in hexane to afford the **36, 37, 38, 39, 40**.

***tert*-Butyl (*tert*-butoxycarbonyl)((4-methyl-[1,1'-biphenyl]-3-yl)methyl)carbamate (36)** ^{1}H NMR (500 MHz, $CDCl_3$) δ 7.37 (d, *J* = 7.0 Hz, 2H), 7.27 – 7.18 (m, 4H), 7.15 (t, *J* = 6.7 Hz, 1H), 7.03 (d, *J* = 7.7 Hz, 1H), 4.67 (s, 2H), 2.17 (s, 2H), 1.26 (s, 18H).. ^{13}C NMR (125 MHz, $CDCl_3$) δ 152.7, 141.3, 139.0, 136.8, 134.1, 130.7, 128.8, 127.1, 127.0, 125.4, 124.1, 82.8, 47.3, 28.0, 18.8.

***tert*-Butyl (*tert*-butoxycarbonyl)((2'-fluoro-4-methyl-[1,1'-biphenyl]-3-yl)methyl)carbamate (37)** ^{1}H NMR (500 MHz, $CDCl_3$) δ 7.41 – 7.25 (m, 4H), 7.23 – 7.08 (m, 3H), 4.83 (s, 2H), 2.35 (s, 3H), 1.42 (s, 18H). ^{13}C NMR (125 MHz, $CDCl_3$) δ 160.7, 158.8, 152.5, 148.9, 136.4, 134.4, 133.5, 130.6 (d, J = 3.6 Hz), 130.2, 129.1 (d, J = 13.4 Hz), 128.6 (d, J = 8.2 Hz), 127.2 (d, J = 3.2 Hz), 125.8 (d, J = 3.0 Hz), 124.2 (d, J = 3.6 Hz), 116.0 (dd, J = 22.7, 4.3 Hz), 83.9, 82.5, 47.1, 27.9, 18.9.

***tert*-Butyl (*tert*-butoxycarbonyl)((3'-fluoro-4-methyl-[1,1'-biphenyl]-3-yl)methyl)carbamate (38)** ^{1}H NMR (500 MHz, $CDCl_3$) δ 7.39 – 7.33 (m, 2H), 7.31 (d, J = 9.3 Hz, 2H), 7.26 – 7.16 (m, 2H), 7.04 – 6.96 (m, 1H), 4.83 (s, 2H), 2.35 (s, 3H), 1.44 (s, 18H). ^{13}C NMR (125 MHz, $CDCl_3$) δ 164.1, 162.2, 152.6, 143.4 (d, J = 7.7 Hz), 137.6 (d, J = 2.3 Hz), 136.9, 134.7, 130.7, 130.1 (d, J = 8.4 Hz), 125.2, 123.9, 122.4 (d, J = 2.7 Hz), 113.7 (dd, J = 21.6, 3.9 Hz), 82.6, 47.1, 27.9, 18.7.

***tert*-Butyl (*tert*-butoxycarbonyl)((4'-fluoro-4-methyl-[1,1'-biphenyl]-3-yl)methyl)carbamate (39)** ^{1}H NMR (500 MHz, $CDCl_3$) δ 7.63 (dd, J = 8.7, 5.3 Hz, 2H), 7.51 – 7.44 (m, 2H), 7.34 (d, J = 7.7 Hz, 1H), 7.24 (t, J = 8.7 Hz, 2H), 4.98 (s, 2H), 2.49 (s, 3H), 1.58 (s, 18H). ^{13}C NMR (125 MHz, $CDCl_3$) δ 163.3, 161.3, 152.6, 137.9, 137.3 (d, J = 3.2 Hz), 136.8, 134.0, 130.6, 128.3 (d, J = 8.2 Hz), 125.2, 123.9, 115.5 (d, J = 21.3 Hz), 82.5, 47.2, 18.7.

***tert*-Butyl (*tert*-butoxycarbonyl)((3',5'-fluoro-4-methyl-[1,1'-biphenyl]-3-yl)methyl)carbamate (40)** ^{1}H NMR (500 MHz, $CDCl_3$) 7.32 (dd, J = 7.8, 2.0 Hz, 1H), 7.29 (d, J = 1.9 Hz, 1H), 7.21 (d, J = 7.8 Hz, 1H), 7.08 – 7.00 (m, 2H), 6.75 (tt, J = 8.9, 2.3 Hz, 1H), 4.83 (s, 2H), 2.35 (s, 3H), 1.45 (s, 18H).^{13}C NMR (125 MHz, $CDCl_3$) δ 164.2 (d, J = 13.2 Hz), 162.3 (d, J = 13.1 Hz), 152.7, 144.5 (t, J = 9.5 Hz), 137.1, 136.6 (t, J = 2.5 Hz), 135.5, 130.8, 125.1, 123.8, 110.0 – 108.7 (m), 102.1 (t, J = 25.5 Hz), 82.7, 47.1, 27.9, 18.8.

General procedure to synthesize 41, 42, 43, 44, 45

To a solution of **36-40** (1 equiv.) in CCl_4 (20 mL) were added NBS (1.2 equiv.) and AIBN (0.3 equiv.). After heating under reflux overnight, the mixture was filtered. The filtrate was washed with brine, dried over anhydrous Na_2SO_4, and concentrated to a light-yellow solid. The residue was purified by CombiFlash column chromatography using 2.5% EtOAc in hexane to afford **41,**

42, 43, 44, 45. However, after the column, the desired product contained over brominated impurity, hence the mixture was carried out to the next step.

General procedure to synthesize 46, 47, 48, 49, 50

A 100 mL round bottom flask was charged with **41-45** (1 equiv.), potassium cyanide (1.5 equiv.), and tetrabutylammonium iodide (0.2 equiv.) under an argon atmosphere. To this, a mixture of DCM and water in a 1:1 ratio (10 mL) was added, and the resulting biphasic mixture was stirred vigorously for 24 h at room temperature. After completion, as indicated by TLC, the organic phase was carefully separated from the aqueous phase and collected in a conical flask. The aqueous phase was extracted with DCM (2 x 50 mL). The combined organic layers were washed with brine, dried over Na_2SO_4, filtered, and concentrated under reduced pressure. The residue was purified by CombFlash column chromatography using 10 % EtOAc in hexane to afford the **46, 47, 48**, **49, 50**

***tert*-butyl (*tert*-butoxycarbonyl)((4-(cyanomethyl)-[1,1'-biphenyl]-3-yl)methyl)carbamate (46)** ^{1}H NMR (500 MHz, $CDCl_3$) δ 7.58 – 7.51 (m, 2H), 7.50 – 7.41 (m, 3H), 7.36 (t, *J* = 7.3 Hz, 1H), 4.85 (s, 2H), 3.90 (s, 2H), 1.45 (s, 19H). ^{13}C NMR (125 MHz, $CDCl_3$) δ 152.6, 141.4, 140.1, 136.4, 129.4, 128.8, 127.6, 127.0, 126.7, 126.6, 126.5, 117.6, 83.1, 46.4, 28.1, 21.0.

***tert*-butyl (*tert*-butoxycarbonyl)((4-(cyanomethyl)- 2'-fluoro- [1,1'-biphenyl]-3-yl)methyl)carbamate (47)**^{1}H NMR (500 MHz, $CDCl_3$) δ 7.49-7.46 (m, 3H), 7.39 (t, *J* = 6.9 Hz, 1H), 7.35 – 7.30 (m, 1H), 7.20 (t, *J* = 8.1 Hz, 1H), 7.17 – 7.12 (m, 1H), 4.85 (s, 2H), 3.90 (s, 2H), 1.44 (s, 18H). ^{13}C NMR (125 MHz, $CDCl_3$) δ 160.7, 158.7, 152.60, 136.1 (d, *J* = 6.4 Hz), 130.5 (d, *J* = 3.2 Hz), 129.3 (d, *J* = 8.2 Hz), 129.0, 128.4 (dd, *J* = 5.6, 3.1 Hz), 128.1 (d, *J* = 13.2 Hz), 124.4 (d, *J* = 3.9 Hz), 117.5, 116.2 (d, *J* = 22.5 Hz), 83.3, 46.3, 27.9, 21.0.

***tert*-butyl (*tert*-butoxycarbonyl)((4-(cyanomethyl)- 3'-fluoro- [1,1'-biphenyl]-3-yl)methyl)carbamate (48)**^{1}H NMR (500 MHz, $CDCl_3$) δ 7.52 (s, 1H), 7.49 (s, 2H), 7.44 – 7.36 (m, 1H), 7.33 (d, *J* = 8.1 Hz, 1H), 7.24 (d, *J* = 10.0 Hz, 1H), 7.09 – 7.02 (m, 1H), 4.85 (s, 2H), 3.91 (s, 2H), 1.46 (s, 18H). ^{13}C NMR (125 MHz, $CDCl_3$) δ 164.1, 162.2, 142.4 (d, *J* = 7.7 Hz), 140.1, 136.6, 130.3 (d, *J* = 8.4 Hz), 129.5, 127.4, 126.6 (d, *J* = 28.6 Hz), 122.6 (d, *J* = 2.7 Hz), 117.5, 114.4 (d, *J* = 21.1 Hz), 113.9 (d, *J* = 22.0 Hz), 83.2, 46.3, 28.0, 21.0.

***tert*-butyl (*tert*-butoxycarbonyl)((4-(cyanomethyl)- 4'-fluoro- [1,1'-biphenyl]-3-yl)methyl)carbamate (49)** ^{1}H NMR (500 MHz, $CDCl_3$) δ 7.52 – 7.41 (m, 5H), 7.14 – 7.06 (m, 2H), 4.82 (s, 2H), 3.88 (s, 2H), 1.43 (s, 18H). ^{13}C NMR (125 MHz, $CDCl_3$) δ 163.6, 161.6, 152.6, 140.4, 136.5, 136.3 (d, *J* = 3.2 Hz), 129.4, 128.5 (d, *J* = 8.2 Hz), 126.8, 126.4 (d, *J* = 25.9 Hz), 117.5, 115.7 (d, *J* = 21.6 Hz), 83.1, 46.3, 27.9, 21.0.

***tert*-butyl (*tert*-butoxycarbonyl)((4-(cyanomethyl)- 3', 5'-difluoro- [1,1'-biphenyl]-3-yl)methyl)carbamate (50)** ^{1}H NMR (500 MHz, $CDCl_3$) δ 7.53 – 7.44 (m, 3H), 7.09 – 7.02 (m, 2H), 6.80 (tt, *J* = 8.8, 2.3 Hz, 1H), 4.84 (s, 2H), 3.92 (s, 2H), 1.47 (s, 18H). ^{13}C NMR (125 MHz, $CDCl_3$) δ 164.3 (d, *J* = 13.0 Hz), 162.3 (d, *J* = 13.2 Hz), 152.7, 143.5 (d, *J* = 9.3 Hz), 139.0, 136.9, 129.6, 128.1, 126.7, 126.4, 117.4, 110.8 – 109.1 (m), 102.9 (t, *J* = 25.4 Hz), 83.3, 46.2, 28.0, 21.0.

General procedure to synthesize 51, 52, 53, 54, 55

To a stirred solution of **46-50** (1 equiv.) in CH_3OH (10 mL), cooled to 0 °C, was added nickel (II) chloride hexahydrate (1.2 equiv.). $NaBH_4$ (3 equiv.) was then added in portions over 10 min. The reaction mixture was stirred at rt for 12 h. Evaporation of the solvent gave a purple residue, which was dissolved in EtOAc (50 mL) and extracted with saturated $NaHCO_3$ (2×50mL). The organic layer was dried over Na_2SO_4, and the solvent was removed by rotary evaporation to yield a black residue. The products were confirmed by LCMS and carried out further without purification.

General procedure to synthesize 56, 57, 58, 59, 60

To a solution of **55-60** (1 equiv.) and *m*-nitrobenzaldehyde (1 equiv.) in anhydrous THF (10 mL) was added glacial acetic acid (0.1 equiv.), and the reaction mixture was stirred for 1 h. $NaBH_3CN$ (1 equiv.) was added in portions for 10 min and stirred overnight at ambient temperature. The reaction mixture in an ice bath was quenched with dropwise addition of MeOH (2 mL) to the reaction mixture and concentrated in *vacuo*. The solid residue was taken up in EtOAc (15 mL) and washed with saturated 1M NaOH (5 mL) and brine (20 mL). The organic layer was dried over anhydrous Na_2SO_4, concentrated, and dried *in vacuo* to yield a highly viscous orange oil. To this mixture, *di-tert*-butyl dicarbonate (1.5 equiv.), $NaHCO_3$ (1.2 equiv.), and anhydrous acetonitrile

(10 mL) were added. The reaction mixture was stirred for 2 h at room temperature. The reaction mixture was concentrated *in vacuo,* and the solid residues were taken up in EtOAc (50 mL). The organic layer was washed with brine (30 mL), dried over anhydrous Na_2SO_4, concentrated, and dried *in vacuo.* CombiFlash chromatography of the crude product using 15% EtOAc in hexane gave **56, 57, 58, 59, 60** as a colorless oil.

***tert*-butyl (*tert*-butoxycarbonyl)((4-(2-((*tert*-butoxycarbonyl)(3-nitrobenzyl)amino)ethyl)-[1,1'-biphenyl]-3-yl)methyl)carbamate (56)** ^{1}H NMR (500 MHz, $CDCl_3$) δ 8.09 (s, 2H), 7.58 – 7.44 (m, 4H), 7.41 – 7.36 (m, 4H), 7.31 – 7.29 (m, 1H), 7.18 – 7.10 (m, 1H), 4.84 (d, *J* = 16.2 Hz, 2H), 4.50 - 4.35 (m, 2H), 3.42 (d, *J* = 42.7 Hz, 2H), 2.92 (d, *J* = 26.0 Hz, 2H), 1.52 – 1.40 (m, 27H).^{13}C NMR (125 MHz, $CDCl_3$) δ 152.7, 148.4, 140.8, 139.8, 136.7, 134.7, 133.6, 133.1, 130.5, 129.5, 128.7, 127.2, 126.9, 125.7, 124.7, 122.3, 82.7, 80.6, 60.4, 50.0, 46.3, 31.6, 28.4, 27.9.

***tert*-butyl (*tert*-butoxycarbonyl)((4-(2-((*tert*-butoxycarbonyl)(3-nitrobenzyl)amino)ethyl)-2'-fluoro-[1,1'-biphenyl]-3-yl)methyl)carbamate (57)** ^{1}H NMR (500 MHz, $CDCl_3$) δ 8.1-8.10 (m, 2H), 7.60 (s, 1H), 7.48 (t, *J* = 8.8 Hz, 1H), 7.35 (d, *J* = 9.4 Hz, 3H), 7.30 (t, *J* = 7.6 Hz, 1H), 7.22 – 7.07 (m, 3H), 4.85 (d, *J* = 20.7 Hz, 2H), 4.53 (s, 1H), 4.37 (s, 1H), 3.49 (d, *J* = 14.1 Hz, 2H), 2.95 (d, *J* = 26.0 Hz, 2H), 1.403-1.42 (m, 27H). ^{13}C NMR (125 MHz, $CDCl_3$) δ 160.7, 158.7, 155.8, 152.6, 148.4, 140.7, 136.3, 133.6, 133.2, 130.5, 130.1, 129.5, 128.8, 127.6, 126.6, 124.3 (d, *J* = 3.6 Hz), 122.3, 116.1 (d, *J* = 22.9 Hz), 82.7, 80.6, 50.0, 48.1, 46.3, 34.4, 28.4, 27.9.

***tert*-butyl (*tert*-butoxycarbonyl)((4-(2-((*tert*-butoxycarbonyl)(3-nitrobenzyl)amino)ethyl)-3'-fluoro-[1,1'-biphenyl]-3-yl)methyl)carbamate (58)** ^{1}H NMR (500 MHz, $CDCl_3$) δ 8.14 – 8.09 (m, 2H), 7.63 – 7.43 (m, 2H), 7.43 – 7.33 (m, 3H), 7.30 (d, *J* = 7.7 Hz, 1H), 7.21 (d, *J* = 9.8 Hz, 2H), 7.14 (d, *J* = 6.4 Hz, 1H), 7.06 – 6.97 (m, 1H), 4.85 (d, *J* = 17.4 Hz, 2H), 4.44 (d, *J* = 75.3 Hz, 2H), 3.43 (d, *J* = 38.8 Hz, 2H), 2.94 (d, *J* = 23.1 Hz, 2H), 1.49 – 1.40 (m, 27H).^{13}C NMR (125 MHz, $CDCl_3$) δ 164.1, 162.2, 152.7, 148.4, 143.1, 140.7, 138.5, 136.8, 133.6, 130.6, 130.1 (d, *J* = 8.4 Hz), 129.5, 125.6, 124.6, 122.4 (d, *J* = 2.5 Hz), 122.3, 114.0 (d, *J* = 19.3 Hz), 113.7 (d, *J* = 22.0 Hz), 82.8, 80.6, 50.1, 48.1, 46.3, 34.4, 28.4, 27.9.

***tert*-butyl (*tert*-butoxycarbonyl)((4-(2-((*tert*-butoxycarbonyl)(3-nitrobenzyl)amino)ethyl)-4'-**

fluoro-[1,1'-biphenyl]-3-yl)methyl)carbamate (59) ^{1}H NMR (500 MHz, $CDCl_3$) δ 8.11 (dq, J = 4.9, 1.5 Hz, 2H), 7.48 (ddd, J = 12.4, 7.0, 3.1 Hz, 4H), 7.41 – 7.29 (m, 2H), 7.15 – 7.05 (m, 3H), 4.86 (d, J = 30.8 Hz, 3H), 4.45 (d, J = 70.8 Hz, 2H), 3.43 (d, J = 44.3 Hz, 2H), 3.03 – 2.83 (m, 2H), 1.47 – 1.37 (m, 27H). ^{13}C NMR (125 MHz, $CDCl_3$) δ 163.4, 161.4, 155.8 (d, J = 11.4 Hz), 155.1, 152.7, 152.7, 148.4, 140.9 (d, J = 38.4 Hz), 138.7 (d, J = 25.0 Hz), 136.8 (d, J = 21.8 Hz), 135.6 – 134.3 (m), 133.6, 133.2, 130.6, 130.3, 129.5, 128.4 (d, J = 7.9 Hz), 125.5 (d, J = 10.0 Hz), 124.6 (d, J = 10.9 Hz), 122.3, 115.6 (d, J = 23.6 Hz), 82.7, 80.61, 65.1, 48.1, 46.3, 29.7, 28.4, 27.9.

General procedure to synthesize 61, 62, 63, 64, 65

A mixture of **56-60** and palladium on activated carbon (10%) in MeOH (3 mL) was stirred under a hydrogen atmosphere (1 atm) at rt overnight. The reaction mixture was filtered through a pad of Celite and concentrated under reduced pressure. **61-65** were confirmed by LCMS and carried out further without purification.

Scheme 5

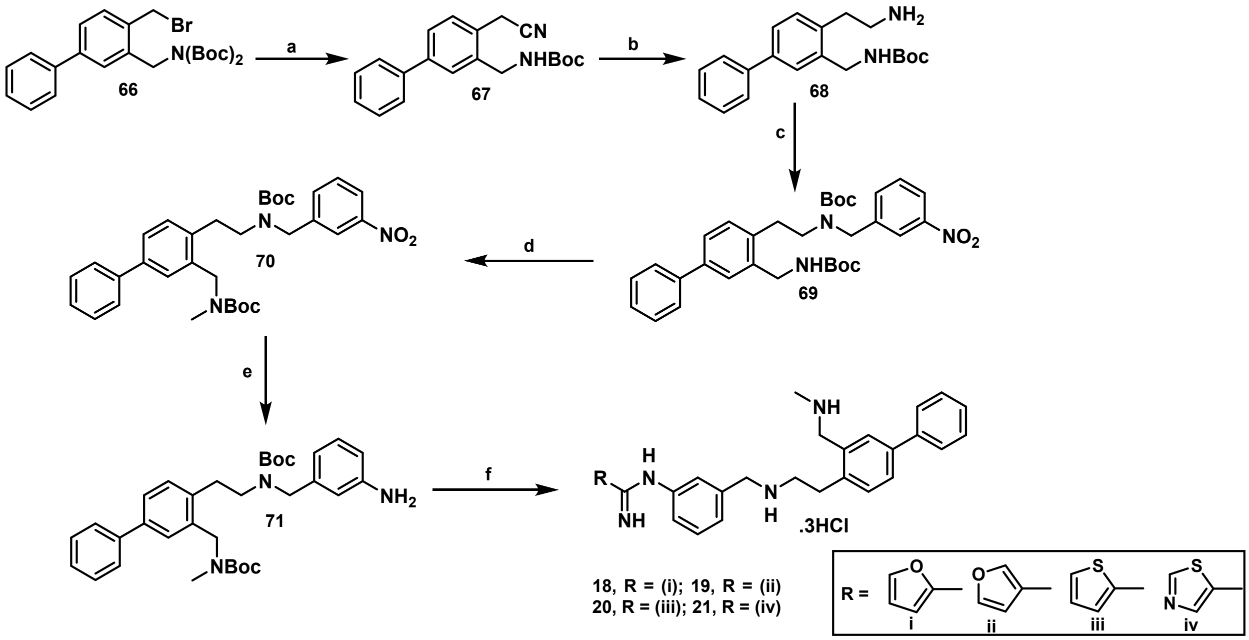

[a]Reagents and conditions: (a)(i) KCN, TBAI, DCM-H_2O (9:1), 24 h. (ii) TFA, DCM, rt, 1 h (b) $NiCl_2.6H_20$, $NaBH_4$, MeOH, rt, 12 h; (c) *m*-nitro benzaldehyde, $NaBH_3CN$, glacial AcOH, THF, 0 °C to rt, 2 h, then $(Boc)_2$. $NaHCO_3$ ACN, rt, 1 h, (d) NaH, MeI, 0 °C to rt, 2 h (e) Pd/C, H_2, MeOH, rt, 24 h; (f)(i) R(NH)SMe.HI, EtOH, rt, 24 h; (ii) HCl (3 M in dioxane), rt, 2 h

***tert*-Butyl ((4-(2-((tert-butoxycarbonyl)(3-nitrobenzyl)amino)ethyl)-[1,1'-biphenyl]-3-yl)methyl)(methyl)carbamate (70)**

Synthesis of **67**, **68**, **69** and **70** were carried out similar to that in Scheme 2. To a stirred solution of **62** (342 mg, 0.6096 mmol) in dry THF (3 mL) at 0 °C was slowly added NaH (60% dispersion in mineral oil) (29 mg, 0.7315 mmol) under nitrogen. The reaction mixture was stirred at room temperature for 30 min, and then methyl iodide (0.2 mL, 1.219 mmol) was added. The reaction mixture was stirred for a further 2 h and then quenched with the addition of H_2O (5 mL). The aqueous solution was extracted with EtOAc (3 × 100 mL). The combined organic layers were washed with H_2O (3 × 100 mL), brine (2 × 100 mL), dried over anhydrous Na_2SO_4, filtered, and evaporated to dryness. CombiFlash chromatography of the crude product using 25% EtOAc in hexane gave **63** as a colorless oil. ^{1}H NMR (500 MHz, $CDCl_3$) δ 7.55 (d, *J* = 7.5 Hz, 2H), 7.42 (ddd, *J* = 7.9, 4.8, 2.8 Hz, 3H), 7.34 (d, *J* = 7.2 Hz, 2H), 7.25 – 7.05 (m, 2H), 6.69 – 6.50 (m, 3H), 4.65 – 4.19 (m, 4H), 3.33 (dt, *J* = 42.5, 8.1 Hz, 2H), 2.94 – 2.72 (m, 5H), 1.64 – 1.32 (m, 18H).^{13}C NMR (125 MHz, $CDCl_3$) δ 155.9, 155.1, 148.4, 140.7, 139.8, 135.8, 133.6, 133.1, 130.8, 129.5, 128.8, 127.3, 126.9, 126.1, 122.3, 122.2, 80.5, 79.9, 49.6, 48.5, 48.1, 33.8, 28.4, 28.4.

***tert*-Butyl ((4-(2-((3-aminobenzyl)(*tert*-butoxycarbonyl)amino)ethyl)-[1,1'-biphenyl]-3-yl)methyl)(methyl)carbamate (71)**

A mixture of **70** and palladium on activated carbon (10%) in MeOH (3 mL) was stirred under a hydrogen atmosphere (1 atm) at rt overnight. The reaction mixture was filtered through a pad of Celite and concentrated under reduced pressure. The residue was purified by CombiFlash chromatography of the crude product using 30% EtOAc in hexanes, giving **71** as orange oils.

^{1}H NMR (500 MHz, $CDCl_3$) δ 7.49-7.46 (m, 3H), 7.39 (t, *J* = 6.9 Hz, 1H), 7.35 – 7.30 (m, 1H), 7.20 (t, *J* = 8.1 Hz, 1H), 7.17 – 7.12 (m, 1H), 4.85 (s, 2H), 3.90 (s, 2H), 1.44 (s, 18H).^{13}C NMR (125 MHz, $CDCl_3$) δ 156.1, 155.6, 146.6, 140.9, 139.7, 139.5, 136.4, 135.9, 130.7, 129.5, 128.8, 127.3, 127.0, 126.4, 125.9, 125.4, 118.4, 117.7, 114.5, 79.9, 51.3, 50.3, 47.3, 34.1, 31.1, 28.6, 28.5.

General procedure 4: Coupling reaction of the amine with various headgroups containing a carbimidothioate moiety and salt formation of the final compounds (1-21)

To a solution of the amine (1 equiv.) in EtOH (absolute, 2 mL) was added previously synthesized headgroups consisting of a carbimidothioate (hydroiodide salt) (2 equiv.). The reaction mixture

was stirred at room temperature for 24 h, and the reaction mixture was concentrated *in vacuo*. The crude residue was purified as listed below under subheadings for all final compounds. The semisolid was dissolved in 3M HCl in dioxane (2 mL). The reaction mixture was stirred at room temperature for 24 h, and the reaction mixture was concentrated *in vacuo*. Anhydrous MeOH (1 mL) was added to the crude solid residue, and the mixture was warmed in a 40 ^{o}C water bath. To this transparent solution, anhydrous diethyl ether was added dropwise (~3-5 mL total) until the solution turned cloudy. The resulting solid precipitate was collected by decanting the solution, washed five times with ice-cold anhydrous diethyl ether (20 mL portions), and dried *in vacuo* to yield all final molecules **(1 – 21)** in trihydrochloride salt form.

***N*-(4-(2-((3-(Furan-2-carboximidamido)benzyl)amino)ethyl)phenyl)furan-2-carboximidamide trihydrochloride salt (1)**

Compound **1** was prepared by following General Procedure 4 via coupling of **2b** (324 mg, 1.20 mmol) and **26** (165 mg, 0.4838 mmol). Purification by flash column chromatography using 60% EtOAc in hexane followed by salt formation as described in General Procedure 4, yielded **1** in the trihydrochloride salt form as a yellow solid (120 mg, 46%).**^{1}H NMR** (500 MHz, CD_3OD) δ 8.07 (dd, *J* = 10.7, 1.7 Hz, 2H), 7.84 – 7.66 (m, 5H), 7.58 (t, *J* = 8.7 Hz, 3H), 7.45 (d, *J* = 8.0 Hz, 2H), 6.91 – 6.87 (m, 2H), 4.40 (s, 2H), 3.48 – 3.41 (m, 2H), 3.30 – 3.21 (m, 2H); **^{13}C NMR** (125 MHz, CD_3OD) δ 152.5, 152.2, 148.9, 148.8, 141.1, 141.0, 137.9, 134.2, 133.5, 132.5, 130.8, 130.6, 130.5, 127.3, 126.6, 126.1, 119.1, 118.8, 113.2, 113.1, 50.4, 48.4, 31.4. **HRMS** (ESI) calcd for $C_{25}H_{26}N_5O_2$ [$(M + H)^+$], 428.2081; found, 428.2088.

***N*-(4-(2-((3-(Furan-3-carboximidamido)benzyl)amino)ethyl)phenyl)furan-3-carboximidamide trihydrochloride salt (2)**

Compound **2** was prepared by following General Procedure 4 via coupling of **2e** (98 mg, 0.3665 mmol) and **26** (50 mg, 0.1466 mmol). Purification by flash column chromatography using 100% EtOAc followed by salt formation as described in General Procedure 4, yielded **2** in the trihydrochloride salt form as a pale yellow solid (30 mg, 38%).**^{1}H NMR** (500 MHz, CD_3OD) δ 8.56 (d, *J* = 21.3 Hz, 2H), 7.90 – 7.84 (m, 2H), 7.78 (s, 1H), 7.76 – 7.67 (m, 2H), 7.58 (d, *J* = 8.2 Hz, 3H), 7.46 (d, *J* = 7.9 Hz, 2H), 7.15 – 7.08 (m, 2H), 4.40 (s, 2H), 3.47 – 3.40 (m, 2H), 3.27 – 3.20 (m, 2H).**^{13}C NMR** (125 MHz, CD_3OD) δ 157.7, 157.5, 147.5, 147.3, 145.5, 145.5, 137.9, 134.4, 133.5, 132.8, 130.9, 130.6, 130.5, 127.3, 126.5, 126.1, 116.2, 108.1, 108.0, 50.4, 48.4, 31.4.

HRMS (ESI) calcd for $C_{25}H_{26}N_5O_2$ [(M + H)$^+$], 428.2081; found, 428.2080.

***N*-(4-(2-((3-(Isoxazole-3-carboximidamido)benzyl)amino)ethyl)phenyl)isoxazole-3-carboximidamide trihydrochloride salt (3)**

Compound **3** was prepared by following General Procedure 4 via coupling of **2f** (69 mg, 0.2565 mmol) and **26** (35 mg, 0.1026 mmol). Purification by flash column chromatography using 5% methanol in dichloromethane followed by salt formation as described in General Procedure 4, gave **3** in the trihydrochloride salt form as a white solid (20 mg, 36%).**^{1}H NMR** (500 MHz, CD_3OD) δ 9.16 (dd, *J* = 7.3, 1.4 Hz, 2H), 7.92 – 7.68 (m, 3H), 7.62 (d, *J* = 7.3 Hz, 3H), 7.50 (d, *J* = 7.4 Hz, 2H), 7.30 (dd, *J* = 20.3, 1.6 Hz, 2H), 4.42 (s, 2H), 3.49-3.42 (m, 2H), 3.27-3.24 (m, 2H).**^{13}C NMR** (125 MHz, CD_3OD) δ 162.7, 154.5, 154.3, 153.5, 138.4, 133.8, 133.6, 132.2, 131.1, 131.0, 130.8, 127.3, 126.5, 126.0, 104.1, 103.9, 50.5, 48.5, 31.5. **HRMS** (ESI) calcd for $C_{23}H_{24}N_7O_2$ [(M + H)$^+$], 430.1986; found, 430.1987

***N*-(4-(2-((3-(Thiophene-3-carboximidamido)benzyl)amino)ethyl)phenyl)thiophene-3-carboximidamide trihydrochloride salt (4)**

Compound **4** was prepared by following General Procedure 4 via coupling of **2g** (100 mg, 0.3519 mmol) and **26** (40 mg, 0.1173 mmol). Purification by flash column chromatography using 10% methanol in dichloromethane followed by salt formation as described in General Procedure 4, gave **4** in the trihydrochloride salt form as a pale yellow solid (20 mg, 33%).**^{1}H NMR** (500 MHz, CD_3OD) δ 8.60 – 8.49 (m, 2H), 7.80 (td, *J* = 5.8, 2.9 Hz, 3H), 7.75 – 7.66 (m, 4H), 7.59 (d, *J* = 7.9 Hz, 3H), 7.47 (d, *J* = 8.2 Hz, 2H), 4.40 (s, 2H), 3.48 – 3.35 (m, 2H), 3.26 – 3.17 (m, 2H); **^{13}C NMR** (125 MHz, CD_3OD) δ 158.7, 158.5, 137.8, 134.7, 133.5, 133.1, 132.7, 132.4, 130.9, 130.6, 130.4, 130.3, 129.3, 128.5, 127.2, 126.5, 126.0, 125.9, 125.8, 50.4, 48.4, 31.4. **HRMS** (ESI) calcd for $C_{25}H_{26}N_5S_2$ [(M + H)$^+$], 460.1624; found, 460.1608.

***N*-(4-(2-((3-(Thiazole-2-carboximidamido)benzyl)amino)ethyl)phenyl)thiazole-2-carboximidamide trihydrochloride salt (5)**

Compound **5** was prepared by following General Procedure 4 via coupling of **2c** (280 mg, 0.9853 mmol) and **26** (120 mg, 0.3519 mmol). Purification by flash column chromatography using 70% EtOAc in hexane followed by salt formation as described in General Procedure 4, yielded **5** in the

trihydrochloride salt form as a yellow solid (50 mg, 25%).[1]**H NMR** (500 MHz, CD_3OD) δ 8.29 (d, *J* = 8.4 Hz, 1H), 7.83 (s, 1H), 7.79 – 7.70 (m, 2H), 7.61 (q, *J* = 6.5 Hz, 3H), 7.51 (d, *J* = 8.2 Hz, 2H), 4.42 (s, 2H), 3.46 (dd, *J* = 9.9, 6.2 Hz, 2H), 3.25 (dd, *J* = 9.9, 6.0 Hz, 2H). **^{13}C NMR** (125 MHz, CD_3OD) δ 155.3, 155.1, 153.7, 153.6 145.0, 145.0, 138.2, 134.2, 133.5, 132.6, 130.9, 130.8, 130.7, 129.7, 127.7, 127.5, 127.4, 126.6, 126.1, 123.5, 50.5, 48.4, 31.4. **HRMS** (ESI) calcd for $C_{23}H_{24}N_7S_2$ [(M + H)$^+$], 462.1529; found, 462.1539.

***N*-(4-(2-((3-(Thiazole-5-carboximidamido)benzyl)amino)ethyl)phenyl)thiazole-5-carboximidamide trihydrochloride salt (6)**

Compound **6** was prepared by following General Procedure 4 via coupling of **2d** (180 mg, 0.6334 mmol) and **26** (90 mg, 0.2639 mmol). Purification by flash column chromatography using 60% EtOAc in hexane followed by salt formation as described in General Procedure 4, gave **6** in the trihydrochloride salt form as a pale yellow solid (30 mg, 20%).**^{1}H NMR** (500 MHz, CD_3OD) δ 9.40 (d, *J* = 33.5 Hz, 2H), 8.69 (d, *J* = 23.5 Hz, 2H), 7.69 – 7.53 (m, 5H), 7.46 (d, *J* = 8.2 Hz, 3H), 4.37 (s, 1H), 3.44 – 3.38 (m, 1H), 3.22 – 3.18 (m, 1H).**^{13}C NMR** (125 MHz, CD_3OD) δ 159.8, 159.2, 154.7, 148.8, 147.7, 138.9, 137.6, 133.2, 130.7, 130.5, 130.3, 128.9, 126.1, 125.6, 125.4, 122.2, 50.6, 48.2, 31.4. **HRMS** (ESI) calcd for $C_{23}H_{24}N_7S_2$ [(M + H)$^+$], 462.1529; found, 462.1526

***N*-(3-(2-((3-(Thiazole-5-carboximidamido)benzyl)amino)ethyl)phenyl)thiazole-5-carboximidamide trihydrochloride salt (7)**

Compound **7** was prepared by following General Procedure 4 via coupling of **2d** (200 mg, 0.6304 mmol) and **29** (86 mg, 0.2521 mmol). Purification by flash column chromatography using 60% EtOAc in hexane followed by salt formation as described in General Procedure 4, gave **7** in trihydrochloride salt form as a white solid (40 mg, 26%).**^{1}H NMR** (500 MHz, CD_3OD) δ 8.07 (d, *J* = 7.3 Hz, 2H), 7.81 – 7.66 (m, 5H), 7.62 – 7.53 (m, 2H), 7.50 (d, *J* = 7.3 Hz, 2H), 7.39 (d, *J* = 8.4 Hz, 1H), 6.89 (ddd, *J* = 4.5, 3.7, 1.8 Hz, 2H), 4.40 (s, 2H), 3.50 – 3.42 (m, 2H), 3.25 – 3.23 (m, 2H).**^{13}C NMR** (125 MHz, CD_3OD) δ 152.4, 152.2, 148.9, 148.8, 141.1, 141.0, 139.2, 134.1, 134.0, 133.5, 130.8, 130.6, 130.5, 129.4, 127.3, 126.6, 126.1, 124.4, 119.1, 118.8, 113.2, 113.1, 50.5, 48.3, 31.5. **HRMS** (ESI) calcd for $C_{23}H_{24}N_7S_2$ [(M + H)$^+$], 462.1529; found, 462.15238.

***N*-(3-(2-((3-(Furan-2-carboximidamido)benzyl)amino)ethyl)phenyl)furan-2-carboximidamide trihydrochloride salt (8)**

Compound **8** was prepared by following General Procedure 4 via coupling of **2b** (265 mg, 0.9912 mmol) and **29** (130 mg, 0.3812 mmol). Purification by flash column chromatography using 70% EtOAc in hexane followed by salt formation as described in General Procedure 4, gave **8** in the trihydrochloride salt form as a white solid (100 mg, 49%).**^{1}H NMR** (500 MHz, CD_3OD) δ 8.07 (dd, *J* = 10.7, 1.7 Hz, 2H), 7.84 – 7.66 (m, 5H), 7.58 (t, *J* = 8.7 Hz, 3H), 7.45 (d, *J* = 8.0 Hz, 2H), 6.91 – 6.87 (m, 2H), 4.40 (s, 2H), 3.48 – 3.41 (m, 2H), 3.30 – 3.21 (m, 2H); ^{13}C NMR (125 MHz, CD_3OD) δ 152.5, 152.2, 148.9, 148.8, 141.1, 141.0, 137.9, 134.2, 133.5, 132.5, 130.8, 130.6, 130.5, 127.3, 126.6, 126.1, 119.1, 118.8, 113.2, 113.1, 50.4, 48.4, 31.4. **HRMS** (ESI) calcd for $C_{25}H_{26}N_5O_2$ [(M + H)$^+$], 428.2081; found, 428.2080.

_N_-(3-(((2-(3-(Aminomethyl)-[1,1'-biphenyl]-4-yl)ethyl)amino)methyl)phenyl)furan-2-carboximidamide trihydrochloride salt (9)

Compound **9** was prepared by following General Procedure 4 via coupling of **2b** (33 mg, 0.1225 mmol) and **61** (43 mg, 0.0680 mmol). Purification by flash column chromatography using 80% EtOAc in hexane followed by salt formation as described in General Procedure 4, yielded **9** in the trihydrochloride salt form as a white yellow solid (20 mg, 55%).**^{1}H NMR** (500 MHz, D_2O) δ 7.93 (s, 1H), 7.76 – 7.68 (m, 5H), 7.64 – 7.53 (m, 5H), 7.49 – 7.44 (m, 3H), 6.76 – 6.72 (m, 1H), 4.38 (s, 2H), 4.35 (s, 2H), 3.42 (t, *J* = 8.0 Hz, 2H), 3.24 – 3.21 (m, 2H).**^{13}C NMR** (125 MHz, D_2O) δ 152.4, 149.0, 140.5, 139.2, 134.1, 133.8, 132.8, 131.6, 131.3, 130.9, 130.4, 129.2, 128.1, 128.1, 128.0, 127.0, 126.9, 126.7, 119.3, 113.4, 50.4, 47.1, 39.7, 28.1. **HRMS** (ESI) calcd for $C_{27}H_{29}N_4O$ [(M + H)$^+$], 425.2336; found, 425.2344.

_N_-(3-(((2-(3-(Aminomethyl)-[1,1'-biphenyl]-4-yl)ethyl)amino)methyl)phenyl)thiophene-2-carboximidamide trihydrochloride salt (10)

Compound **10** was prepared by following General Procedure 4 via coupling of **2a** (35 mg, 0.1225 mmol) and **61** (43 mg, 0.0680 mmol). Purification by flash column chromatography using 70% EtOAc in hexane followed by salt formation as described in General Procedure 4, gave **10** in the trihydrochloride salt form as a yellow solid (22 mg, 61%).**^{1}H NMR** (500 MHz, D_2O) δ 8.03 – 7.96 (m, 2H), 7.79 – 7.68 (m, 5H), 7.64 (d, *J* = 7.8 Hz, 1H), 7.56 (dt, *J* = 15.2, 7.7 Hz, 3H), 7.51 – 7.44 (m, 3H), 7.36 (s, 1H), 4.39 (s, 2H), 4.36 (s, 2H), 3.42 (t, *J* = 7.7 Hz, 2H), 3.23 (t, *J* = 8.0 Hz, 2H).**^{13}C NMR** (125 MHz, D_2O) δ 158.0, 140.4, 139.2, 134.5, 134.4, 134.0, 132.1, 131.6, 131.3,

130.9, 130.4, 129.2, 128.9, 128.1, 128.1, 128.0, 126.9, 126.8, 50.4, 47.1, 39.8, 28.1. **HRMS** (ESI) calcd for $C_{27}H_{29}N_4S$ [(M + H)$^+$], 441.2107; found, 441.2118.

***N*-(3-(((2-(3-(Aminomethyl)-[1,1'-biphenyl]-4-yl)ethyl)amino)methyl)phenyl)thiazole-5-carboximidamide trihydrochloride salt (11)**

Compound **11** was prepared by following General Procedure 4 via coupling of **2d** (35 mg, 0.1225 mmol) and **61** (43 mg, 0.0680 mmol). Purification by flash column chromatography using 60% EtOAc in hexane followed by salt formation as described in General Procedure 4, produced **11** in the trihydrochloride salt form as a white solid (25 mg, 66%).**^{1}H NMR** (500 MHz, D_2O) δ 9.41 (s, 1H), 8.64 (s, 1H), 7.79 – 7.69 (m, 5H), 7.64 (d, *J* = 7.7 Hz, 1H), 7.61 – 7.52 (m, 3H), 7.50 – 7.40 (m, 3H), 4.39 (s, 2H), 4.36 (s, 2H), 3.48 – 3.38 (m, 2H), 3.23 (t, *J* = 7.9 Hz, 2H).**^{13}C NMR** (125 MHz, D_2O) δ 160.9, 156.5, 147.6, 140.5, 139.2, 134.3, 134.1, 132.8, 131.7, 131.4, 130.9, 130.6, 129.2, 128.2, 128.1, 128.0, 126.8, 126.6, 125.7, 50.3, 47.1, 39.7, 28.1. **HRMS** (ESI) calcd for C26H28N5S [(M + H)$^+$], 442.2060; found, 442.2073

***N*-(3-(((2-(3-(Aminomethyl)-[1,1'-biphenyl]-4-yl)ethyl)amino)methyl)phenyl)furan-3-carboximidamide trihydrochloride salt (12)**

Compound **12** was prepared by following General Procedure 4 via coupling of **2e** (38 mg, 0.1411 mmol) and **61** (50 mg, 0.0783 mmol). Purification by flash column chromatography using 70% EtOAc in hexane followed by salt formation as described in General Procedure 4, gave **12** in the trihydrochloride salt form as a yellow-white solid (26 mg, 62%).**^{1}H NMR** (500 MHz, D_2O) δ 8.39 (s, 1H), 7.80 – 7.68 (m, 6H), 7.64 (d, *J* = 7.8 Hz, 1H), 7.56 (q, *J* = 7.0 Hz, 3H), 7.51 – 7.43 (m, 3H), 6.96 (s, 1H), 4.39 (s, 2H), 4.36 (s, 2H), 3.42 (t, *J* = 8.0 Hz, 2H), 3.23 (t, *J* = 8.0 Hz, 2H). **^{13}C NMR** (125 MHz, D_2O) δ 157.8, 147.2, 145.6, 140.4, 139.2, 134.0, 134.0, 132.8, 131.6, 131.3, 130.9, 130.5, 129.2, 128.1, 128.1, 128.0, 127.0, 126.8, 115.7, 108.1, 50.4, 47.2, 39.8, 28.1. **HRMS** (ESI) calcd for $C_{27}H_{29}N_4O$ [(M + H)$^+$], 425.2336; found, 425.2349.

***N*-(3-(((2-(3-(Aminomethyl)-4'-fluoro-[1,1'-biphenyl]-4-yl)ethyl)amino)methyl)phenyl)furan-2-carboximidamide trihydrochloride salt (13)**

Compound **13** was prepared by following General Procedure 4 via coupling of **2b** (26 mg, 0.9695 mmol) and **64** (35 mg, 0.0538 mmol). Purification by flash column chromatography using 80%

EtOAc in hexane followed by salt formation as described in General Procedure 4, produced **13** in the trihydrochloride salt form as a yellow solid (18 mg, 60%).^{1}**H NMR** (500 MHz, D_2O) δ 7.85 (s, 1H), 7.61 (dd, *J* = 15.1, 8.1 Hz, 5H), 7.54 (d, *J* = 7.8 Hz, 1H), 7.48 (t, *J* = 7.5 Hz, 2H), 7.38 (d, *J* = 7.9 Hz, 1H), 7.31 (s, 1H), 7.15 (t, *J* = 8.6 Hz, 2H), 6.73 (d, *J* = 3.8 Hz, 1H), 4.29 (s, 2H), 4.25 (s, 2H), 3.33 (t, *J* = 7.9 Hz, 2H), 3.13 (t, *J* = 7.8 Hz, 2H). ^{13}**C NMR** (125 MHz, D_2O) δ 163.5, 161.5, 152.3, 149.0, 140.4, 139.6, 135.3 (d, *J* = 3.2 Hz), 134.0, 133.7, 132.7, 131.6, 131.3, 130.5, 130.4, 128.5 (d, *J* = 8.4 Hz), 128.0 (d, *J* = 4.3 Hz), 126.9 (d, *J* = 7.4 Hz), 119.3, 115.8 (d, *J* = 21.7 Hz), 113.4, 50.3, 47.0, 39.7, 28.0. **HRMS** (ESI) calcd for $C_{27}H_{28}FN_4O$ [(M + H)$^+$], 443.2242; found, 443.2258.

***N*-(3-(((2-(3-(Aminomethyl)-4'-fluoro-[1,1'-biphenyl]-4-yl)ethyl)amino)methyl)phenyl)furan-3-carboximidamide trihydrochloride salt (14)**

Compound **14** was prepared by following General Procedure 4 via coupling of **2e** (26 mg, 0.9695 mmol) and **64** (35 mg, 0.0538 mmol). Purification by flash column chromatography using 80% EtOAc in hexane followed by salt formation as described in General Procedure 4, yielded **14** in the trihydrochloride salt form as a yellow solid (20 mg, 67%).^{1}**H NMR** (500 MHz, D_2O) δ 8.39 (s, 1H), 7.78 (s, 1H), 7.74 – 7.66 (m, 5H), 7.63 (d, *J* = 7.8 Hz, 1H), 7.57 (d, *J* = 8.0 Hz, 1H), 7.50 – 7.43 (m, 2H), 7.29 – 7.21 (m, 2H), 6.96 (s, 1H), 4.39 (s, 2H), 4.35 (s, 2H), 3.42 (t, *J* = 8.0 Hz, 2H), 3.22 (t, *J* = 7.9 Hz, 2H).^{13}**C NMR** (125 MHz, D_2O) δ 163.5, 161.5, 157.8, 147.1, 145.6, 139.6, 135.4 (d, *J* = 3.0 Hz), 134.0, 132.8, 131.6, 131.3, 130.9, 130.5, 128.6 (d, *J* = 8.4 Hz), 128.0 (d, *J* = 5.1 Hz), 127.0, 115.8 (d, *J* = 21.7 Hz), 108.1, 50.4, 47.1, 39.7, 28.0. **HRMS** (ESI) calcd for $C_{27}H_{28}FN_4O$ [(M + H)$^+$], 443.2242; found, 443.22570.

***N*-(3-(((2-(3-(Aminomethyl)-3'-fluoro-[1,1'-biphenyl]-4-yl)ethyl)amino)methyl)phenyl)furan-2-carboximidamide trihydrochloride salt (15)**

Compound **15** was prepared by following General Procedure 4 via coupling of **2b** (41 mg, 0.1539 mmol) and **62** (50 mg, 0.0769 mmol). Purification by flash column chromatography using 80% EtOAc in hexane followed by salt formation as described in General Procedure 4, gave **15** in the trihydrochloride salt form as a pale yellow solid (24 mg, 56%).^{1}**H NMR** (500 MHz, D_2O) δ 7.94 (s, 1H), 7.76 – 7.67 (m, 3H), 7.64 (d, *J* = 7.8 Hz, 1H), 7.60 – 7.45 (m, 2H), 7.49 (q, *J* = 7.2 Hz, 3H), 7.44 – 7.38 (m, 2H), 7.16 (tt, *J* = 8.7, 3.8 Hz, 1H), 6.81 (t, *J* = 2.7 Hz, 1H), 4.39 (s, 2H), 4.35

(s, 2H), 3.42 (t, *J* = 7.9 Hz, 2H), 3.23 (t, *J* = 8.0 Hz, 2H).^{13}C NMR (125 MHz, D_2O) δ 163.9, 162.0, 152.3, 149.0, 141.4 (d, *J* = 7.8 Hz), 140.4, 139.2 (d, *J* = 2.3 Hz), 134.7, 133.7, 132.7, 131.7, 131.3, 130.9, 130.8 (d, *J* = 8.6 Hz), 130.5, 128.1 (d, *J* = 1.7 Hz), 126.9 (d, *J* = 4.8 Hz), 122.5 (d, *J* = 2.6 Hz), 119.3, 114.7, 114.5, 113.5, 113.3 (d, *J* = 7.3 Hz), 50.4, 47.1, 39.7, 28.1. **HRMS** (ESI) calcd for $C_{27}H_{28}FN_4O$ [(M + H)$^+$], 443.2242; found, 443.2252.

***N*-(3-(((2-(3-(Aminomethyl)-2'-fluoro-[1,1'-biphenyl]-4-yl)ethyl)amino)methyl)phenyl)furan-2-carboximidamide trihydrochloride salt (16)**

Compound **16** was prepared by following General Procedure 4 via coupling of **2b** (37 mg, 0.1385 mmol) and **62** (45 mg, 0.0692 mmol). Purification by flash column chromatography using 70% EtOAc in hexane followed by salt formation as described in General Procedure 4, gave **16** in the trihydrochloride salt form as a pale yellow solid (24 mg, 63%).**^{1}H NMR** (500 MHz, D_2O) δ 7.93 (s, 1H), 7.73 – 7.66 (m, 3H), 7.64 – 7.53 (m, 4H), 7.51 – 7.43 (m, 2H), 7.40 (s, 1H), 7.34 (t, *J* = 7.5 Hz, 1H), 7.28 (dd, *J* = 11.3, 8.3 Hz, 1H), 6.84 – 6.79 (m, 1H), 4.37 (s, 2H), 4.34 (s, 2H), 3.42 (t, *J* = 7.8 Hz, 2H), 3.23 (t, *J* = 7.9 Hz, 2H). ^{13}C NMR (125 MHz, D_2O) δ 160.3, 158.3, 152.4, 148.9, 140.6, 135.5, 134.5, 132.8, 131.3 (d, *J* = 10.7 Hz), 130.5, 130.5 (d, *J* = 3.5 Hz), 130.3, 130.2, 130.0 (d, *J* = 7.8 Hz), 127.1, 127.0, 126.9 (d, *J* = 12.5 Hz), 124.8 (d, *J* = 4.1 Hz), 119.1, 116.1 (d, *J* = 22.5 Hz), 113.3, 50.4, 47.0, 39.6, 28.1. **HRMS** (ESI) calcd for $C_{27}H_{28}FN_4O$ [(M + H)$^+$], 443.2242; found, 443.2255.

***N*-(3-(((2-(3-(Aminomethyl)-3',5'-difluoro-[1,1'-biphenyl]-4-yl)ethyl)amino)methyl)phenyl)furan-2-carboximidamide trihydrochloride salt (17)**

Compound **17** was prepared by following General Procedure 4 via coupling of **2b** (30 mg, 0.1124 mmol) and **65** (50 mg, 0.0749 mmol). Purification by flash column chromatography using 80% EtOAc in hexane followed by salt formation as described in General Procedure 4, produced **17** in the trihydrochloride salt form as a pale yellow solid (26 mg, 60%).**^{1}H NMR** (500 MHz, D_2O) δ 7.90 (s, 1H), 7.71 – 7.59 (m, 4H), 7.58 – 7.50 (m, 2H), 7.45 (d, *J* = 8.0 Hz, 1H), 7.40 (d, *J* = 1.9 Hz, 1H), 7.25 – 7.17 (m, 2H), 6.91 (td, *J* = 9.2, 2.1 Hz, 1H), 6.80 – 6.76 (m, 1H), 4.37 (s, 2H), 4.32 (s, 2H), 3.40 (t, *J* = 8.0 Hz, 2H), 3.21 (t, *J* = 8.0 Hz, 2H). ^{13}C NMR (125 MHz, D_2O) δ 164.0 (d, *J* = 13.5 Hz), 162.0 (d, *J* = 13.5 Hz), 152.2, 149.0, 142.4 (t, *J* = 9.8 Hz), 140.3, 138.1, 135.2, 133.6, 132.6, 131.7, 131.3, 131.0, 130.5, 128.1 (d, *J* = 5.1 Hz), 126.9 (d, *J* = 2.0 Hz), 119.3, 113.3, 109.6

(d, J = 6.3 Hz), 109.5 (d, J = 6.3 Hz), 102.8 (t, J = 25.9 Hz), 50.3, 47.0, 39.7, 28.1. **HRMS** (ESI) calcd for $C_{27}H_{27}F_2N_4O$ [$(M + H)^+$], 461.2147; found, 461.2161

***N*-(3-(((2-(3-((Methylamino)methyl)-[1,1'-biphenyl]-4-yl)ethyl)amino)methyl)phenyl)furan-2-carboximidamide trihydrochloride salt (18)**

Compound **18** was prepared by following General Procedure 4 via coupling of **2b** (40 mg, 0.1467 mmol) and **71** (40 mg, 0.0733 mmol). Purification by flash column chromatography using 5% methanol in dichloromethane followed by salt formation as described in General Procedure 4, gave **18** in the trihydrochloride salt form as a yellow solid (21 mg, 52%). **^{1}H NMR** (500 MHz, D_2O) δ 7.94 (s, 1H), 7.81 – 7.76 (m, 2H), 7.71 (dd, J = 11.8, 7.7 Hz, 3H), 7.62 (d, J = 7.8 Hz, 1H), 7.57 – 7.45 (m, 6H), 7.43 (d, J = 2.6 Hz, 1H), 6.82 (t, J = 2.8 Hz, 1H), 4.38 - 4.37 (m, 4H), 3.41 (t, J = 7.8 Hz, 2H), 3.24 (t, J = 7.9 Hz, 2H), 2.82 (s, 3H). **^{13}C NMR** (125 MHz, D_2O) δ 152.4, 149.0, 140.5, 140.4, 139.0, 134.6, 133.8, 132.7, 131.3, 131.0, 130.4, 129.9, 129.2, 129.1, 128.6, 128.2, 127.0, 126.9, 126.7, 119.3, 113.4, 50.3, 48.9, 47.2, 32.4, 28.1. **HRMS** (ESI) calcd for $C_{28}H_{31}N_4O$ [$(M + H)^+$], 439.2492; found, 439.2504.

***N*-(3-(((2-(3-((Methylamino)methyl)-[1,1'-biphenyl]-4-yl)ethyl)amino)methyl)phenyl)furan-3-carboximidamide trihydrochloride salt (19)**

Compound **19** was prepared by following General Procedure 4 via coupling of **2e** (40 mg, 0.1467 mmol) and **71** (40 mg, 0.0733 mmol). Purification by flash column chromatography using 60% EtOAc in hexane followed by salt formation as described in General Procedure 4, yielded **19** in the trihydrochloride salt form as a yellow solid (23 mg, 57%). **^{1}H NMR** (500 MHz, D_2O) δ 8.29 (s, 1H), 7.70 (d, J = 7.1 Hz, 3H), 7.63 (t, J = 7.7 Hz, 3H), 7.53 (d, J = 7.9 Hz, 1H), 7.47 (t, J = 7.7 Hz, 3H), 7.43 – 7.33 (m, 3H), 6.86 (s, 1H), 4.30 – 4.29 (m, 2H), 3.34 – 3.30 (m, 2H), 3.14 (t, J = 8.0 Hz, 2H), 2.74 (s, 3H). ^{13}C NMR (125 MHz, D_2O) δ 157.8, 155.6, 147.1, 145.6, 140.5, 139.1, 134.6, 134.0, 132.8, 131.3, 131.0, 130.5, 129.9, 129.2, 129.1, 128.6, 128.2, 127.0, 126.9, 126.8, 115.7, 108.0, 50.4, 48.9, 47.2, 32.4, 28.1. **HRMS** (ESI) calcd for $C_{28}H_{31}N_4O$ [$(M + H)^+$]; 439.2492. found, 439.2503

***N*-(3-(((2-(3-((Methylamino)methyl)-[1,1'-biphenyl]-4-yl)ethyl)amino)methyl)phenyl)thiophene-2-carboximidamide trihydrochloride salt (20)**

Compound **20** was prepared by following General Procedure 4 via coupling of **2a** (40 mg, 0.1467 mmol) and **71** (40 mg, 0.0733 mmol). Purification by flash column chromatography using 60% EtOAc in hexane followed by salt formation as described in General Procedure 4, gave **20** in the trihydrochloride salt form as a yellow solid (24 mg, 58%).**^{1}H NMR** (500 MHz, D_2O) δ 8.06 – 7.92 (m, 2H), 7.79 – 7.77 (m, 2H), 7.73 – 7.70 (m, 3H), 7.65 – 7.45 (m, 7H), 7.36 (s, 1H), 4.39 – 4.38 (m, 4H), 3.41 – 3.40 (m, 2H), 3.24 (t, J = 7.9 Hz, 2H), 2.83 (s, 3H).**^{13}C NMR** (125 MHz, D_2O) δ 158.1, 140.5, 139.1, 134.6, 134.5, 134.0, 132.8, 131.3, 131.0, 130.4, 130.0, 129.2, 129.1, 128.9, 128.6, 128.2, 126.9, 126.8, 50.4, 48.9, 47.2, 32.4, 28.1. **HRMS** (ESI) calcd for $C_{28}H_{31}N_4S$ [(M + H)$^+$], 455.2264; found, 455.2281.

***N*-(3-(((2-(3-((Methylamino)methyl)-[1,1'-biphenyl]-4-yl)ethyl)amino)methyl)phenyl)thiazole-5-carboximidamide trihydrochloride salt (21)**

Compound **21** was prepared by following General Procedure 4 via coupling of **2d** (40 mg, 0.1467 mmol) and **71** (40 mg, 0.0733 mmol). Purification by flash column chromatography using 60% EtOAc in hexane followed by salt formation as described in General Procedure 4, produced **21** in the trihydrochloride salt form as a yellow solid (22 mg, 53%).**^{1}H NMR** (500 MHz, D_2O) δ 9.40 (s, 1H), 8.63 (s, 1H), 7.82 – 7.76 (m, 2H), 7.73 – 7.69 (m, 3H), 7.63 (d, J = 7.8 Hz, 1H), 7.59 – 7.46 (m, 5H), 7.42 (s, 1H), 4.39 – 4.35 (m, 4H), 3.42 – 3.40 (m, 2H), 3.24 (t, J = 7.9 Hz, 2H), 2.83 (s, 3H). **^{13}C NMR** (125 MHz, D_2O) δ 160.9, 156.4, 147.5, 140.6, 139.0, 134.6, 132.8, 131.4, 131.0, 130.5, 130.0, 129.2, 129.1, 128.6, 128.2, 126.8, 126.7, 126.6, 50.3, 48.9, 47.1, 32.4, 28.1. **HRMS** (ESI) calcd for $C_{27}H_{30}N_5S$ [(M + H)$^+$], 456.2216; found, 456.2224.

12. Copies of ^{1}H NMR and ^{13}C NMR of Target Compounds 1-21

Compound 1

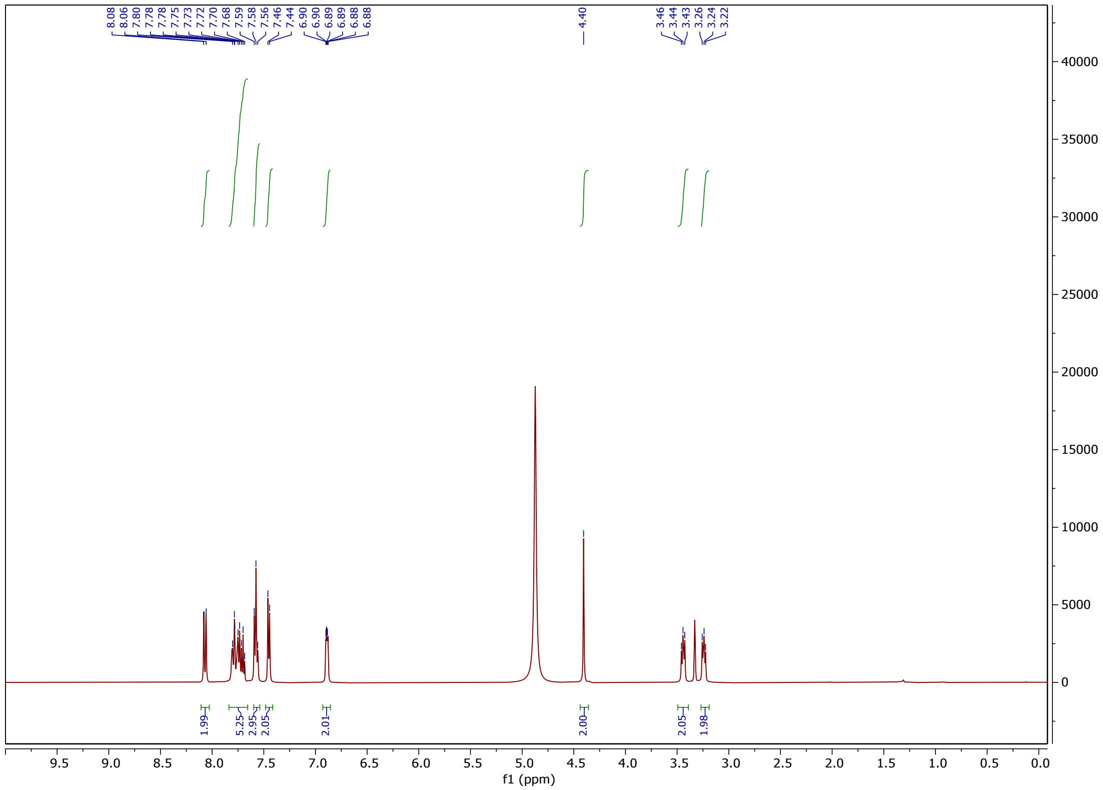

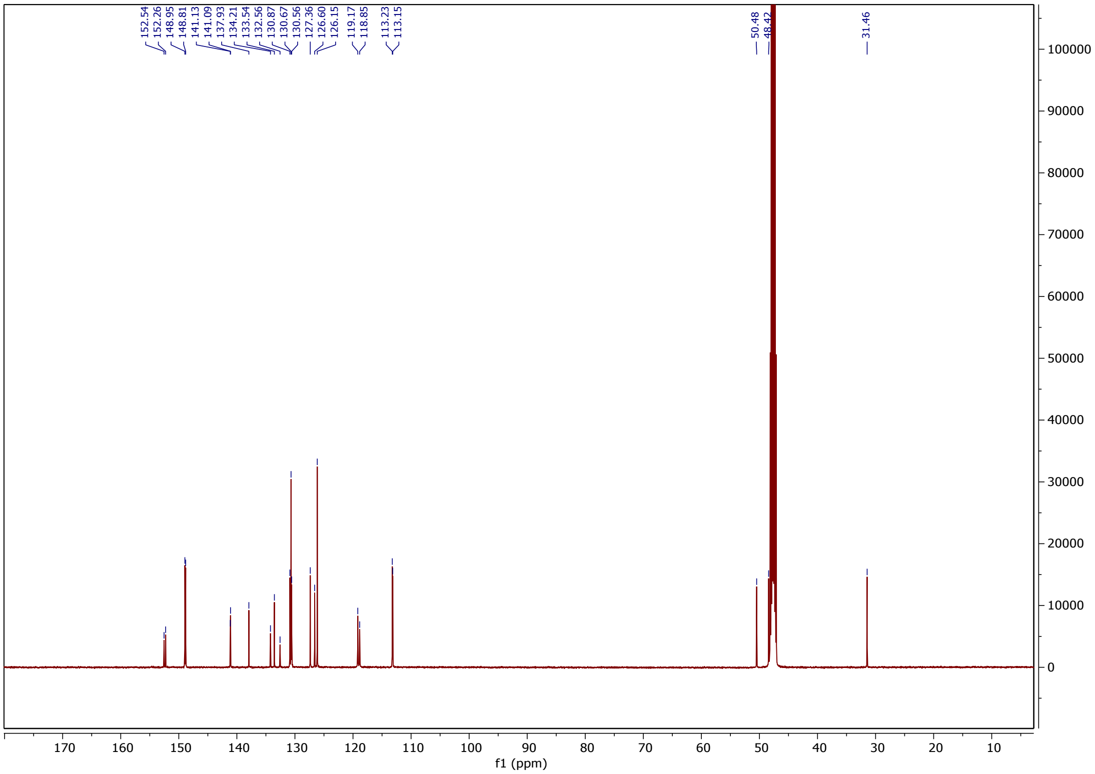

Compound 2

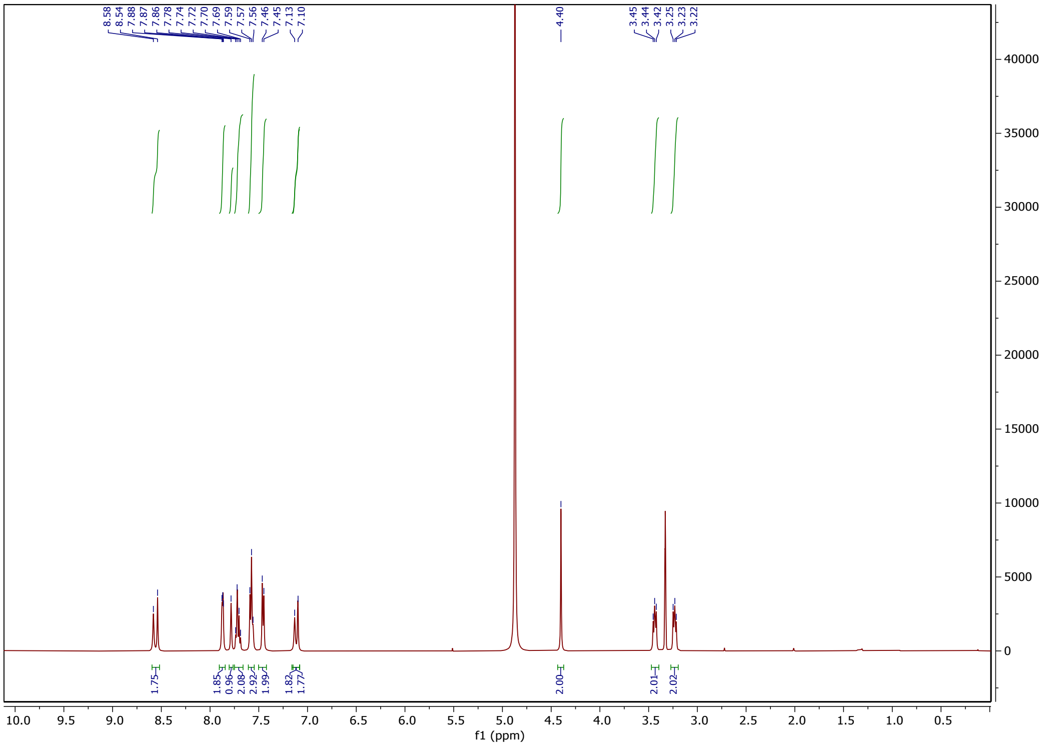
8.58
8.54
7.88
7.87
7.86
7.78
7.74
7.72
7.70
7.69
7.59
7.57
7.56
7.46
7.45
7.13
7.10
4.40
3.45
3.44
3.42
3.25
3.23
3.22
1.75
1.85
0.96
2.08
2.92
1.99
1.82
1.77
2.00
2.01
2.02
f1 (ppm)

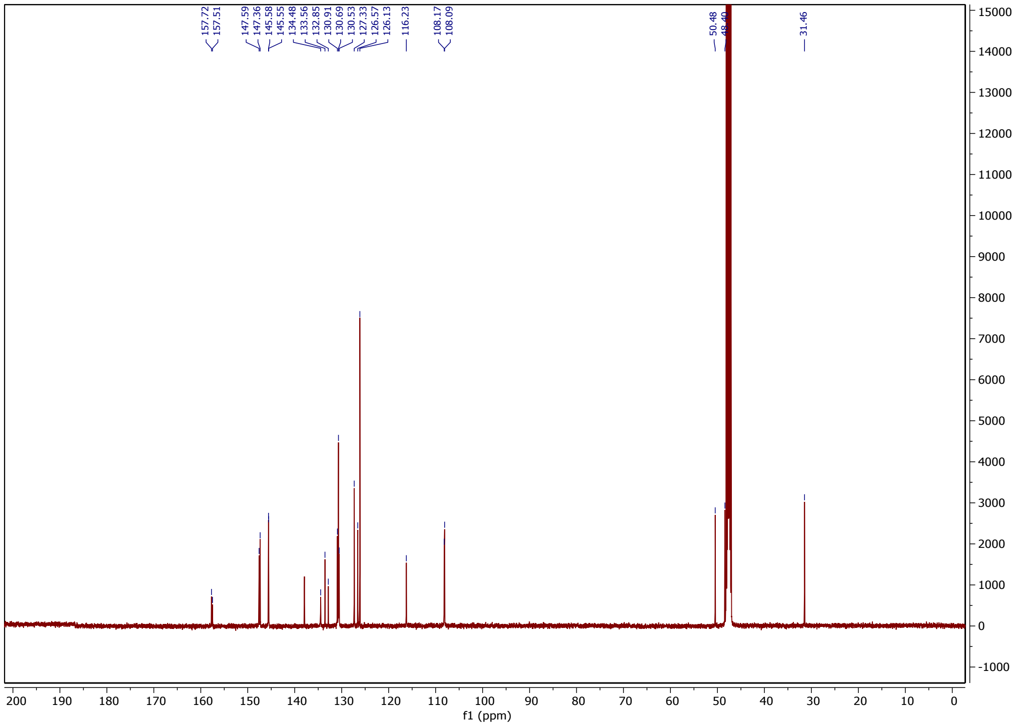
157.72
157.51
147.59
147.36
145.58
145.55
134.48
133.56
132.85
130.91
130.69
130.53
127.33
126.57
126.13
116.23
108.17
108.09
50.48
31.46
f1 (ppm)

Compound 3

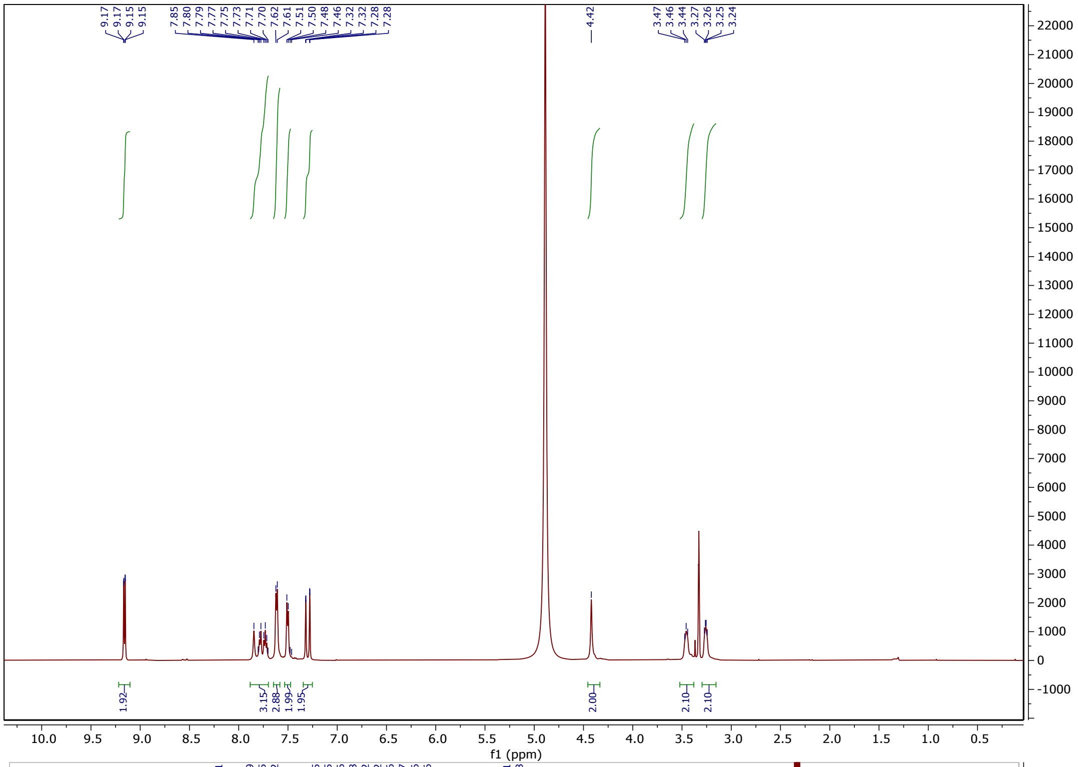
9.17
9.17
9.15
9.15
7.85
7.80
7.79
7.77
7.75
7.73
7.71
7.70
7.62
7.61
7.51
7.50
7.48
7.46
7.32
7.32
7.28
7.28
4.42
3.47
3.46
3.44
3.27
3.26
3.25
3.24
1.92
3.15
2.88
1.99
1.95
2.00
2.10
2.10
f1 (ppm)

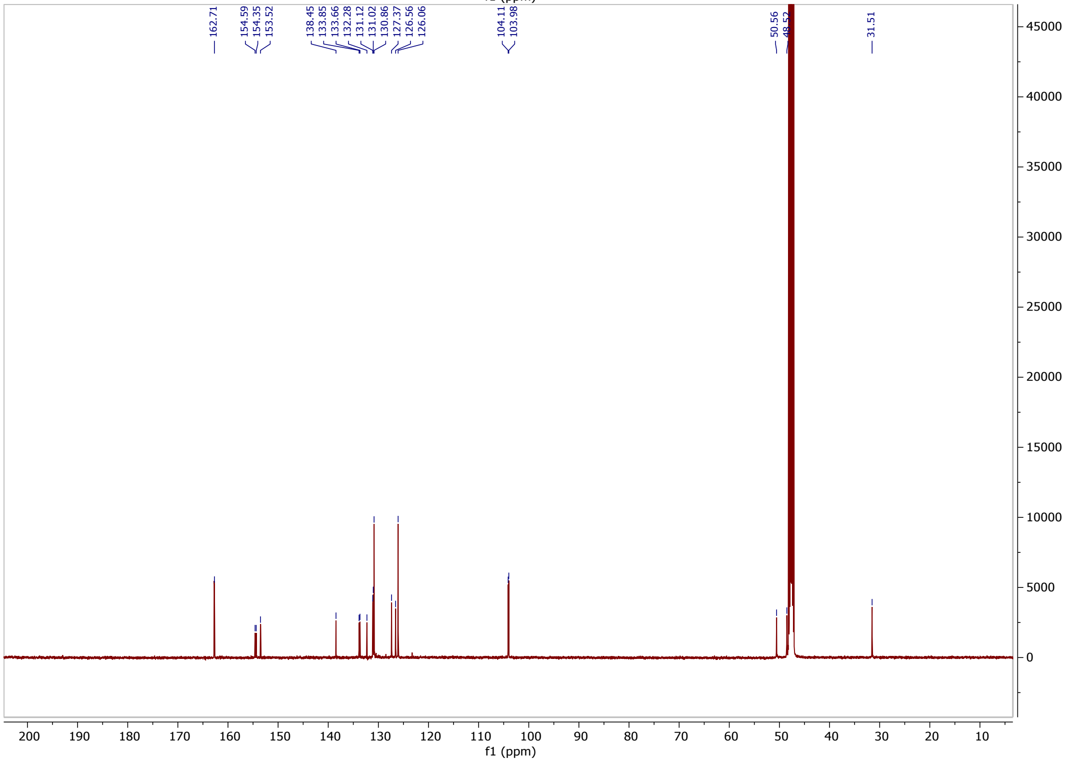
162.71
154.59
154.35
153.52
138.45
133.85
133.66
132.28
131.12
131.02
130.86
127.37
126.56
126.06
104.11
103.98
50.56
48.52
31.51
f1 (ppm)

Compound 4

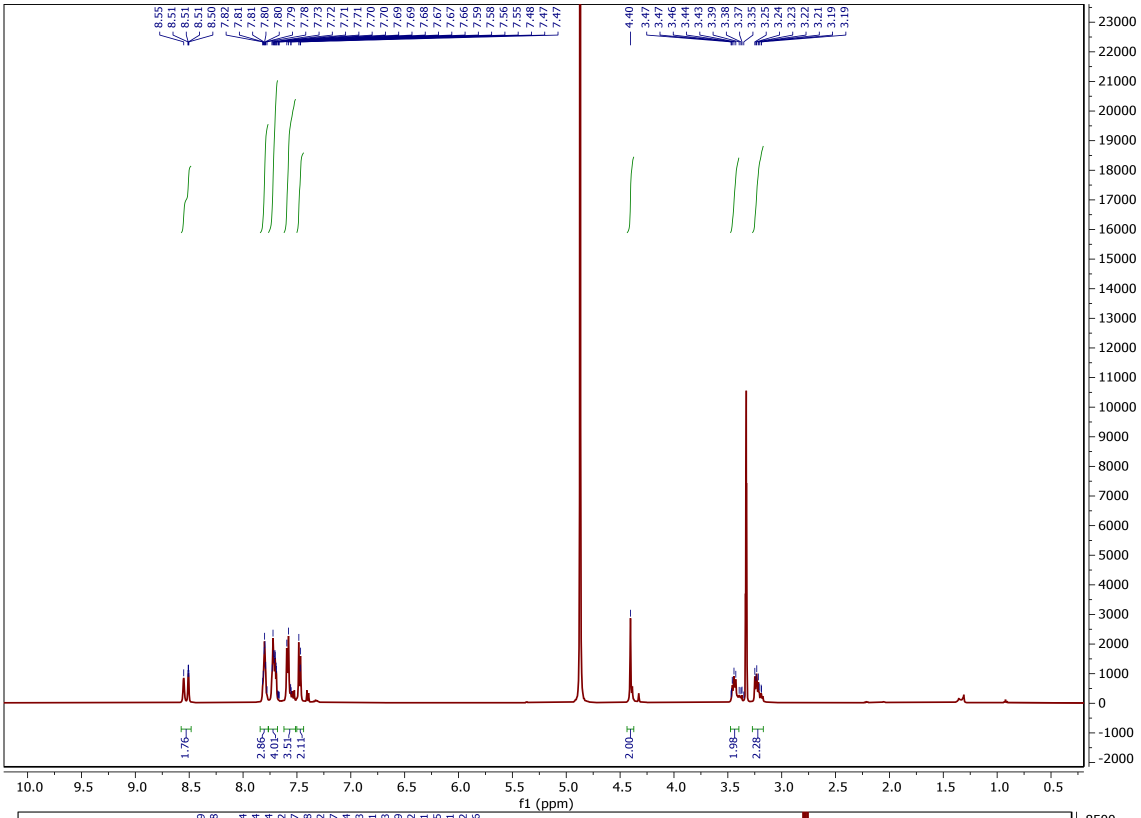

8.55
8.51
8.51
8.51
8.50
7.82
7.81
7.81
7.80
7.80
7.79
7.78
7.73
7.72
7.71
7.71
7.70
7.70
7.69
7.69
7.68
7.67
7.67
7.66
7.59
7.58
7.56
7.55
7.48
7.47
7.47
4.40
3.47
3.47
3.46
3.44
3.43
3.39
3.38
3.37
3.35
3.25
3.24
3.23
3.22
3.21
3.19
3.19
1.76
2.86
4.01
3.51
2.11
2.00
1.98
2.28
f1 (ppm)

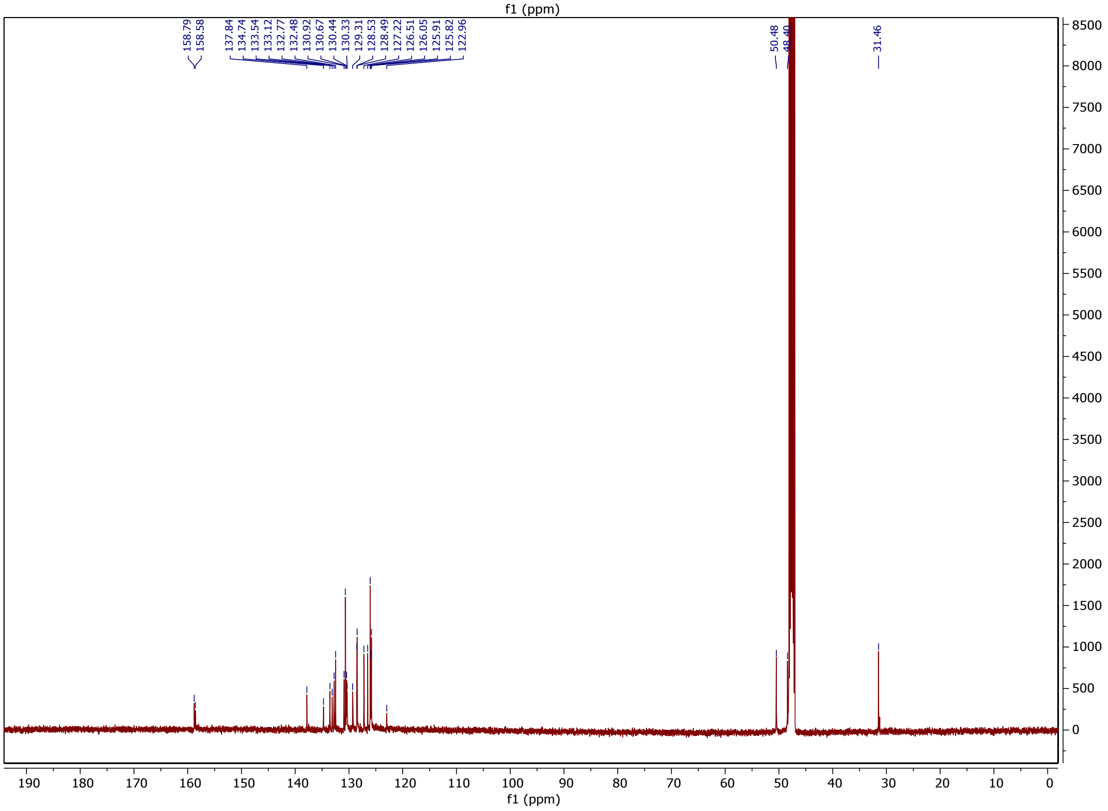

158.79
158.58
137.84
134.74
133.54
133.12
132.77
132.48
130.92
130.67
130.44
130.33
129.31
128.53
128.49
127.22
126.51
126.05
125.91
125.82
122.96
50.48
48.40
31.46
f1 (ppm)

Compound 5

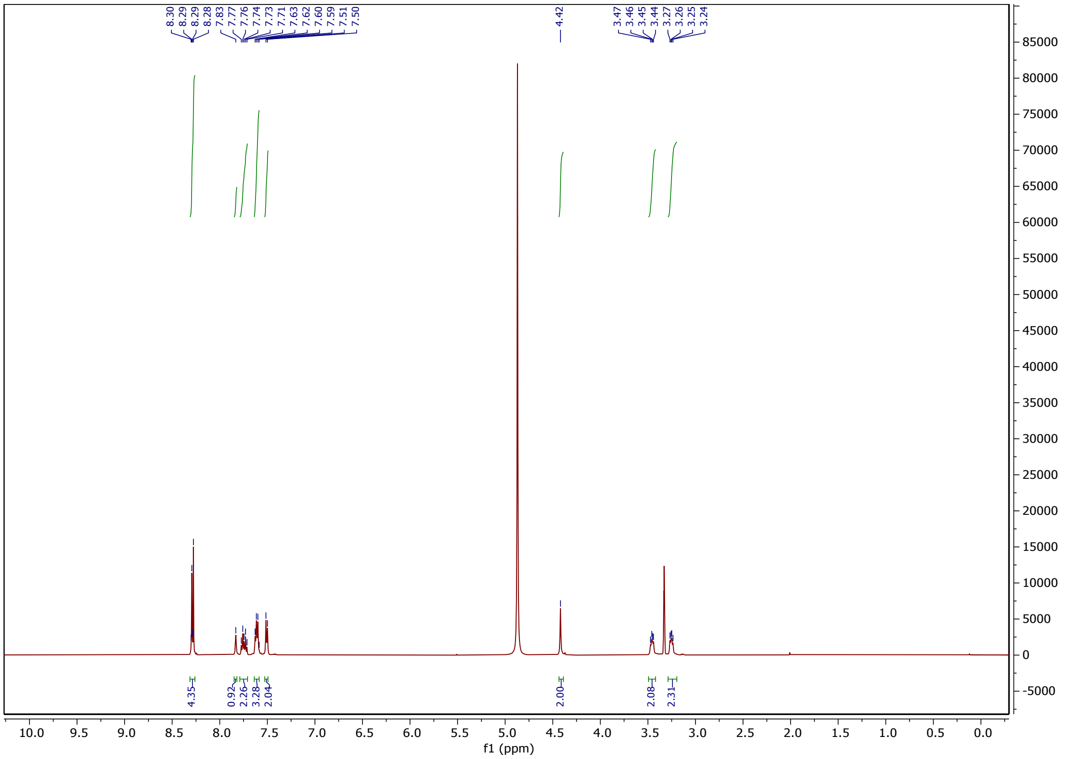
8.30
8.29
8.29
8.28
7.83
7.77
7.76
7.74
7.73
7.71
7.63
7.62
7.60
7.59
7.51
7.50
4.42
3.47
3.46
3.45
3.44
3.27
3.26
3.25
3.24
4.35
0.92
2.26
3.28
2.04
2.00
2.08
2.31
f1 (ppm)

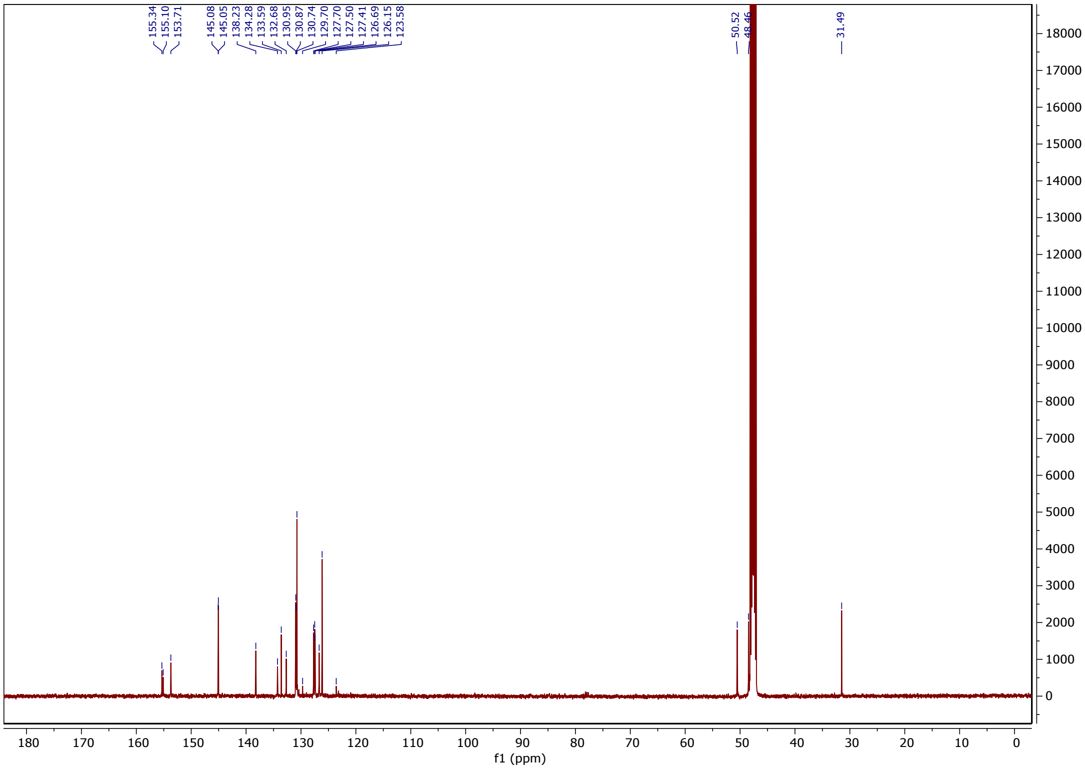
155.34
155.10
153.71
145.08
145.05
138.23
134.28
133.59
132.68
130.95
130.87
130.74
129.70
127.70
127.50
127.41
126.69
126.15
123.58
50.52
48.46
31.49
f1 (ppm)

Compound 6

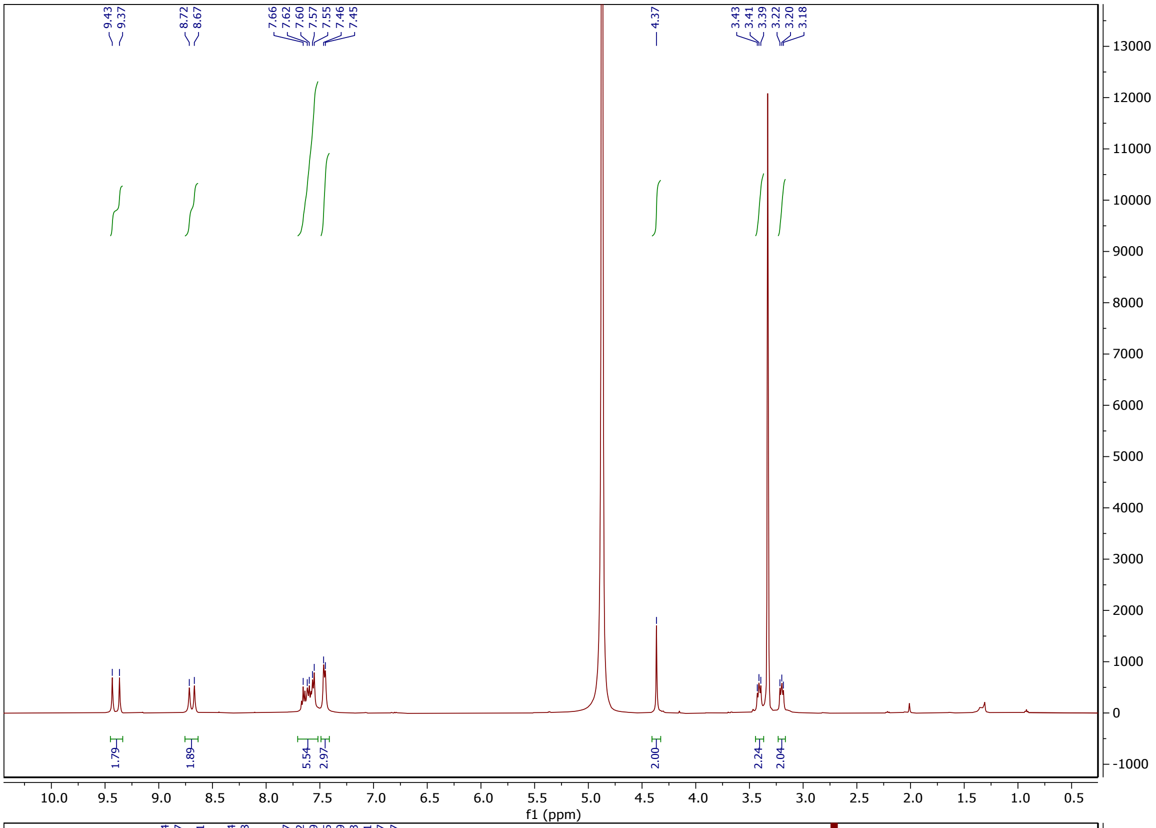

9.43
9.37
8.72
8.67
7.66
7.62
7.60
7.57
7.55
7.46
7.45
4.37
3.43
3.41
3.39
3.22
3.20
3.18
1.79
1.89
5.54
2.97
2.00
2.24
2.04
f1 (ppm)

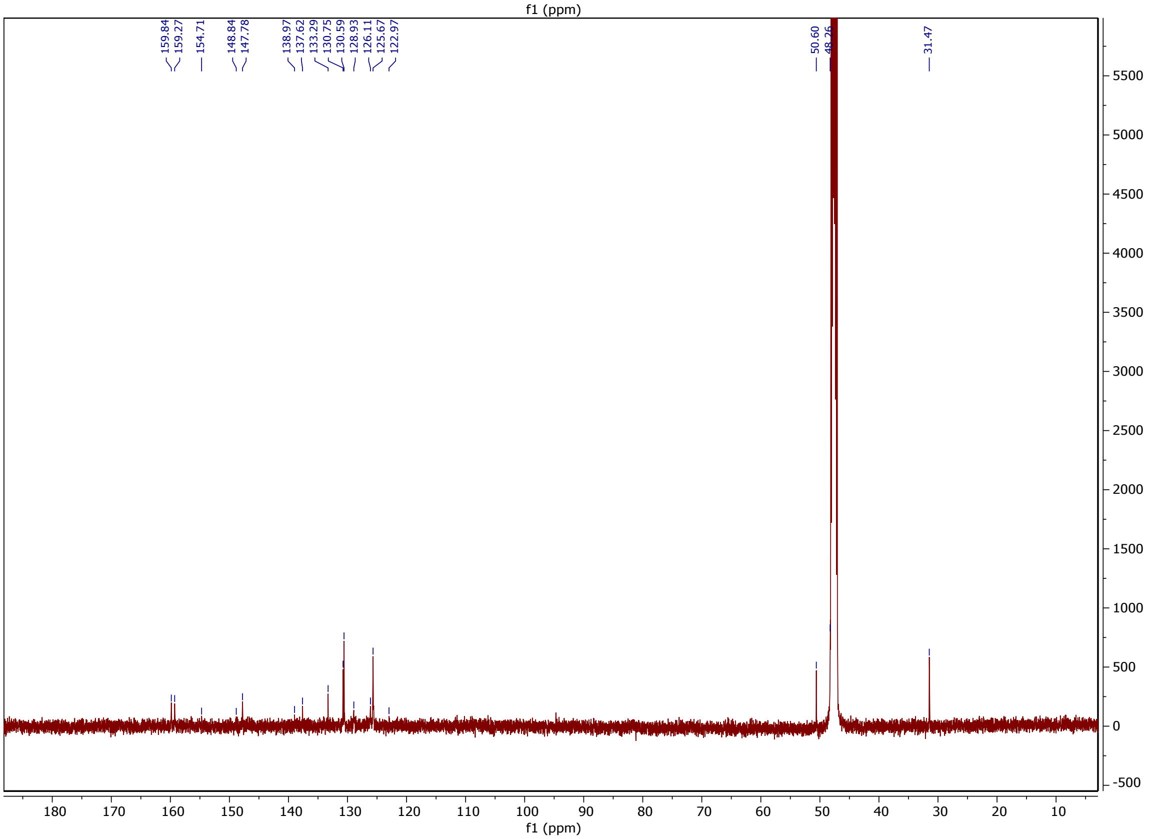

159.84
159.27
154.71
148.84
147.78
138.97
137.62
133.29
130.75
130.59
128.93
126.11
125.67
122.97
50.60
48.26
31.47
f1 (ppm)

Compound 7

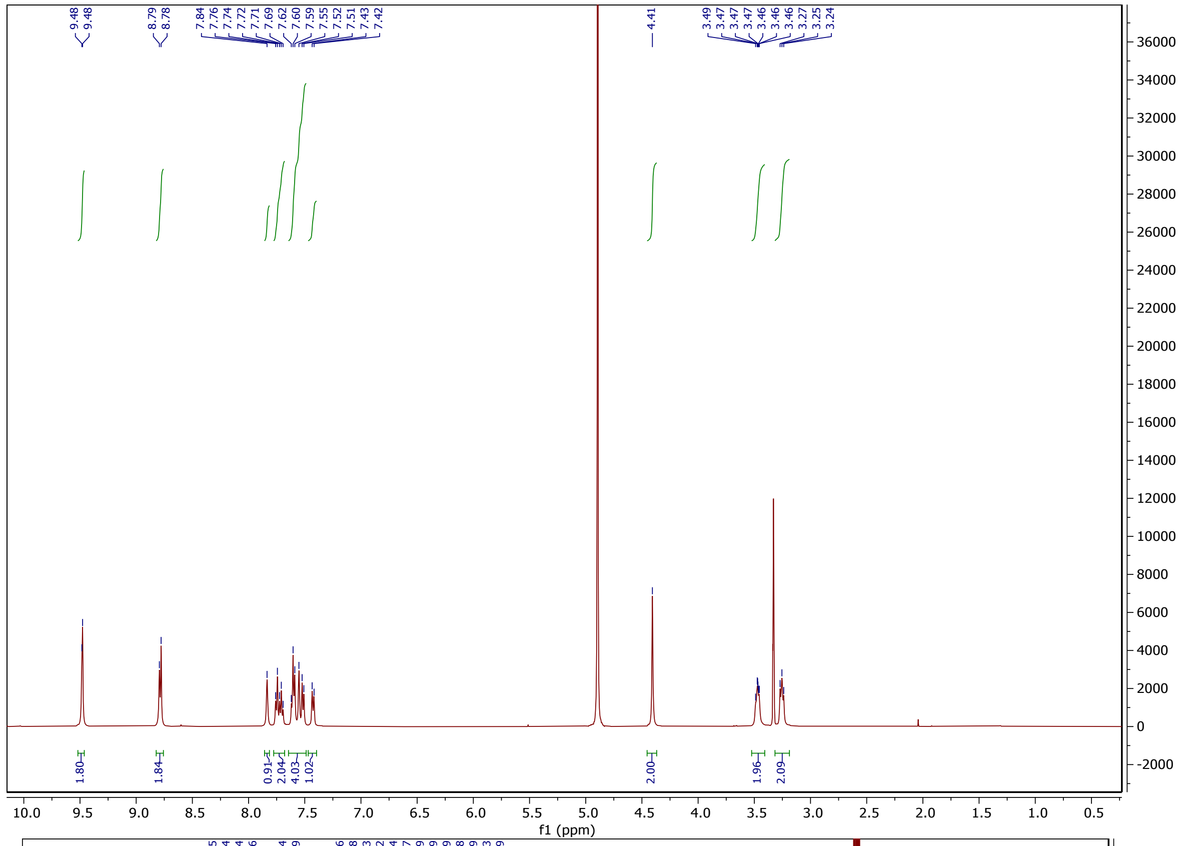
9.48
9.48
8.79
8.78
7.84
7.76
7.74
7.72
7.71
7.69
7.62
7.60
7.59
7.55
7.52
7.51
7.43
7.42
4.41
3.49
3.47
3.47
3.47
3.46
3.46
3.46
3.27
3.25
3.24
1.80
1.84
0.91
2.04
4.03
1.02
2.00
1.96
2.09
f1 (ppm)

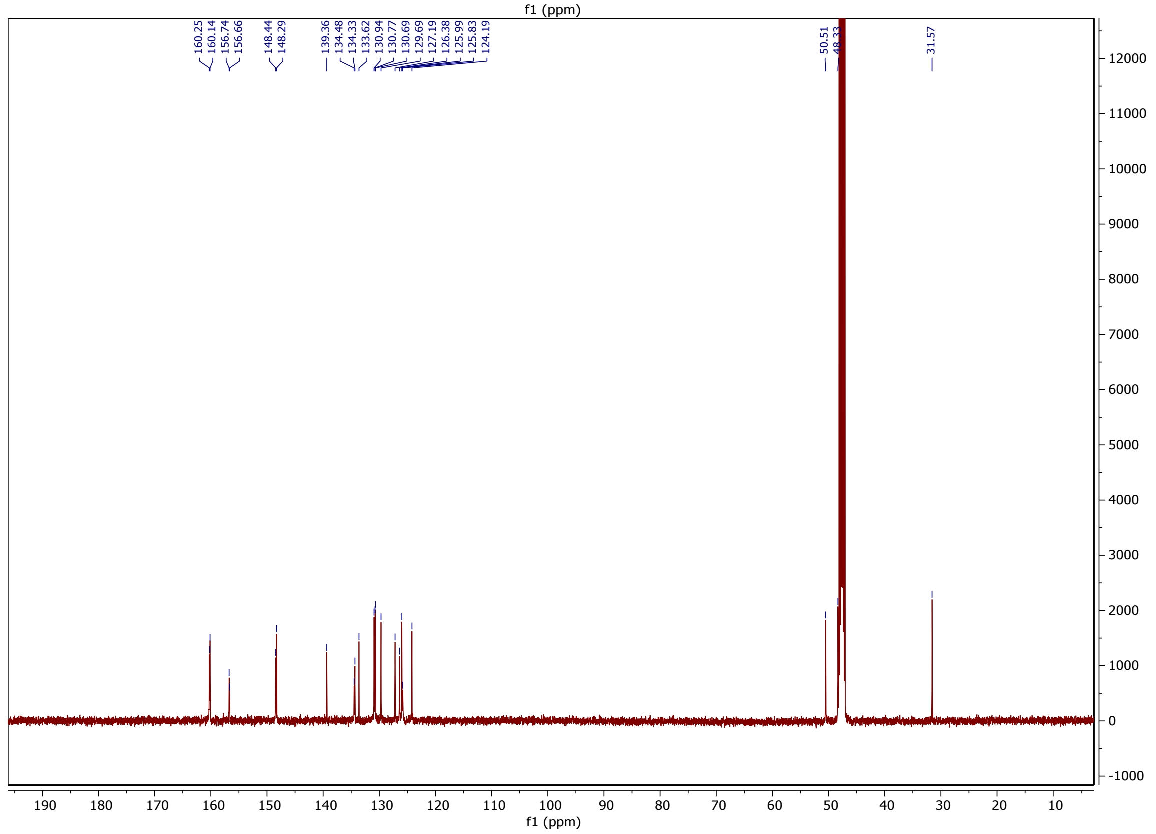
160.25
160.14
156.74
156.66
148.44
148.29
139.36
134.48
134.33
133.62
130.94
130.77
130.69
129.69
127.19
126.38
125.99
125.83
124.19
50.51
48.33
31.57
f1 (ppm)

Compound 8

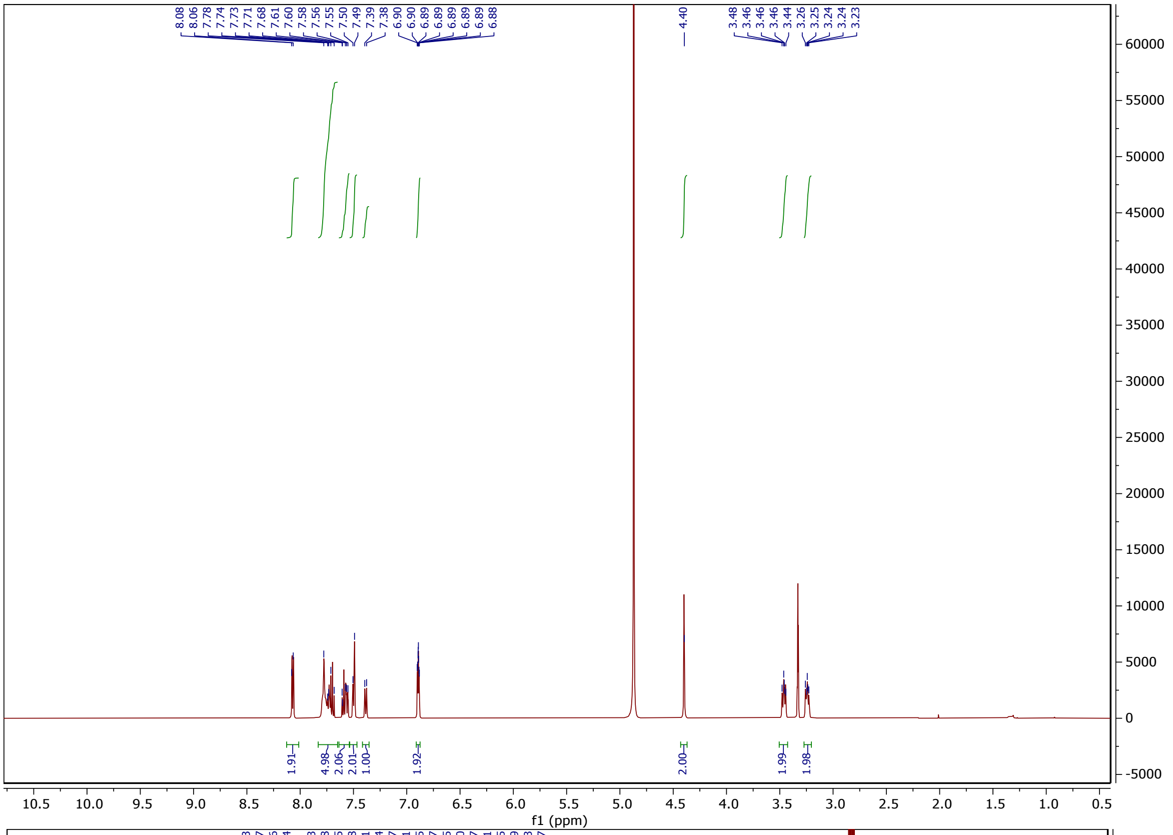
8.08
8.06
7.78
7.74
7.73
7.71
7.68
7.61
7.60
7.58
7.56
7.55
7.50
7.49
7.39
7.38
6.90
6.90
6.89
6.89
6.89
6.89
6.89
6.88
4.40
3.48
3.46
3.46
3.44
3.26
3.25
3.24
3.23
1.91
4.98
2.06
2.01
1.00
1.92
2.00
1.99
1.98
f1 (ppm)

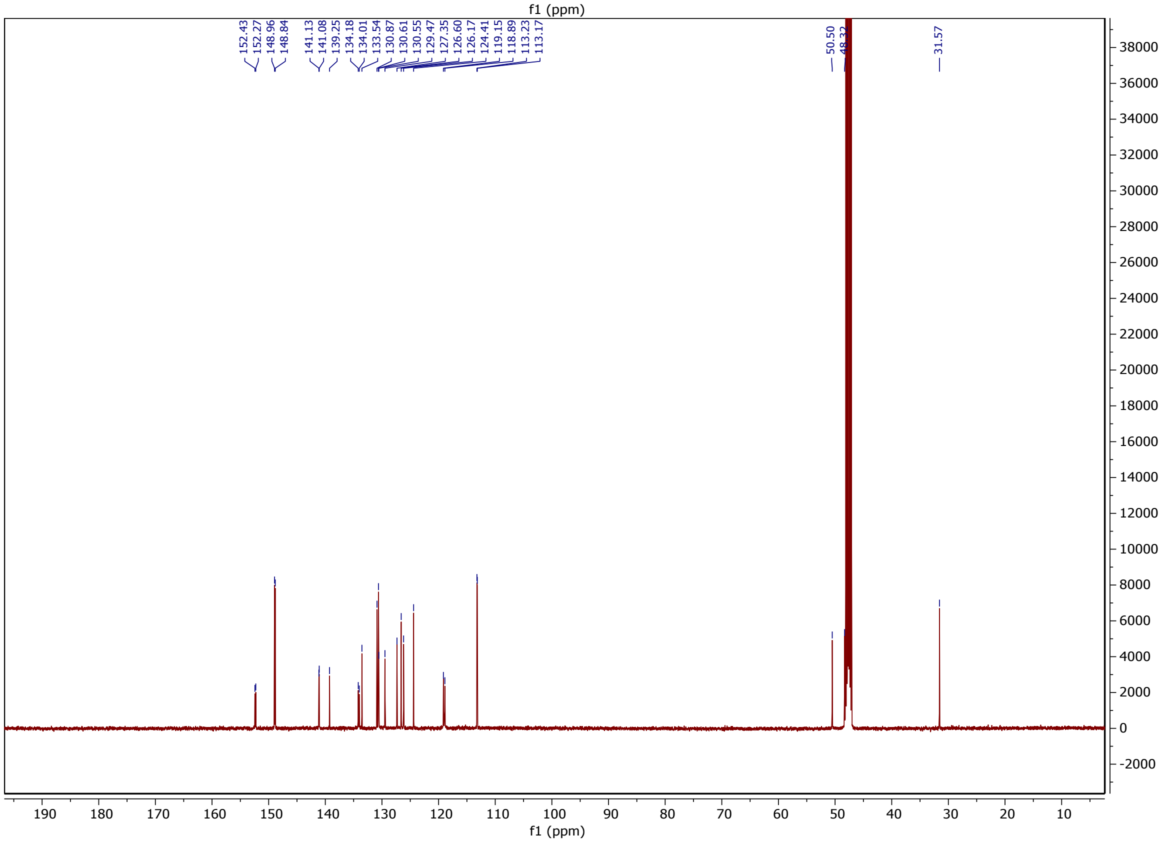
152.43
152.27
148.96
148.84
141.13
141.08
139.25
134.18
134.01
133.54
130.87
130.61
130.55
129.47
127.35
126.60
126.17
124.41
119.15
118.89
113.23
113.17
50.50
48.32
31.57
f1 (ppm)

Compound 9

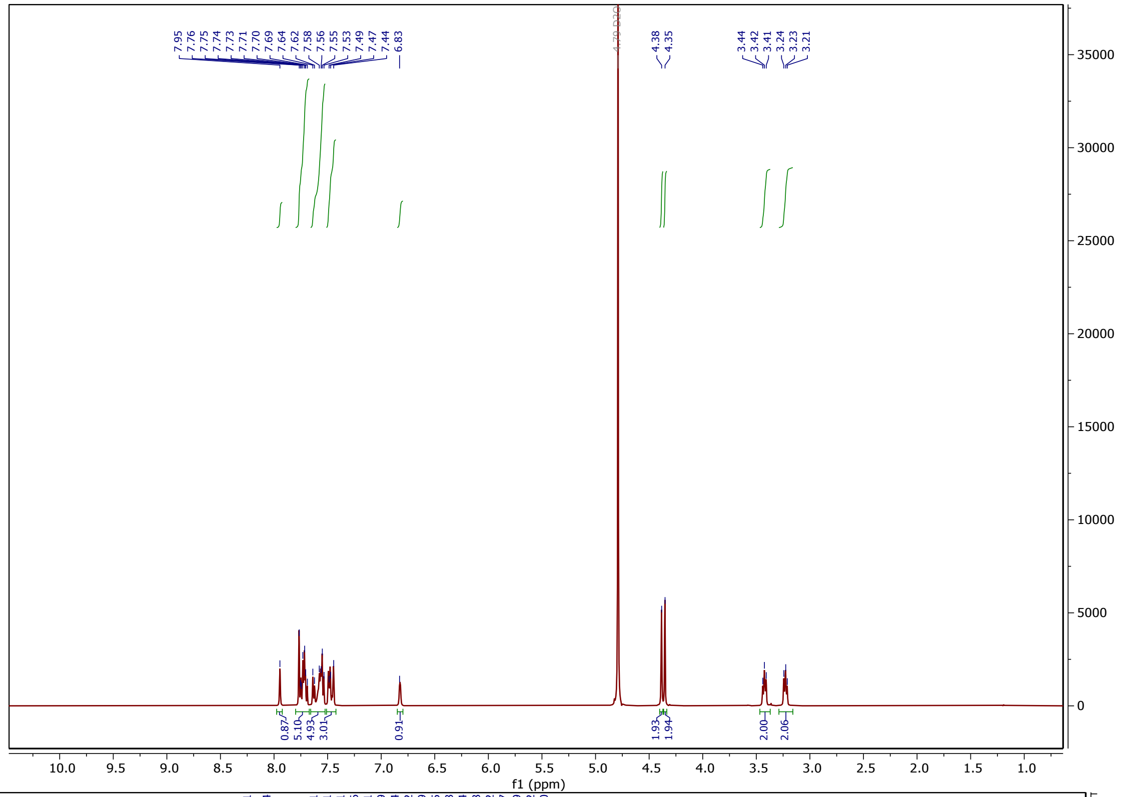
7.95
7.76
7.75
7.74
7.73
7.71
7.70
7.69
7.64
7.62
7.58
7.56
7.55
7.53
7.49
7.47
7.44
6.83
4.38
4.35
3.44
3.42
3.41
3.24
3.23
3.21
0.87
5.10
4.93
3.01
0.91
1.93
1.94
2.00
2.06
f1 (ppm)

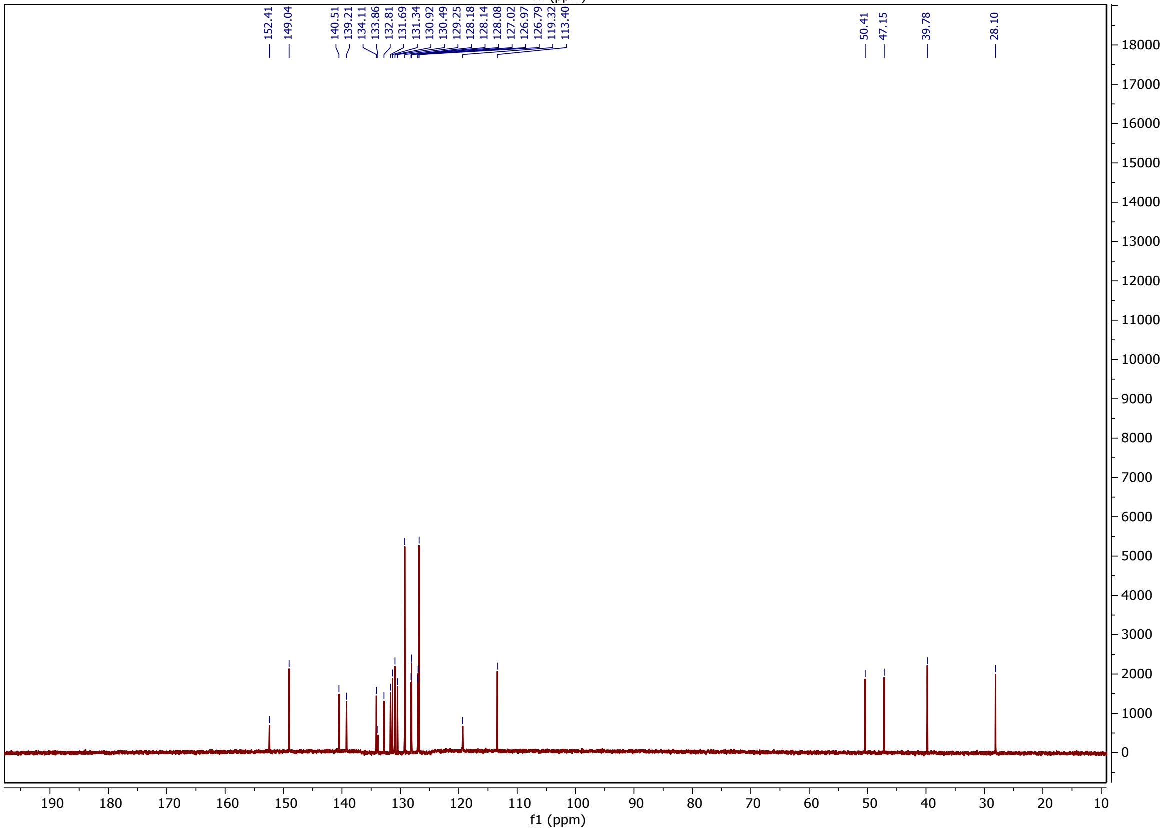
152.41
149.04
140.51
139.21
134.11
133.86
132.81
131.69
131.34
130.92
130.49
129.25
128.18
128.14
128.08
127.02
126.97
126.79
119.32
113.40
50.41
47.15
39.78
28.10
f1 (ppm)

Compound 10

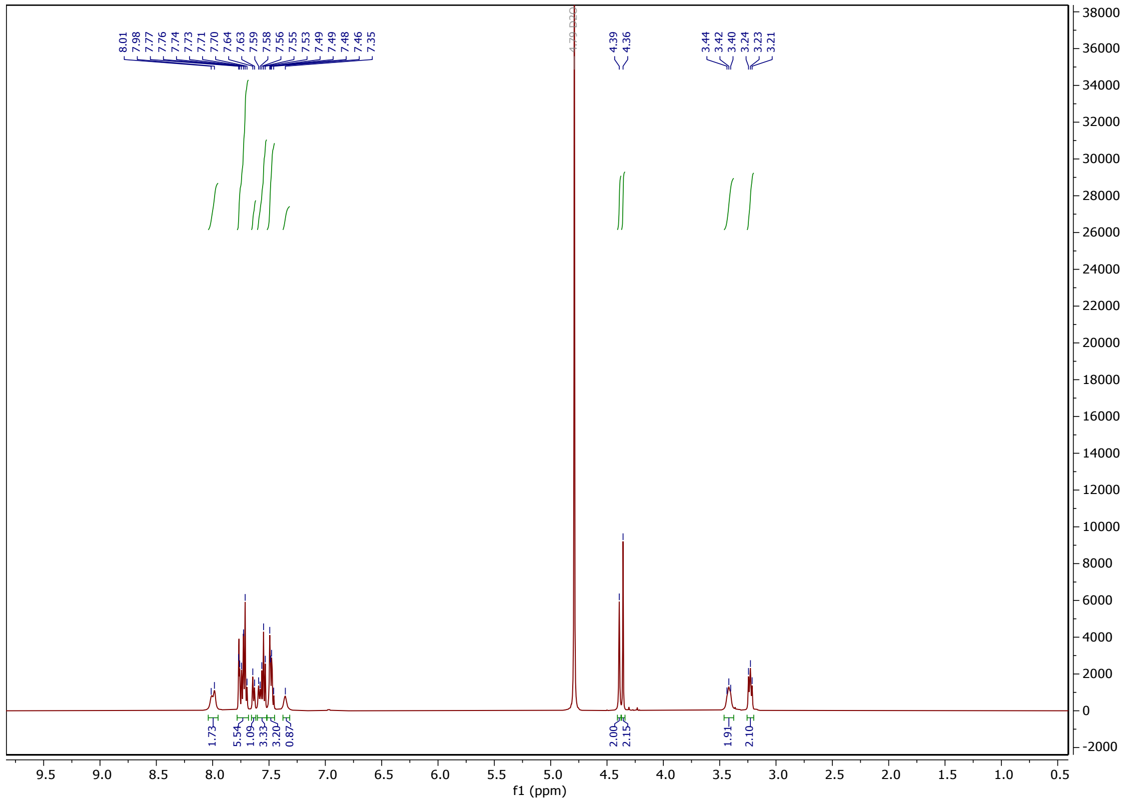

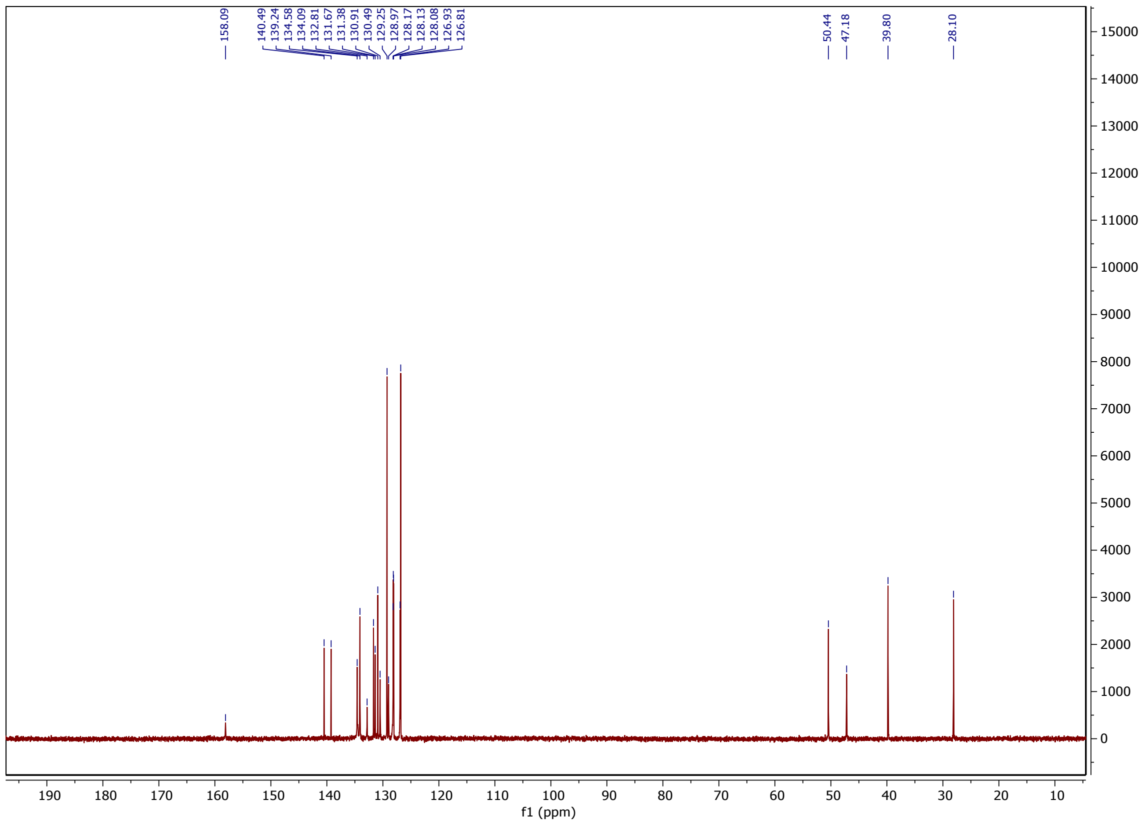

Compound 11

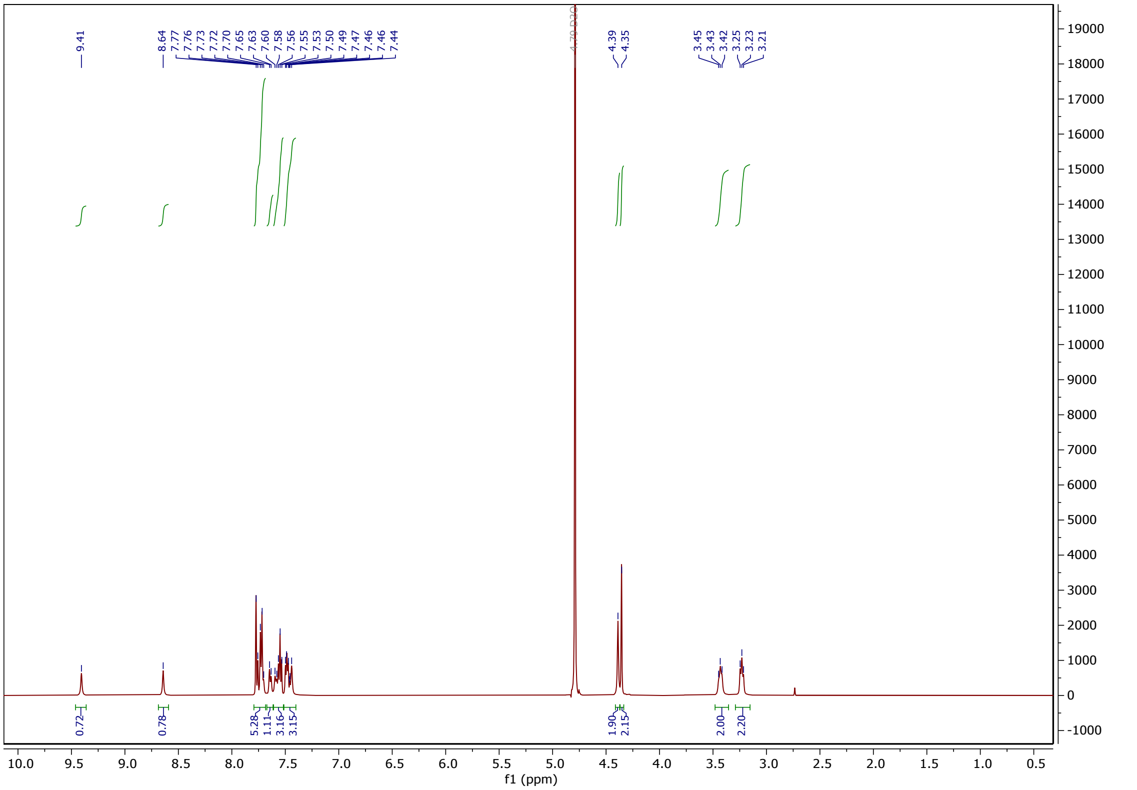

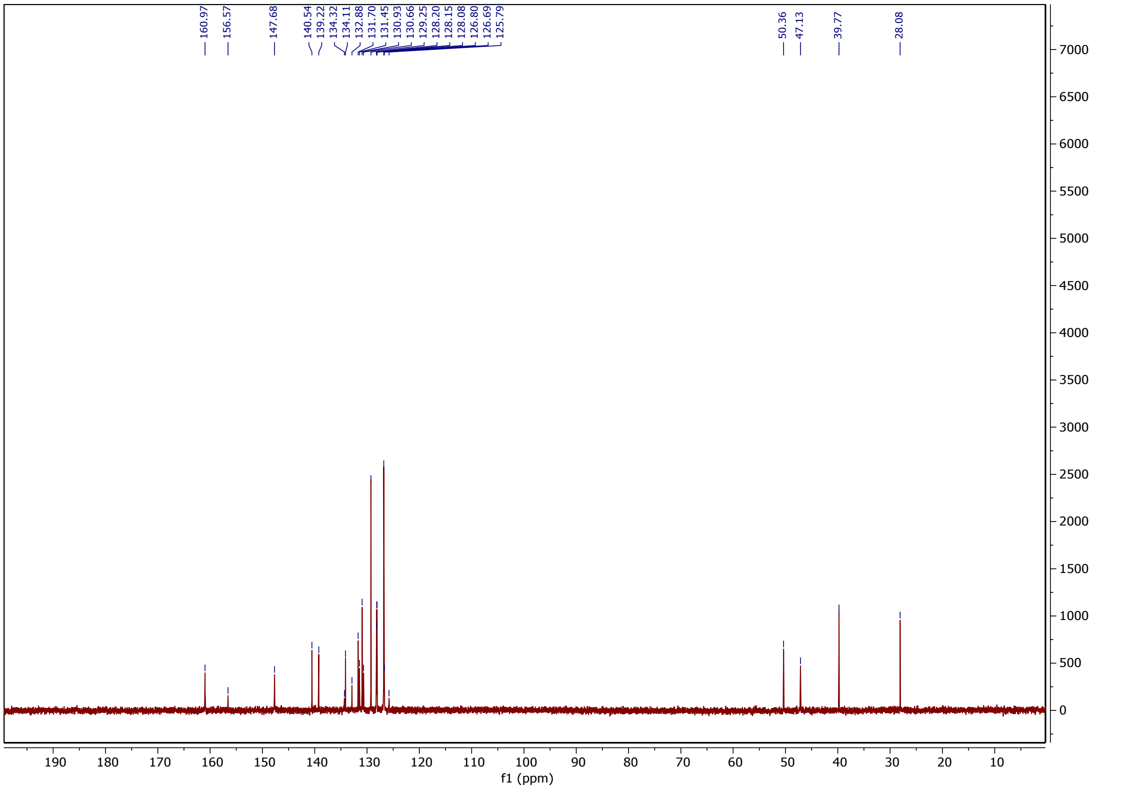

Compound 12

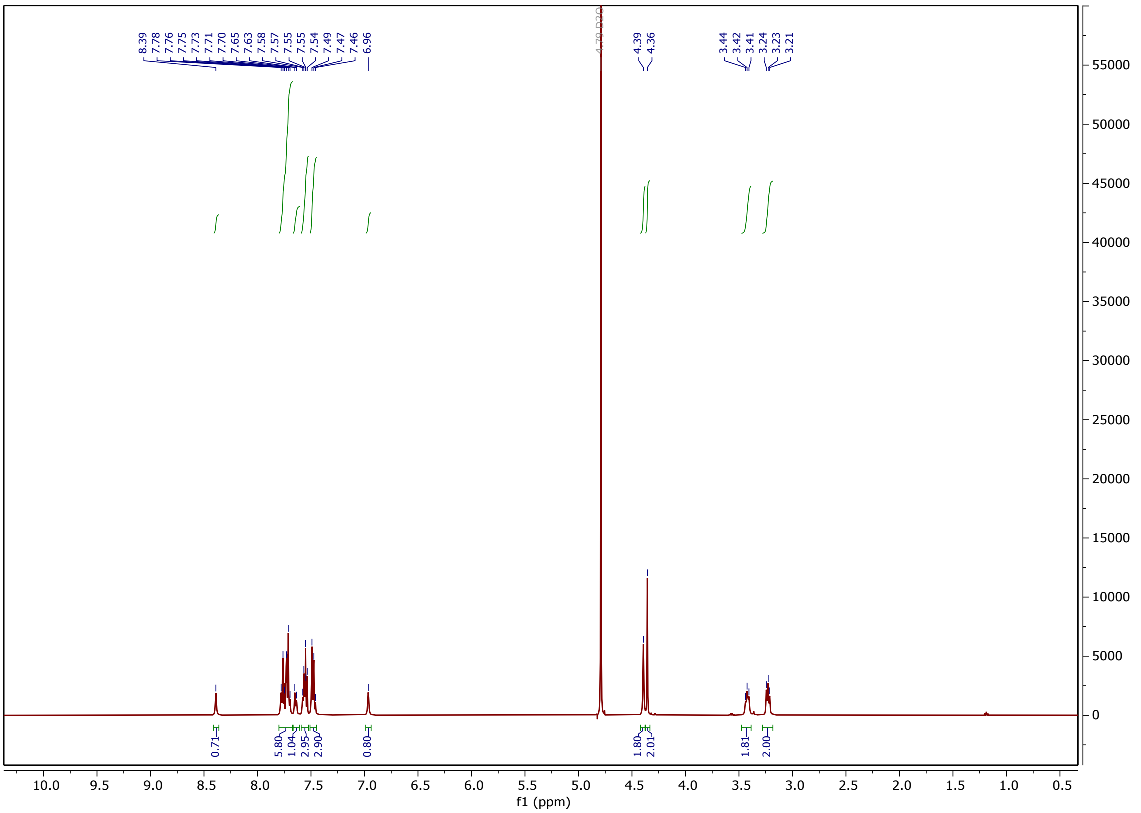

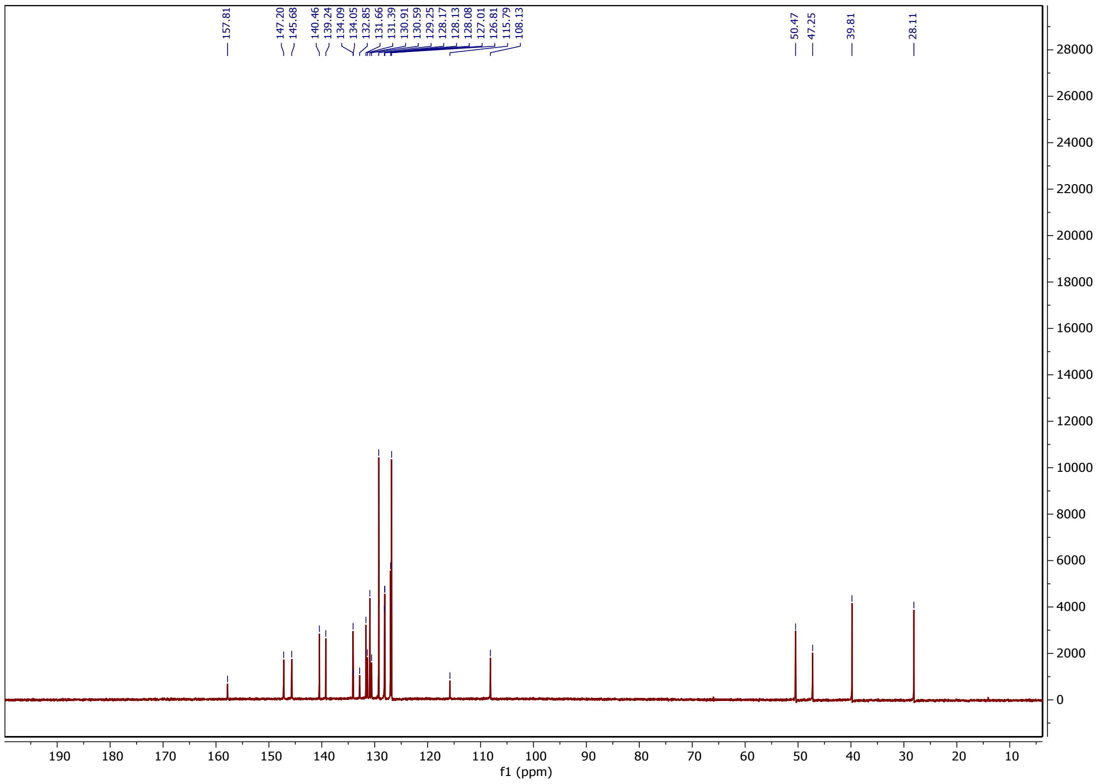

Compound 13

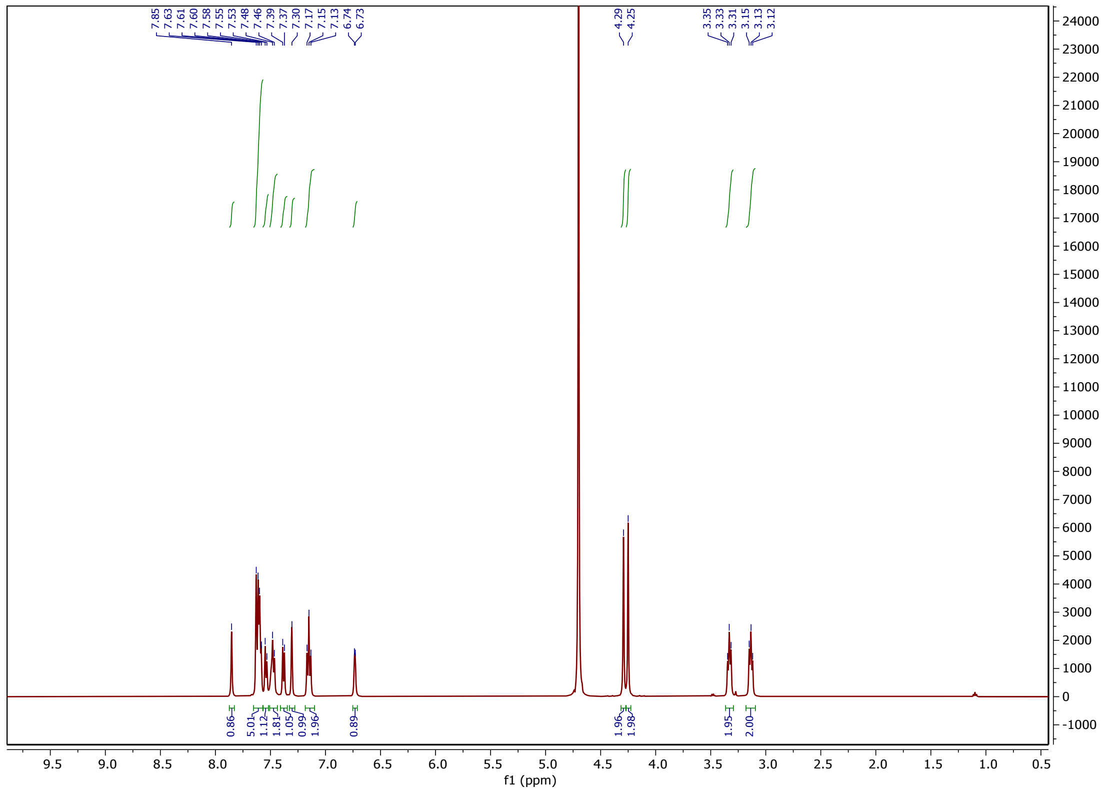

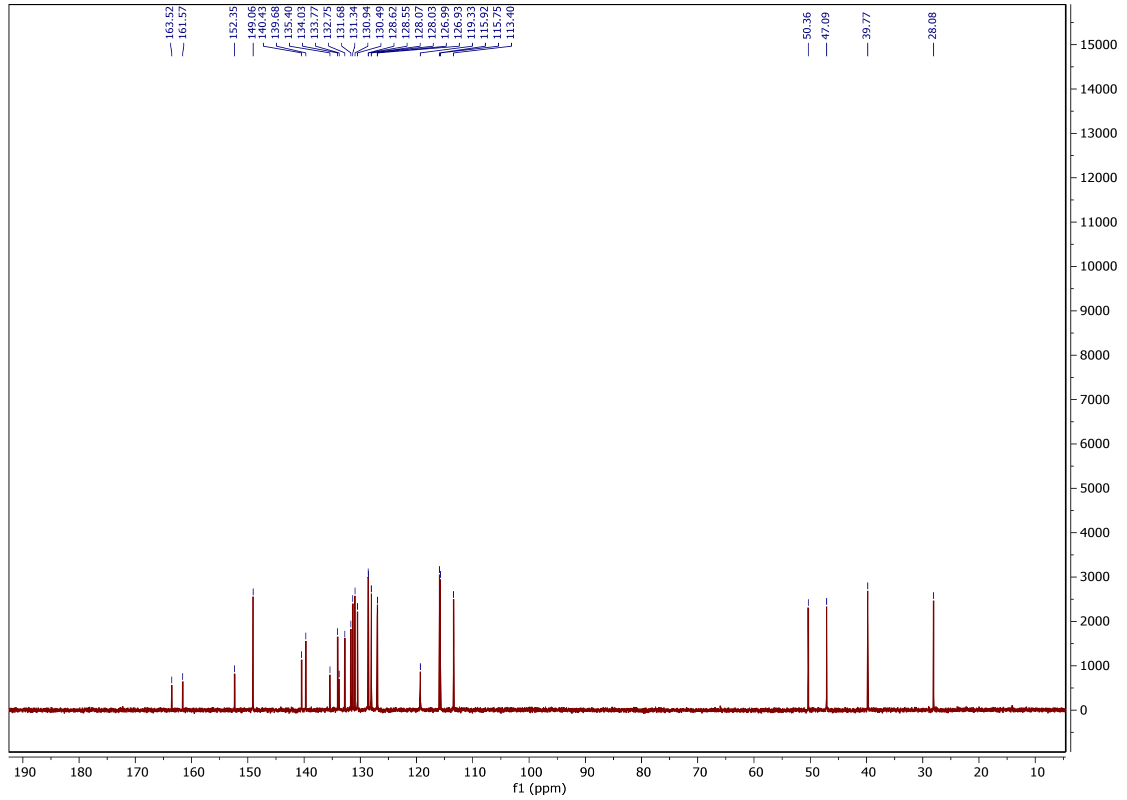

Compound 14

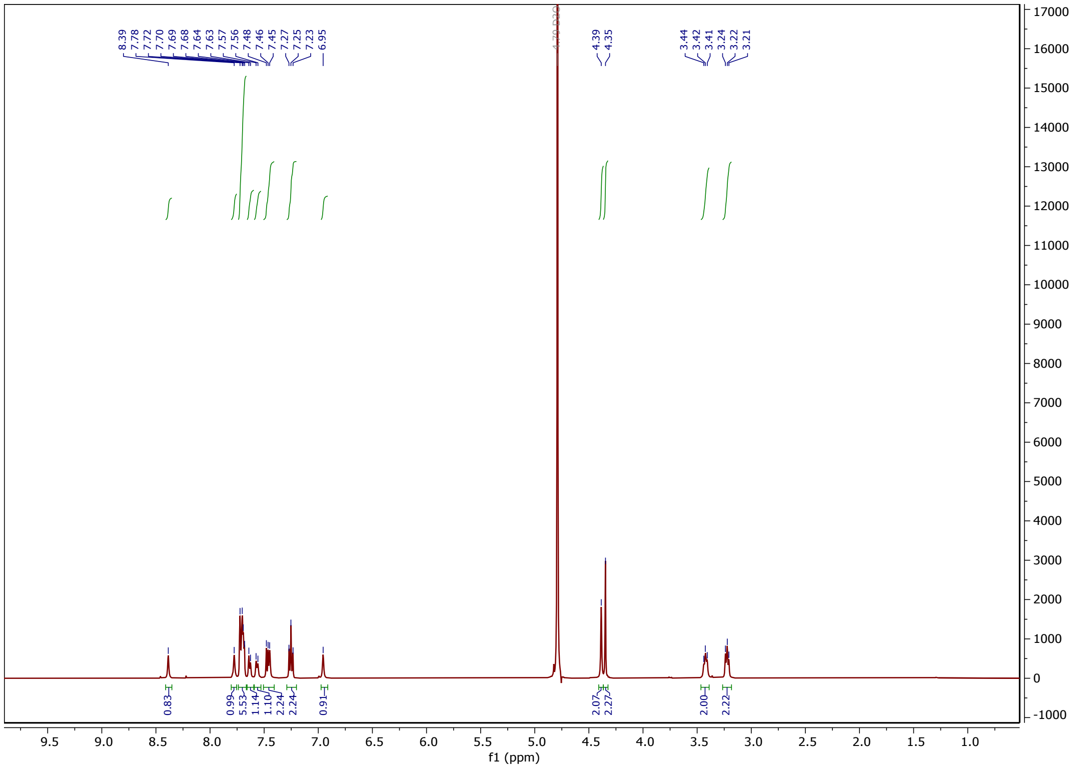
8.39
7.78
7.72
7.70
7.69
7.68
7.64
7.63
7.57
7.56
7.48
7.46
7.45
7.27
7.25
7.23
6.95
4.39
4.35
3.44
3.42
3.41
3.24
3.22
3.21
0.83
0.99
5.53
1.14
1.10
2.24
2.24
0.91
2.07
2.27
2.00
2.22
f1 (ppm)

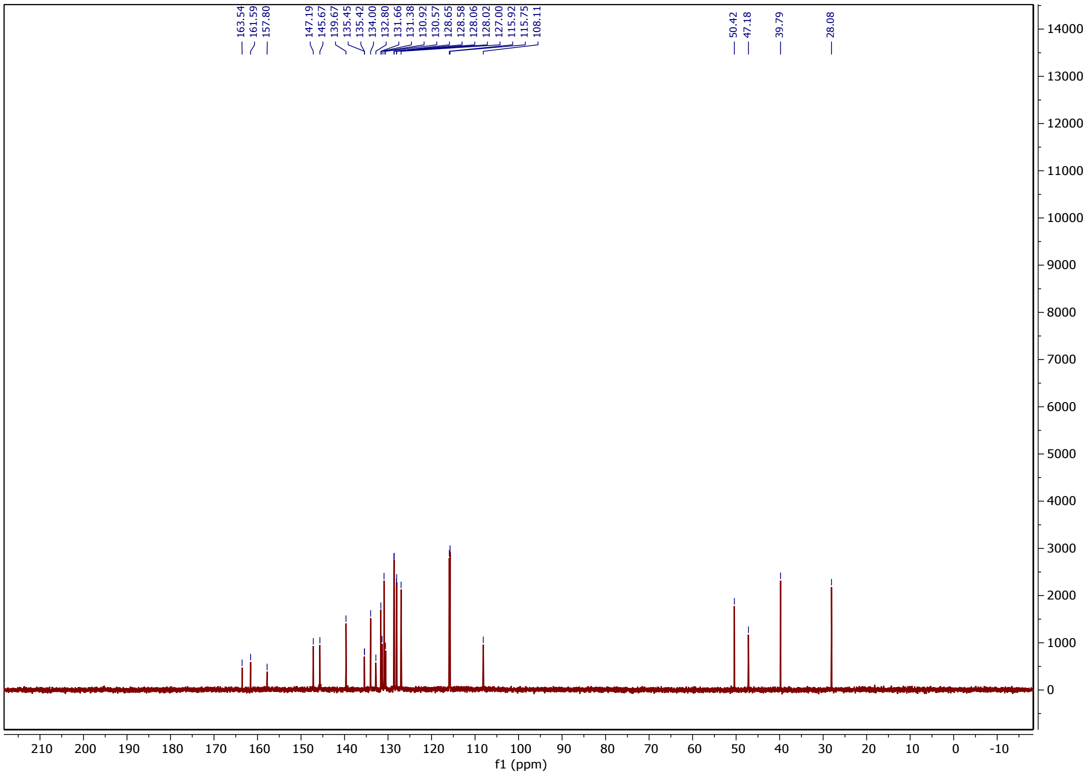
163.54
161.59
157.80
147.19
145.67
139.67
135.45
135.42
134.00
132.80
131.66
131.38
130.92
130.57
128.65
128.58
128.06
128.02
127.00
115.92
115.75
108.11
50.42
47.18
39.79
28.08
f1 (ppm)

Compound 15

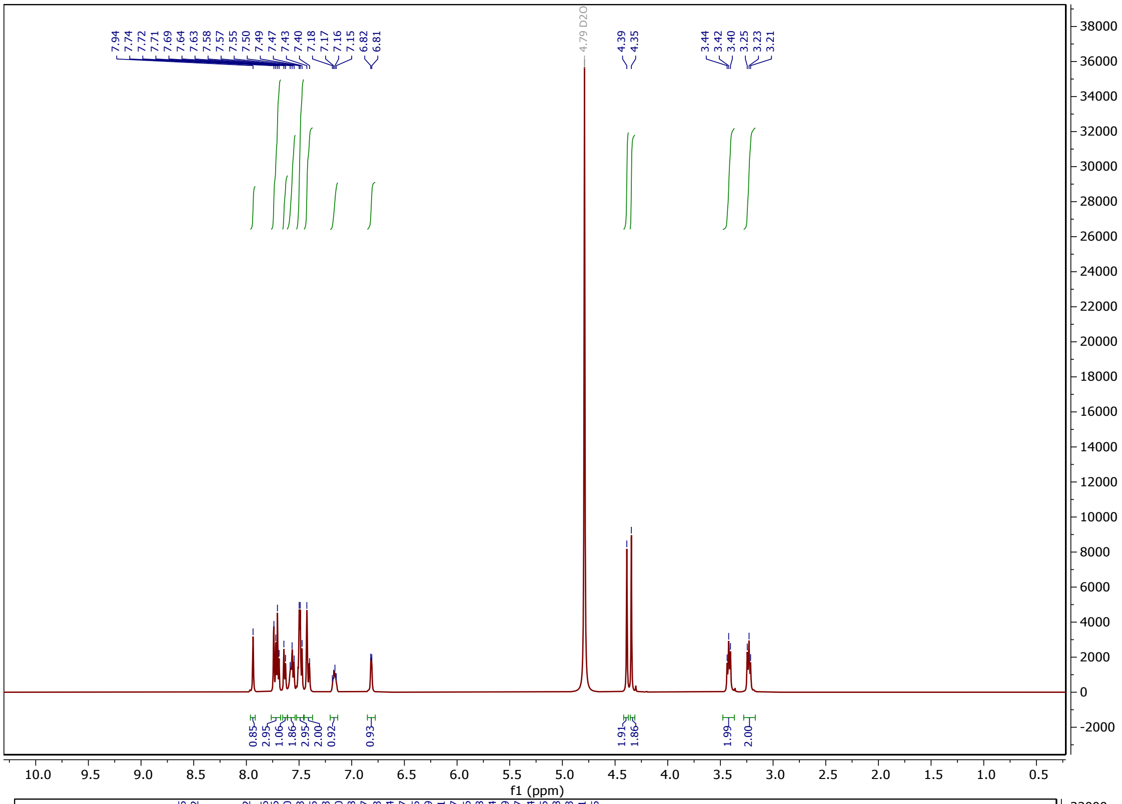
7.94 7.74 7.72 7.71 7.69 7.64 7.63 7.58 7.57 7.55 7.50 7.49 7.47 7.43 7.40 7.18 7.17 7.16 7.15 6.82 6.81
4.79 D2O
4.39 4.35
3.44 3.42 3.40 3.25 3.23 3.21
0.85 2.95 1.06 1.86 2.95 2.00 0.92 0.93 1.91 1.86 1.99 2.00
f1 (ppm)

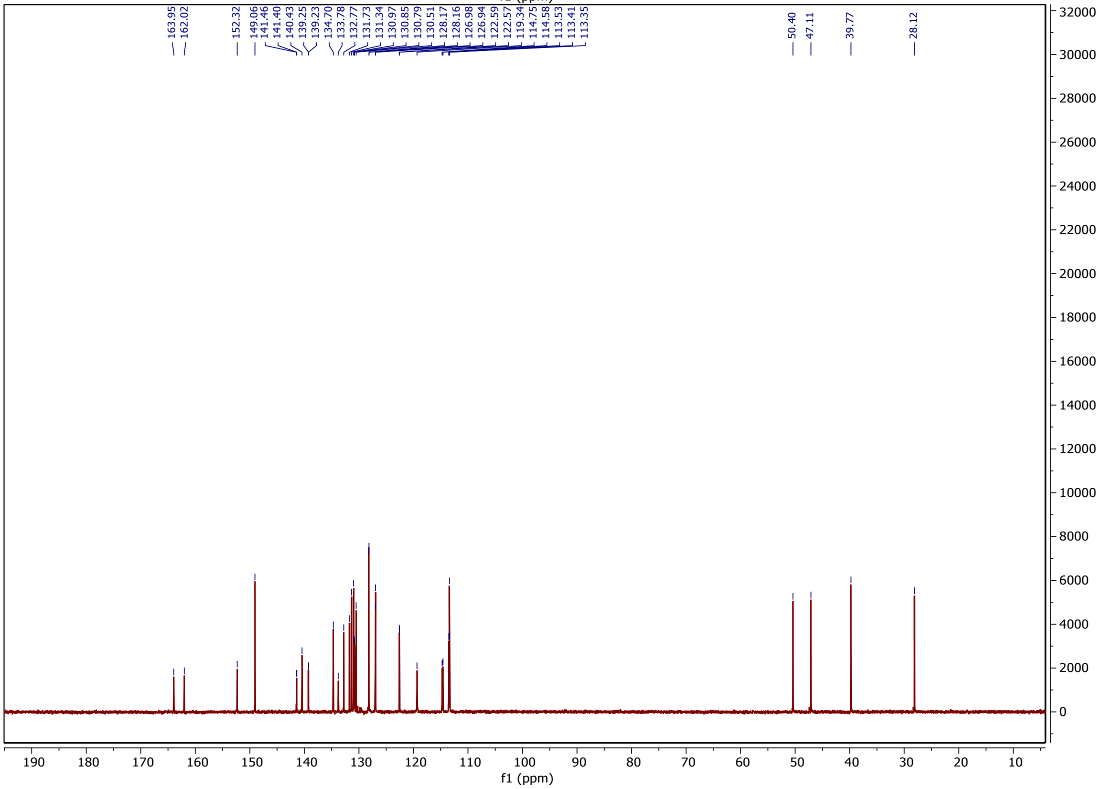
163.95 162.02 152.32 149.06 141.46 141.40 140.43 139.25 139.23 134.70 133.78 132.77 131.73 131.34 130.97 130.85 130.79 130.51 128.17 128.16 126.98 126.94 122.59 122.57 119.34 114.75 114.58 113.53 113.41 113.35
50.40 47.11 39.77 28.12
f1 (ppm)

Compound 16

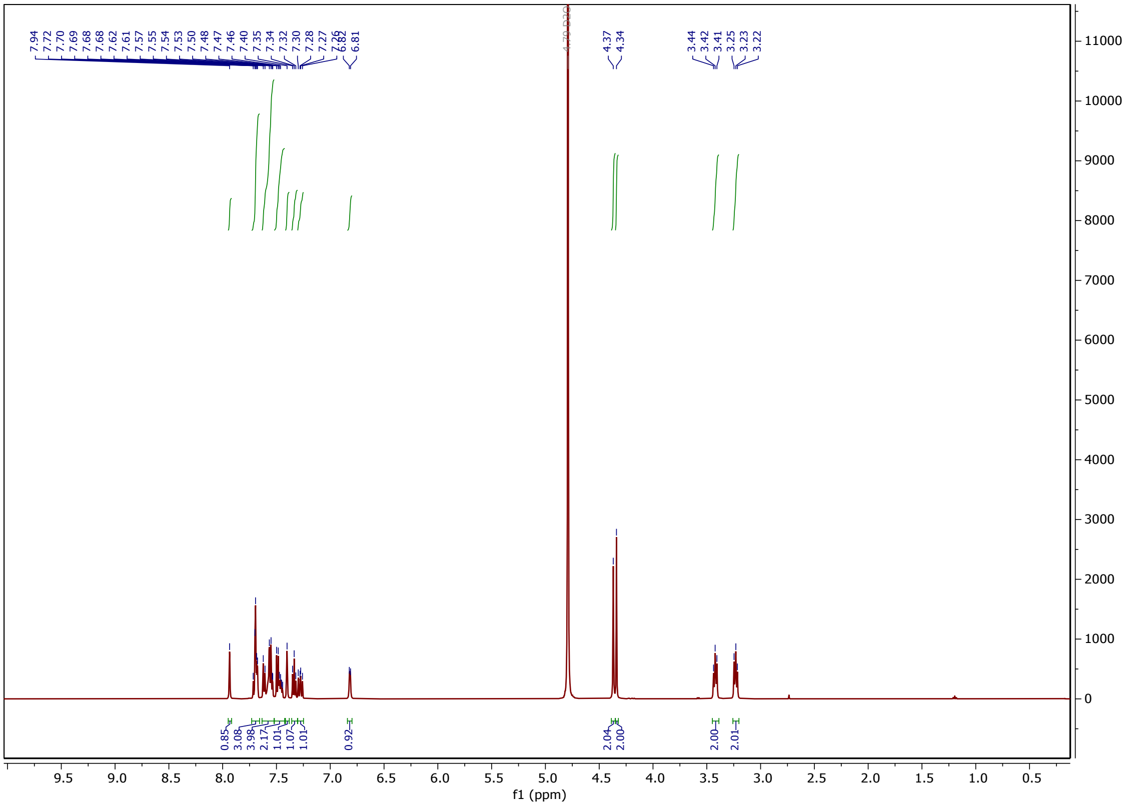

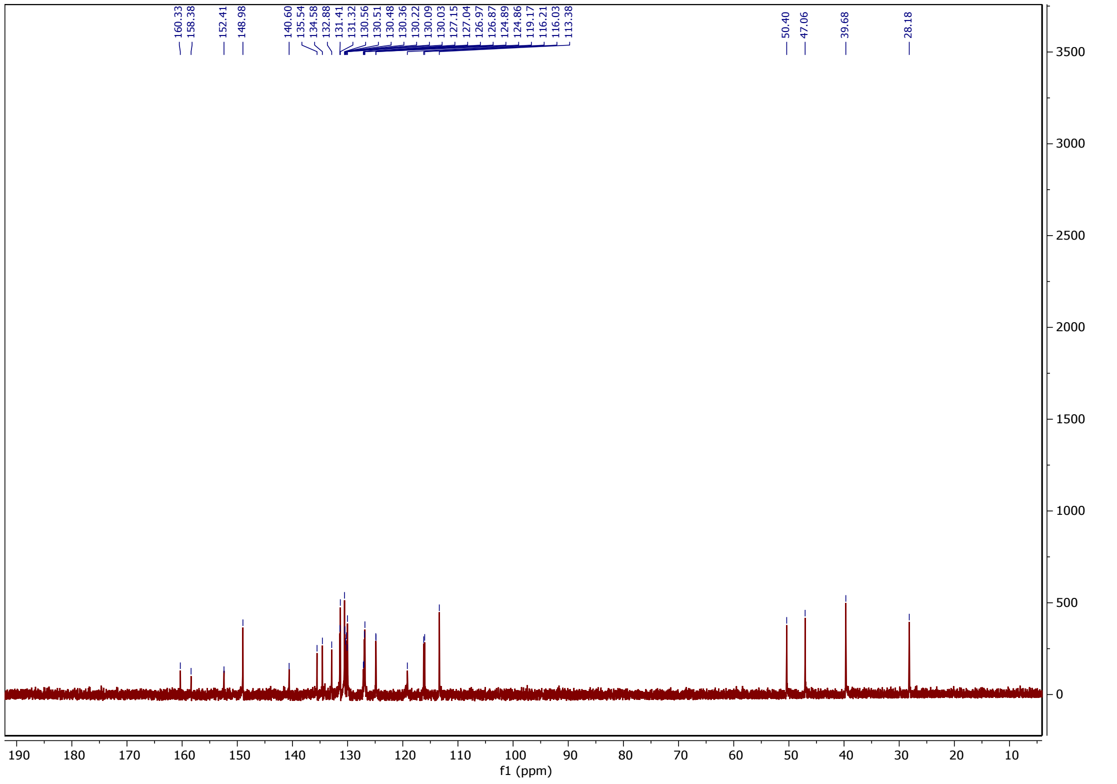

Compound 17

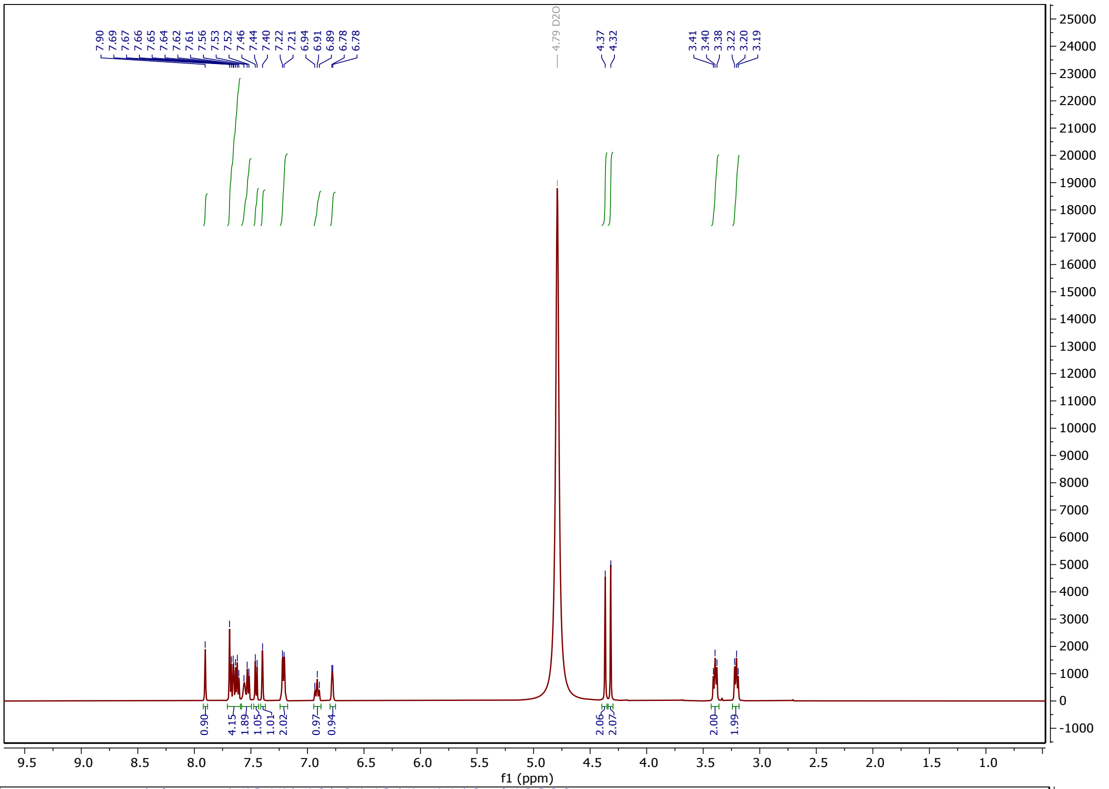

7.90
7.69
7.67
7.66
7.65
7.64
7.62
7.61
7.56
7.53
7.52
7.46
7.44
7.40
7.22
7.21
6.94
6.91
6.89
6.78
6.78
4.79 D2O
4.37
4.32
3.41
3.40
3.38
3.22
3.20
3.19
0.90
4.15
1.89
1.05
1.01
2.02
0.97
0.94
2.06
2.07
2.00
1.99
f1 (ppm)

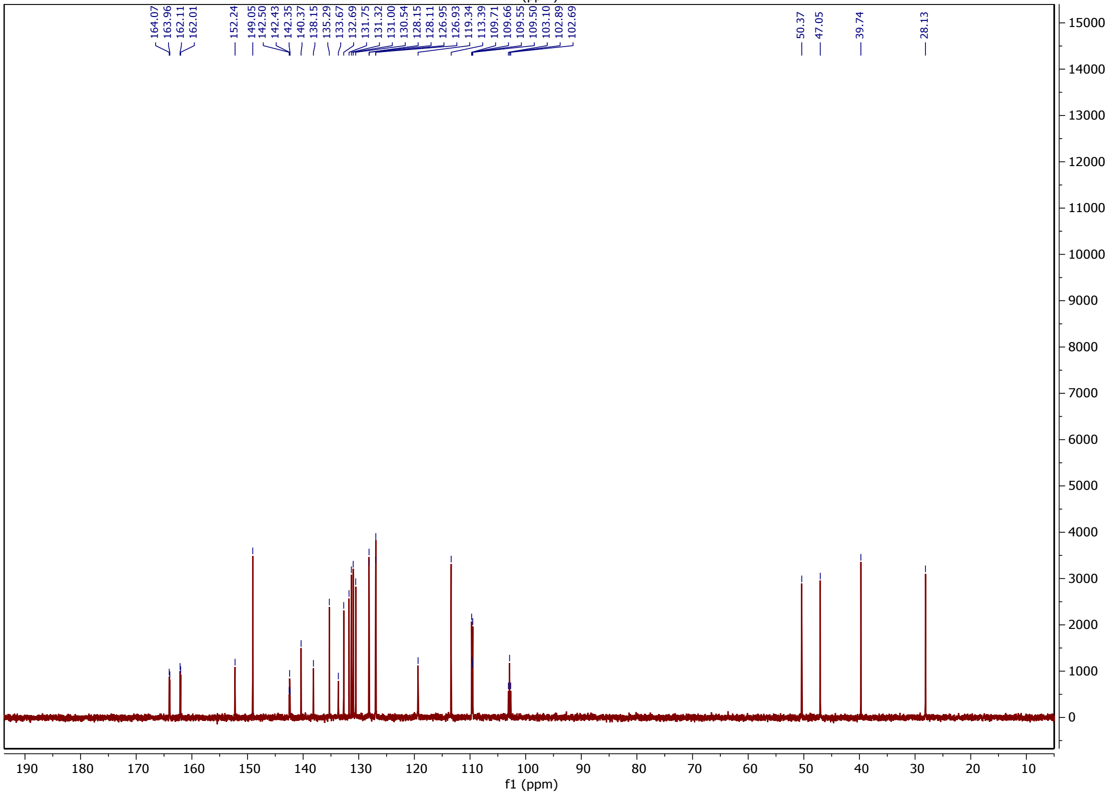

164.07
163.96
162.11
162.01
152.24
149.05
142.50
142.43
142.35
140.37
138.15
135.29
133.67
132.69
131.75
131.32
131.00
130.54
128.15
128.11
126.95
126.93
119.34
113.39
109.71
109.66
109.55
109.50
103.10
102.89
102.69
50.37
47.05
39.74
28.13
f1 (ppm)

Compound 18

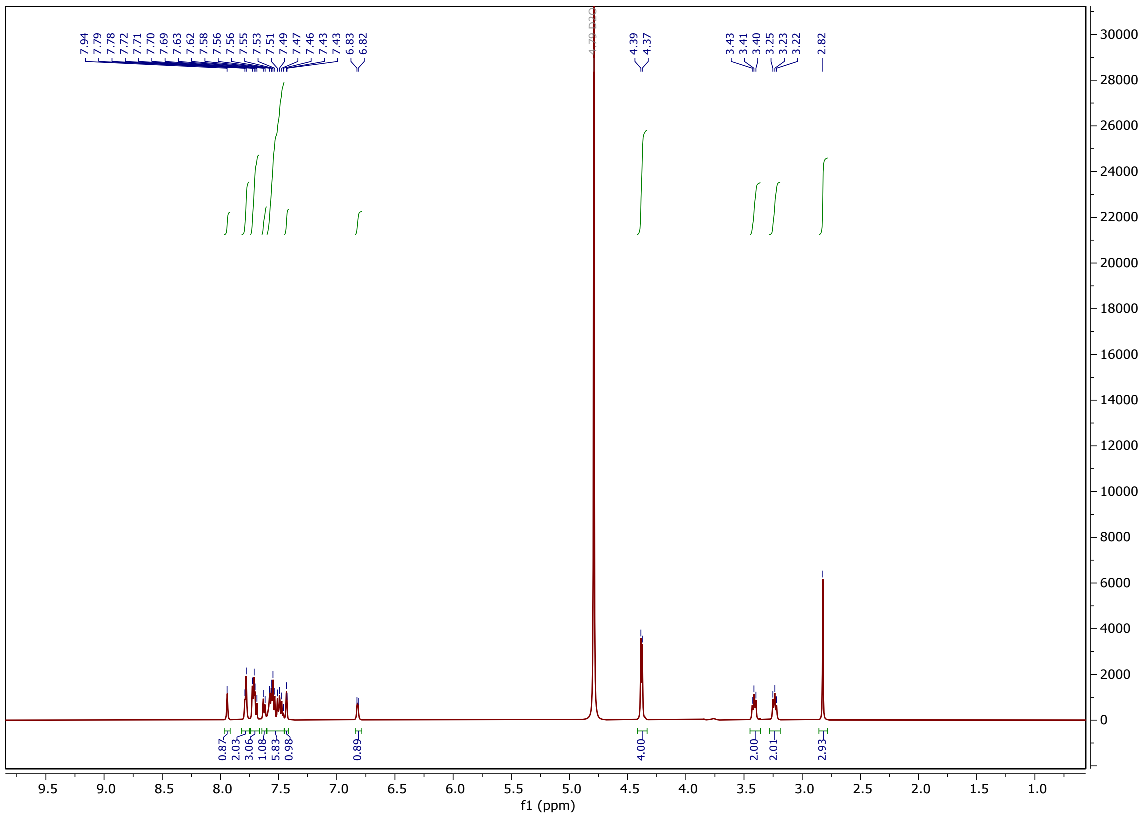

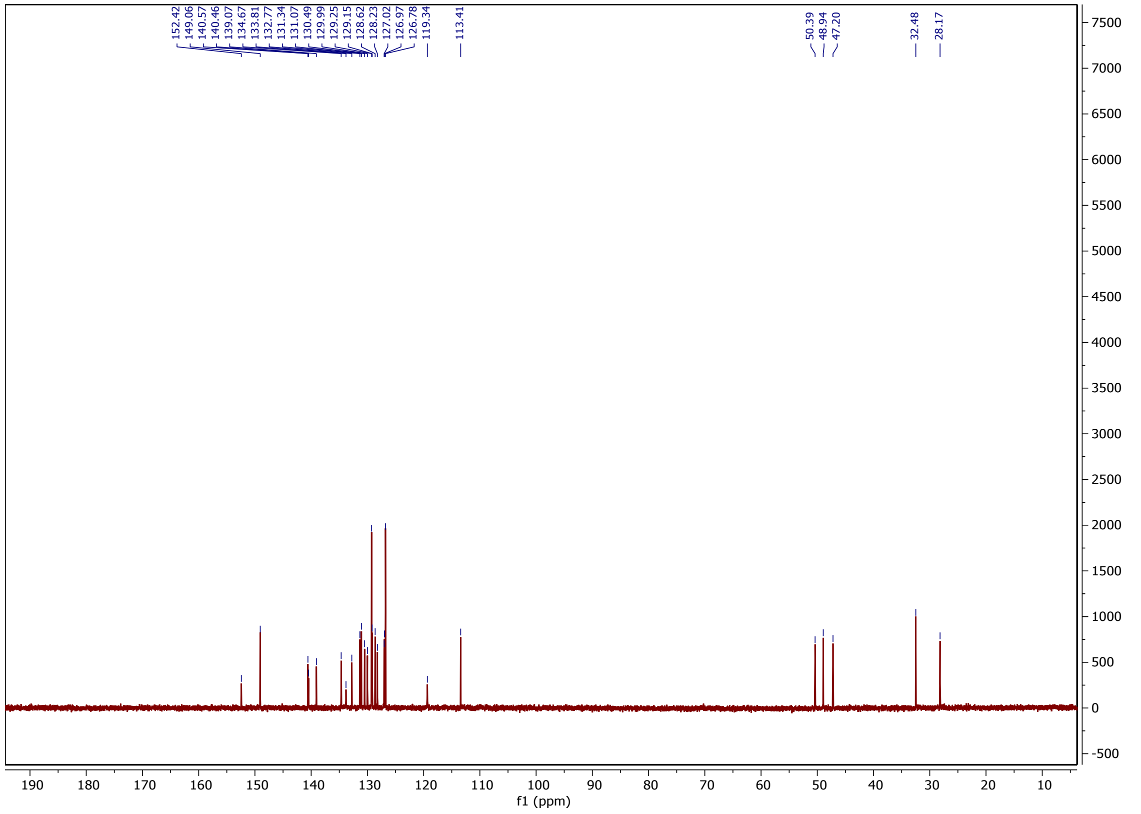

Compound 19

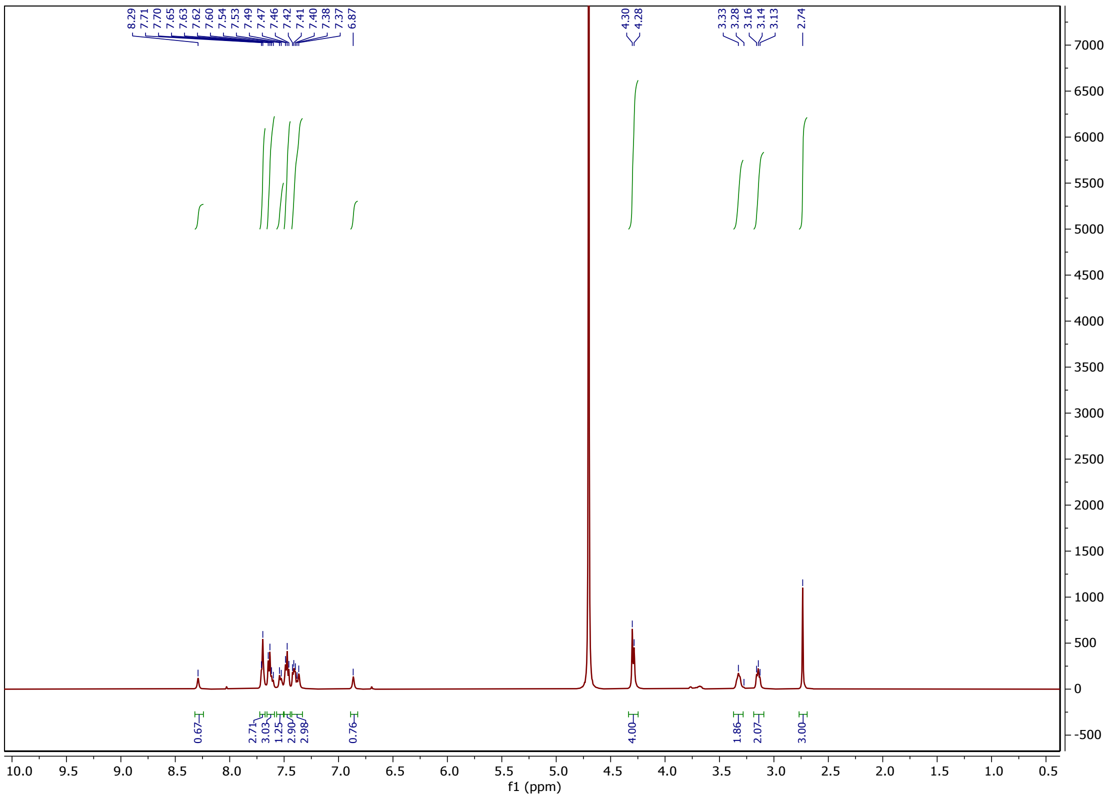

8.29 7.71 7.70 7.65 7.63 7.62 7.60 7.54 7.53 7.49 7.47 7.46 7.42 7.41 7.40 7.38 7.37 6.87
4.30 4.28
3.33 3.28 3.16 3.14 3.13
2.74
0.67 2.71 3.03 1.25 2.90 2.98 0.76 4.00 1.86 2.07 3.00
f1 (ppm)

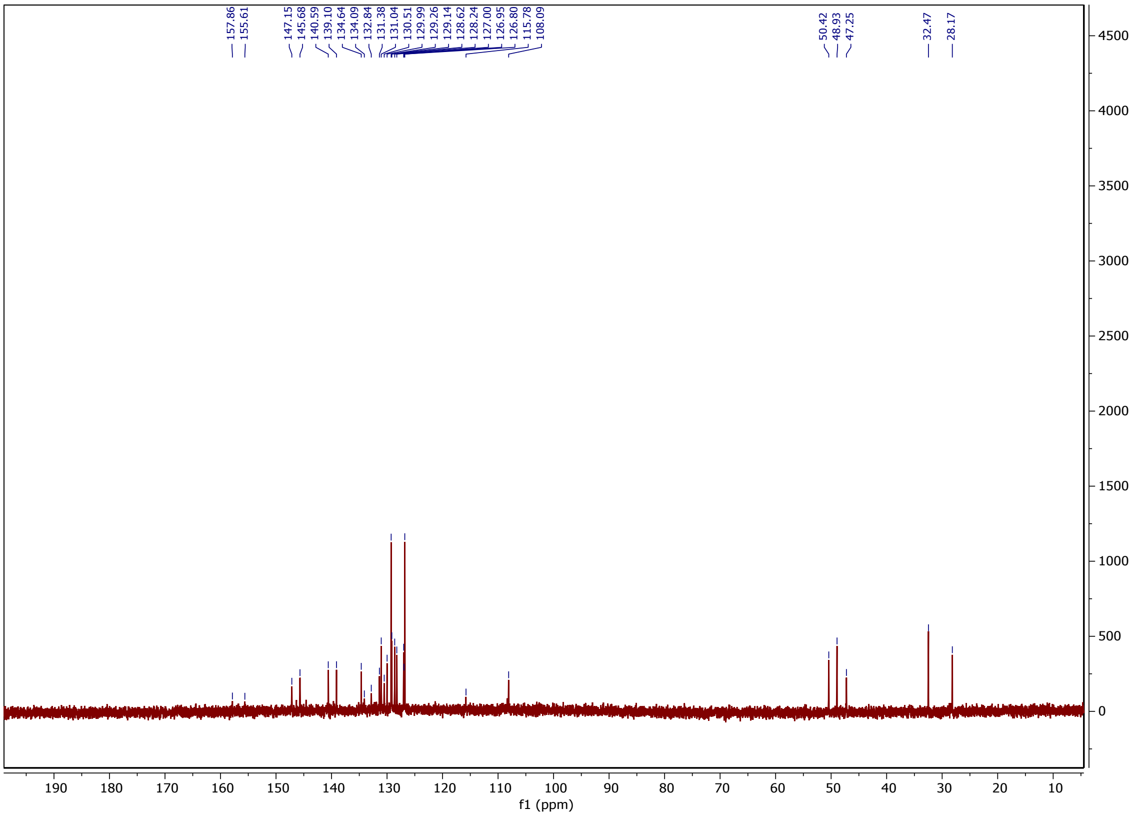

157.86 155.61 147.15 145.68 140.59 139.10 134.64 134.09 132.84 131.38 131.04 130.51 129.99 129.26 129.14 128.62 128.24 127.00 126.95 126.80 115.78 108.09
50.42 48.93 47.25
32.47 28.17
f1 (ppm)

Compound 20

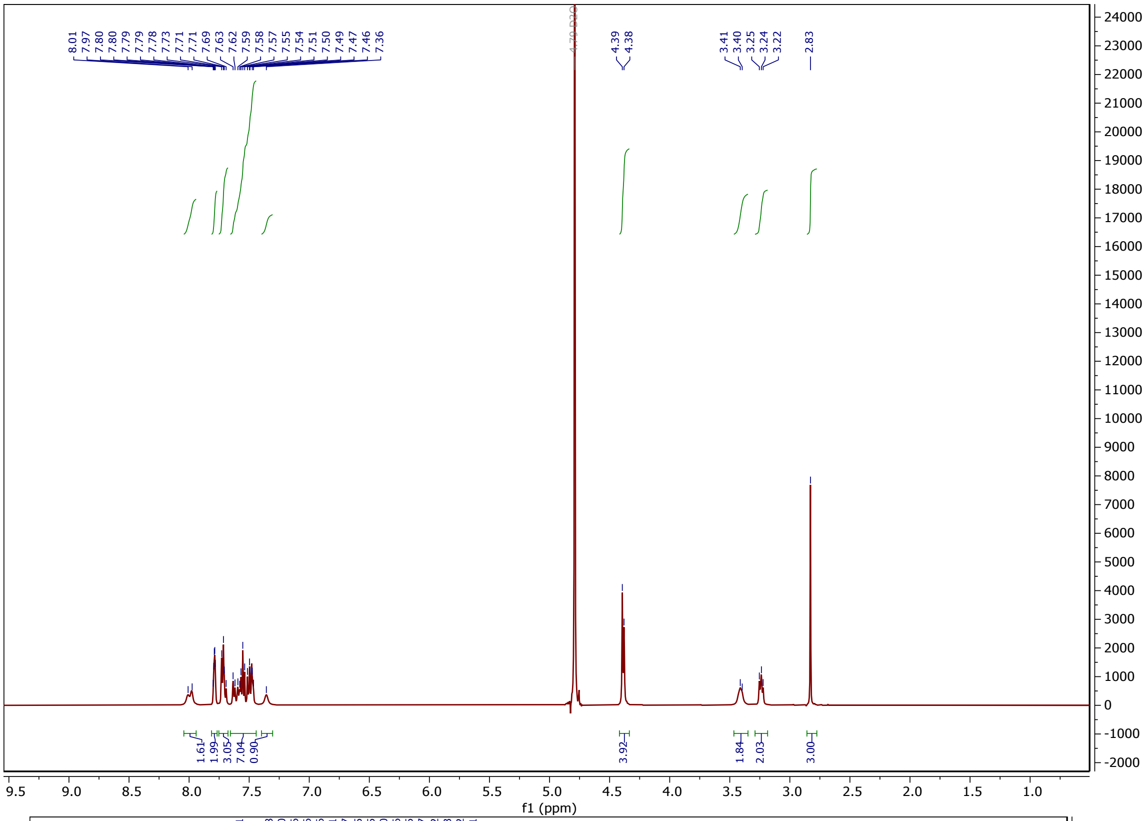
8.01
7.97
7.80
7.80
7.79
7.79
7.78
7.73
7.71
7.71
7.69
7.63
7.62
7.59
7.58
7.57
7.55
7.54
7.51
7.50
7.49
7.47
7.46
7.36
4.39
4.38
3.41
3.40
3.25
3.24
3.22
2.83
1.61
1.99
3.05
7.04
0.90
3.92
1.84
2.03
3.00
f1 (ppm)

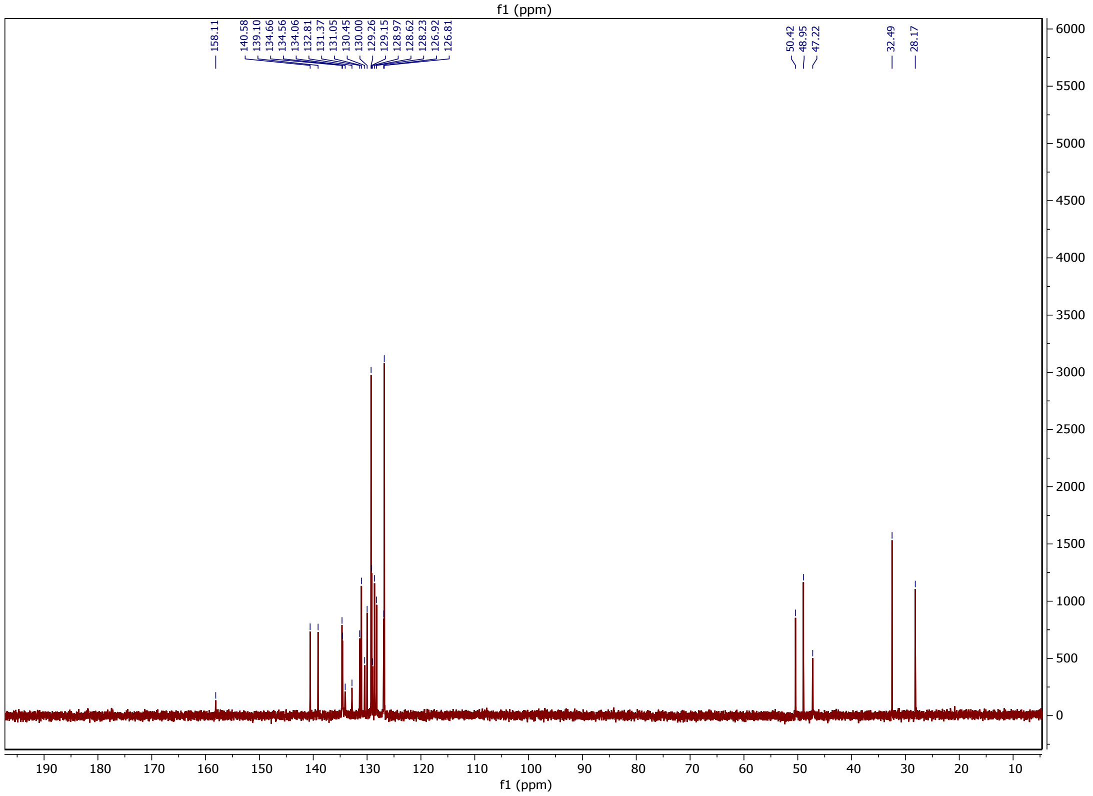
158.11
140.58
139.10
134.66
134.56
134.06
132.81
131.37
131.05
130.45
130.00
129.26
129.15
128.97
128.62
128.23
126.92
126.81
50.42
48.95
47.22
32.49
28.17
f1 (ppm)

Compound 21

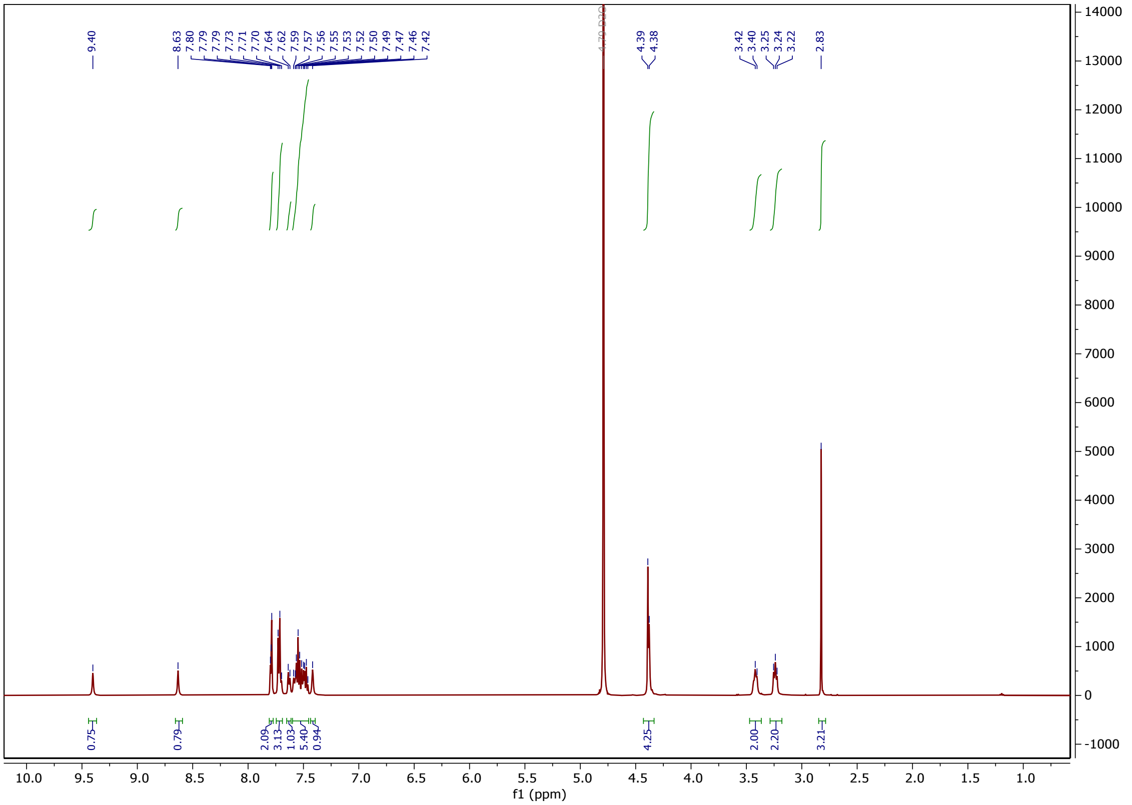

9.40
8.63 7.80 7.79 7.79 7.73 7.71 7.70 7.64 7.62 7.59 7.57 7.56 7.55 7.53 7.52 7.50 7.49 7.47 7.46 7.42
4.39 4.38
3.42 3.40 3.25 3.24 3.22
2.83
0.75 0.79 2.09 3.13 1.03 5.40 0.94 4.25 2.00 2.20 3.21
f1 (ppm)

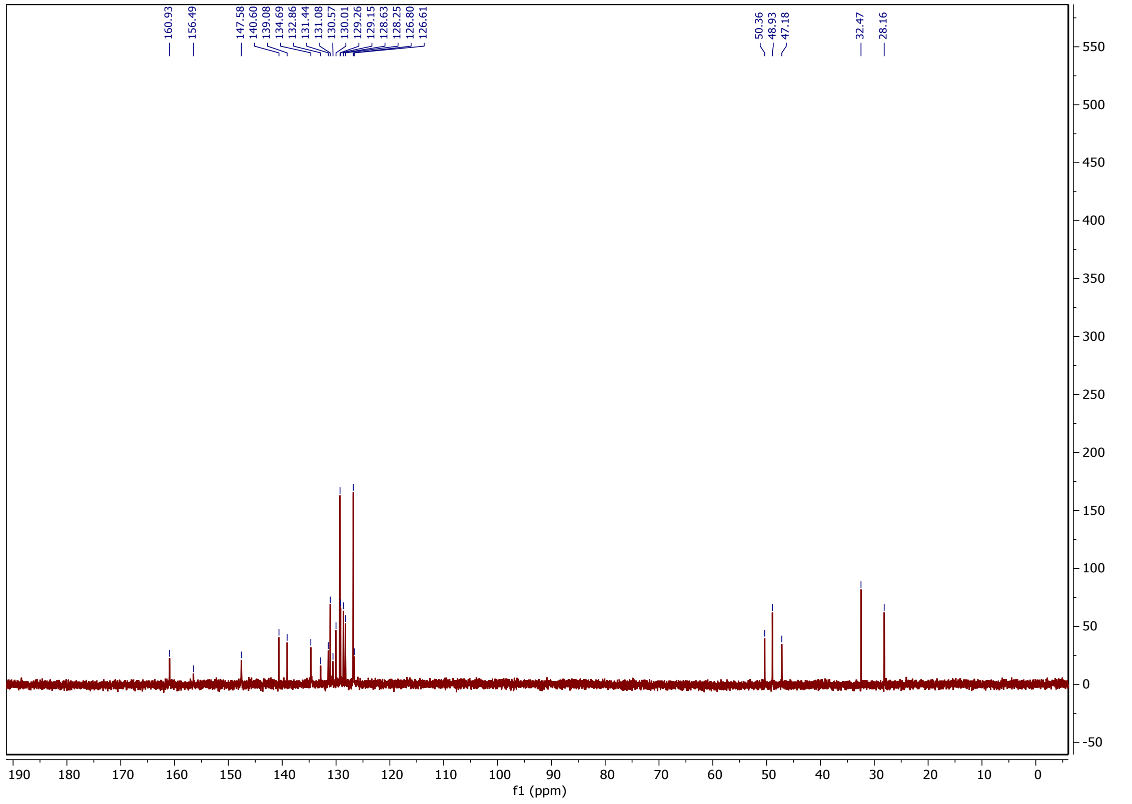

160.93 156.49 147.58 140.60 139.08 134.69 132.86 131.44 131.08 130.57 130.01 129.26 129.15 128.63 128.25 126.80 126.61
50.36 48.93 47.18 32.47 28.16
f1 (ppm)

13. HPLC traces of target compounds 1-21

Compound 1

Sample Information

Acquired by : System Administrator
Date Acquired : 6/17/2025 5:09:57 PM
Sample Name : AA-01-63 salt1
Tray# : 1
Vial# : 1
Data File : 20250616_Shutdown_LC-MS-ELSD_AA-01-63 salt1_004.lcd
Method File : LC-MS-ELSD.lcm
Report Format File : DEFAULT.lsr
Processed by : System Administrator
Date Processed : 7/2/2025 2:50:45 PM

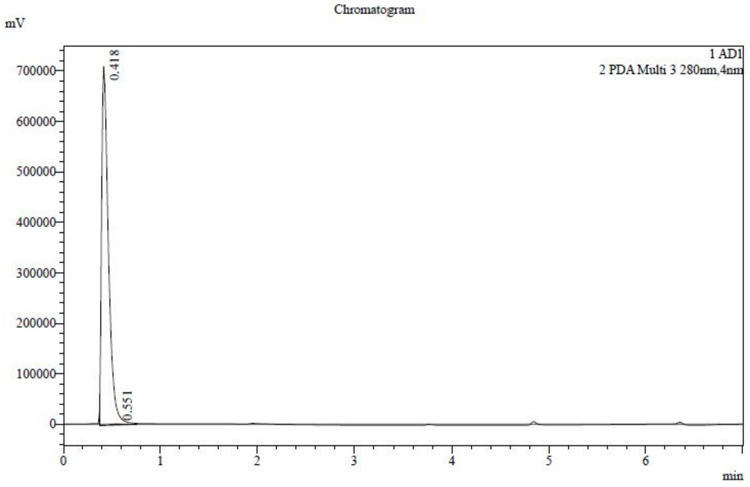

Peak Table

PDA Ch3 280nm

Peak#	Ret. Time	Area	Height	Area%
1	0.418	3487668	711948	100.000
Total		3487668	711948	100.000

Compound 2

Sample Information

Acquired by : System Administrator
Date Acquired : 6/17/2025 5:40:31 PM
Sample Name : AA-01-133 salt1
Tray# : 1
Vial# : 3
Data File : 20250616_Shutdown_LC-MS-ELSD_AA-01-133 salt1_001.lcd
Method File : LC-MS-ELSD.lcm
Report Format File : DEFAULT.lsr
Processed by : System Administrator
Date Processed : 7/2/2025 3:10:14 PM

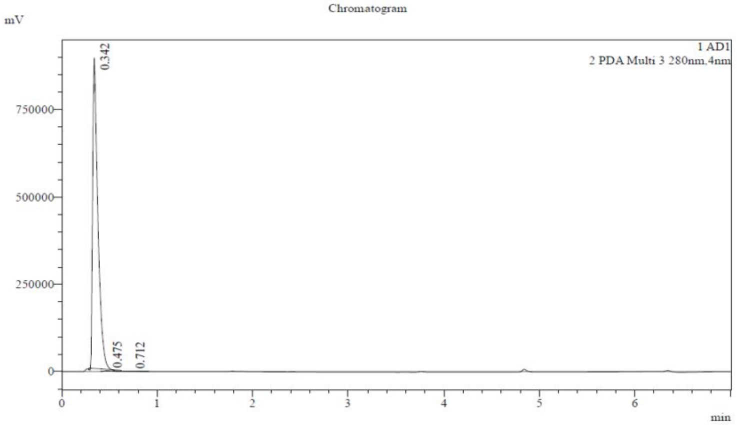

Peak Table

PDA Ch3 280nm

Peak#	Ret. Time	Area	Height	Area%
1	0.342	3377662	890791	100.000
Total		3377662	890791	100.000

Compound 3

Sample Information

Acquired by	: System Administrator
Date Acquired	: 6/17/2025 7:02:09 PM
Sample Name	: AA-01-155 SALT 3
Tray#	: 1
Vial#	: 6
Data File	: 20250616_Shutdown_LC-MS-ELSD_AA-01-155 SALT 3_001.lcd
Method File	: LC-MS-ELSD.lcm
Report Format File	: DEFAULT.lsr
Processed by	: System Administrator
Date Processed	: 7/2/2025 3:54:01 PM

Chromatogram

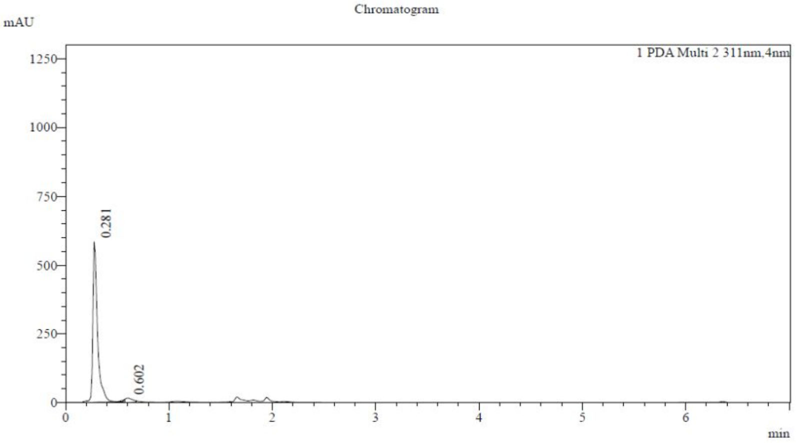

Peak Table

PDA Ch2 311nm

Peak#	Ret. Time	Area	Height	Area%
1	0.281	1860659	586179	96.146
2	0.602	74589	16097	3.854
Total		1935248	602276	100.000

Compound 4

Sample Information

Acquired by	: System Administrator
Date Acquired	: 6/17/2025 6:32:31 PM
Sample Name	: AA-02-59 salt 2
Tray#	: 1
Vial#	: 7
Data File	: 20250616_Shutdown_LC-MS-ELSD_AA-02-59 salt 2_001.lcd
Method File	: LC-MS-ELSD.lcm
Report Format File	: DEFAULT.lsr
Processed by	: System Administrator
Date Processed	: 7/2/2025 3:28:33 PM

Chromatogram

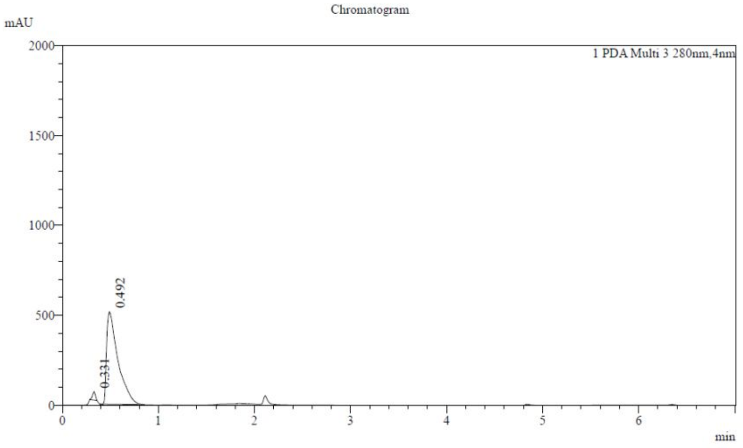

Peak Table

PDA Ch3 280nm

Peak#	Ret. Time	Area	Height	Area%
1	0.331	94104	47191	2.174
2	0.492	4233774	514858	97.826
Total		4327879	562049	100.000

Compound 5

Sample Information

Acquired by	: System Administrator
Date Acquired	: 6/17/2025 5:55:19 PM
Sample Name	: AA-01-115 salt1
Tray#	: 1
Vial#	: 5
Data File	: 20250616_Shutdown_LC-MS-ELSD_AA-01-115 salt1_003.lcd
Method File	: LC-MS-ELSD.lcm
Report Format File	: DEFAULT.lsr
Processed by	: System Administrator
Date Processed	: 7/2/2025 3:30:02 PM

Chromatogram

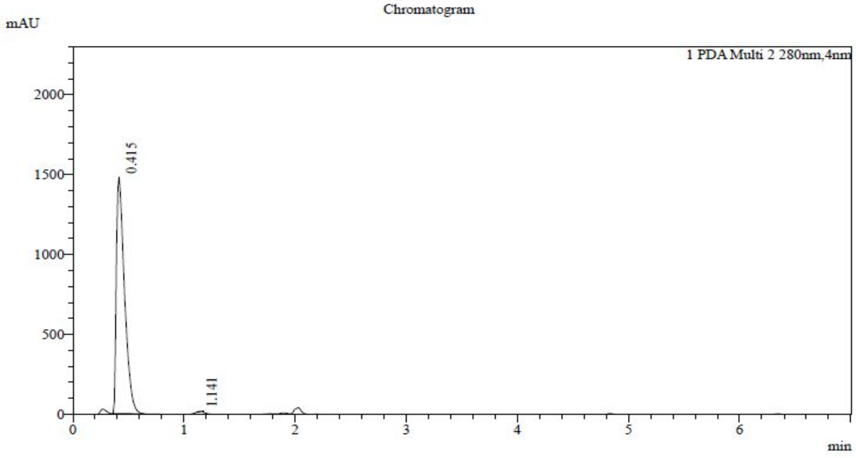

Peak Table

PDA Ch2 280nm

Peak#	Ret. Time	Area	Height	Area%
1	0.415	7506309	1480584	99.995
2	1.141	386	3109	0.005
Total		7506695	1483693	100.000

Compound 6

Sample Information

Acquired by	: System Administrator
Date Acquired	: 6/17/2025 6:17:28 PM
Sample Name	: AA-01-73 salt1
Tray#	: 1
Vial#	: 8
Data File	: 20250616_Shutdown_LC-MS-ELSD_AA-01-73 salt1_006.lcd
Method File	: LC-MS-ELSD.lcm
Report Format File	: DEFAULT.lsr
Processed by	: System Administrator
Date Processed	: 7/2/2025 4:11:00 PM

Chromatogram

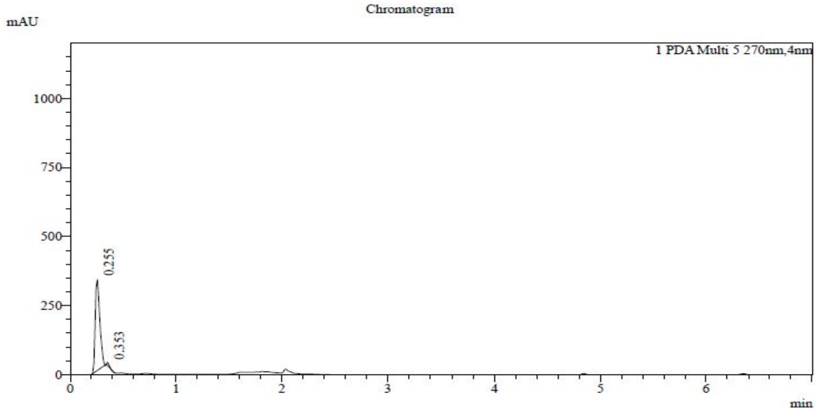

Peak Table

PDA Ch5 270nm

Peak#	Ret. Time	Area	Height	Area%
1	0.255	1009849	330315	99.416
2	0.353	5930	11802	0.584
Total		1015779	342118	100.000

Compound 7

Sample Information

Acquired by	: System Administrator
Date Acquired	: 6/17/2025 5:47:56 PM
Sample Name	: AA-01-135 salt2
Tray#	: 1
Vial#	: 4
Data File	: 20250616_Shutdown_LC-MS-ELSD_AA-01-135 salt2_002.lcd
Method File	: LC-MS-ELSD.lcm
Report Format File	: DEFAULT.lsr
Processed by	: System Administrator
Date Processed	: 7/2/2025 3:34:47 PM

Chromatogram

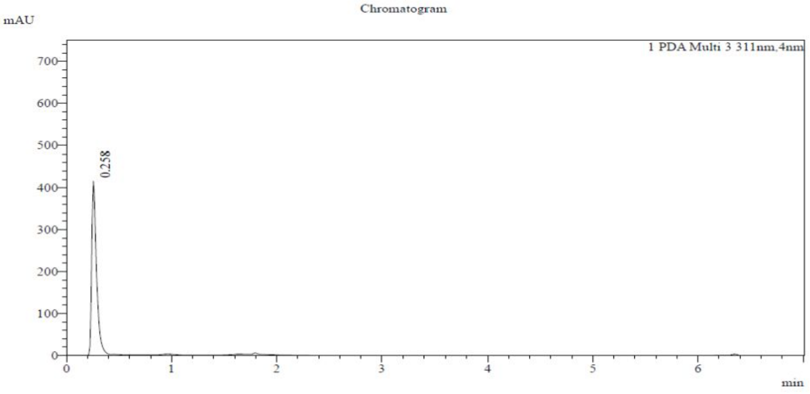

Peak Table

PDA Ch3 311nm

Peak#	Ret. Time	Area	Height	Area%
1	0.258	1357300	416287	100.000
Total		1357300	416287	100.000

Compound 8

Sample Information

Acquired by	: System Administrator
Date Acquired	: 6/17/2025 5:24:45 PM
Sample Name	: AA-01-65 salt1
Tray#	: 1
Vial#	: 2
Data File	: 20250616_Shutdown_LC-MS-ELSD_AA-01-65 salt1_002.lcd
Method File	: LC-MS-ELSD.lcm
Report Format File	: DEFAULT.lsr
Processed by	: System Administrator
Date Processed	: 7/2/2025 3:00:08 PM

Chromatogram

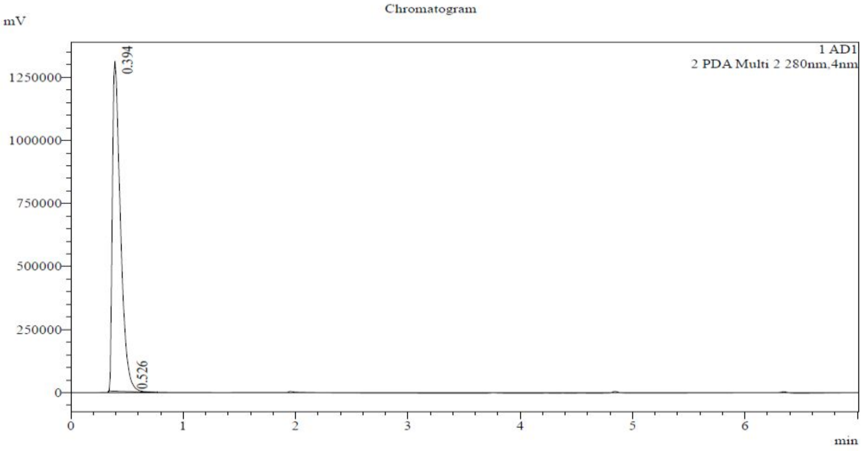

Peak Table

PDA Ch2 280nm

Peak#	Ret. Time	Area	Height	Area%
1	0.394	6522551	1310064	100.000
Total		6522551	1310064	100.000

Compound 9

Sample Information

Acquired by : System Administrator
Date Acquired : 6/30/2025 5:13:43 PM
Sample Name : AA-02-16 -9
Tray# : 1
Vial# : 9
Data File : 20250617_Shutdown_LC-MS-ELSD_AA-02-16 -9_001.lcd
Method File : LC-MS-ELSD.lcm
Report Format File : DEFAULT.lsr
Processed by : System Administrator
Date Processed : 7/2/2025 3:38:18 PM

Chromatogram

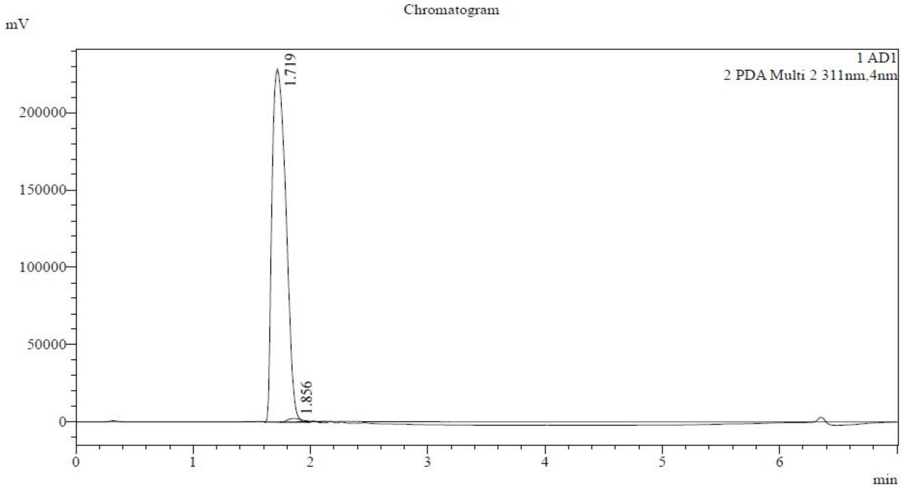

Peak Table

PDA Ch2 311nm

Peak#	Ret. Time	Area	Height	Area%
1	1.719	1885092	228131	100.000
Total		1885092	228131	100.000

Compound 10

Sample Information

Acquired by : System Administrator
Date Acquired : 6/30/2025 5:21:06 PM
Sample Name : AA-02-17 -10
Tray# : 1
Vial# : 10
Data File : 20250617_Shutdown_LC-MS-ELSD_AA-02-17 -10_001.lcd
Method File : LC-MS-ELSD.lcm
Report Format File : DEFAULT.lsr
Processed by : System Administrator
Date Processed : 7/2/2025 3:39:13 PM

Chromatogram

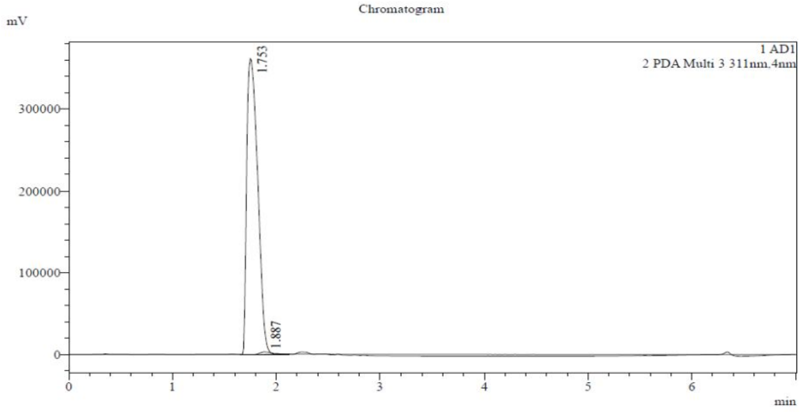

Peak Table

PDA Ch3 311nm

Peak#	Ret. Time	Area	Height	Area%
1	1.753	2684349	361474	100.000
Total		2684349	361474	100.000

Compound 11

Sample Information

Acquired by	: System Administrator
Date Acquired	: 6/30/2025 5:28:30 PM
Sample Name	: AA-02-18 -11
Tray#	: 1
Vial#	: 11
Data File	: 20250617_Shutdown_LC-MS-ELSD_AA-02-18 -11_002.lcd
Method File	: LC-MS-ELSD.lcm
Report Format File	: DEFAULT.lsr
Processed by	: System Administrator
Date Processed	: 7/2/2025 3:41:17 PM

Chromatogram

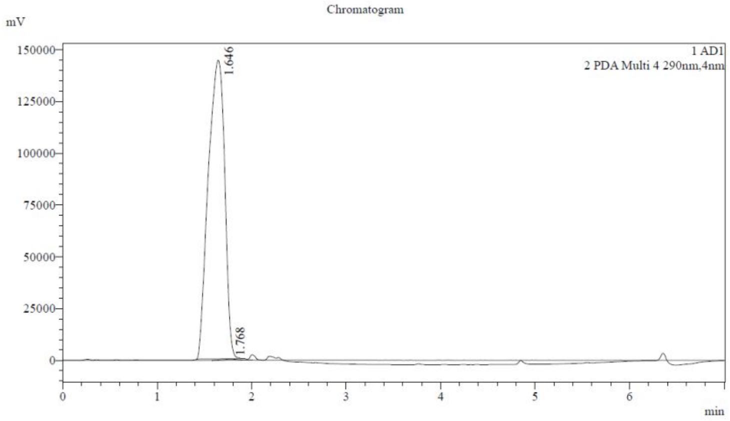

Peak Table

PDA Ch4 290nm

Peak#	Ret. Time	Area	Height	Area%
1	1.646	1741071	144166	100.000
Total		1741071	144166	100.000

Compound 12

Sample Information

Acquired by	: System Administrator
Date Acquired	: 6/30/2025 6:57:14 PM
Sample Name	: AA-02-77 12B
Tray#	: 1
Vial#	: 22
Data File	: 20250617_Shutdown_LC-MS-ELSD_AA-02-77 12B_001.lcd
Method File	: LC-MS-ELSD.lcm
Report Format File	: DEFAULT.lsr
Processed by	: System Administrator
Date Processed	: 7/2/2025 3:42:37 PM

Chromatogram

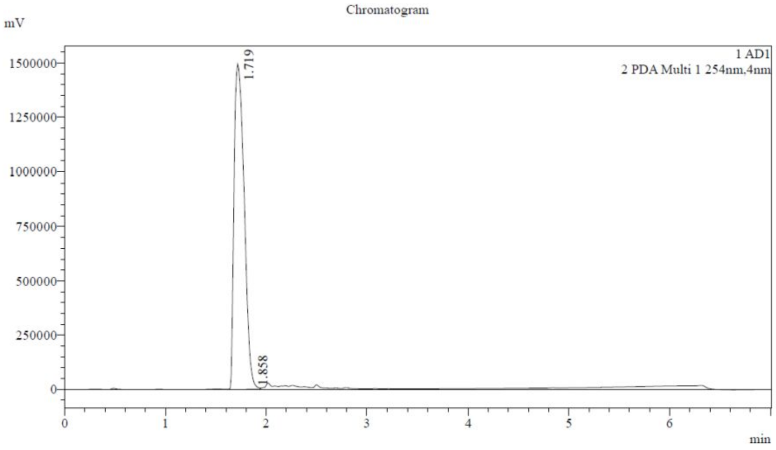

Peak Table

PDA Ch1 254nm

Peak#	Ret. Time	Area	Height	Area%
1	1.719	10472800	1494795	100.000
Total		10472800	1494795	100.000

Compound 13

Sample Information

Acquired by	: System Administrator
Date Acquired	: 6/30/2025 5:43:18 PM
Sample Name	: AA-02-81 -13
Tray#	: 1
Vial#	: 13
Data File	: 20250617_Shutdown_LC-MS-ELSD_AA-02-81 -13_004.lcd
Method File	: LC-MS-ELSD.lcm
Report Format File	: DEFAULT.lsr
Processed by	: System Administrator
Date Processed	: 7/2/2025 3:43:27 PM

Chromatogram

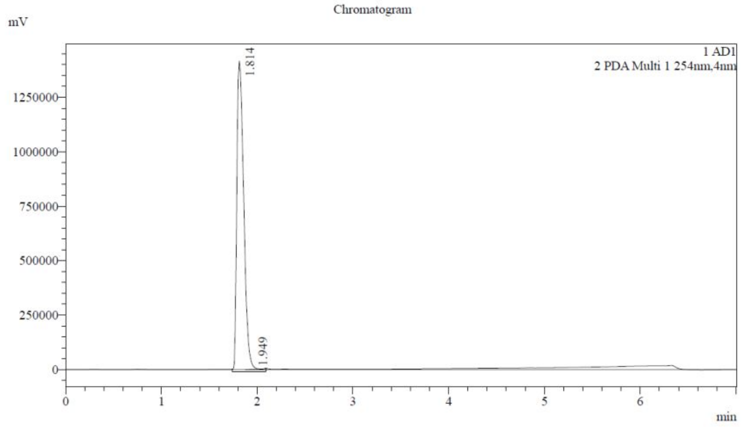

Peak Table

PDA Ch1 254nm

Peak#	Ret. Time	Area	Height	Area%
1	1.814	7276410	1425361	100.000
Total		7276410	1425361	100.000

Compound 14

Sample Information

Acquired by	: System Administrator
Date Acquired	: 6/30/2025 5:50:41 PM
Sample Name	: AA-02-82-14
Tray#	: 1
Vial#	: 14
Data File	: 20250617_Shutdown_LC-MS-ELSD_AA-02-82-14_005.lcd
Method File	: LC-MS-ELSD.lcm
Report Format File	: DEFAULT.lsr
Processed by	: System Administrator
Date Processed	: 7/2/2025 3:43:58 PM

Chromatogram

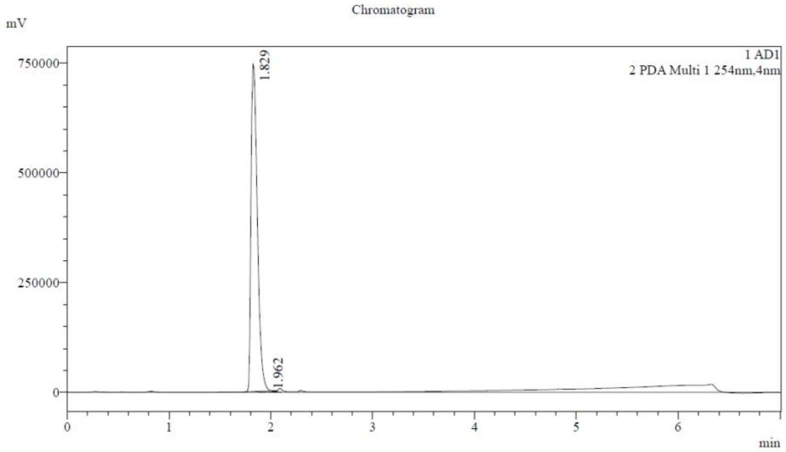

Peak Table

PDA Ch1 254nm

Peak#	Ret. Time	Area	Height	Area%
1	1.829	3207629	744567	100.000
Total		3207629	744567	100.000

Compound 15

Sample Information

Acquired by	: System Administrator
Date Acquired	: 6/30/2025 5:58:06 PM
Sample Name	: AA-02-111 -15
Tray#	: 1
Vial#	: 15
Data File	: 20250617_Shutdown_LC-MS-ELSD_AA-02-111 -15_006.lcd
Method File	: LC-MS-ELSD.lcm
Report Format File	: DEFAULT.lsr
Processed by	: System Administrator
Date Processed	: 7/2/2025 3:44:43 PM

Chromatogram

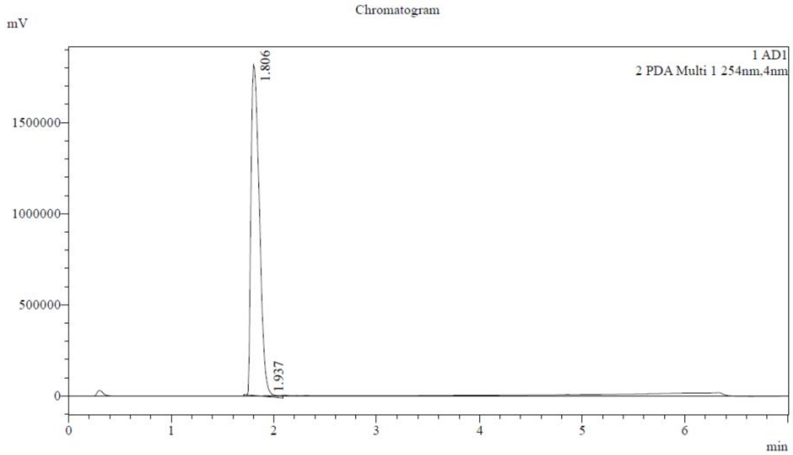

Peak Table

PDA Ch1 254nm

Peak#	Ret. Time	Area	Height	Area%
1	1.806	10410126	1812472	100.000
Total		10410126	1812472	100.000

Compound 16

Sample Information

Acquired by	: System Administrator
Date Acquired	: 6/30/2025 6:05:29 PM
Sample Name	: AA-02-112 -16
Tray#	: 1
Vial#	: 16
Data File	: 20250617_Shutdown_LC-MS-ELSD_AA-02-112 -16_007.lcd
Method File	: LC-MS-ELSD.lcm
Report Format File	: DEFAULT.lsr
Processed by	: System Administrator
Date Processed	: 7/2/2025 3:45:06 PM

Chromatogram

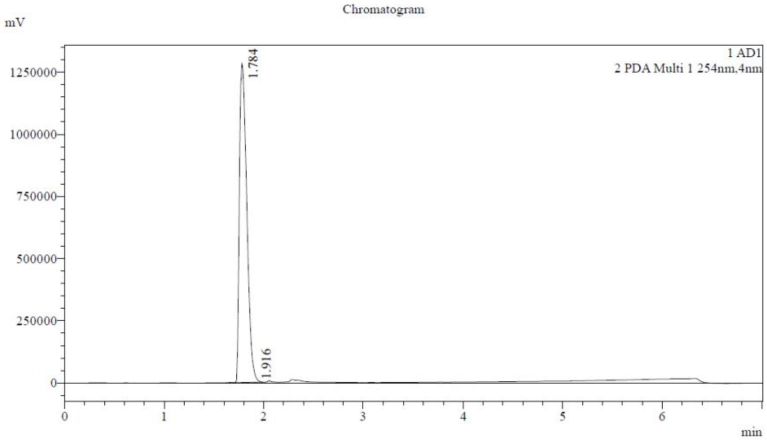

Peak Table

PDA Ch1 254nm

Peak#	Ret. Time	Area	Height	Area%
1	1.784	6659112	1285564	100.000
Total		6659112	1285564	100.000

Compound 17

Sample Information

Acquired by	: System Administrator
Date Acquired	: 6/30/2025 6:12:52 PM
Sample Name	: AA-02-181 -17
Tray#	: 1
Vial#	: 17
Data File	: 20250617_Shutdown_LC-MS-ELSD_AA-02-181 -17_008.lcd
Method File	: LC-MS-ELSD.lcm
Report Format File	: DEFAULT.lsr
Processed by	: System Administrator
Date Processed	: 7/2/2025 3:45:37 PM

Chromatogram

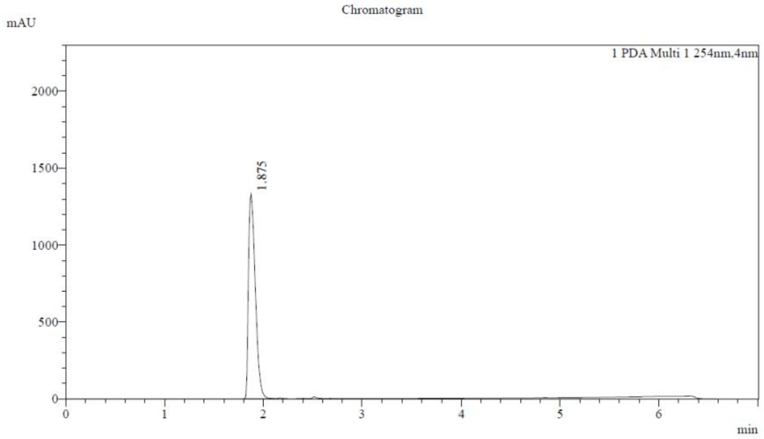

Peak Table

PDA Ch1 254nm

Peak#	Ret. Time	Area	Height	Area%
1	1.875	6423439	1331232	100.000
Total		6423439	1331232	100.000

Compound 18

Sample Information

Acquired by	: System Administrator
Date Acquired	: 6/30/2025 6:20:15 PM
Sample Name	: AA-02-48-18
Tray#	: 1
Vial#	: 18
Data File	: 20250617_Shutdown_LC-MS-ELSD_AA-02-48-18_009.lcd
Method File	: LC-MS-ELSD.lcm
Report Format File	: DEFAULT.lsr
Processed by	: System Administrator
Date Processed	: 7/2/2025 3:45:55 PM

Chromatogram

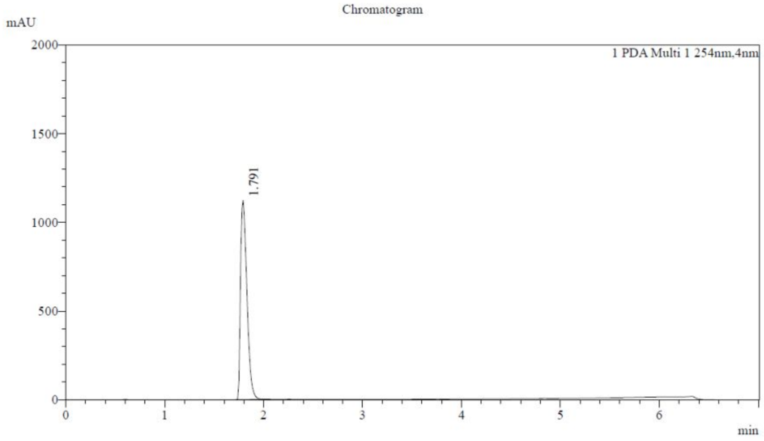

Peak Table

PDA Ch1 254nm

Peak#	Ret. Time	Area	Height	Area%
1	1.791	5069106	1124100	100.000
Total		5069106	1124100	100.000

Compound 19

Sample Information

Acquired by : System Administrator
Date Acquired : 6/30/2025 6:49:50 PM
Sample Name : AA-02-49 -19
Tray# : 1
Vial# : 19
Data File : 20250617_Shutdown_LC-MS-ELSD_AA-02-49 -19_001.lcd
Method File : LC-MS-ELSD.lcm
Report Format File : DEFAULT.lsr
Processed by : System Administrator
Date Processed : 7/2/2025 3:46:23 PM

Chromatogram

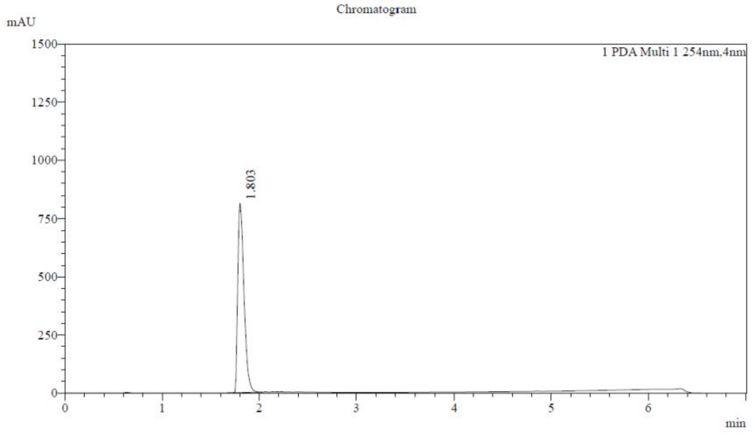

Peak Table

PDA Ch1 254nm

Peak#	Ret. Time	Area	Height	Area%
1	1.803	3649274	816573	100.000
Total		3649274	816573	100.000

Compound 20

Sample Information

Acquired by : System Administrator
Date Acquired : 6/30/2025 6:35:02 PM
Sample Name : AA-02-50 -20
Tray# : 1
Vial# : 20
Data File : 20250617_Shutdown_LC-MS-ELSD_AA-02-50 -20_011.lcd
Method File : LC-MS-ELSD.lcm
Report Format File : DEFAULT.lsr
Processed by : System Administrator
Date Processed : 7/2/2025 3:46:42 PM

Chromatogram

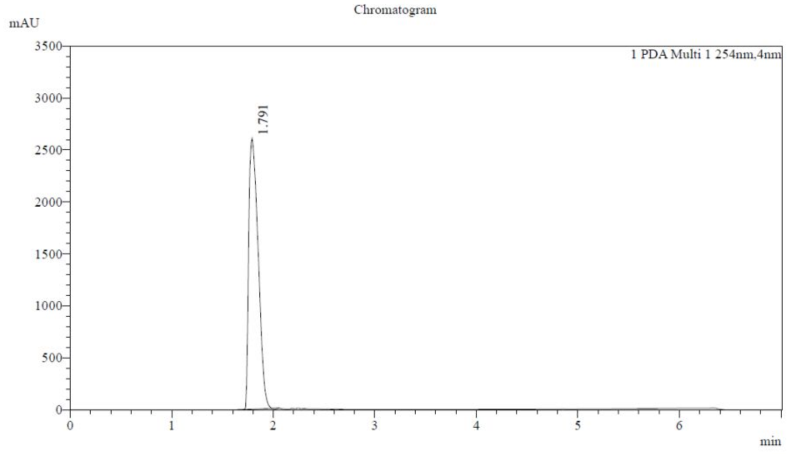

Peak Table

PDA Ch1 254nm

Peak#	Ret. Time	Area	Height	Area%
1	1.791	16837005	2606721	100.000
Total		16837005	2606721	100.000

Compound 21

Sample Information

Acquired by	: System Administrator
Date Acquired	: 6/30/2025 6:42:26 PM
Sample Name	: AA-02-51 -21
Tray#	: 1
Vial#	: 21
Data File	: 20250617_Shutdown_LC-MS-ELSD_AA-02-51 -21_012.lcd
Method File	: LC-MS-ELSD.lcm
Report Format File	: DEFAULT.lsr
Processed by	: System Administrator
Date Processed	: 7/2/2025 3:47:05 PM

Chromatogram

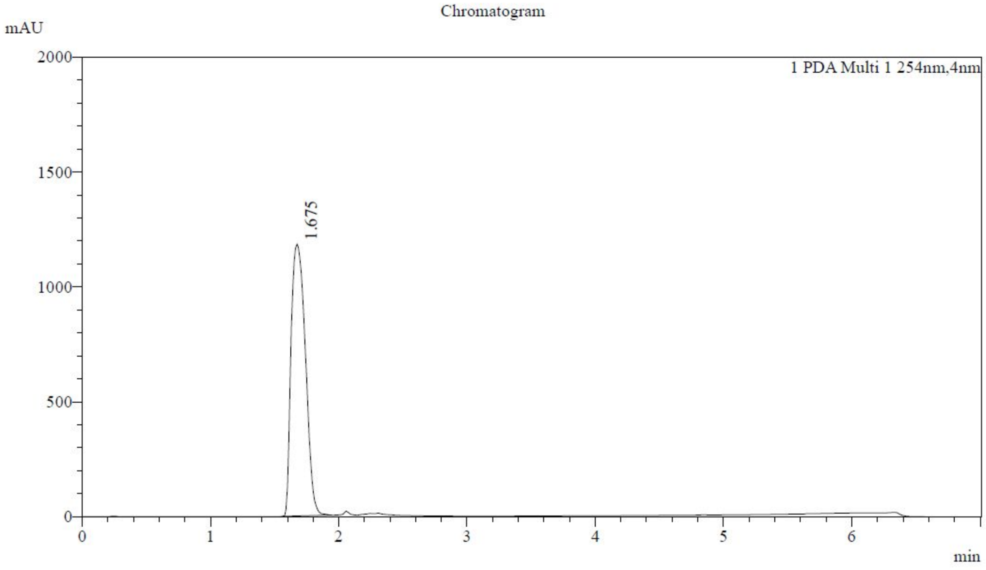

Peak Table

PDA Ch1 254nm

Peak#	Ret. Time	Area	Height	Area%
1	1.675	9202019	1183195	100.000
Total		9202019	1183195	100.000

14. References

1. Hevel, J. M.; Marletta, M. A. Nitric-oxide synthase assays. *Methods Enzymol*. **1994**, 233, 250−258.
2. Do, H. T.; Wang, H.-Y.; Li, H.; Chreifi, G.; Poulos, T. L.; Silverman, R. B. Improvement of cell permeability of human neuronal nitric oxide synthase inhibitors using potent and selective 2- aminopyridine-based scaffolds with a fluorobenzene linker. *J. Med. Chem.* **2017**, *60*, 9360−9375
3. Roman, L. J.; Sheta, E. A.; Martasek, P.; Gross, S. S.; Liu, Q.; Masters, B. S. High level expression of functional rat neuronal nitric oxide synthase in Escherichia coli. *Proc. Natl. Acad. Sci. U. S. A.* **1995**, *92*, 8428−8432.
4. Li, H.; Shimizu, H.; Flinspach, M.; Jamal, J.; Yang, W.; Xian, M.; Cai, T.; Wen, E.Z.; Jia, Q.; Wang, P.G.; Poulos, T.L. The novel binding mode of *N*-alkyl-*N′*-Hydroxy guanidine to neuronal nitric oxide synthase provides mechanistic insights into NO biosynthesis. *Biochemistry* **2002**, 41, 13868–13875.
5. Li, H.; Jamal, J.; Plaza, C.; Pineda, S. H.; Chreifi, G.; Jing, Q.; Cinelli, M. A.; Silverman, R. B.; Poulos, T. L. Structures of human constitutive nitric oxide synthases. *Acta Crystallogr., Sect. D: Biol. Crystallogr.* **2014**, *70*, 2667−2674.
6. Do, H.T.; Li, H.; Chreifi, G.; Poulos, T.L.; Silverman, R.B. Optimization of blood brain barrier permeability with Potent and selective human neuronal nitric oxide synthase inhibitors having a 2-aminopyridine scaffold. *J. Med. Chem.* **2019**, *62*, 2690−2707.
7. Cheng, Y.-C.; Prusoff, W. H. Relationship between the inhibition constant (*K*i) and the concentration of inhibitor which causes 50 percent inhibition (IC50) of an enzymatic reaction. *Biochem. Pharmacol.* **1973**, *22*, 3099−3108.
8. Leber, A.; Hemmens, B.; Klösch, B.; Goessler, W.; Raber, G.; Mayer, B.; Schmidt, K. Characterization of recombinant human endothelial nitric-oxide synthase purified from the yeast Pichia pastoris. *J. Biol. Chem.* **1999**, *274*, 37658−37664.
9. Di, L.; Kerns, E. H.; Fan, K.; McConnell, O. J.; Carter, G. T. High throughput artificial membrane permeability assay for blood− brain barrier. *Eur. J. Med. Chem.* **2003**, *38*, 223−232.
10. Cahlíková, L.; Pérez, D. I.; Štěpánková, Š.; Chlebek, J.; Šafratová, M.; Hošt'álková, A.; Opletal, L. In vitro inhibitory effects of 8-Odemethylmaritidine and undulatine on

acetylcholinesterase and their predicted penetration across the blood−brain barrier. *J. Nat. Prod.* **2015**, *78*, 1189−1192.

11. Könczöl, Á.; Müller, J.; Földes, E.; Béni, Z.; Végh, K.; Kéry, Á.; Balogh, G. T. Applicability of a blood−brain barrier specific artificial membrane permeability assay at the early stage of natural product-based CNS drug discovery. *J. Nat. Prod.* **2013**, *76*, 655−663.

12. Müller, J.; Esső, K.; Dargó, G.; Könczöl, Á.; Balogh, G. T. Tuning the predictive capacity of the PAMPA-BBB model. *Eur. J. Pharm. Sci.* **2015**, *79*, 53−60.

13. Vasu, D.; Li, H.; Hardy, C. D.; Poulos, T. L.; Silverman, R. B. 2-Aminopyridines with a Shortened Amino Sidechain as Potent, Selective, and Highly Permeable Human Neuronal Nitric Oxide Synthase Inhibitors, *Bioorg. Med. Chem.* **2022**, *69*, 116878.

14. Delker, S. L.; Xue, F.; Li, H.; Jamal, J.; Silverman, R. B.; Poulos, T. L.; The Role of Zinc in Isoform Selective Inhibitor Binding to Neuronal Nitric Oxide Synthase. *Biochemistry*. **2010** 49, 10803–10810.

15. McPhillips, T. M.; McPhillips, S. E.; Chiu, H.-J.; Cohen, A. E.; Deacon, A. M.; Ellis, P. J.; Garman, E.; Gonzalez, A.; Sauter, N. K.; Phizackerley, R. P.; Soltis, S. M.; Kuhn, P. Blu-ice and the distributed control system: software for data acquisition and instrument control at macromolecular crystallography beamlines. *J. Synchrotron Radiat.* **2002**, *9*, 401−406.

16. Kang, S.; Li, H.; Martasek, P.; Roman, L.J.; Poulos, T.L.; Silverman, R.B. 2-Aminopyridines with a Truncated Side Chain to Improve Human Neuronal Nitric Oxide Synthase Inhibitory Potency and Selectivity, *J. Med. Chem.* **2015**, *58*, 5548.

17. McCoy, A. J.; Grosse-Kunstleve, R. W.; Adams, P. D.; Winn, M. D.; Storoni, L. C.; Read, R. J. Phaser crystallographic software. *J. Appl. Crystallogr.* **2007**, *40*, 658−674.

18. Battye, T. G. G.; Kontogiannis, L.; Johnson, O.; Powell, H. R.; Leslie, A. G. W. iMOSFLM: a new graphical interface for diffractionimage processing with MOSFLM. *Acta Crystallogr., Sect. D: Biol. Crystallogr.* **2011**, *67*, 271−281.

19. Kabsch, W. XDS. Acta Crystallogr., Sect. D: Biol. Crystallogr. 2010, 66, 125−132.

20. Evans, P. Scaling and assessment of data quality. *Acta Crystallogr., Sect. D: Struct. Biol.* **2006**, *62*, 72−82.

21. Murshudov, G. N.; Vagin, A. A.; Dodson, E. J. Refinement of macromolecular structures by the maximum-likelihood method. *Acta Crystallogr., Sect. D: Struct. Biol.* **1997**, *53*,

240−255.

22. Emsley, P.; Cowtan, K. Coot: model-building tools for molecular graphics. *Acta Crystallogr., Sect. D: Biol. Crystallogr.* **2004**, *60*, 2126−2132.

23. Adams, P. D.; Afonine, P. V.; Bunkoczi, G.; Chen, V. B.; Davis, I. W.; Echols, N.; Headd, J. J.; Hung, L.-W.; Kapral, G. J.; Grosse- Kunstleve, R. W.; McCoy, A. J.; Moriarty, N. W.; Oeffner, R.; Read, R. J.; Richardson, D. C.; Richardson, J. S.; Terwilliger, T. C.; Zwart, P. H. PHENIX: a comprehensive Python-based system for macromolecular structure solution. *Acta Crystallogr., Sect. D: Struct. Biol.* **2010**, *66*, 213−221.

24. Winn, M. D.; Isupov, M. N.; Murshudov, G. N. Use of TLS parameters to model anisotropic displacements in macromolecular refinement. *Acta Crystallogr., Sect. D: Struct. Biol.* **2001**, *57*, 122−133.

25. Liebschner, D.; Afonine, P. V.; Moriarty, N. W.; Poon, B. K.; Sobolev, O. V.; Terwilliger, T. C.; Adams, P. D. Polder maps: improving OMIT maps by excluding bulk solvent. *Acta Crystallogr., Sect. D: Struct. Biol.* **2017**, *73*, 148−157.

26. Madhavi Sastry, G.; Adzhigirey, M.; Day, T.; Annabhimoju, R.; Sherman, W. Protein and ligand preparation: parameters, protocols, and influence on virtual screening enrichments. *J. Comput. Aided Mol. Des.* **2013**, *27*, 221-234.

27. Jacobson, M. P.; Pincus, D. L.; Rapp, C. S.; Day, T. J.; Honig, B.; Shaw, D. E.; Friesner, R. A. A hierarchical approach to all-atom protein loop prediction. *Proteins: Structure, Function, and Bioinformatics* **2004**, *55* (2), 351-367.

28. Lu, C.; Wu, C.; Ghoreishi, D.; Chen, W.; Wang, L.; Damm, W.; Ross, G. A.; Dahlgren, M. K.; Russell, E.; Von Bargen, C. D. OPLS4: Improving force field accuracy on challenging regimes of chemical space. *Journal of chemical theory and computation* **2021**, *17* (7), 4291-4300.

29. Bowers, K. J.; Chow, E.; Xu, H.; Dror, R. O.; Eastwood, M. P.; Gregersen, B. A.; Klepeis, J. L.; Kolossvary, I.; Moraes, M. A.; Sacerdoti, F. D. Scalable algorithms for molecular dynamics simulations on commodity clusters. In *Proceedings of the 2006 ACM/IEEE Conference on Supercomputing*, **2006**; pp 84-es.

30. Patel A, Tong S, Roosan MR, et al. Crosstalk Between nNOS/NO and COX-2 Enhances Interferon-Gamma-Stimulated Melanoma Progression. Cancers (Basel). 2025;17(3):477. Published 2025 Jan 31. doi:10.3390/cancers17030477
